# A widespread riboswitch candidate that controls bacterial genes involved in molybdenum cofactor and tungsten cofactor metabolism

**DOI:** 10.1111/j.1365-2958.2008.06208.x

**Published:** 2008-05

**Authors:** Elizabeth E Regulski, Ryan H Moy, Zasha Weinberg, Jeffrey E Barrick, Zizhen Yao, Walter L Ruzzo, Ronald R Breaker

**Affiliations:** 1 Department of Molecular, Cellular and Developmental Biology, Yale University New Haven, CT 06520, USA; 2 Department of Molecular Biophysics and Biochemistry, Yale University New Haven, CT 06520, USA; 3 Howard Hughes Medical Institute, Yale University New Haven, CT 06520, USA; 4 Departments of Computer Science and Engineering, University of Washington Seattle, WA 98195, USA; 5 Departments of Genome Sciences, University of Washington Seattle, WA 98195, USA

## Abstract

We have identified a highly conserved RNA motif located upstream of genes encoding molybdate transporters, molybdenum cofactor (Moco) biosynthesis enzymes, and proteins that utilize Moco as a coenzyme. Bioinformatics searches have identified 176 representatives in γ-Proteobacteria, δ-Proteobacteria, Clostridia, Actinobacteria, Deinococcus-Thermus species and DNAs from environmental samples. Using genetic assays, we demonstrate that a Moco RNA in *Escherichia coli* associated with the Moco biosynthetic operon controls gene expression in response to Moco production. In addition, we provide evidence indicating that this conserved RNA discriminates against closely related analogues of Moco. These results, together with extensive phylogenetic conservation and typical gene control structures near some examples, indicate that representatives of this structured RNA represent a novel class of riboswitches that sense Moco. Furthermore, we identify variants of this RNA that are likely to be triggered by the related tungsten cofactor (Tuco), which carries tungsten in place of molybdenum as the metal constituent.

## Introduction

Riboswitches are structured RNA domains that selectively bind metabolites or metal ions and function as gene control elements (Mandal and Breaker, 2004; Soukup and Soukup, 2004; Winkler, 2005; Winkler and Breaker 2005). Riboswitches are commonly found in the untranslated regions of eubacterial mRNAs, and usually exert control over gene expression by regulating transcription elongation or translation initiation (Barrick and Breaker, 2007). With few exceptions (Winkler *et al.*, 2004; Barrick *et al.*, 2004), riboswitches are composed of two modular regions, an aptamer domain and an expression platform. The aptamer forms a selective binding pocket for the target metabolite, and this binding event allosterically regulates the folding of the adjoining expression platform to control gene expression. The metabolite-binding pocket of each riboswitch class is highly conserved in sequence and structure, even among representatives identified from distantly related organisms (Barrick and Breaker, 2007).

Riboswitches make excellent targets for discovery using bioinformatics searches. For example, the CMfinder comparative genomics pipeline (Weinberg *et al.*, 2007; Yao *et al.*, 2007) represents one bioinformatics approach that was used to identify novel functional RNA elements. It identifies the putative 5′ untranslated regions (5′ UTRs) of homologous genes, and subsequently examines these regions for evidence of sequence and secondary-structure conservation among the UTRs from various organisms. The patterns of sequence and structure conservation are used to identify additional homologues for refinement of the initial predicted motif. The CMfinder pipeline has identified a number of promising riboswitch candidates that have recently been verified (Wang *et al.*, 2008) or that await verification. One novel motif that has been reported is a large and complex RNA domain that is commonly found upstream of open reading frames (ORFs) coding for proteins involved in molybdenum cofactor (Moco) metabolism (Weinberg *et al.*, 2007).

Moco is a tricyclic pyranopterin (Baugh *et al.*, 1998; Schwarz, 2005) that coordinates an atom of molybdenum (Mo), which is a transition metal located in the same column of the periodic table of the elements as chromium and tungsten. After biosynthesis, Moco is inserted into the active site of Mo-dependent enzymes, which harness the redox activity of Mo to catalyse key reactions in the carbon, nitrogen and sulphur metabolic cycles (Baugh *et al.*, 1998). One hundred and seventy-six instances of this motif have been identified in a wide variety of eubacteria including γ-Proteobacteria, δ-Proteobacteria, Clostridia, Actinobacteria and Deinococcus-Thermus and environmental sequences (Weinberg *et al.*, 2007). This motif displays features typical of metabolite-sensing riboswitches, including extensive sequence conservation, evidence of nucleotide covariation within predicted base-paired elements, and an elaborate assembly of base-paired elements intermixed with conserved unpaired nucleotides.

In this report, we provide evidence that a representative Moco RNA from *Escherichia coli* selectively senses Moco and controls expression of adjacent genes in response to changing levels of this coenzyme. Furthermore, we show that the Moco RNA is involved in regulatory discrimination between Moco and the closely related analogue Tuco, thus providing further evidence that Moco is specifically recognized by aptamers formed by Moco RNAs. These experimental findings, together with a correlation between certain RNA variants and coenzyme metabolism characteristics, suggest that this class of structured RNAs includes distinct Moco- and Tuco-sensing riboswitches.

## Results and discussion

### The Moco motif is widespread and highly conserved

**Fig. 1:**
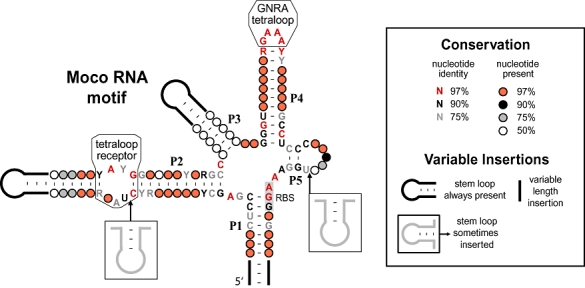
Consensus sequence and secondary structure model of the most common form of Moco RNA motif derived from 176 representatives. R represents A or G and Y represents C or U. Boxed nucleotides denoted RBS are predicted to be the ribosome binding site for the adjacent ORF in some Moco RNA representatives.

When a comparative genomics pipeline (Yao *et al.*, 2007) was used to examine UTRs of genes encoding molybdenum cofactor biosynthesis protein A in γ-Proteobacteria, a motif was identified that exhibits covariation and other features suggestive of a structured RNA (Weinberg *et al.*, 2007). These searches resulted in the identification of 176 instances of this motif (termed ‘Moco RNA’). These sequences were compared by manual alignment using RALEE (Griffiths-Jones, 2005) guided by the putative consensus structure (Fig. S1–4). This alignment reveals that sequence conservation is highest near the central multistem junction of the motif (Fig. 1), which carries most of the nucleotides exhibiting 75–100% sequence conservation.

As many as five base-paired elements (labelled P1–P5) form the secondary structure architecture of Moco RNAs. Within this conserved secondary structure is a GNRA tetraloop (Heus and Pardi, 1991) and a tetraloop receptor (Costa and Michel, 1995) found at the distal end of P4 and centred in a P2 bulge, respectively. The tetraloop sequence in all but five Moco RNAs is GAAA. However, all four Moco RNAs carrying GCAA tetraloops lack the receptor, while only two Moco RNAs with GAAA tetraloops are lacking the receptor. A similar correlation was previously observed for representatives of another new-found RNA motif called the GEMM motif (Weinberg *et al.*, 2007).

**Fig. 2:**
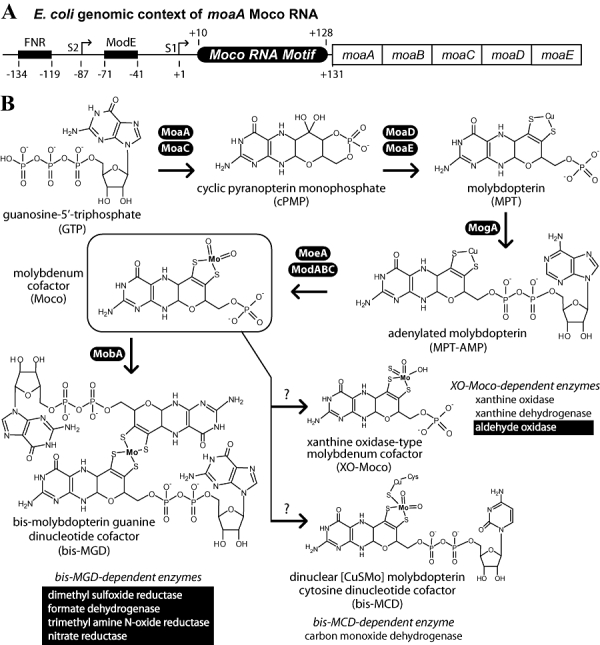
A. Schematic representation of the *moaABCDE* operon of *E. coli*. Nucleotide numbers for the genome region upstream of the *moaA* ORF are established by defining the first transcription start site (S1) as +1 and a second transcription start site (S2) as −87. The approximate locations of the FNR and ModE binding sites are indicated with filled boxes, and the region designated as the Moco RNA motif begins and ends with the terminal nucleotides of P1 (Fig. 1). Numbering system and locations of various features are as reported previously (Anderson *et al.*, 2000). B. Pathway for molybdenum cofactor biosynthesis in eubacteria (Schwarz, 2005). Proteins with abbreviated designations are enzymes in the pathway except for ModABC, which is an ABC-type molybdate transporter. The precise function of MoaB is unknown, but structural studies (Bader *et al.*, 2004; Sanishvili *et al.*, 2004) indicate that it is similar to MogA and therefore is likely to be involved in molybdopterin biosynthesis. Question marks indicate that the biosynthetic enzymes for the conversion are not known. Other proteins listed are enzymes that use Moco derivatives as coenzymes. Proteins whose coding regions are located downstream and near a Moco RNA motif in at least one organism are highlighted with black shading.

Representatives of the Moco motif can be categorized into two groups based on the presence or absence of the P3 stem (Fig. S4). The alignment of all known representatives reveals an apparent aptamer region that is highly conserved, followed by sequences that appear to form different types of expression platforms that are typical of known metabolite-sensing riboswitches. For example, a possible expression platform of the *E. coli* Moco RNA upstream of the *moaABCDE* operon (Fig. 2A, see further discussion below) appears to encompass the ribosome binding site for the downstream ORF within the nucleotides forming the P1 stem. If the Moco RNA indeed is a riboswitch, this arrangement suggests that ligand binding by the aptamer domain will repress gene expression. Examples also exist wherein the putative aptamer domain is found upstream of a stem-loop structure that conforms to the expected sequence and structure of a bacterial intrinsic transcription terminator (Gusarov and Nudler, 1999; Yarnell and Roberts, 1999). Moreover, some organisms carry more than one Moco RNA representative and manifest both types of expression platforms in the same organism.

Although most organisms carry one Moco RNA representative per genome, some organisms whose genomes have been fully sequenced have as many as four or five instances of the motif. The highest numbers of Moco RNA representatives are carried by *Desulfitobacterium hafniense*, which has eight, and *Syntrophomonas wolfei*, which has at least 15 versions (Fig. S4). Interestingly, tandem arrangements of Moco RNA motifs exist in *S. wolfei* and *Geobacter metallireducens.* Although rare, tandem-arranged riboswitches or their subdomains have been identified with other metabolite-sensing riboswitch classes (Famulok, 2004; Mandal and Breaker, 2004; Sudarsan *et al.*, 2006; Stoddard and Batey, 2006). These more complex architectures are used by riboswitches to achieve sophisticated functions such as the ability to respond to multiple signalling inputs (Sudarsan *et al.*, 2006) or are more responsive to smaller changes in ligand concentrations (Mandal *et al.*, 2004; Welz and Breaker, 2007). One operon in *S. wolfei* carries three Moco RNAs in series, and the possible functions of this RNA are discussed in greater detail later in this report.

### The Moco motif is associated with Moco biosynthesis genes

Most known riboswitches function *in cis* to regulate expression of proximal genes in response to binding of their cognate ligand. The ligand sensed by the riboswitch aptamer is typically the final product of the pathway catalysed by the enzyme or enzymes encoded by the mRNA under control. Alternatively, the small molecule ligand is sometimes required as a cofactor or substrate for the gene product whose expression is controlled by the riboswitch. As riboswitches usually use *cis* regulatory mechanisms, the functions of the proteins encoded by the genes located downstream of riboswitches can provide much information about the identity of the ligand.

In our effort to establish the function of Moco RNAs, we considered the functions of genes in the neighbouring genomic locations that carry representatives of this conserved RNA. In γ-Proteobacteria, including *E. coli*, the motif is found upstream of the *moaABCDE* operon (Fig. 2A), which encodes proteins responsible for molybdopterin (MPT) biosynthesis (Schwarz, 2005). In some bacteria, representatives also reside upstream of *modABC*, which encodes a high-affinity molybdate transporter complex (Grunden and Shanmugam, 1997). There are also three species of γ-Proteobacteria (*Haemophilus somnus*, *Haemophilus ducreyi* and *Actinobacillus pleuropneumoniae* serovar 1) where Moco motifs are located upstream of both the MPT biosynthetic operon and a gene predicted to encode MPT oxidoreductase. The Moco motif is also found upstream of different arrangements of genes for Moco biosynthesis enzymes, for high-affinity molybdate transporters, and for enzymes that use Moco to catalyse reactions. In general, these genomic contexts point to Moco or a biosynthetic intermediate of this cofactor as the potential ligand for this riboswitch candidate. Several of the known riboswitch classes sense and respond to other common coenzymes, and therefore we considered Moco as the most likely ligand for this riboswitch candidate.

Molybdenum cofactor biosynthesis is a complex and ancient pathway that is conserved from eubacteria to humans. The only organisms that do not require molybdenum or Moco utilize tungsten and the analogous coenzyme Tuco in its place (Schwarz, 2005). In *E. coli* there are five known operons involved in Moco metabolism: *moa*, *mob*, *mod*, *moe* and *mog* (Shanmugam *et al.*, 1992), which encode a total of 15 proteins. The first two steps of Moco biosynthesis are catalysed by genes encoded by the *moa* operon (Fig. 2B). Specifically, guanosine-5′-triphosphate is converted to cyclic pyranopterin monophosphate (cPMP) in a reaction catalysed by the *S*-adenosylmethionine-dependent enzyme, MoaA, and the uncharacterized homohexameric MoaC (Wuebbens and Rajagopalan, 1995). With a half-life of less than 1 h, cPMP is the most stable Moco biosynthetic intermediate (Santamaria-Araujo *et al.*, 2004). The heterotetrameric MPT synthase complex formed by MoaD and MoaE then catalyses the transfer of two sulphur atoms to cPMP, resulting in a MPT dithiolate (Pitterle *et al.*, 1993).

Moco is subsequently synthesized from MPT by the products of *mogA* and *moeA*, through a series of independent steps involving the adenylation of MPT and insertion of Mo to form molybdenum cofactor (Nichols and Rajagopalan, 2002). In *E. coli*, Moco is further modified to bis-MPT guanine dinucleotide cofactor (bis-MGD) by MobA (Lake *et al.*, 2000). The bis-MGD is then inserted by unknown mechanisms into molybdenum-containing enzymes. In other eubacteria, Moco can be modified to yield other derivatives that also find use as coenzymes (Schwarz, 2005). All of the Moco biosynthetic genes noted above, as well as several genes coding for enzymes that use Moco or its derivatives as coenzymes, are associated with Moco RNA motifs in at least some bacteria (Fig. 2B and Fig. S1–4).

### Biochemical analysis of the structural characteristics of a Moco RNA

**Fig. 3:**
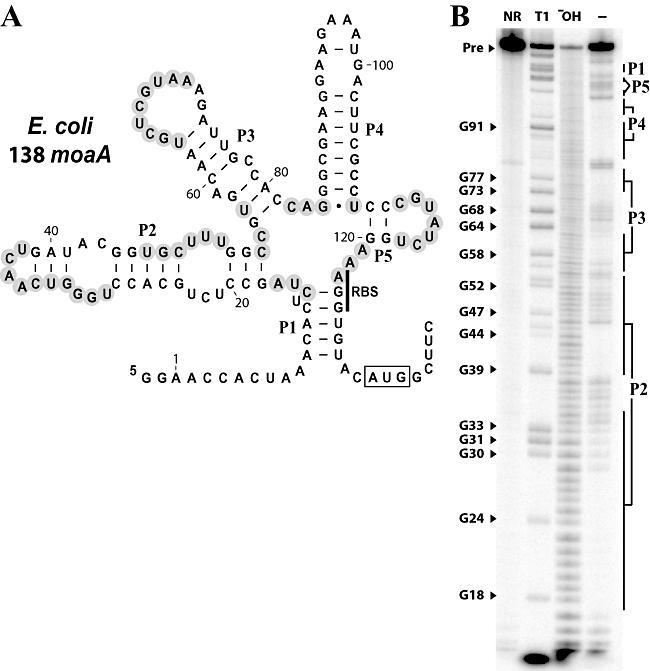
In-line probing assay of a Moco RNA. A. Sequence of the 138 *moaA* RNA of *E. coli* depicted to conform to the secondary structure model predicted from comparative sequence analysis data (Fig. 1A). Nucleotides shaded in grey undergo greater rates of spontaneous 3′ phosphodiester cleavage (from data depicted in B), which typically indicates an elevated level of structural flexibility relative to internucleotide linkages present in stable secondary or tertiary structures. The bar identifies purine nucleotides predicted to serve as a ribosome binding site and the translation start codon for *moaA* is boxed. B. Gel image of the products generated during an in-line probing assay of the 138 *moaA* RNA from *E. coli.* Lanes 1–3 contain the 5′^32^P-labelled precursor RNA (Pre) loaded on the gel either without incubation (no reaction, NR), partially digested by T1 RNase (T1), or subjected to partial alkaline digestion (^–^OH). Lane 4 was loaded with labelled precursor RNA after incubation under in-line probing conditions in the absence (–) of a possible ligand compound. Indicated are regions of the gel containing radiolabelled RNA fragments cleaved after nucleotides predicted to be base paired.

Given the chemical instability of Moco and its biosynthetic intermediates, it was not practical to test these compounds for direct binding interactions *in vitro*. Therefore, we chose to conduct a series of biochemical and genetic experiments to determine if the Moco RNA from *E. coli* has characteristics that are consistent with riboswitch function. To assess the proposed structural model for this Moco RNA representative, we examined a 138 nt RNA construct (termed 138 *moaA*, Fig. 3A) containing the Moco motif extending from 9 nt upstream of the 5′ beginning of the base-pairing P1 element to 10 nt beyond the 3′ end of P1. 138 *moaA* includes a P3 stem (which is absent in some representatives), the predicted ribosome binding site, and AUG translation start codon of the *moaA* ORF. The RNA also carries two additional G residues at the 5′-terminus to facilitate production by *in vitro* transcription. A trace amount of 5′^32^P-labelled 138 *moaA* RNA was subjected to in-line probing analysis (Soukup and Breaker, 1999), which exploits the differences in the frequency of spontaneous cleavage of various internucleotide linkages caused by differences in RNA structure to identify regions of structural rigidity and flexibility.

When separated by polyacrylamide gel electrophoresis (PAGE), the resulting pattern of RNA cleavage products (Fig. 3B) generated during in-line probing is consistent with most of the major secondary structure features that were predicted to form on the basis of comparative sequence analysis (Fig. 1). Specifically, most sites exhibiting a high level of spontaneous phosphoester transfer are localized to the loops of stems P3 and P5, and are also clustered in the junctions that bridge between the predicted base-paired stems. However, the pattern of spontaneous cleavage is not consistent with the secondary structure proposed for much of the P2 stem. Although this stem is predicted to have two internal bulges that could possibly exhibit structural flexibility, some RNA fragments observed are the result of cleavage at nucleotides that are predicted to be base paired.

If Moco RNAs indeed serve as aptamer domains for riboswitches, the in-line probing results suggest that the P2 stem and the junctions carrying several of the highly conserved nucleotides might undergo a conformational change to form a ligand binding pocket. This would be consistent with the behaviour of other known riboswitch aptamers, some of which are observed by in-line probing assays to undergo substantial changes in structure on addition of the target metabolite (Nahvi *et al.*, 2002; Winkler *et al.*, 2002a; Mandal *et al.*, 2003).

### Moco RNA functions as a gene control element

In *E. coli*, there are two promoter sites and accompanying protein factor binding sites that are known to regulate transcription of the *moa* operon (Fig. 2A). The S2 promoter site is dependent on the anaerobic protein transcription factor, FNR (Spiro and Guest, 1990; Anderson *et al.*, 2000). This transcriptional upregulation of the operon in response to anaerobic conditions permits increased biosynthesis of Moco, which certain proteins involved in anaerobic respiration require as a coenzyme. The S1 promoter site of the *moa* operon is activated by molybdate-bound ModE (Anderson *et al.*, 2000), which is a transcription factor that binds molybdate in the cell and upregulates transcription of the *modABCD* operon (Gruden *et al.*, 1999). This ensures that there is sufficient molybdate available in the cell to synthesize Moco. In addition, there is evidence for the control of some genes in the *moa* operon by the copper-responsive factor CueR (Yamamoto and Ishihama, 2005), although the predicted binding site for CueR occurs downstream of the Moco element.

In addition to this multilayer transcription control network, it was known that a 131 nt stretch between the S1 promoter and the *moaA* start codon contributed a third regulatory system (Anderson *et al.*, 2000). Specifically, the presence of this region permits repression of the *moa* operon when Moco is present in cells (Baker and Boxer, 1991; Anderson *et al.*, 2000). The effects of the FNR and ModE transcription factors on *moa* operon expression in *E. coli* can only be observed when this portion of the 5′ UTR is deleted. Intriguingly, this 131 nt region precisely encompasses the Moco RNA motif. Furthermore, no protein regulatory factor has been identified that acts on this portion of the DNA or the corresponding RNA transcript, leaving open the possibility that this portion of the mRNA might serve as a direct sensor for Moco.

**Fig. 4:**
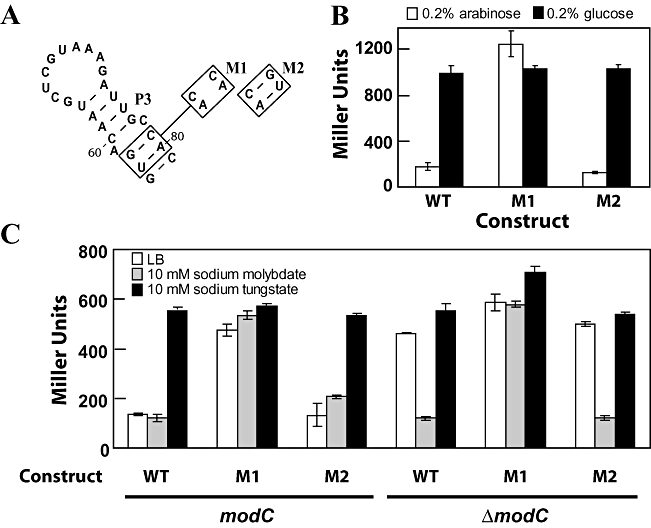
Moco RNA from *E. coli* requires Moco biosynthesis for gene repression. A. Depiction of the P3 region of the *E. coli* 149 *moaA* RNA whose corresponding DNA template was fused to a β-galactosidase reporter gene. M1 and M2 carry nucleotide changes as indicated relative to the wild-type (WT) RNA. B. Expression of β-galactosidase reporter mRNAs fused to the WT and mutant Moco RNAs under different Moco concentrations. Moco concentrations are elevated or reduced by the presence or absence of arabinose in the growth medium, respectively, due to the presence of a transgenic *moa* operon controlled by an arabinose-inducible promoter. C. Dependence of expression of the reporter genes described above on the availability of molybdate or tungstate in cells. The presence (*modC*) or absence (Δ*modC*) of functional molybdate transporter protein in the *E. coli* strain used for the analysis is indicated. Cells were grown in LB containing 0.2% arabinose. Reporter gene expression assays were performed in triplicate (see *Experimental procedures*) and their mean values were plotted, with error bars indicating standard deviation for the replicates.

The architectural features of Moco RNAs, coupled with the known gene control characteristics of the *E. coli moa* operon, strongly suggest that Moco RNA representatives are metabolite-sensing riboswitches. To confirm that a Moco RNA functions as a genetic control element, we used PCR to amplify a portion of the *E. coli* genome corresponding to the wild-type (WT) *moaA* 5′ UTR. This genome segment encodes a 149 nt RNA (termed 149 *moaA*) that begins 9 nt upstream of the 5′ beginning of P1 and extends 21 nt beyond the 3′ end of P1 into the *moaA* coding region. Also, two variant DNAs were generated that express the 149 *moaA* RNA with mutations that disrupt (M1) and subsequently restore (M2) base pairing in the P3 stem (Fig. 4A).

These sequences were cloned into a translational fusion plasmid upstream of, and in-frame with a β-galactosidase reporter gene. The fusion was placed under the control of a constitutively active *lacUV5* promoter to eliminate the effects of upstream anaerobic- and molybdate-dependent promoter regulation that naturally restricts expression of the *moaA* operon. This series of plasmids was placed in a strain of *E. coli* wherein the *moaA* gene was deleted. The resulting transformants were transformed with a plasmid containing the *moaABCDE* operon under the control of an arabinose-inducible and glucose-repressible promoter (Guzman *et al.*, 1995). These doubly transformed strains enabled us to determine the role of the 149 *moaA* RNA on reporter gene expression when Moco concentrations are either high or low.

Cells carrying the WT 149 *moaA* construct exhibited low β-galactosidase gene expression when grown in the presence of arabinose (elevated Moco concentrations expected; Fig. 4B). In contrast, cells with the WT construct exhibit more than a threefold increase in reporter gene expression when grown in the presence of glucose (low Moco concentrations expected). This result suggests that the Moco RNA element represses gene expression, most likely by responding to a small molecule product in the Moco biosynthetic pathway. The function of this Moco RNA as a genetic ‘OFF’ switch is consistent with the mechanism predicted by bioinformatics (see discussion above).

Disruption of the conserved secondary structure of Moco RNAs by the introduction of mutations in the P3 stem (M1) results in derepression of gene expression. In contrast, altering P3 sequences using additional mutations that restore base pairing (M2) also restores gene repression on arabinose-induction of Moco biosynthesis. The disruption of gene control and the return to WT reporter gene expression levels exhibited by these variants indicates that the Moco RNA is a gene control element that requires its conserved secondary structure for activity.

### Molybdenum transport affects Moco RNA gene control function

Molybdate is transported into *E. coli* cells via a high-affinity protein-dependent ABC transporter that is encoded by the *mod* operon (Maupin-Furlow *et al.*, 1995; Grunden and Shanmugam, 1997). In media without molybdate supplementation, WT *E. coli* has an estimated intracellular molybdate concentration of 1.0 µM, whereas the intracellular molybdate concentration in a molybdate transporter deficient strain is estimated at 0.2 µM (Scott and Amy, 1989). However, this molybdate deficiency and the adverse effects it causes can be overcome by the addition of sodium molybdate to the growth medium. Under high extracellular sodium molybdate concentrations, the sulphate transport system is able to import molybdate, bringing the intracellular molybdate concentration of the WT and molybdate-transporter deficient strains to approximately 5 µM (Scott and Amy, 1989; Rosentel *et al.*, 1995).

To determine whether molybdate is critical for gene regulation by 149 *moaA* RNA, we constructed a *modC* knockout strain of *E. coli* to eliminate high-affinity molybdate transporter function. This strain was then transformed with the *moaABCDE*-inducible expression plasmid and the WT and mutant 149 *moaA*–reporter fusion plasmids. When grown in Luria–Bertani (LB) medium (Sambrook and Russell, 2001), which contains trace amounts of molybdate, the *modC* deletion causes the WT 149 *moaA*–reporter fusion to exhibit a high level of gene expression (Fig. 4C). In contrast, when the same strain is grown in LB supplemented with 10 mM sodium molybdate, expression is repressed to a level similar to those of the transformant carrying the functional ModC. This suggests that the molecule needed to trigger gene repression via the Moco RNA is not present at a sufficient concentration unless an adequate amount of molybdate is available in cells.

Interestingly, when cells are grown in LB supplemented with 10 mM sodium tungstate, the WT 149 *moaA*–reporter fusion construct in the transformant carrying the *modC* deletion becomes derepressed. As noted above, tungsten can enter cells and replace molybdenum as the metal ion component of the cofactor. However, if the 149 *moaA* RNA functions as a metabolite-sensing riboswitch (e.g. Moco), it has the ability to discriminate between a compound coordinated to molybdenum and the analogous compound coordinated with tungsten. In all cases, the reporter fusion constructs carrying the disruptive (M1) and compensatory (M2) mutations yield the expected gene expression profiles.

### Mutations in biosynthesis genes suggest Moco is the regulatory ligand

**Fig. 5:**
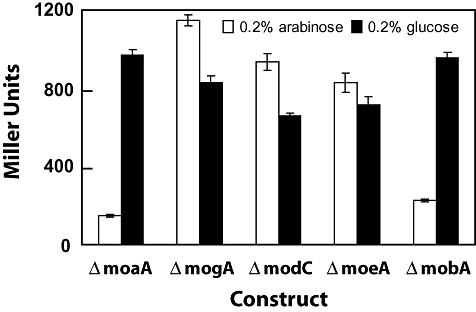
The effects of knockouts of various genes in the *E. coli* Moco biosynthetic pathway on expression of a β-galactosidase reporter gene fused to a DNA template for the WT 149 *moaA* RNA. Each transgenic strain carries a plasmid encoding *E. coli moaABCDE* under the control of an arabinose-inducible promoter.

Given the results above, we speculated that Moco is the regulatory factor that might be directly sensed by Moco RNAs. To provide further evidence that a specific compound in the Moco biosynthetic pathway is responsible for gene expression control, knockout mutations were made to various genes along the pathway. In addition to the *moaA* knockout examined earlier (Fig. 4B), strains with knockouts (Δ) of *mogA, modC* or *moeA* were made and transformed with the *moaABCDE*-inducible expression plasmid and the WT 149 *moaA*–reporter fusion plasmid. The Δ*mogA*, Δ*modC* and Δ*moeA* knockout strains exhibit derepression regardless of the induction of the *moaABCDE* expression plasmid (Fig. 5). The results indicate that these three proteins are essential for the production of the small molecule signal, and that the induction of the *moa* operon cannot compensate for the loss of these proteins.

The gene expression characteristics of the Δ*modC* strain are similar to that observed previously (Fig. 4C), which demonstrates that molybdate transport is required to form the signalling compound. However, the characteristics of the Δ*moeA* strain reveal that molybdate alone is not likely to be the signalling compound. MoeA catalyses the insertion of molybdenum into MPT (the final step in the Moco biosynthetic pathway), and this activity is required for regulation of gene expression by the 149 *moaA* RNA.

For numerous riboswitch classes, the ligand sensed by the riboswitch aptamer is the final coenzyme or metabolic product in the biosynthetic pathway being regulated (Winkler and Breaker, 2005). Moco is a compound that is widespread in all domains of life, while some bacteria generate derivatives of Moco that are inserted into the active sites of Mo-dependent enzymes. As the precise chemical structures of these derivatives vary among various organisms, it seems most reasonable to speculate that the last compound in the pathway that is common to these organisms is more likely to serve as the regulatory signal. Therefore, if Moco RNAs indeed are metabolite-sensing riboswitches, then we hypothesize that Moco would be the most logical ligand for this riboswitch class.

In *E. coli*, before Moco can be inserted into a protein it must be further modified by MobA (Johnson *et al.*, 1991; Iobbi-Nivol *et al.*, 1995). The resulting bis-MGD form of Moco (Fig. 2B) can then be inserted into molybdoproteins. To determine whether a derivative of Moco might be a riboswitch ligand, we constructed a Δ*mobA* strain and assessed its effects on reporter gene expression as described above. When grown in LB with glucose, we see the same derepression as in all other transformants (Fig. 5). However, repression of the reporter fusion construct is restored when the cells are grown in LB supplemented with arabinose. Even without MobA, the cell is able to produce the small molecule required to repress gene expression controlled by the *moaA* 149 RNA. These results are consistent with our hypothesis that Moco is the small molecule responsible for Moco RNA-dependent gene regulation.

### Evidence that Moco RNAs can distinguish between Moco and Tuco

Riboswitches are known for their abilities to differentiate among closely related intermediates within a biosynthetic pathway. Species of Clostridia and several thermophilic archaean species incorporate tungsten rather than molybdenum into MPT, resulting in the formation of the closely related compound Tuco (Johnson *et al.*, 1993). The atomic and ionic radii of Mo and W are virtually identical, as are their electron affinities (Kletzin and Adams, 1996). Importantly for the current study, it has been shown (Buc *et al.*, 1999) that *E. coli* is capable of incorporating W into a final W-bis-MGD cofactor and then inserting the cofactor into a functional trimethylamine *N*-oxide reductase.

In a previous study (Anderson *et al.*, 2000), the apparent repression of the *moaABCDE* operon by the 131 nt upstream region occurs in the presence of Moco, but it is derepressed in the presence of Tuco. This previous observation is consistent with our data (Fig. 4C and data not shown), which demonstrates that cells carrying Moco RNA–reporter fusion constructs are not affected by the addition of tungstate to the medium. Thus, Moco RNAs might be able to distinguish between these two nearly identical cofactors. As the Moco riboswitch candidate 149 *moaA* RNA does not respond to the presence of Tuco in *E. coli*, there must be some distinctive structural features that would allow the binding pocket of the aptamer to discriminate against Tuco. Similarly, there could be distinctive structural features to this class of RNAs when occurring in organisms that use Tuco in place of Moco.

On inspection of the phylogenetic distribution and sequence alignment of Moco RNA representatives, it became apparent that there were two major classes of the motif. Of the 176 examples of the Moco RNA motif, 58 are lacking a P3 stem (Fig. S4). However, the groupings do not break down strictly along phylogenetic divisions, which would be consistent with the propagation of structural variants of a riboswitch aptamer that recognize the same ligand. Therefore, we suspected that there might be some biochemical reason for the structural distinctions and their particular phylogenetic distribution.

Interestingly, the two structural types of Moco RNAs exhibit near perfect correspondence with the utilization of Moco and Tuco among bacteria (Table 1), suggesting that the two RNA types allow the selective recognition of either Moco or Tuco. Organisms that use Moco or Tuco can be tentatively identified by the presence of genes encoding proteins that are predicted to use or transport compounds related to these cofactors. Moco RNAs that include the P3 stem are exclusively associated with genes that encode proteins that synthesize MPT or Moco, and are also associated only with genes encoding enzymes that are known or predicted to use Moco as a coenzyme. In contrast, 17 of the 19 organisms that carry Moco RNA representatives lacking the P3 stem also carry genes that are indicative of Tuco metabolism. Although genes involved in Tuco metabolism have not been identified in *Haemophilus ducreyi* and *Hyphomonas neptunium*, our bioinformatics searches for coenzyme-related genes are unlikely to be comprehensive. Furthermore, the P3-lacking RNAs are almost exclusively associated with genes encoding enzymes associated with MPT biosynthesis (MPT is a precursor to both Tuco and Moco biosynthesis) or with genes encoding enzymes that are known or predicted to use Tuco as a coenzyme.

**Table 1:** Correlation between the presence or absence of P3 and the presence of Moco- or Tuco-related genes.

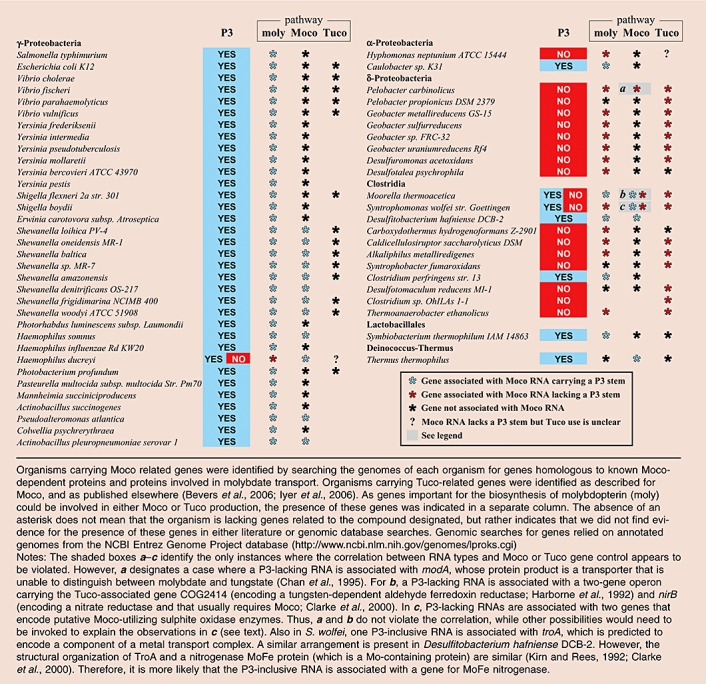

An apparent exception to the segregation of Moco RNA types with Moco and Tuco metabolism is evident for *Pelobacter carbinolicus*, which has a P3-lacking RNA associated with a predicted molybdate transporter (Table 1, annotation ***a***). However, a member of this transporter class is not able to discriminate against tungstate, and therefore the transporter in this organism might actually be important for Tuco biosynthesis. Even in organisms that carry both structural variations of Moco RNAs, the segregation of the P3-inclusive and P3-lacking RNAs to Moco and Tuco metabolic processes, respectively, is largely is maintained. An apparent exception is found with *Moorella thermoacetica* (Table 1, ***b***), where a P3-lacking RNA is associated with a gene (*nirB*) whose function is predicted to be related to Moco metabolism. However, this gene resides in an operon with a predicted Tuco-related gene (see the legend to Table 1), and this provides some rationale for why the P3-lacking RNA is present. The remaining possible exceptions to the correlation are two P3-lacking RNAs in *S. wolfei* (Tables 1, ***c***) that are associated with genes that encode putative Moco-utilizing sulphite oxidase enzymes. If the gene products indeed use Moco, perhaps expression of these genes is repressed when Tuco concentrations increase if the cell has an alternative biosynthetic pathway that uses Tuco. Also, we cannot rule out the possibility that the gene products might actually use Tuco instead of Moco.

Regardless, the striking segregation of the two types of Moco RNAs could be explained by the existence of two Moco RNA types, differing by the presence or absence of the P3 stem, that selectively sense Moco or Tuco respectively. We have deleted the P3 stem from the *E. coli* Moco RNA examined in this study, but this change completely disrupted gene regulation under all growth conditions tested (data not shown). Presumably, this deletion alone is insufficient to switch specificity of Moco RNAs from Moco to Tuco.

As noted above, a triple arrangement of RNA elements is present in an apparent seven-gene operon in *S. wolfei*. The genes in this operon are predicted to encode proteins involved in MPT synthesis, molybdate or tungstate transport, and other genes of unknown function. All three RNAs in this series lack P3 stems. In addition, we observe putative intrinsic transcription terminators following the first two RNA motifs, and a long gap between the third motif and the first ORF in the operon. If each riboswitch indeed functions via a separate expression platform, and if the absence of a P3 stem is indicative of Tuco binding, then the triple arrangement might allow cells to be exceptionally responsive to small changes in Tuco concentration, with greater ‘digital’ gene control character compared with tandem riboswitches describe previously (Welz and Breaker, 2007).

## Conclusions

In *E*. *coli*, transcription of the *moaABCDE* operon is upregulated by both ModE and FNR. However, translation of this operon is prevented when the Moco RNA and Moco are present. ModE and FNR regulation ensure that the *moaABCDE* transcript is only created when conditions needing (anaerobic growth conditions sensed by FNR) and enabling (sufficient levels of molybdate sensed by ModE) Moco production are present in the cell. If the Moco RNA indeed is a riboswitch, it adds an additional layer of regulation. A Moco-sensing riboswitch would ensure that if there are sufficient levels of Moco already present, as indicated by free Moco binding to the *moaABCDE* transcript, then the Moco biosynthetic proteins are not made. This system allows for a rapid yet efficient response to the changing demand for Moco within the cell.

The experimental and bioinformatics data presented herein is consistent with our hypothesis that RNAs conforming to the Moco RNA motif are most likely serving as Moco- or Tuco-sensing riboswitch aptamers. The RNAs have genomic distributions that are consistent with riboswitch function, and the 149 *moaA* RNA exhibits gene control functions that are dependent on its secondary structure. However, the instability of Moco and its metabolic intermediates precludes us from demonstrating direct binding of these coenzymes to the RNA with commonly used techniques such as in-line probing, equilibrium dialysis or fluorescence-based assays. These latter experiments are typically used to prove that ligand binding occurs in the complete absence of protein factors.

Although our experiments have not ruled out the existence of a protein factor that senses Moco or Tuco, several lines of evidence strongly suggest that a protein factor is not involved in metabolite sensing and that the RNA is serving as a direct sensor. Previous studies have identified protein factors responding to oxygen and molybdate that regulate Moco biosynthetic genes in *E. coli* (Spiro and Guest, 1990; Gruden *et al.*, 1999; Anderson *et al.*, 2000). However, no factor has been identified for the regulatory region located immediately upstream of the *moaA* gene in this organism. Moreover, the Moco RNA representatives can be sorted into two distinct architectures (with and without P3), and the distributions of these motif types correlate with an organism's use of Moco, Tuco, or both (Table 1). Such assortment of these structurally distinct RNAs is expected if the RNAs serve as direct sensors of Moco and Tuco.

**Fig. 6:**
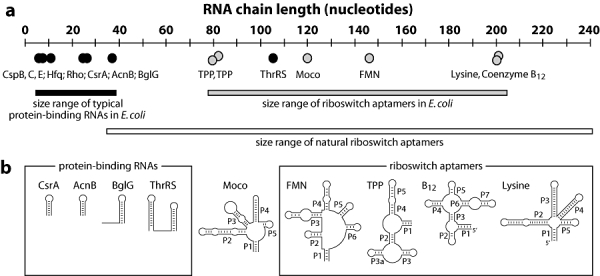
The Moco RNA size and structure is similar to other riboswitch aptamers from *E. coli*. A. Comparison of the length of the *E. coli moaA* Moco aptamer with the lengths of regulatory RNA elements from *E. coli* that either are known to be bound by proteins or function as riboswitch aptamers. RNA targets for ribosomal proteins are not included in this analysis. B. Secondary structure models for the RNAs depicted in (A). RNAs not shown do not have secondary structures established. See the text for references.

Moco RNAs also share several important characteristics with other riboswitch representatives. For example, there are four other classes of riboswitch aptamers known to exist in *E. coli* that respond to coenzyme B_12_ (Nahvi *et al.*, 2004), thiamine pyrophosphate (Winkler *et al.*, 2002a), flavin mononucleotide (Winkler *et al.*, 2002b) and lysine (Sudarsan *et al.*, 2003). As with Moco RNAs (Weinberg *et al.*, 2007), these riboswitches are all large compared with non-riboswitch gene control elements composed of RNA (Fig. 6), and they all have high levels of nucleotide sequence and structural conservation (Barrick and Breaker, 2007). Moco RNAs and the known riboswitch aptamers from *E. coli* are larger than 100 nt, and carry four to six stems that exhibit evidence of nucleotide covariation even in representatives from distantly related organisms.

The known metabolic regulatory proteins from *E. coli* that bind RNA, such as CspA (Phadtare and Inouye, 1999), BglG (Houman *et al.*, 1990) and others (Richardson and Richardson, 1996; Liu *et al.*, 1997; Tang and Guest, 1999; Torres-Larios *et al.*, 2002; Vecerek *et al.*, 2005), typically recognize far smaller stretches of RNA. If there is any secondary structure present in these protein-binding sequences, then it generally consists of a single hairpin. One exception to this trend is the ThrRS RNA element (Torres-Larios *et al.*, 2002), which has evolved a structure to favour binding by an aminoacyl–tRNA synthetase that normally binds a large structured tRNA. In contrast to most protein-binding RNA motifs, the Moco RNA from the *E. coli moaA* RNA spans 138 nt and forms at least five conserved stem-loop elements. Thus, the large and extensively conserved structures of Moco RNAs are more characteristic of the known riboswitch RNAs from *E. coli* than of most RNA gene control elements that are bound by protein factors. These characteristics strongly implicate Moco RNAs as members of a new-found class of metabolite-sensing riboswitches.

## Experimental procedures

### Oligonucleotides and chemicals

Synthetic DNAs were synthesized by the Howard Hughes Medical Institute Keck Foundation Biotechnology Resource Center at Yale University. The following chemicals were purchased from Sigma-Aldrich: carbenicillin, chloramphenicol, kanamycin, l-arabinose, d-glucose, glycerol, sodium molybdate and sodium tungstate.

### Bacterial strains and media

The *E. coli* strain DJ480 (MG1655 Δ*lacX74*) (Cabrera and Jin, 2001) was obtained from D.J. Jin (National Institutes of Health) and NEB5α cells were obtained from New England Biolabs. Plasmids corresponding to the Wanner gene disruption set (pKD46, pKD13 and pCP20; Datsenko and Wanner, 2000) were obtained from the *E. coli* Genetic Stock Center, Yale University. Media supplements were added as noted for each experiment: carbenicillin, 50 µg ml^−1^; chloramphenicol, 5 µg ml^−1^; kanamycin 50 µg ml^−1^; l-arabinose, 0.2% (w/v); d-glucose, 0.2% (w/v). Glycerol, 0.2% (w/v), is added to the medium when arabinose supplementation is applied (Guzman *et al.*, 1995).

### Preparation of Moco RNA

The 138 *moaA* RNA construct used for in-line probing was prepared by *in vitro* transcription using a template generated by successive PCR amplification reactions. The intergenic region upstream of *moaA* was amplified by PCR from *E. coli* K12 genomic DNA using the primers 5′-GAATTCCCTGGAGTCAGATTATCCGC and 5′-CTGGATCCAGTTGTGAAGCCATGTACAC. Following digestion by EcoRI and BamHI, the PCR product was cloned into pRS414 and the integrity of the resulting plasmid was confirmed by DNA sequencing. This plasmid was used as the template for PCR amplification of the DNA construct encoding the 138 *moaA* construct (including the GG addition corresponding to the 5′ terminus of the RNA transcript) where the primer for the non-template strand included the promoter sequence for T7 RNA polymerase. RNA molecules were prepared by transcription *in vitro* using T7 RNA polymerase and purified using denaturing PAGE as described previously (Roth *et al.*, 2006).

### In-line probing analysis of 138 *moaA* RNA

Approximately 40 pmol of RNA prepared by *in vitro* transcription was dephosphorylated with alkaline phosphatase (Roche Diagnostics) and 20 pmol was radiolabelled with [γ-^32^P]-ATP using T4 polynucleotide kinase (New England Biolabs) following protocols supplied by the manufacturers. In-line probing reactions using radiolabelled RNAs purified by PAGE were assembled as previously described (Mandal *et al.*, 2003) and were incubated for 40 h at 25°C in a 10 µl volume containing 50 mM Tris-HCl (pH 8.3 at 23°C), 100 mM KCl, 20 mM MgCl_2_ and ∼5 nM precursor RNA. The resulting RNA fragments were separated by using 10% denaturing PAGE, and were imaged using a Molecular Dynamics PhosphorImager and quantified using ImageQuaNT software.

### Generation of *moaA*, *mogA*, *modC*, *moeA* and *mobA* knockouts

*Escherichia coli* DJ480 strains with specific gene deletions were generated using the λ-red recombination method and appropriate PCR products as described elsewhere (Datsenko and Wanner, 2000). In brief, mutants were made by PCR amplification using plasmid pKD13 and the following primers: *moaA*, 5′-AGGAAGAAATGACTTCGCCTCCCGTATCTGGAAAGGTGTACATGGCTATTCCGGGGATCCGTCGACC and 5′-ACCTGACTCATCTGATCTCTCCTTTTGACGTTTTA-GCCGCCAATGTACGATGTAGGCTGGAGCTGCTTCG; *mogA*, 5′-TGTATCATTCTGTTTAACGAGACTGTTTAAACGGAAAAATCTTGATGAATATTCCGGGGATCCGTCGACC and 5′-TTGAGATCCCCCCGCTCGGGGGGATTTTTTTATTCGCTAACGTCGCGTCTTGTAGGCTGGAGCTGCTTCG; *moeA*, 5′-GACATAATAGGCAAATTCGATTTTGCCTCCGCAGGA-GTGTTTTCATGGAAATTCCGGGGATCCGTCGACC and 5′-CAGCATCTCCTGATCG-CTGAGTTCCGCCATTACAGGCCTCCGAACAACGCTGTAGGCTGGAGCTGCTTCG; *modC*, 5′-GAATGGCTGGCCAGAATCAGCCGTGAACGGGCGGGGCGCTAATCATGCTG-ATTCCGGGGATCCGTCGACC and 5′-TTGCCCAGTTCATTTATAGCCACCTGAT-TTAATCAGGCGGTTATCGACACTGTAGGCTGGAGCTGCTTCG; *mobA*, 5′-GACACGTTAGCAGGGTCAATCCCACAATAAAAGAGGCGATATCGGTGAATATTCCGGGGATCCGTCGACC and 5′-ACTCCACGCGGCAAAGGCGAGTAACGGTATCATCGTTTTTCCTGCCATCGTGTAGGCTGGAGCTGCTTCG.

The resulting ∼1.4 kb long PCR products, containing a kanamycin resistance cassette flanked by FLP recognition target sites and identical 50 nt sequence identities to adjacent chromosomal sequences, were purified and subjected to DpnI digestion. DJ480 cells were transformed with the Red helper plasmid, pKD46, encoding the Red recombinase from phage λ that promotes homologous recombination. Transformants were selected for ampicillin resistance. DJ480/pKD46 cells grown with l-arabinose were transformed with the gene-specific/pKD13 PCR product. Recombination of the PCR products into the chromosome of DJ480 was determined by selection for kanamycin-resistant transformants. All resulting deletion mutants were verified by PCR with both internal primers specific for the kanamycin cassette (k1 5′-CAGTCATAGCCGAATAGCCT and k2 5′-CGGTGCCCTGAATGAACTGC) and flanking gene-specific primers (*moaA* 5′-CGCTAGTATCGGCATAACCAC and 5′-GAATCGCGCAGATAGTGACCG, *mogA* 5′-GCTACCTCTTCTGAAGCCTGTCTGT and 5′-ATGGGTGAAGTACTGAACGAGCAGT, *moeA* 5′-GATGGATATGGCATGTAAAGGCAGG and 5′-AGCACGCGAGAATCTTTCAGCGCCT, *modC* 5′-GAGCGGCGAGACTGTGCATTA and 5′-GCAACGCCGATGACGCGGTA, and *mobA* 5′-CTTCATTCAGACGTTTACATTTCATAG and 5′-CATCCATATCATGGTGCGTATGCTTA). The kanamycin cassette was then eliminated by site-specific recombination by using the FLP plasmid pCP20. The resulting kanamycin sensitive transformants were verified by PCR with the same primers used for the pre-FLP verification.

### Construction of β-galactosidase translational fusion and mutants

Preparation of a Moco RNA–reporter fusion construct driven by the constitutively active *lac* UV5 promoter lacking the operator binding site was achieved as follows. The nucleotide sequence from 1 to 145 relative to the S1 transcription start site for the *E. coli moaABCDE* operon (Fig. 2A) was amplified by PCR from *E. coli* strain K12 using the primer 5′-GAATTCCTCATTAGGCACCCCAGGCTTTACACTTTATGCTTCCGGCTCGTATAATGTGTGGAACCACTAAACACTCTAGCCTC, containing the 56 nt *lac* UV5 promoter sequence (underlined) (Dickson *et al.*, 1975), and the primer 5′-CTGGATCCAGTTGTGAAGCCATGTACAC. Following digestion with EcoRI and BamHI, the amplification product was cloned into pRS414 plasmid (Simons *et al.*, 1987) which contains a promoterless copy of *lacZ*, resulting in the in-frame fusion between the ninth codon of *lacZ* and the fifth codon of *moaA*. NEB-5α cells were transformed with the resulting plasmid. Transformants were selected for ampicillin resistance and the integrity of the *lac* UV5–Moco motif–*lacZ* translational fusion was confirmed by DNA sequencing. All site-directed mutations were introduced into the Moco riboswitch using the QuikChange site-directed mutagenesis kit (Stratagene) and the appropriate mutagenic DNA primers. All mutations were confirmed by DNA sequencing.

### Construction of MoaABCDE pBAD33 overexpression plasmid

The *moaABCDE* operon was cloned into the pBAD33 plasmid (Guzman *et al.*, 1995) to enable arabinose-inducible control of Moco levels within the cell. A 2.7 kb KpnI-HindIII *moaABCDE* fragment was amplified by PCR from *E. coli* K12 using primers 5′-CTAGGTACCTCTGGAAAGGTGTACATGGCTTCACAAC and 5′-TAGAAGCTTAACTACCAGCGTTTTGCCGCCTGCTG. The PCR product was purified by agarose gel electrophoresis and subjected to KpnI and HindIII digestion, as was pBAD33. The digested fragment was then ligated into pBAD33 downstream of the arabinose-inducible promoter and transformed into NEB-5α cells. Transformants were selected for chloramphenicol resistance and the fusion was confirmed by DNA sequencing.

### Generation of mutant strains

The *moaA*, *mogA*, *modC*, *moeA* and *mobA* knockout DJ480 strains were transformed simultaneously with both the *lac* UV5–Moco motif–*lacZ* fusion pRS414 plasmid containing WT or mutant 149 moaA–reporter fusions and the *moaABCDE* pBAD33 overexpression plasmid. Transformants were selected for both ampicillin and chloramphenicol resistance.

### *In vivo* analysis of gene control by the 149 *moaA* RNA from *E. coli*

To measure gene expression from *lacZ* in the WT and mutant reporter strains of *E. coli*, the cells were grown in a 14 ml culture vial with shaking overnight at 37°C in 3 ml of LB with supplementation as indicated. The cells were then diluted to an OD_600_ of 0.05 in 150 μl of LB in 96-well plates and grown to an A_600_ between 0.5 and 0.7 with shaking at 37°C, and then harvested for gene expression experiments. β-Galactosidase activity was assayed using a 96-well microplate protocol described elsewhere (Blount *et al.*, 2007).

### Bioinformatics analysis of Moco and Tuco metabolism

The assignment of Moco and Tuco metabolism to various organisms and the assignment of Moco RNA motif types to genes involved in three processes (MPT biosynthesis, Moco biosynthesis or use, and Tuco biosynthesis or use; Table 1) were made by sequence homology analysis. To identify organisms that use Tuco, but that have not been experimentally demonstrated to do so, we used blast to search for genes homologous to *tupA* and *tupB*. These genes have been shown in *Eubacterium acidaminophilum* to encode transporters specific for tungstate (Makdessi *et al.*, 2001). Tuco metabolism in *Pelobacter carbinolicus* and *Desulfotalea psychrophilia* were assigned based on the presence of *wtpA* homologues, which have been shown to be distinct but selective transporters of tungstate (Bevers *et al.*, 2006). Assignments of Moco RNA motifs to the three processes listed above were made by examining the annotated functions of protein products of adjoining genes, or by assignment of functions using sequence homology if annotation was not available.

## Supplementary material

This material is available as part of the online article from: http://www.blackwell-synergy.com/doi/abs/10.1111/j.1365-2958.2008.06208.x (This link will take you to the article abstract).
