# Supplementary material for: A widespread riboswitch candidate that controls bacterial genes involved in molybdenum cofactor and tungsten cofactor metabolism
Source: Mol Microbiol. 2008 May;68(4):918–32. doi: 10.1111/j.1365-2958.2008.06208.x (PMC2408646; doi:10.1111/j.1365-2958.2008.06208.x)
Supplement: Supplementary file 1 [file mmi0068-0918-SD1.pdf]

Supplementary data:
A widespread riboswitch candidate that controls bacterial genes involved in molybdenum cofactor metabolism

Elizabeth E. Regulski[1], Ryan H. Moy[2], Zasha Weinberg[1], Jeffrey E. Barrick[2,3], Zizhen Yao[4], Walter L. Ruzzo[4,5] and Ronald R. Breaker[1,2,3]

[1] Department of Molecular, Cellular and Developmental Biology,
[2] Department of Molecular Biophysics and Biochemistry,
[3] Howard Hughes Medical Institute,
Yale University, New Haven, Connecticut 06520.
[4] Department of Computer Science and Engineering,
[5] Department of Genome Sciences,
University of Washington, Seattle, Washington 98195.

Correspondence should be addressed to R.R.B. (ronald.breaker@yale.edu)

Note: the presentation of this supplementary data on Moco RNA sequences follows the pattern of our presentation of previous conserved RNA motifs [1].

Contents

Supplementary Figure S1: taxonomy of Moco RNAs **2**

Supplementary Figure S2: gene context of Moco RNAs **5**

Supplementary Figure S3: conserved domains present in genes downstream of Moco RNAs **11**

Supplementary Figure S4: Multiple sequence alignment of Moco RNAs with 3′ flanking sequences **13**

Supplementary Figure S5: alignment of sequences containing P3 **29**

Supplementary Figure S6: alignment of sequences lacking P3 **40**

Supplementary Figure S1: taxonomy of Moco RNAs

The taxonomy of each organism containing a putative Moco RNA is listed, with abbreviations (e.g., "Eco-1-1") used to denote that hit in later figures.

**abbrev. of hits**	**taxonomy of species**
Aba-1-1	Acidobacteria Acidobacteriales Acidobacteriaceae *Acidobacteria bacterium* Ellin345
Sth-1-1	Actinobacteria *Symbiobacterium thermophilum* IAM 14863
Tth-1-1	Deinococcus-Thermus Deinococci Thermales Thermaceae *Thermus thermophilus* HB27
Tth-2-1	Deinococcus-Thermus Deinococci Thermales Thermaceae *Thermus thermophilus* HB8
Bce-1-1	Firmicutes Bacillales Bacillaceae Bacillus Bacilluscereus group *Bacillus cereus subsp. cytotoxis* NVH 391-98
Esi-1-1	Firmicutes Bacillales Bacillaceae *Exiguobacterium sibiricum* 255-15
Ame-1-1 to Ame-1-2	Firmicutes Clostridia Clostridiales Clostridiaceae *Alkaliphilus metalliredigenes* QYMF
Cpe-1-1	Firmicutes Clostridia Clostridiales Clostridiaceae *Clostridium perfringens* ATCC 13124
Cpe-2-1	Firmicutes Clostridia Clostridiales Clostridiaceae *Clostridium perfringens* SM101
Cpe-3-1	Firmicutes Clostridia Clostridiales Clostridiaceae *Clostridium perfringens str.* 13
Csp-1-1	Firmicutes Clostridia Clostridiales Clostridiaceae *Clostridium sp.* OhILAs
Chy-1-1	Firmicutes Clostridia Clostridiales Peptococcaceae *Carboxydothermus hydrogenoformans* Z-2901
Dha-1-1 to Dha-1-8	Firmicutes Clostridia Clostridiales Peptococcaceae *Desulfitobacterium hafniense* DCB-2
Dha-2-1 to Dha-2-7	Firmicutes Clostridia Clostridiales Peptococcaceae *Desulfitobacterium hafniense* Y51
Dre-1-1 to Dre-1-3	Firmicutes Clostridia Clostridiales Peptococcaceae *Desulfotomaculum reducens* MI-1
Csa-1-1	Firmicutes Clostridia Clostridiales Syntrophomonadaceae *Caldicellulosiruptor saccharolyticus* DSM 8903
Swo-1-1 to Swo-1-15	Firmicutes Clostridia Clostridiales Syntrophomonadaceae *Syntrophomonas wolfei subsp. wolfei str.* Goettingen
Mth-1-1 to Mth-1-5	Firmicutes Clostridia Thermoanaerobacteriales Thermoanaerobacteriaceae Moorella group *Moorella thermoacetica* ATCC 39073
Tet-1-1 to Tet-1-2	Firmicutes Clostridia Thermoanaerobacteriales Thermoanaerobacteriaceae *Thermoanaerobacter ethanolicus* ATCC 33223
Tet-2-1 to Tet-2-2	Firmicutes Clostridia Thermoanaerobacteriales Thermoanaerobacteriaceae *Thermoanaerobacter ethanolicus* X514
Csp-2-1	α-proteobacteria Caulobacterales Caulobacteraceae *Caulobacter sp.* K31
Hne-1-1	α-proteobacteria Rhodobacterales Hyphomonadaceae *Hyphomonas neptunium* ATCC 15444
Dps-1-1	δ-proteobacteria Desulfobacterales Desulfobulbaceae *Desulfotalea psychrophila* LSv54
Dac-1-1 to Dac-1-3	δ-proteobacteria Desulfuromonadales Desulfuromonadaceae *Desulfuromonas acetoxidans* DSM 684
Gme-1-1 to Gme-1-4	δ-proteobacteria Desulfuromonadales Geobacteraceae *Geobacter metallireducens* GS-15
Gsp-1-1 to Gsp-1-5	δ-proteobacteria Desulfuromonadales Geobacteraceae *Geobacter sp.* FRC-32
Gsu-1-1	δ-proteobacteria Desulfuromonadales Geobacteraceae *Geobacter sulfurreducens* PCA
Gur-1-1 to Gur-1-4	δ-proteobacteria Desulfuromonadales Geobacteraceae *Geobacter uraniumreducens* Rf4
Pca-1-1 to Pca-1-5	δ-proteobacteria Desulfuromonadales Pelobacteraceae *Pelobacter carbinolicus* DSM 2380
Ppr-1-1 to Ppr-1-2	δ-proteobacteria Desulfuromonadales Pelobacteraceae *Pelobacter propionicus* DSM 2379
Sfu-1-1	δ-proteobacteria Syntrophobacterales Syntrophobacteraceae *Syntrophobacter fumaroxidans* MPOB
Ahy-1-1	γ-proteobacteria Aeromonadales Aeromonadaceae *Aeromonas hydrophila subsp. hydrophila* ATCC 7966
Ama-1-1	γ-proteobacteria Alteromonadales Alteromonadaceae *Alteromonas macleodii* 'Deep ecotype'
Cps-1-1	γ-proteobacteria Alteromonadales Colwelliaceae *Colwellia psychrerythraea* 34H
Pat-1-1 to Pat-1-2	γ-proteobacteria Alteromonadales Pseudoalteromonadaceae *Pseudoalteromonas atlantica* T6c
Pin-1-1	γ-proteobacteria Alteromonadales Psychromonadaceae *Psychromonas ingrahamii* 37
Sam-1-1	γ-proteobacteria Alteromonadales Shewanellaceae *Shewanella amazonensis* SB2B
Sba-1-1	γ-proteobacteria Alteromonadales Shewanellaceae *Shewanella baltica* OS155
Sba-2-1	γ-proteobacteria Alteromonadales Shewanellaceae *Shewanella baltica* OS195
Sde-1-1	γ-proteobacteria Alteromonadales Shewanellaceae *Shewanella denitrificans* OS217
Sfr-1-1	γ-proteobacteria Alteromonadales Shewanellaceae *Shewanella frigidimarina* NCIMB 400
Son-1-1	γ-proteobacteria Alteromonadales Shewanellaceae *Shewanella oneidensis* MR-1
Spe-1-1	γ-proteobacteria Alteromonadales Shewanellaceae *Shewanella pealeana* ATCC 700345
Spu-1-1	γ-proteobacteria Alteromonadales Shewanellaceae *Shewanella putrefaciens* CN-32
Ssp-1-1	γ-proteobacteria Alteromonadales Shewanellaceae *Shewanella sp.* ANA-3
Ssp-2-1	γ-proteobacteria Alteromonadales Shewanellaceae *Shewanella sp.* MR-4
Ssp-3-1	γ-proteobacteria Alteromonadales Shewanellaceae *Shewanella sp.* MR-7
Ssp-4-1	γ-proteobacteria Alteromonadales Shewanellaceae *Shewanella sp.* PV-4
Ssp-5-1 to Ssp-5-2	γ-proteobacteria Alteromonadales Shewanellaceae *Shewanella sp.* W3-18-1
Swo-2-1	γ-proteobacteria Alteromonadales Shewanellaceae *Shewanella woodyi* ATCC 51908
Esp-1-1	γ-proteobacteria Enterobacteriales Enterobacteriaceae *Enterobacter sp.* 638
Eco-1-1	γ-proteobacteria Enterobacteriales Enterobacteriaceae *Escherichia coli* 101-1
Eco-2-1	γ-proteobacteria Enterobacteriales Enterobacteriaceae *Escherichia coli* 536
Eco-3-1	γ-proteobacteria Enterobacteriales Enterobacteriaceae *Escherichia coli* 53638
Eco-4-1	γ-proteobacteria Enterobacteriales Enterobacteriaceae *Escherichia coli* APEC O1
Eco-5-1	γ-proteobacteria Enterobacteriales Enterobacteriaceae *Escherichia coli* B171

Eco-6-1	γ-proteobacteria Enterobacteriales Enterobacteriaceae *Escherichia coli* B7A
Eco-7-1	γ-proteobacteria Enterobacteriales Enterobacteriaceae *Escherichia coli* CFT073
Eco-8-1	γ-proteobacteria Enterobacteriales Enterobacteriaceae *Escherichia coli* E110019
Eco-9-1	γ-proteobacteria Enterobacteriales Enterobacteriaceae *Escherichia coli* E22
Eco-10-1	γ-proteobacteria Enterobacteriales Enterobacteriaceae *Escherichia coli* E24377A
Eco-11-1	γ-proteobacteria Enterobacteriales Enterobacteriaceae *Escherichia coli* F11
Eco-12-1	γ-proteobacteria Enterobacteriales Enterobacteriaceae *Escherichia coli* HS
Eco-13-1	γ-proteobacteria Enterobacteriales Enterobacteriaceae *Escherichia coli* K12
Eco-14-1	γ-proteobacteria Enterobacteriales Enterobacteriaceae *Escherichia coli* O157:H7 EDL933
Eco-15-1	γ-proteobacteria Enterobacteriales Enterobacteriaceae *Escherichia coli* O157:H7 str. Sakai
Eco-16-1	γ-proteobacteria Enterobacteriales Enterobacteriaceae *Escherichia coli* UTI89
Eco-17-1	γ-proteobacteria Enterobacteriales Enterobacteriaceae *Escherichia coli* W3110
Eca-1-1	γ-proteobacteria Enterobacteriales Enterobacteriaceae Pectobacterium *Erwinia carotovora subsp. atroseptica* SCRI1043
Plu-1-1	γ-proteobacteria Enterobacteriales Enterobacteriaceae *Photorhabdus luminescens subsp. laumondii* TTO1
Sen-1-1	γ-proteobacteria Enterobacteriales Enterobacteriaceae *Salmonella enterica subsp. enterica serovar* Paratyphi A str. ATCC
Sen-3-1	γ-proteobacteria Enterobacteriales Enterobacteriaceae *Salmonella enterica subsp. enterica serovar* Typhi str. CT18
Sen-2-1	γ-proteobacteria Enterobacteriales Enterobacteriaceae *Salmonella enterica subsp. enterica serovar* Typhi Ty2
Sty-1-1	γ-proteobacteria Enterobacteriales Enterobacteriaceae *Salmonella typhimurium* LT2
Spr-1-1	γ-proteobacteria Enterobacteriales Enterobacteriaceae *Serratia proteamaculans* 568
Sbo-1-1	γ-proteobacteria Enterobacteriales Enterobacteriaceae *Shigella boydii* BS512
Sbo-2-1	γ-proteobacteria Enterobacteriales Enterobacteriaceae *Shigella boydii* Sb227
Sdy-1-1	γ-proteobacteria Enterobacteriales Enterobacteriaceae *Shigella dysenteriae* Sd197
Sfl-1-1	γ-proteobacteria Enterobacteriales Enterobacteriaceae *Shigella flexneri* 2a str. 2457T
Sfl-2-1	γ-proteobacteria Enterobacteriales Enterobacteriaceae *Shigella flexneri* 2a str. 301
Sfl-3-1	γ-proteobacteria Enterobacteriales Enterobacteriaceae *Shigella flexneri* 5 str. 8401
Sso-1-1	γ-proteobacteria Enterobacteriales Enterobacteriaceae *Shigella sonnei* Ss046
Sgl-1-1	γ-proteobacteria Enterobacteriales Enterobacteriaceae *Sodalis glossinidius str. 'morsitans'*
Ybe-1-1	γ-proteobacteria Enterobacteriales Enterobacteriaceae *Yersinia bercovieri* ATCC 43970
Yfr-1-1	γ-proteobacteria Enterobacteriales Enterobacteriaceae *Yersinia frederiksenii* ATCC 33641
Yin-1-1	γ-proteobacteria Enterobacteriales Enterobacteriaceae *Yersinia intermedia* ATCC 29909
Ymo-1-1	γ-proteobacteria Enterobacteriales Enterobacteriaceae *Yersinia mollaretii* ATCC 43969
Ype-1-1	γ-proteobacteria Enterobacteriales Enterobacteriaceae *Yersinia pestis* Angola
Ype-2-1	γ-proteobacteria Enterobacteriales Enterobacteriaceae *Yersinia pestis* Antiqua
Ype-7-1	γ-proteobacteria Enterobacteriales Enterobacteriaceae *Yersinia pestis biovar* Microtus str. 91001
Ype-8-1	γ-proteobacteria Enterobacteriales Enterobacteriaceae *Yersinia pestis biovar* Orientalis str. IP275
Ype-3-1	γ-proteobacteria Enterobacteriales Enterobacteriaceae *Yersinia pestis* CO92
Ype-4-1	γ-proteobacteria Enterobacteriales Enterobacteriaceae *Yersinia pestis* FV-1
Ype-5-1	γ-proteobacteria Enterobacteriales Enterobacteriaceae *Yersinia pestis* KIM
Ype-6-1	γ-proteobacteria Enterobacteriales Enterobacteriaceae *Yersinia pestis* Nepal516
Yps-1-1	γ-proteobacteria Enterobacteriales Enterobacteriaceae *Yersinia pseudotuberculosis* IP 31758
Yps-2-1	γ-proteobacteria Enterobacteriales Enterobacteriaceae *Yersinia pseudotuberculosis* IP 32953
Apl-1-1 to Apl-1-2	γ-proteobacteria Pasteurellales Pasteurellaceae *Actinobacillus pleuropneumoniae serovar* 1 str. 4074
Asu-1-1	γ-proteobacteria Pasteurellales Pasteurellaceae *Actinobacillus succinogenes* 130Z
Hdu-1-1 to Hdu-1-2	γ-proteobacteria Pasteurellales Pasteurellaceae *Haemophilus ducreyi* 35000HP
Hin-1-1	γ-proteobacteria Pasteurellales Pasteurellaceae *Haemophilus influenzae* 86-028NP
Hin-2-1	γ-proteobacteria Pasteurellales Pasteurellaceae *Haemophilus influenzae* R2846
Hin-3-1	γ-proteobacteria Pasteurellales Pasteurellaceae *Haemophilus influenzae* R2866
Hin-4-1	γ-proteobacteria Pasteurellales Pasteurellaceae *Haemophilus influenzae* Rd KW20
Hso-1-1 to Hso-1-2	γ-proteobacteria Pasteurellales Pasteurellaceae Histophilus *Haemophilus somnus* 129PT
Hso-2-1 to Hso-2-2	γ-proteobacteria Pasteurellales Pasteurellaceae Histophilus *Haemophilus somnus* 2336
Mha-1-1 to Mha-1-2	γ-proteobacteria Pasteurellales Pasteurellaceae *Mannheimia haemolytica* PHL213
Msu-1-1	γ-proteobacteria Pasteurellales Pasteurellaceae *Mannheimia succiniciproducens* MBEL55E
Pmu-1-1	γ-proteobacteria Pasteurellales Pasteurellaceae *Pasteurella multocida subsp. multocida str.* Pm70
Rsp-1-1	γ-proteobacteria *Reinekea sp.* MED297
Ppr-2-1	γ-proteobacteria Vibrionales Vibrionaceae *Photobacterium profundum* 3TCK
Ppr-3-1	γ-proteobacteria Vibrionales Vibrionaceae *Photobacterium profundum* SS9
Psp-1-1	γ-proteobacteria Vibrionales Vibrionaceae *Photobacterium sp.* SKA34
Val-1-1	γ-proteobacteria Vibrionales Vibrionaceae *Vibrio alginolyticus* 12G01
Van-1-1	γ-proteobacteria Vibrionales Vibrionaceae *Vibrio angustum* S14
Vch-1-1	γ-proteobacteria Vibrionales Vibrionaceae *Vibrio cholerae* 1587
Vch-2-1	γ-proteobacteria Vibrionales Vibrionaceae *Vibrio cholerae* 623-39
Vch-3-1	γ-proteobacteria Vibrionales Vibrionaceae *Vibrio cholerae* AM-19226
Vch-4-1	γ-proteobacteria Vibrionales Vibrionaceae *Vibrio cholerae* B33
Vch-5-1	γ-proteobacteria Vibrionales Vibrionaceae *Vibrio cholerae* MAK 757
Vch-6-1	γ-proteobacteria Vibrionales Vibrionaceae *Vibrio cholerae* MO10

Vch-7-1	γ-proteobacteria Vibrionales Vibrionaceae *Vibrio cholerae* MZO-2
Vch-8-1	γ-proteobacteria Vibrionales Vibrionaceae *Vibrio cholerae* MZO-3
Vch-9-1	γ-proteobacteria Vibrionales Vibrionaceae *Vibrio cholerae* NCTC 8457
Vch-10-1	γ-proteobacteria Vibrionales Vibrionaceae *Vibrio cholerae* O1 biovar eltor str. N16961
Vch-11-1	γ-proteobacteria Vibrionales Vibrionaceae *Vibrio cholerae* O395
Vch-12-1	γ-proteobacteria Vibrionales Vibrionaceae *Vibrio cholerae* RC385
Vch-13-1	γ-proteobacteria Vibrionales Vibrionaceae *Vibrio cholerae* V51
Vch-14-1	γ-proteobacteria Vibrionales Vibrionaceae *Vibrio cholerae* V52
Vfi-1-1	γ-proteobacteria Vibrionales Vibrionaceae *Vibrio fischeri* ES114
Vpa-1-1	γ-proteobacteria Vibrionales Vibrionaceae *Vibrio parahaemolyticus* RIMD 2210633
Vsp-1-1	γ-proteobacteria Vibrionales Vibrionaceae *Vibrio sp.* Ex25
Vsp-2-1	γ-proteobacteria Vibrionales Vibrionaceae *Vibrio sp.* MED222
Vsp-3-1	γ-proteobacteria Vibrionales Vibrionaceae *Vibrio splendidus* 12B01
Vvu-1-1	γ-proteobacteria Vibrionales Vibrionaceae *Vibrio vulnificus* CMCP6
Vvu-2-1	γ-proteobacteria Vibrionales Vibrionaceae *Vibrio vulnificus* YJ016
Sen-4-1	γ-proteobacteria Enterobacteriales Enterobacteriaceae *Salmonella enterica subsp. enterica serovar* Choleraesuis str
env-1 to env-10	environmental samples

Supplementary Figure S2: gene context of Moco RNAs

All hits (indicated by "RNA→") are listed with their downstream genes, according to the RefSeq annotation. Environmental sequences and some RefSeq entries lack gene annotations, and no genes are listed for such sequences. Lines beginning with a superscript "1" have some atypical sequence features, particularly in P2. Those marked with superscript "2" have a weak P2. Tandem arrangements of Moco RNAs are shown with both "RNA→" indicators in the same line. The direction of each downstream gene is indicated with an arrow (→), and each conserved domain in the gene is colored. Conserved domains associated with more than one Moco RNAs are assigned a color; other domains are gray. Conserved domains are explained in Supplementary Figure S3. Nucleotide coordinates are given for the 5′ and 3′ boundaries of the Moco RNAs. Note that these coordinates are for the full sequence listed in Supplementary Figure S4, including extra downstream nucleotides used to annotate transcription terminators and start codons. Therefore the listed 3′ coordinate will extend past the actual Moco RNA.

abbrev	RefSeq accession		5′ at	3′ at	genes
[1]Mth-1-1	NC_007644.1	+	750991	751396	**RNA**→ HybA (COG0437)→ COG2414 (COG2414)→ MoaD (cd00754)→ NirB (COG1251)→
Pca-1-1	NC_007498.2	+	3245437	3245851	**RNA**→ ModA (COG0725)→ CysU (COG0555)→ ABC_ModC_like (cd03299)→
Tth-1-1	NC_005835.1	-	352065	351644	**RNA**→ ModA (COG0725)→ ModC (COG4149)→ PotA (COG3842)→
Tth-2-1	NC_006461.1	-	681426	681005	**RNA**→ ModA (COG0725)→ ModC (COG4149)→ PotA (COG3842)→
Sth-1-1	NC_006177.1	-	3132643	3132211	**RNA**→ MoeA (COG0303)→ MoeA (COG0303)COG1910 (COG1910)→ MoaA (cd01420)→ MoaC_PE (cd01420)→
Ama-1-1	NZ_AAOD01000002.1	+	166500	166949	**RNA**→ MoaA (cd01420)→ MobA (COG0746)→ MoCF_biosynth (pfam00994)→ MoaC_PE (cd01420)→ MoaD (cd00754)→ MoaE (pfam02391)→
Dha-2-1	NC_007907.1	+	1348493	1348946	**RNA**→ Crp (COG0664)→
Dha-1-1	NZ_AAAW04000002.1	-	364364	363912	**RNA**→ Crp (COG0664)→ FepD (COG0609)→ FepC (COG1120)→ COG2014 (COG2014)→ TroA_e (cd01142)→
Dha-1-2	NZ_AAAW04000002.1	-	364364	363940	**RNA**→ Crp (COG0664)→ FepD (COG0609)→ FepC (COG1120)→ COG2014 (COG2014)→ TroA_e (cd01142)→
Aba-1-1	NC_008009.1	+	5126307	5126741	**RNA**→ MoaA (cd01420)→
Dha-2-2	NC_007907.1	-	4378234	4377805	**RNA**→ MoeA (COG0303)→ NifH (COG1348)→ Oxidoreductase_nitrogenase (cd00316)→ Oxidoreductase_nitrogenase (cd00316)→ MoeA (COG0303)→ FecCD (pfam01032)→ FepC (COG1120)→ TroA_e (cd01142)→ BioB (COG0502)→
Dha-1-3	NZ_AAAW04000002.1	-	1125327	1124898	**RNA**→ MoeA (COG0303)→ NifH (cd02040)→ Oxidoreductase_nitrogenase (cd00316)→ Oxidoreductase_nitrogenase (cd00316)→ MoeA (COG0303)→ FecCD (pfam01032)→ FepC (COG1120)→ TroA_e (cd01142)→
Dre-1-1	NZ_AAOP01000023.1	-	43467	43062	**RNA**→ COG2414 (COG2414)→
Dha-2-3	NC_007907.1	-	1874938	1874489	**RNA**→ MoCF_biosynth (pfam00994)→
Dha-1-4	NZ_AAAW04000001.1	-	91667	91218	**RNA**→ MoeA (COG0303)→
Pin-1-1	NC_008709.1	-	2652936	2652483	**RNA**→ MoaA (cd01420)→
Rsp-1-1	NZ_AAOE01000024.1	+	17701	18130	**RNA**→ MoaA (cd01420)→ MobA (COG0746)→ MoCF_biosynth (pfam00994)→ MoaC_PE (cd01420)→ MoaD (cd00754)→ MoaE (pfam02391)→ ModA (COG0725)→ ModC (COG4149)→ ModC (COG4148)→ COG5373 (COG5373)→ GdhA (COG0334)→
Cps-1-1	NC_003910.7	+	4895122	4895611	**RNA**→ MoaA (cd01420)→ MobA (COG0746)→
Hdu-1-1	NC_002940.2	+	1141155	1141624	**RNA**→ MopB_DMSOR-BSOR-TMAOR (cd02769)MopB_CT_DMSOR-BSOR-TMAOR (cd02793)→ hypo→ COG2933 (COG2933)→
Apl-1-1	NZ_AACK01000004.1	-	65976	65510	**RNA**→ MopB_DMSOR-BSOR-TMAOR (cd02769)MopB_CT_DMSOR-BSOR-TMAOR (cd02793)→
[2]Hdu-1-2	NC_002940.2	-	1139711	1139297	**RNA**→ MoaA (cd01420)→ MoaC_PE (cd01420)→ MoaD (cd00754)→ MoaE (pfam02391)→ COG0319 (COG0319)→ COG1444 (COG1444)→
Mha-1-1	NZ_AASA01000098.1	+	11609	12062	**RNA**→
Apl-1-2	NZ_AACK01000004.1	+	67276	67760	**RNA**→ MoaA (cd01420)→
Msu-1-1	NC_006300.1	+	1003377	1003823	**RNA**→ MoaA (cd01420)→ MoaC_PE (cd01420)→ MoaD (cd00754)→ MoaE (pfam02391)→
Hso-1-1	NC_008309.1	-	1138694	1138263	**RNA**→ MoaA (cd01420)→ MoaC_PE (cd01420)→ MoaD (cd00754)→ MoaE (pfam02391)→
Hso-2-1	NZ_AACJ01000021.1	+	28999	29430	**RNA**→ MoaA (cd01420)→
Hin-3-1	NZ_AADP01000001.1	-	1117893	1117455	**RNA**→ MoaA (cd01420)→ MoaC_PE (cd01420)→ MoaD (cd00754)→ MoaE (pfam02391)→
Hin-4-1	NC_000907.1	-	1745366	1744929	**RNA**→ MoaA (cd01420)→ MoaC_PE (cd01420)→ MoaD (cd00754)→ MoaE (pfam02391)→
Hin-2-1	NZ_AADO01000007.1	-	21362	20925	**RNA**→ MoaA (cd01420)→ MoaC_PE (cd01420)→ MoaD (cd00754)→ MoaE (pfam02391)→
Hin-1-1	NC_007146.1	-	1825938	1825500	**RNA**→ MoaA (cd01420)→ MoaC_PE (cd01420)→ MoaD (cd00754)→ MoaE (pfam02391)→

Pmu-1-1	NC_002663.1	-	728600	728154	RNA→ MoaA (cd01420)→ MoaC_PE (cd01420)→ MoaD (cd00754)→ MoaE (pfam02391)→
Asu-1-1	NZ_AAKC01000041.1	+	2109	2559	RNA→ MoaA (cd01420)→ MoaC_PE (cd01420)→ MoaD (cd00754)→ MoaE (pfam02391)→
Hso-2-2	NZ_AACJ01000015.1	-	7972	7538	RNA→ MopB_DMSOR-BSOR-TMAOR (cd02769)MopB_CT_DMSOR-BSOR-TMAOR (cd02793)→
Hso-1-2	NC_008309.1	-	881170	880736	RNA→ MopB_DMSOR-BSOR-TMAOR (cd02769)MopB_CT_DMSOR-BSOR-TMAOR (cd02793)→
Mha-1-2	NZ_AASA01000098.1	-	8433	7940	RNA→
Dha-2-4	NC_007907.1	-	4532670	4532248	RNA→ MoaA (cd01420)→ MoaC_PE (cd01420)→ MOSC (pfam03473)→ MoCF_biosynth (pfam00994)→
Dha-1-5	NZ_AAAW04000002.1	-	1257100	1256678	RNA→ MoaA (cd01420)→ MoaC_PE (cd01420)→ MOSC (pfam03473)→ MoCF_biosynth (pfam00994)→
Csp-1-1	NZ_AAQV01000001.1	-	76873	76453	RNA→ TupB (COG2998)→
Dps-1-1	NC_006138.1	-	3382961	3382539	RNA→ MoaA (cd01420)→ MoeA (COG0303)→
Mth-1-2	NC_007644.1	-	2236534	2236096	RNA→ MoeA (COG0303)→
Ame-1-1	NZ_AAKU01000064.1	-	2597	2184	RNA→ MoeA (COG0303)→ MoeA (COG0303)→
Ame-1-2	NZ_AAKU01000050.1	+	11778	12195	RNA→ MoaA (cd01420)→ MOSC (pfam03473)→ UgpB (COG1653)→
Ahy-1-1	NC_008570.1	+	1732669	1733105	RNA→ MoaA (cd01420)→
env-1	AACY01062372.1	+	124	560	RNA→ unknown→
Plu-1-1	NC_005126.1	+	1797531	1797972	RNA→ MoaA (cd01420)→ MoaC_PE (cd01420)→ MoaD (cd00754)→ MoaE (pfam02391)→
Sgl-1-1	NC_007712.1	+	1475425	1475862	RNA→ MoaC_PE (cd01420)→
Ymo-1-1	NZ_AALD01000048.1	-	3422	2961	RNA→ MoaA (cd01420)→ MoaC_PE (cd01420)→ MoaD (cd00754)→ MoaE (pfam02391)→
Yps-2-1	NC_006155.1	+	1419284	1419727	RNA→ MoaA (cd01420)→
Ype-2-1	NC_008150.1	+	1219676	1220119	RNA→ MoaA (cd01420)→
Ype-6-1	NC_008149.1	-	3199155	3198712	RNA→ MoaA (cd01420)→
Ype-5-1	NC_004088.1	-	3330087	3329644	RNA→ MoaA (cd01420)→ MoaC_PE (cd01420)→ MoaD (cd00754)→ MoaE (pfam02391)→
Ype-3-1	NC_003143.1	+	1306720	1307163	RNA→ MoaA (cd01420)→
Ype-7-1	NC_005810.1	-	1078165	1077722	RNA→ MoaA (cd01420)→
Ype-1-1	NZ_AAKS01000002.1	-	4040	3597	RNA→ MoaA (cd01420)→
Ype-8-1	NZ_AAOS01000107.1	-	6040	5597	RNA→ MoaA (cd01420)→
Ype-4-1	NZ_AAUB01000157.1	+	9443	9886	RNA→
Yps-1-1	NZ_AAKT02000001.1	-	3203744	3203301	RNA→ MoaA (cd01420)→
Yfr-1-1	NZ_AALE01000012.1	-	112610	112168	RNA→ MoaA (cd01420)→
Yin-1-1	NZ_AALF01000058.1	+	11923	12364	RNA→ MoaA (cd01420)→ MoaC_PE (cd01420)→ MoaD (cd00754)→ MoaE (pfam02391)→
Ybe-1-1	NZ_AALC01000042.1	-	3242	2781	RNA→ MoaA (cd01420)→ MoaC_PE (cd01420)→ MoaD (cd00754)→ MoaE (pfam02391)→
Spr-1-1	NZ_AAUN01000005.1	+	39566	40005	RNA→ MoaA (cd01420)→ MoCF_biosynth (pfam00994)→ MoaC_PE (cd01420)→ MoaD (cd00754)→ MoaE (pfam02391)→
Eca-1-1	NC_004547.2	-	3157560	3157115	RNA→ MoaA (cd01420)→ MoCF_biosynth (pfam00994)→ MoaC_PE (cd01420)→ MoaD (cd00754)→ MoaE (pfam02391)→
Esp-1-1	NZ_AAVF01000005.1	+	3453	3884	RNA→ MoaA (cd01420)→ MoCF_biosynth (pfam00994)→ MoaC_PE (cd01420)→ MoaD (cd00754)→ MoaE (pfam02391)→ COG0670 (COG0670)→
env-2	AACY01072431.1	-	3004	2562	RNA→ unknown→ unknown→
Eco-17-1	AC_000091.1	+	817334	817775	RNA→ MoaA (cd01420)→ MoCF_biosynth (pfam00994)→ MoaC_PE (cd01420)→ MoaD (cd00754)→ MoaE (pfam02391)→
Eco-13-1	NC_000913.2	+	816135	816576	RNA→ MoaA (cd01420)→ MoCF_biosynth (pfam00994)→ MoaC_PE (cd01420)→ MoaD (cd00754)→ MoaE (pfam02391)→
Esi-1-1	NZ_AADW02000034.1	-	14522	14081	RNA→ MoaA (cd01420)→ MoCF_biosynth (pfam00994)→ MoaC_PE (cd01420)→ MoaD (cd00754)→ MoaE (pfam02391)→
Bce-1-1	NZ_AALL01000063.1	-	15154	14713	RNA→ MoaA (cd01420)→ MoCF_biosynth (pfam00994)→ MoaC_PE (cd01420)→ MoaD (cd00754)→ MoaE (pfam02391)→
Eco-6-1	NZ_AAJT01000095.1	+	3720	4161	RNA→ MoaA (cd01420)→ MoCF_biosynth (pfam00994)→ MoaC_PE (cd01420)→ MoaD (cd00754)→ MoaE (pfam02391)→
Eco-9-1	NZ_AAJV01000017.1	-	101323	100882	RNA→ MoaA (cd01420)→ MoCF_biosynth (pfam00994)→ MoaC_PE (cd01420)→ MoaD (cd00754)→ MoaE (pfam02391)→
Eco-8-1	NZ_AAJW01000015.1	-	98222	97781	RNA→ MoaA (cd01420)→ MoCF_biosynth (pfam00994)→ MoaC_PE (cd01420)→ MoaD (cd00754)→ MoaE (pfam02391)→
Eco-5-1	NZ_AAJX01000014.1	-	101443	101002	RNA→ MoaA (cd01420)→ MoCF_biosynth (pfam00994)→ MoaC_PE (cd01420)→ MoaD (cd00754)→ MoaE (pfam02391)→
Eco-10-1	NZ_AAJZ01000001.1	+	3627779	3628220	RNA→ MoaA (cd01420)→ MoCF_biosynth (pfam00994)→ MoaC_PE (cd01420)→ MoaD (cd00754)→ MoaE (pfam02391)→

Eco-3-1	NZ_AAKB01000019.1	+	3744	4185	**RNA**→ MoaA (cd01420)→ MoCF_biosynth (pfam00994)→ MoaC_PE (cd01420)→ MoaD (cd00754)→ MoaE (pfam02391)→
Eco-1-1	NZ_AAMK01000014.1	+	3873	4314	**RNA**→ MoaA (cd01420)→ MoCF_biosynth (pfam00994)→ MoaC_PE (cd01420)→ MoaD (cd00754)→ MoaE (pfam02391)→
Sbo-2-1	NC_007613.1	+	682934	683375	**RNA**→ MoaA (cd01420)→ MoCF_biosynth (pfam00994)→ MoaC_PE (cd01420)→ MoaD (cd00754)→ MoaE (pfam02391)→
Sso-1-1	NC_007384.1	+	805998	806439	**RNA**→ MoaA (cd01420)→ MoCF_biosynth (pfam00994)→ MoaC_PE (cd01420)→ MoaD (cd00754)→ MoaE (pfam02391)→
Eco-12-1	NZ_AAJY01000001.1	+	843515	843956	**RNA**→ MoaA (cd01420)→ MoCF_biosynth (pfam00994)→ MoaC_PE (cd01420)→ MoaD (cd00754)→ MoaE (pfam02391)→
Sfl-3-1	NC_008258.1	+	795717	796159	**RNA**→ MoaA (cd01420)→ MoCF_biosynth (pfam00994)→ MoaC_PE (cd01420)→ MoaD (cd00754)→ MoaE (pfam02391)→
Eco-2-1	NC_008253.1	+	832790	833231	**RNA**→ MoaA (cd01420)→ MoCF_biosynth (pfam00994)→ MoaC_PE (cd01420)→ MoaD (cd00754)→ MoaE (pfam02391)→
Eco-16-1	NC_007946.1	+	779855	780296	**RNA**→ MoaA (cd01420)→ MoCF_biosynth (pfam00994)→
Eco-4-1	NC_008563.1	+	781456	781897	**RNA**→ MoaA (cd01420)→ MoCF_biosynth (pfam00994)→ MoaC_PE (cd01420)→
Eco-7-1	NC_004431.1	+	839533	839974	**RNA**→ MoaA (cd01420)→ MoCF_biosynth (pfam00994)→
Eco-11-1	NZ_AAJU01000056.1	+	3338	3779	**RNA**→ MoaA (cd01420)→ MoCF_biosynth (pfam00994)→ MoaC_PE (cd01420)→ MoaD (cd00754)→ MoaE (pfam02391)→
Sfl-2-1	NC_004337.1	+	759739	760180	**RNA**→ MoaA (cd01420)→ MoCF_biosynth (pfam00994)→ MoaC_PE (cd01420)→ MoaD (cd00754)→ MoaE (pfam02391)→
Sfl-1-1	NC_004741.1	+	755896	756337	**RNA**→ MoaA (cd01420)→ MoCF_biosynth (pfam00994)→ MoaC_PE (cd01420)→ MoaD (cd00754)→ MoaE (pfam02391)→
Sdy-1-1	NC_007606.1	-	768837	768396	**RNA**→ MoaA (cd01420)→ MoCF_biosynth (pfam00994)→ MoaC_PE (cd01420)→ MoaD (cd00754)→ MoaE (pfam02391)→
Sbo-1-1	NZ_AAKA01000006.1	-	222070	221629	**RNA**→ MoaA (cd01420)→ MoCF_biosynth (pfam00994)→ MoaC_PE (cd01420)→ MoaD (cd00754)→ MoaE (pfam02391)→
Eco-15-1	NC_002695.1	+	939277	939718	**RNA**→ MoaA (cd01420)→ MoCF_biosynth (pfam00994)→ MoaC_PE (cd01420)→ MoaD (cd00754)→ MoaE (pfam02391)→
Eco-14-1	NC_002655.2	+	940930	941371	**RNA**→ MoaA (cd01420)→ MoCF_biosynth (pfam00994)→ MoaC_PE (cd01420)→ MoaD (cd00754)→ MoaE (pfam02391)→
Sty-1-1	NC_003197.1	+	870506	870947	**RNA**→ MoaA (cd01420)→ MoCF_biosynth (pfam00994)→ MoaC_PE (cd01420)→ MoaD (cd00754)→ MoaE (pfam02391)→ COG0670 (COG0670)→
Sen-1-1	NC_006511.1	-	2029594	2029153	**RNA**→ MoaA (cd01420)→ MoCF_biosynth (pfam00994)→ MoaC_PE (cd01420)→ MoaD (cd00754)→ MoaE (pfam02391)→ COG0670 (COG0670)→
Sen-4-1	NC_006905.1	+	899104	899545	**RNA**→ MoaA (cd01420)→ MoCF_biosynth (pfam00994)→ MoaC_PE (cd01420)→ MoaD (cd00754)→ MoaE (pfam02391)→ COG0670 (COG0670)→
Sen-2-1	NC_004631.1	-	2146005	2145564	**RNA**→ MoaA (cd01420)→ MoCF_biosynth (pfam00994)→ MoaC_PE (cd01420)→ MoaD (cd00754)→ MoaE (pfam02391)→
Sen-3-1	NC_003198.1	+	835072	835513	**RNA**→ MoaA (cd01420)→ MoCF_biosynth (pfam00994)→ MoaC_PE (cd01420)→ MoaD (cd00754)→ MoaE (pfam02391)→
Sam-1-1	NC_008700.1	+	4159189	4159620	**RNA**→ MoaA (cd01420)→ MoCF_biosynth (pfam00994)→ MoaC_PE (cd01420)→ MoaD (cd00754)→ MoaE (pfam02391)→ ModA (COG0725)→ ModC (COG4149)→ PotA (COG3842)→ DUF388 (pfam04076)→ hypo→ OmpR (COG0745)→ BaeS (COG0642)→ PseudoU_synth_RluCD_like (cd02869)→
Spe-1-1	NZ_AAVJ01000006.1	+	115740	116173	**RNA**→ MoaA (cd01420)→ MoCF_biosynth (pfam00994)→ MoaC_PE (cd01420)→ MoaD (cd00754)→ MoaE (pfam02391)→ ModA (COG0725)→ ModC (COG4149)→ PotA (COG3842)→
Ssp-4-1	NZ_AALS01000010.1	-	48879	48448	**RNA**→ MoaA (cd01420)→
Swo-2-1	NZ_AAUO01000014.1	-	46067	45636	**RNA**→ MoaA (cd01420)→
Ssp-5-1	NC_008750.1	+	268441	268729	**RNA**→ MoaA (cd01420)→ MoaC_PE (cd01420)→ MoaD (cd00754)→ MoaE (pfam02391)→ ModA (COG0725)→ ModC (COG4149)→ PotA (COG3842)→
env-3	AACY01258774.1	+	651	939	**RNA**→
Ssp-5-2	NC_008750.1	+	268441	268871	**RNA**→ MoaA (cd01420)→ MoaC_PE (cd01420)→ MoaD (cd00754)→ MoaE (pfam02391)→ ModA (COG0725)→ ModC (COG4149)→ PotA (COG3842)→
Spu-1-1	NZ_AALB01000013.1	-	104357	103927	**RNA**→ MoaA (cd01420)→ MoaC_PE (cd01420)→ MoaD (cd00754)→ MoaE (pfam02391)→ ModA (COG0725)→ ModC (COG4149)→ PotA (COG3842)→
Sba-1-1	NZ_AAIO01000006.1	-	98330	97900	**RNA**→ MoaA (cd01420)→ MoaC_PE (cd01420)→
Sba-2-1	NZ_AATK01000015.1	-	41805	41375	**RNA**→ MoaA (cd01420)→ MoaC_PE (cd01420)→

Ssp-1-1	NC_008577.1	+	309677	310107	**RNA**→ MoaA (cd01420)→
env-4	AACY01016904.1	-	44433	44003	**RNA**→ unknown→ unknown→ unknown→ unknown→ unknown→
Ssp-2-1	NC_008321.1	+	294832	295262	**RNA**→ MoaA (cd01420)→
Ssp-3-1	NC_008322.1	-	4455501	4455071	**RNA**→ MoaA (cd01420)→
Son-1-1	NC_004347.1	-	4641235	4640805	**RNA**→ MoaA (cd01420)→
env-5	AACY01051512.1	-	31696	31266	**RNA**→ unknown→ unknown→ unknown→ unknown→ unknown→
Sfr-1-1	NC_008345.1	+	97810	98239	**RNA**→ MoaA (cd01420)→ MoaC_PE (cd01420)→ MoaD (cd00754)→ MoaE (pfam02391)→ ModA (COG0725)→ ModC (COG4149)→ PotA (COG3842)→
Sde-1-1	NC_007954.1	+	114598	115028	**RNA**→ MoaA (cd01420)→
Vch-3-1	NZ_AATY01000030.1	-	31957	31521	**RNA**→
Vch-1-1	NZ_AAUR01000042.1	-	26458	26022	**RNA**→
Vch-8-1	NZ_AAUU01000009.1	-	30696	30260	**RNA**→
Vch-10-1	NC_002505.1	+	1093019	1093455	**RNA**→ MoaA (cd01420)→ MoCF_biosynth (pfam00994)→ MoaC_PE (cd01420)→ MoaD (cd00754)→ MoaE (pfam02391)→
Vch-6-1	NZ_AAKF02000033.1	+	13639	14075	**RNA**→ MoaA (cd01420)→ MoCF_biosynth (pfam00994)→ MoaC_PE (cd01420)→ MoaD (cd00754)→ MoaE (pfam02391)→
Vch-11-1	NZ_AAKG01000001.1	+	583754	584190	**RNA**→ MoaA (cd01420)→ MoCF_biosynth (pfam00994)→ MoaC_PE (cd01420)→ MoaD (cd00754)→ MoaE (pfam02391)→
Vch-14-1	NZ_AAKJ01000024.1	-	31281	30845	**RNA**→ MoaA (cd01420)→ MoCF_biosynth (pfam00994)→ MoaC_PE (cd01420)→ MoaD (cd00754)→ MoaE (pfam02391)→
Vch-5-1	NZ_AAUS01000053.1	-	12317	11881	**RNA**→
Vch-9-1	NZ_AAWD01000062.1	-	12304	11868	**RNA**→
Vch-4-1	NZ_AAWE01000035.1	-	31834	31398	**RNA**→
Vch-13-1	NZ_AAKI02000041.1	-	24441	24005	**RNA**→ MoaA (cd01420)→ MoCF_biosynth (pfam00994)→ MoaC_PE (cd01420)→ MoaD (cd00754)→ MoaE (pfam02391)→
Vch-7-1	NZ_AAWF01000006.1	-	90475	90039	**RNA**→
Vch-2-1	NZ_AAWG01000034.1	-	4112	3676	**RNA**→
Vch-12-1	NZ_AAKH02000052.1	+	6641	7077	**RNA**→ MoaA (cd01420)→ MoCF_biosynth (pfam00994)→ MoaC_PE (cd01420)→ MoaD (cd00754)→ MoaE (pfam02391)→
Vvu-1-1	NC_004459.2	-	3156155	3155708	**RNA**→ MoaA (cd01420)→ MoCF_biosynth (pfam00994)→ MoaC_PE (cd01420)→ MoaD (cd00754)→ MoaE (pfam02391)→
Vvu-2-1	NC_005139.1	+	1214618	1215065	**RNA**→ MoaA (cd01420)→ MoCF_biosynth (pfam00994)→ MoaC_PE (cd01420)→ MoaD (cd00754)→ MoaE (pfam02391)→
Vsp-3-1	NZ_AAMR01000039.1	-	24877	24428	**RNA**→ MoaA (cd01420)→
Vsp-2-1	NZ_AAND01000029.1	-	42323	41874	**RNA**→ MoaA (cd01420)→ MoCF_biosynth (pfam00994)→ MoaC_PE (cd01420)→ MoaD (cd00754)→ MoaE (pfam02391)→
Vpa-1-1	NC_004603.1	-	2204413	2203983	**RNA**→ MoaA (cd01420)→
Vsp-1-1	NZ_AAKK02000029.1	-	45601	45170	**RNA**→ MoaA (cd01420)→
Val-1-1	NZ_AAPS01000028.1	+	5735	6166	**RNA**→ MoaA (cd01420)→
Vfi-1-1	NC_006840.1	+	1036831	1037276	**RNA**→ MoaA (cd01420)→ MoaC_PE (cd01420)→ MoaD (cd00754)→ MoaE (pfam02391)→
Psp-1-1	NZ_AAOU01000009.1	+	42833	43301	**RNA**→ MoaA (cd01420)→ MoaC_PE (cd01420)→ MoaD (cd00754)→ MoaE (pfam02391)→
Van-1-1	NZ_AAOJ01000002.1	-	672485	672019	**RNA**→ MoaA (cd01420)→ MoaC_PE (cd01420)→ MoaD (cd00754)→ MoaE (pfam02391)→
Ppr-3-1	NC_006370.1	+	1247584	1248033	**RNA**→ MoaA (cd01420)→
Ppr-2-1	NZ_AAPH01000008.1	+	168857	169305	**RNA**→ MoaA (cd01420)→
Dre-1-2	NZ_AAOP01000063.1	+	12330	12744	**RNA**→ hypo→ hypo→
Swo-1-1	NC_008346.1	-	1858702	1858284	**RNA**→ TupA (COG4662)→ ABC_PstB_phosphate_transporter (cd03260)→ TupB (COG2998)SH3b (smart00287)→
Pat-1-1	NC_008228.1	-	2608745	2608298	**RNA**→ MoaA (cd01420)→ MobA (COG0746)→
Pat-1-2	NC_008228.1	-	2608745	2608266	**RNA**→ MoaA (cd01420)→ MobA (COG0746)→
Swo-1-2	NC_008346.1	-	2093060	2092643	**RNA**→ COG3383 (COG3383)→ MopB_Formate-Dh-H (cd02753)MopB_CT_Formate-Dh_H (cd02790)→
env-6	AACY01737198.1	-	169	1	**RNA**→
env-7	AACY01304733.1	+	395	811	**RNA**→ unknown→
Swo-1-3	NC_008346.1	-	2126197	2125783	**RNA**→ MoaA (cd01420)→ MoaC_PE (cd01420)→ MOSC (pfam03473)→ MoCF_biosynth (pfam00994)→
Swo-1-4	NC_008346.1	-	2126197	2125782	**RNA**→ MoaA (cd01420)→ MoaC_PE (cd01420)→ MOSC (pfam03473)→ MoCF_biosynth (pfam00994)→
Chy-1-1	NC_007503.1	+	711593	712009	**RNA**→ MoaA (cd01420)→ MoaC_PE (cd01420)→ MOSC (pfam03473)→ MoCF_biosynth (pfam00994)→

Swo-1-5	NC_008346.1	-	1907441	1907004	**RNA**→ TroA_e (cd01142)→
Mth-1-3	NC_007644.1	-	1884333	1883901	**RNA**→ ModA (COG0725)→ CysU (COG0555)→ PotA (COG3842)→
Swo-1-6	NC_008346.1	-	2528175	2527762	**RNA**→ SO_family_Moco (cd00321)arch_bact_SO_family_Moco (cd02109)→
Csa-1-1	NZ_AALW01000009.1	+	12803	13234	**RNA**→ TupB (COG2998)→ TupA (COG4662)→ ABC_PstB_phosphate_transporter (cd03260)→<br>MoeA (COG0303)→ MoeA (COG0303)→ MoaA (cd01420)→ MoaC_PE (cd01420)MOSC (pfam03473)→
Dac-1-1	NZ_AAEW02000019.1	-	27542	27126	**RNA**→ COG2414 (COG2414)→
Dac-1-2	NZ_AAEW02000009.1	+	10109	10524	**RNA**→ TupB (COG2998)→ TupA (COG4662)→ ABC_cobalt_transport_domain1 (cd03225)→<br>MoeA (COG0303)→ EutG (COG1454)→ FhlA (COG2203)COG4191 (COG4191)→ AtoC (COG2204)→<br>Radical_SAM (pfam04055)→ COG1051 (COG1051)→
Hne-1-1	NC_008358.1	+	1582667	1583091	**RNA**→ MoaA (cd01420)→
Swo-1-7	NC_008346.1	-	1376295	1375882	**RNA**→ hypo→
Dac-1-3	NZ_AAEW02000009.1	-	8135	7717	**RNA**→ COG2414 (COG2414)→
Gsp-1-1	NZ_AASH01000001.1	-	18358	17939	**RNA**→ COG2414 (COG2414)→ MoaD (cd00754)→ ThiF_MoeB_HesA_family (cd00757)→ TatA (COG1826)→<br>HTH_MARR (smart00347)→
Gsp-1-3	NZ_AASH01000047.1	+	24335	24763	**RNA**→ TupA (COG4662)→ ABC_cobalt_transport_domain1 (cd03225)→ MoeA (COG0303)→<br>MoaC_PE (cd01420)→
Pca-1-2	NC_007498.2	+	804624	805042	**RNA**→ COG2414 (COG2414)→
Gsp-1-4	NZ_AASH01000041.1	+	13425	13840	**RNA**→ **RNA**→ Porin_O_P (pfam07396)→
Gsp-1-2	NZ_AASH01000041.1	+	13636	14050	(shown above)
Ppr-1-1	NC_008609.1	-	621095	620681	**RNA**→ COG2414 (COG2414)→
Gur-1-1	NZ_AAON01000002.1	+	97236	97650	**RNA**→ COG2414 (COG2414)→
Gur-1-2	NZ_AAON01000002.1	+	111352	111769	**RNA**→ hypo→
Gsu-1-1	NC_002939.4	+	2979275	2979690	**RNA**→ MoaE (pfam02391)→ TupB (COG2998)→ TupA (COG4662)→ ABC_Solutes_Sugar (cd03259)→<br>MoeA (COG0303)→ MoaC_PE (cd01420)→ MoCF_biosynth (pfam00994)→
Gme-1-1	NC_007517.1	-	1157929	1157512	**RNA**→ Porin_O_P (pfam07396)→
Gur-1-3	NZ_AAON01000002.1	+	101466	101882	**RNA**→ MoaE (pfam02391)→
Pca-1-3	NC_007498.2	-	556955	556539	**RNA**→ COG2414 (COG2414)→
Gme-1-2	NC_007517.1	-	1161059	1160645	**RNA**→ COG2414 (COG2414)→ MoaD (cd00754)→ ThiF_MoeB_HesA_family (cd00757)→
Pca-1-4	NC_007498.2	+	319211	319634	**RNA**→ MoeA (COG0303)→
Ppr-1-2	NC_008609.1	+	935247	935661	**RNA**→ COG2414 (COG2414)→
Pca-1-5	NC_007498.2	-	2958797	2958381	**RNA**→ COG2414 (COG2414)→
Gur-1-4	NZ_AAON01000003.1	+	149537	149952	**RNA**→ COG2414 (COG2414)→ MoaD (cd00754)→ ThiF_MoeB_HesA_family (cd00757)→
Gsp-1-5	NZ_AASH01000041.1	+	9841	10254	**RNA**→ COG2414 (COG2414)→ MoaD (cd00754)→ ThiF_MoeB_HesA_family (cd00757)→ MopI (COG3585)→
Gme-1-4	NC_007517.1	+	2016356	2016772	**RNA**→ **RNA**→ COG2414 (COG2414)→ HycB (COG1142)→
Gme-1-3	NC_007517.1	+	2016583	2016999	(shown above)
Csp-2-1	NZ_AATH01000004.1	-	223802	223368	**RNA**→ MoaA (cd01420)→
env-8	AATD01006421.1	+	74	485	**RNA**→
Tet-1-1	NZ_AAKQ01000001.1	+	275618	276038	**RNA**→ TupB (COG2998)→ TupA (COG4662)→ ABC_Solutes_Sugar (cd03259)→
Tet-2-1	NZ_AATV01000009.1	+	44162	44582	**RNA**→ TupB (COG2998)→ TupA (COG4662)→ ABC_Solutes_Sugar (cd03259)→
env-9	AACY01543970.1	+	548	892	**RNA**→
env-10	AACY01600064.1	+	618	926	**RNA**→
Swo-1-8	NC_008346.1	+	2196867	2197285	**RNA**→ **RNA**→ NapF (COG1145)Fe_hyd_lg_C (pfam02906)→ Fe_hyd_SSU (pfam02256)→ FdnI (COG2864)→
Swo-1-10	NC_008346.1	+	2197046	2197465	(shown above)
Swo-1-9	NC_008346.1	+	1210598	1211013	**RNA**→ ←H_PPase (pfam03030)
Swo-1-11	NC_008346.1	-	2086437	2086020	**RNA**→ Molybdop_Fe4S4 (pfam04879)→<br>MopB_Formate-Dh-Na-like (cd02752)MopB_CT_Formate-Dh-Na-like (cd02792)→ HybA (COG0437)→<br>FdnI (COG2864)→ putative formate dehydrogenase formation protein FdhE→
Dha-1-6	NZ_AAAW04000002.1	-	955371	954945	**RNA**→ hypo→
Dha-2-5	NC_007907.1	+	4256230	4256659	**RNA**→ hypo→
Dha-1-7	NZ_AAAW04000002.1	+	1036782	1037211	**RNA**→ hypo→
Mth-1-4	NC_007644.1	-	1470761	1470327	**RNA**→ COG2014 (COG2014)→ FecCD (pfam01032)→ FepC (COG1120)→
Dha-1-8	NZ_AAAW04000001.1	-	1635277	1634869	**RNA**→ CCG (pfam02754)→ hypo (cons)→
Dha-2-7	NC_007907.1	-	3493239	3492810	**RNA**→ **RNA**→ CCG (pfam02754)→ hypo→ hypo→ GlyRS_alpha_core (cd00733)→ GlyS (COG0751)→
Dha-2-6	NC_007907.1	-	3493229	3492821	(shown above)

Mth-1-5	NC_007644.1	-	1482216	1481784	**RNA**→ COG2014 (COG2014)→
Cpe-2-1	NC_008262.1	-	1962720	1962264	**RNA**→ MoeA (COG0303)→ MobA (COG0746)→
Cpe-1-1	NC_008261.1	-	2288585	2288094	**RNA**→ MoeA (COG0303)→ MobA (COG0746)→
Cpe-3-1	NC_003366.1	-	2073405	2072914	**RNA**→ MoeA (COG0303)→ MobA (COG0746)→
Dre-1-3	NZ_AAOP01000063.1	-	1703	1288	**RNA**→ TupB (COG2998)→
Swo-1-12	NC_008346.1	-	2249503	2249088	**RNA**→ SO_family_Moco (cd00321)→
Tet-1-2	NZ_AAKQ01000001.1	+	273969	274383	**RNA**→ MobB (pfam03205)FeS (pfam04060)→
Tet-2-2	NZ_AATV01000009.1	+	42513	42927	**RNA**→ MobB (pfam03205)FeS (pfam04060)→
Swo-1-14	NC_008346.1	-	1172054	1171634	**RNA**→ **RNA**→ **RNA**→ COG3383 (COG3383)→ MopB_Formate-Dh-H (cd02753)MopB_CT_Formate-Dh_H (cd02790)→ MoCF_biosynth (pfam00994)→ MoeA (COG0303)→ NuoE (COG1905)→ NuoF (COG1894)→
Swo-1-13	NC_008346.1	-	1171874	1171457	(shown above)
Swo-1-15	NC_008346.1	-	1171689	1171143	(shown above)
Sfu-1-1	NC_008554.1	+	38745	39152	**RNA**→ TupB (COG2998)→ TupA (COG4662)→ ABC_Solutes_Sugar (cd03259)→

Supplementary Figure S3: conserved domains present in genes downstream of Moco RNAs

Conserved domains found in downstream genes (Supplementary Figure S2) are listed, with the first sentence in their description from the Conserved Domain Database. Conserved domains downstream of more than one Moco RNA are assigned a color, while others are shown in gray. Note: because there are many domains associated with Moco RNAs, some colors are re-used.

cd00316 The nitrogenase enzyme system catalyzes the ATP-dependent reduction of dinitrogen to ammonia. [4Fe-4S]
cd00321 Sulfite oxidase (SO) family, molybdopterin binding domain.
cd00733 Class II Glycyl-tRNA synthetase (GlyRS) alpha subunit core catalytic domain.
cd00754 Molybdopterin converting factor, small subunit [Coenzyme metabolism]
cd00757 ThiF_MoeB_HesA.
cd01142 Periplasmic binding protein TroA_e.
cd01420 Molybdenum cofactor biosynthesis enzyme [Coenzyme metabolism]
cd02040 NifH gene encodes component II (iron protein) of nitrogenase.
cd02109 bacterial and archael members of the sulfite oxidase (SO) family of molybdopterin binding domains.
cd02752 Formate dehydrogenase N, alpha subunit (Formate-Dh-Na) is a major component of nitrate respiration in bacteria such as in the E. [4Fe-4S] center, and a small subunit that harbors a series of three [4Fe-4S]
cd02753 Formate dehydrogenase H (Formate-Dh-H) catalyzes the reversible oxidation of formate to CO2 with the release of a proton and two electrons. [4Fe-4S]
cd02769 The MopB_DMSOR-BSOR-TMAOR CD contains dimethylsulfoxide reductase (DMSOR), biotin sulfoxide reductase (BSOR), trimethylamine N-oxide reductase (TMAOR) and other related proteins.
cd02790 Formate dehydrogenase H (Formate-Dh-H) catalyzes the reversible oxidation of formate to CO2 with the release of a proton and two electrons. [4Fe-4S]
cd02792 Formate dehydrogenase N, alpha subunit (Formate-Dh-Na) is a major component of nitrate respiration in bacteria such as in the E. [4Fe-4S] center, and a small subunit that harbors a series of three [4Fe-4S]
cd02793 The MopB_DMSOR-BSOR-TMAOR CD contains dimethylsulfoxide reductase (DMSOR), biotin sulfoxide reductase (BSOR), trimethylamine N-oxide reductase (TMAOR) and other related proteins.
cd02869 PseudoU_synth_RsuA/RluD: Pseudouridine synthase, RsuA/RluD family.
cd03225 Domain I of the ATPase component of a cobalt transport family found in both bacteria and archaea.
cd03259 This family comprised of systems involved in the transport of apparently unrelated solutes and systems specific for di- and oligosaccharides and polyols.
cd03260 Phosphate uptake is of fundamental importance in the cell physiology of bacteria because phosphate is required as a nutrient.
cd03299 Archeal protein closely related to ModC.
COG0303 Molybdopterin biosynthesis enzyme [Coenzyme metabolism]
COG0319 Predicted metal-dependent hydrolase [General function prediction only]
COG0334 Glutamate dehydrogenase/leucine dehydrogenase [Amino acid transport and metabolism]
COG0437 Fe-S-cluster-containing hydrogenase components 1 [Energy production and conversion]
COG0502 Biotin synthase and related enzymes [Coenzyme metabolism]
COG0555 ABC-type sulfate transport system, permease component [Posttranslational modification, protein turnover, chaperones]
COG0609 ABC-type Fe3+-siderophore transport system, permease component [Inorganic ion transport and metabolism]
COG0642 Signal transduction histidine kinase [Signal transduction mechanisms]
COG0664 cAMP-binding proteins - catabolite gene activator and regulatory subunit of cAMP-dependent protein kinases [Signal transduction mechanisms]
COG0670 Integral membrane protein, interacts with FtsH [General function prediction only]
COG0725 ABC-type molybdate transport system, periplasmic component [Inorganic ion transport and metabolism]
COG0745 Response regulators consisting of a CheY-like receiver domain and a winged-helix DNA-binding domain [Signal transduction mechanisms / Transcription]
COG0746 Molybdopterin-guanine dinucleotide biosynthesis protein A [Coenzyme metabolism]
COG0751 Glycyl-tRNA synthetase, beta subunit [Translation, ribosomal structure and biogenesis]
COG1051 ADP-ribose pyrophosphatase [Nucleotide transport and metabolism]
COG1120 ABC-type cobalamin/Fe3+-siderophores transport systems, ATPase components [Inorganic ion transport and metabolism / Coenzyme metabolism]
COG1142 Fe-S-cluster-containing hydrogenase components 2 [Energy production and conversion]
COG1145 Ferredoxin [Energy production and conversion]
COG1251 NAD(P)H-nitrite reductase [Energy production and conversion]
COG1348 Nitrogenase subunit NifH (ATPase) [Inorganic ion transport and metabolism]
COG1444 Predicted P-loop ATPase fused to an acetyltransferase [General function prediction only]
COG1454 Alcohol dehydrogenase, class IV [Energy production and conversion]
COG1653 ABC-type sugar transport system, periplasmic component [Carbohydrate transport and metabolism]
COG1826 Sec-independent protein secretion pathway components [Intracellular trafficking and secretion]
COG1894 NADH:ubiquinone oxidoreductase, NADH-binding (51 kD) subunit [Energy production and conversion]
COG1905 NADH:ubiquinone oxidoreductase 24 kD subunit [Energy production and conversion]
COG1910 Periplasmic molybdate-binding protein/domain [Inorganic ion transport and metabolism]
COG2014 Uncharacterized conserved protein [Function unknown]
COG2203 FOG: GAF domain [Signal transduction mechanisms]
COG2204 Response regulator containing CheY-like receiver, AAA-type ATPase, and DNA-binding domains [Signal transduction mechanisms]
COG2414 Aldehyde:ferredoxin oxidoreductase [Energy production and conversion]
COG2864 Cytochrome b subunit of formate dehydrogenase [Energy production and conversion]
COG2933 Predicted SAM-dependent methyltransferase [General function prediction only]

COG2998 ABC-type tungstate transport system, permease component [Coenzyme metabolism]
COG3383 Uncharacterized anaerobic dehydrogenase [General function prediction only]
COG3585 Molybdopterin-binding protein [Coenzyme metabolism]
COG3842 ABC-type spermidine/putrescine transport systems, ATPase components [Amino acid transport and metabolism]
COG4148 ABC-type molybdate transport system, ATPase component [Inorganic ion transport and metabolism]
COG4149 ABC-type molybdate transport system, permease component [Inorganic ion transport and metabolism]
COG4191 Signal transduction histidine kinase regulating C4-dicarboxylate transport system [Signal transduction mechanisms]
COG4662 ABC-type tungstate transport system, periplasmic component [Coenzyme metabolism]
COG5373 Predicted membrane protein [Function unknown]
pfam00994 Probable molybdopterin binding domain.
pfam01032 FecCD transport family.
pfam02256 Iron hydrogenase small subunit.
pfam02391 MoaE protein.
pfam02754 Cysteine-rich domain.
pfam02906 Iron only hydrogenase large subunit, C-terminal domain.
pfam03030 Inorganic H+ pyrophosphatase.
pfam03205 Molybdopterin guanine dinucleotide synthesis protein B.
pfam03473 MOSC domain.
pfam04055 Radical SAM superfamily.
pfam04060 Putative Fe-S cluster.
pfam04076 Domain unknown function (DUF388).
pfam04879 Molybdopterin oxidoreductase Fe4S4 domain.
pfam07396 Phosphate-selective porin O and P.
smart00287 Bacterial SH3 domain homologues;
smart00347 helix_turn_helix multiple antibiotic resistance protein;

Supplementary Figure S4: Multiple sequence alignment of Moco RNAs with 3′ flanking sequences

The multiple sequence alignment of Moco RNAs follows. The alignment includes sequences containing the putative Moco RNAs, as well as downstream sequence, in which Shine-Dalgarno sequences, rho-independent transcription terminators and start codons are annotated. Lines beginning with a superscript "1" have some atypical sequence features, particularly in P2. Those marked with superscript "2" have a weak P2. Nucleotides proposed to base-pair are colored when they comprise Watson-Crick or G-U pairs. Otherwise they are gray. Colors are as follows: P1, P2, P2b, P3, P4, P5 and stems of predicted rho-independent transcription terminators. Putative Shine-Dalgarno and start codons are colored green. Start codons are derived from RefSeq annotations, while Shine-Dalgarno sequences were estimated manually based on annotated start codons. Stems (except for terminators) are also indicated at the bottom of the alignment by angle brackets, where matching < and > denote base-paired columns. Below these angle brackets, the symbol "2" denotes base pairs exhibiting covariation, "1" denotes base pairs exhibiting compatible mutations, "0" denotes base pairs that are not observed to mutate and "?" denotes base pairs that have a significant frequency of non-canonical nucleotides for Watson-Crick or G-U pairs. Below these base pair annotation is the consensus sequence: "R" = "A" or "G", "Y" = "C" or "U", red nucleotides: nucleotide identity conserved more than 97% of the time, black nucleotides: 90%, gray nucleotides: 75%, red circle (•): nucleotide is present 97% of the time, black circle (•): 90%, gray circle (•): 75%, white circle (◦): 50%. Columns containing putative GNRA tetraloops are indicated below with "GNRA", while their receptors are indicated below with "-TR". Columns that often, but not always, contain a likely Shine-Dalgarno are indicated below with "SD+++". The following Moco RNAs are not shown because they have an identical nucleotide sequence to other hits that are shown: Bce-1-1, Dha-1-4, Dha-1-8, Eco-10-1, Eco-11-1, Eco-12-1, Eco-13-1, Eco-16-1, Eco-4-1, Eco-5-1, Eco-7-1, Eco-8-1, Eco-9-1, Esi-1-1, Sen-3-1, Sfl-1-1, Tet-2-1, Tet-2-2, Vch-11-1, Vch-14-1, Vch-2-1, Vch-4-1, Vch-5-1, Vch-6-1, Vch-9-1, Ype-1-1, Ype-2-1, Ype-3-1, Ype-4-1, Ype-5-1, Ype-6-1, Ype-7-1, Ype-8-1, env-3

alignment positions 1 · · · 160

```
 1Mth-1-1  UCCAUAAGCGACUUC.CGAGUUG.UCCUC.UCC....C........................ACC...............................................................................................
  Pca-1-1  ACCUAUGUGUUCCUC.CAUGUGC.UG.CG.UUUUGGCGGA...................... UAC...............................................................................................
  Tth-1-1  GGAUGGCCUCGCCUC.CGAGCCC.GG.GU.AG....ACCC......................CCU...............................................................................................
  Tth-2-1  GGAUGGCCUUGCCUC.CGAGCCC.GG.GU.AG....ACCC......................CCU...............................................................................................
  Sth-1-1  GAUGUGAUCACGUCC.CGAGUCA.UG.CG.ACCUGUACCC......................GAUG..............................................................................................
  Ama-1-1  AGGUAACAAAAACUC.CGAGCUC.UA.AU.ACCUAAAGUU......................CGAAC.............................................................................................
  Dha-2-1  AAUAGUGAAAUCUUU.CGAAUCG.UUUCC.CGCUAAAGC.......................CAU...............................................................................................
  Dha-1-1  AAUAGUGAAAUCUUU.CGAAUCG.UUUCC.CGCUAAAGC.......................CAU...............................................................................................
  Dha-1-2  AAUAGUGAAAUCUUU.CGAAUCG.UUUCC.CGCUAAAGC.......................CAU...............................................................................................
  Aba-1-1  CUAGGAACGUGUUUC.CGACGUU.CG.CU.GCCUACGG........................AAUA..............................................................................................
  Dha-2-2  AAUUGAAUAGUUUUC.CGAGCUC.CU.UU.GUCUAAAAGA......................CUA...............................................................................................
  Dha-1-3  AAUUGAAUAGUUUUC.CGAGCUC.CU.UU.GUCUAAAAGA......................CUA...............................................................................................
  Dre-1-1  UGAUAUUUACACUUC.CGAUUUA.CU.AU.AU....UCCU......................ACU...............................................................................................
  Dha-2-3  AUAAUAGGGAAUGGC.CGAGCCA.UA.UU.UCCUACUGA.......................CUGAAGCAAGUCUGACGCUA..............................................................................
  Pin-1-1  UUUUAAAACCAGCUC.CGAGCUU.AA.UAAAACUAAGUUUCCUU..................UUUA..............................................................................................
  Rsp-1-1  CAUUAAGCAAUACAC.CGAGUGA.CA.GGGCGCUAAGUGG......................UCU...............................................................................................
  Cps-1-1  UUUAUUUUAUUACAC.CGAGCUA.UC.AC.AGCUAACAUUGUUUU.AUUUUUUGGCU.....AAAUUA............................................................................................
  Hdu-1-1  UAACUUAAACGAUAC.CGAGCUU.GU.UA.GCCUAAGUC.......................UAUU..............................................................................................
  Apl-1-1  UAACUUACAGGAUAC.CGAGCUU.GU.AU.GCCUAAGUC.......................UUA...............................................................................................
 2Hdu-1-2  AUUAUUUUUAGACUC.CGAGCCU.AC.UU.UCCUAAG.........................UCU...............................................................................................
  Mha-1-1  AUUUAUUGAAGACUC.CGAGCAU.UA.UU.UCCUAACUCUAAAAAUU...............AAAGAC............................................................................................
  Apl-1-2  UUUAUCAACAGACUC.CGAGCUU.GU.GA.ACCUAACUUCU.........CGGUCG.UUUUACAAAUUUUCGU.......................................................................................
  Msu-1-1  AGAAAUUUUAUAUAC.CGAGCUU.GU.AC.GCCUAAGUUUUUUAAA................UUUA..............................................................................................
  Hso-1-1  GUUAUCGCAUAACUC.CGAGCUU.GU.UU.ACCUAAGUU.......................GAAA..............................................................................................
  Hso-2-1  GUUAUCGCAUAACUC.CGAGCUU.GU.UU.ACCUAAGUU.......................GAAA..............................................................................................
  Hin-3-1  UUUAACGCAUAACUC.CGAGCUU.GU.UUAGCCUAAGUUU......................CACAAA............................................................................................
  Hin-4-1  UUUAACGCAUAACUC.CGAGCUU.GU.UUAGCCUAAGUUUC.....................AUA...............................................................................................
  Hin-2-1  UUUAACGCAUAACUC.CGAGCUU.GU.UUAGCCUAAGUUUC.....................AUC...............................................................................................
  Hin-1-1  UUUAACGCAUAACUC.CGAGCUU.GU.UUAGCCUAAGUUUC.....................AGAA..............................................................................................
  Pmu-1-1  AUUAUCGCAUAACUC.CGAGCUU.AU.UUAACCUACGUUUCUA...................CAA...............................................................................................
  Asu-1-1  UCAAUUUUCGUUUAC.CGAGCUU.GU.GU.GCCUAAGUUCUU..CCGG..............UUAU..............................................................................................

           ..<<<<<<<<<<<<<.<..<<<<.<<.<<.<<<...<<<<<<<<<<<<<<<<<<<<<<<<<<..................................................................................................
           ..??????????2?2.1..222?.??.??.?21...?2222??????????2?222??0000..................................................................................................
           ●●●●●●●●●●●●CUC-CGAGCY●-●●-●●-RCCUA●R●●○----------------------●●●○----------------------------------------------------------------------------------------------
                                             -TR-
```

```
Hso-2-2   AAAAACUGACGACUC.CGAGCUU.GU.UG.ACCUAAGUU.......................GCAA..............................................................................................
Hso-1-2   AAAAACUGACGACUC.CGAGCUU.GU.UG.ACCUAAGUU.......................GCAA..............................................................................................
Mha-1-2   CAACUUAGAGGAUAC.CGAGCUU.GU.GA.GCCUAAGUGUCUGCU.................GAG...............................................................................................
Dha-2-4   UAUGUUUAUGUGCUC.CGAAACU.GG.AA.ACCUAAAGU.......................UA................................................................................................
Dha-1-5   UAUGUUUAUGUGCUC.CGAAACU.GG.AA.ACCUAAAGU.......................UA................................................................................................
Csp-1-1   AUAACUAAAUAUCUC.CGAGUUC.AU.CGAUCCUAAUAC.......................CUGUUUUC..........................................................................................
Dps-1-1   UUAAGAAUAUAACUC.CGAGCUA.AA.AG.GCCUUCGGUUU.....................CUGCAU............................................................................................
Mth-1-2   AACUCUAUAAACCUC.CGAGCCA.GU.GG.GCCUAAGGUU......................UUC...............................................................................................
Ame-1-1   AAUGUUACGAAUUUC.CGAGUGA.GA.UU.AUCUAAGGC.......................GAU...............................................................................................
Ame-1-2   AAUAAAAUUUAUUUC.CGAGUUA.CU.UU.UCCUAAGGGCA.....................AAC...............................................................................................
Ahy-1-1   AAACUAGAUGAACUC.CGAGCGG.UU.UU.GCCUAAGUC.......................CUCAU.............................................................................................
env-1     AAACUAGAUGAACUC.CGAGCGG.UU.UU.GCCUAAGUC.......................CUCAU.............................................................................................
Plu-1-1   CUAUGGAAUUAACUC.CUAGCCU.CU.GC.AUCUAAGUCACU....................UUUUACAC..........................................................................................
Sgl-1-1   AGGUAUACAUAACUC.CUAGCCU.UU.AC.GCCUAAGUGGCCC...................GUCAU.............................................................................................
Ymo-1-1   ACUGAACAUAAACUC.CUAGCCU.UG.GU.GCCUACGUUGUCC.....CGAGCCGCG.....AAUA..............................................................................................
Yps-2-1   GCUGAACAUAAACUC.CUAGCCU.UA.GC.GUCUACGUUGUC....................UUUUUUGUAUU.......................................................................................
Yps-1-1   ACUGAACAUAAACUC.CUAGCCU.UA.GC.GUCUACGUUGUC....................UUUUUUGUAUU.......................................................................................
Yfr-1-1   ACUGAACAUAAACUC.CUAGCCU.UA.GU.GUCUACGUUGUU....................GUGUUUUUCU........................................................................................
Yin-1-1   ACUGAACAUAAACUC.CUAGCCU.UG.GC.GUCUACGUUGUCUCG.................UUCG..............................................................................................
Ybe-1-1   ACUGAACAUAAACUC.CUAGCCU.UG.GU.GUCUACGUUGUCCGGUGUCGCGAA........UACGUUU...........................................................................................
Spr-1-1   CUGAAACCAAAACUC.CUAGCCU.CU.GG.GCCUACGUUGUUC...................UUGU..............................................................................................
Eca-1-1   AUGAGAAUCAAACUC.CUAGCCU.UG.GU.GCCUAAGUGAUGCGC.................UUAUU.............................................................................................
Esp-1-1   GAUGAAAACAUACACUCUAGCCU.UG.AC.GCCUGUGUC.......................ACAU..............................................................................................
env-2     GACGAGUAUAAACACUCUAGCCU.CU.GC.ACCUGGGUC.......................AAAU..............................................................................................
Eco-17-1  AUAACCACUAAACACUCUAGCCU.CU.GC.ACCUGGGUC.......................AACU..............................................................................................
Eco-6-1   AUAACCACUAAACACUCUAGCCU.CU.GC.ACCUGGGUC.......................AACU..............................................................................................
Eco-3-1   AUAACCACUAAACACUCUAGCCU.CU.GC.ACCUGGGUC.......................AACU..............................................................................................
Eco-1-1   AUAACCACUAAACACUCUAGCCU.CU.GC.ACCUGGGUC.......................AACU..............................................................................................
Sbo-2-1   UUAACCACUAAACACUCUAGCCU.CU.GC.ACCUGGGUC.......................AACU..............................................................................................
Sso-1-1   UUAACCACUAAACACUCUAGCCU.CU.GC.ACCUGGGUC.......................AACU..............................................................................................
Sfl-3-1   UUAACCACUAAACACUCUAGCCU.CU.GC.ACCUGGGUC.......................AACU..............................................................................................
Eco-2-1   UUAACCACUAAACACUCUAGCCU.CU.GC.ACCUGGGUC.......................AACU..............................................................................................
Sfl-2-1   UUAACCACUAAACACUCUAGCCU.CU.GC.ACCUGGGUC.......................AACU..............................................................................................
Sdy-1-1   UUAACCACUAAACACUCUAGCCU.CU.GC.ACCUAGGUC.......................AACU..............................................................................................
Sbo-1-1   UUAACCACUAAACACUCUAGCCU.CU.GC.ACCUAGGUC.......................AACU..............................................................................................
Eco-15-1  UUAACCACUAAACACUCUAGCCU.CU.GC.ACCUGGGUC.......................AACU..............................................................................................
Eco-14-1  UUAACCACUAAACACUYUAGCCU.CU.GC.ACCUGGGUC.......................AACU..............................................................................................
Sty-1-1   AACCACAGUAAACACUCUAGCCU.CU.GC.ACCUGGGUCA......................UUUU..............................................................................................
Sen-1-1   AACCACAAUAAACACUCUAGCCU.CU.GC.ACUUGGGUCA......................UUUU..............................................................................................
Sen-4-1   AACCACAGUAAACACUCUAGCCU.CU.GC.ACCUGGGUCA......................UUUU..............................................................................................
Sen-2-1   AACCACAAUAAACACUCUAGCCU.CU.GC.ACCUGGGUCA......................UUUU..............................................................................................
Sam-1-1   UCAAUGACGGUUCUC.CGAGCUC.CU.GC.ACCUAAGUC.......................AAUG..............................................................................................
Spe-1-1   AGGUUGACGGUUCUC.CGAGCUU.CU.UA.CCCUACGUC.G.....................AGA...............................................................................................
Ssp-4-1   GAAACGACGGUUCUC.CGAGCUU.UC.UC.UCCUACGUC.......................CACA..............................................................................................
Swo-2-1   UCACUGACGGUUCUC.CGAGCUU.UU.UG.UCCUACGUC.......................AACA..............................................................................................
Ssp-5-1   CCAGCGACGGUUCUC.CGAGCUU.GC.CU.ACCUACGUC.......................AAUA..............................................................................................
Ssp-5-2   CCAGCGACGGUUCUC.CGAGCUU.GC.CU.ACCUACGUC.......................AAUA..............................................................................................
Spu-1-1   CCAGCGACGGUUCUC.CGAGCUU.GC.CU.ACCUACGUC.......................AAUA..............................................................................................
Sba-1-1   CCGGUGACGGUUCUC.CGAGCUU.GC.CU.ACCUAAGUC.......................GAAA..............................................................................................
Sba-2-1   CCGGUGACGGUUCUC.CGAGCUU.GC.CU.ACCUAAGUC.......................GAAA..............................................................................................
Ssp-1-1   CGAAUGACGGUUCUC.CGAGCUU.GC.CU.ACCUAAGUC.......................GACA..............................................................................................
env-4     CGAAUGACGGUUCUC.CGAGCUU.GC.CU.ACCUAAGUC.......................GACA..............................................................................................
Ssp-2-1   CGAAUGACGGUUCUC.CGAGCUU.GC.CU.ACCUAAGUC.......................GACA..............................................................................................
Ssp-3-1   CGAAUGACGGUUCUC.CGAGCUU.GC.CU.ACCUAAGUC.......................GACA..............................................................................................
Son-1-1   CAGCUGACGGUUCUC.CGAGCUU.GC.CU.ACCUAAGUC.......................AACA..............................................................................................
env-5     CGGCUGACGGUUCUC.CGAGCUU.GC.CU.ACCUAAGUC.......................AACA..............................................................................................
Sfr-1-1   GAUCUGACGGUUCUC.CGAGCUU.UU.UG.CCCUACGUC.......................AAU...............................................................................................
Sde-1-1   GCAUUGACGGUUCUC.CGAGCUU.CU.UA.UCCUAAGUC.......................UUUA..............................................................................................
Vch-3-1   GCUUGGCCUUAACUC.CGAGCUU.AC.CG.CGCUAAGUUUA.....................AACCUG............................................................................................
Vch-1-1   GCUUGGCCUUAACUC.CGAGCUU.AC.CG.CGCUAAGUUUAA....................ACCU..............................................................................................
Vch-8-1   GCUUGGCCUUAACUC.CGAGCUU.AC.CG.CGCUAAGUUUAA....................ACCU..............................................................................................

          ..<<<<<<<<<<<<<.<..<<<<.<<.<<.<<<...<<<<<<<<<<<<<<<<<<<<<<<<<<..................................................................................................
          ..??????????2?2.1..222?.??.??.?21...?2222??????????2?222??0000..................................................................................................
          ●●●●●●●●●●●●CUC-CGAGCY●-●●-●●-RCCUA●R●●○----------------------●●●○----------------------------------------------------------------------------------------------
                                        -TR-
```

```
Vch-10-1   GCUUGGCCUUAACUC.CGAGCUU.AC.CG.CGCUAAGUUUAA....................ACCU.............................................................................................................
Vch-13-1   GCUUGGCCUUAACUC.CGAGCUU.AC.CG.CGCUAAGUUUAA....................ACCU.............................................................................................................
Vch-7-1    GCUUGGCCUUAACUC.CGAGCUU.AC.CG.CGCUAAGUUUAA....................ACCU.............................................................................................................
Vch-12-1   GCUUGGCCUUAACUC.CGAGCUU.AC.CG.CGCUAAGUUUAA....................ACCU.............................................................................................................
Vvu-1-1    GGGUUGCCAUAACUC.CGAGCUU.AA.CU.UGCUAAGUUAUUUUAUU...............UCAAU............................................................................................................
Vvu-2-1    GGGUUGCCAUAACUC.CGAGCUU.AA.CU.UGCUAAGUUAUUUUAUU...............UCAAU............................................................................................................
Vsp-3-1    CAGUUGCCAUAACUC.CGAGCUC.UC.GCAUGCUAAGUCG.CACAGUG..............GUAUU............................................................................................................
Vsp-2-1    CAGUUGCCAUAACUC.CGAGCUC.UC.GCAUGCUAAGUCG.CACAGUG..............GUAUU............................................................................................................
Vpa-1-1    UGGUUGCCAUAACUC.CGAGCUC.AC.UA.UGCUAAGUCC......................GA...............................................................................................................
Vsp-1-1    UGGUUGCCAUAACUC.CGAGCUC.AC.UAUUGCUAAGUCC......................GA...............................................................................................................
Val-1-1    UGGUUGCCAUAACUC.CGAGCUC.AC.UAUUGCUAAGUCC......................UU...............................................................................................................
Vfi-1-1    UGGCUGCCAUAACUC.CGAGCUC.AA.GC.UACUAAGUUG....UUUU..............UUAA.............................................................................................................
Psp-1-1    UAACUGCCAUAACUC.CGAGCAU.UC.UU.CACUAAGUUA.CUAAGCGAUGU..........UGGACUUAUAUAUAUAU................................................................................................
Van-1-1    UAACUGCCAUAACUC.CGAGCAU.UU.UU.CACUAAGUUA.CUAAGCGAUGU..........UGGACAUUAAUAUAU..................................................................................................
Ppr-3-1    UAGCUGCCAUAACUC.CGAGCUU.UA.GU.CACUACGUUUUCUGC.UUU.............UCUU.............................................................................................................
Ppr-2-1    UAGCUGCCAUAACUC.CGAGCUU.UG.GU.CACUACGUUUUCUGUUUU..............UCUU.............................................................................................................
Dre-1-2    GUAAUAACUUGUCUC.CGAGCCG.CU.UG.GUCUAAGGC.......................ACUU.............................................................................................................
Swo-1-1    AUAAUUAUGGCUCGC.CGAGUUU.UU.CC.GUCUAAGGUUUU....................UUA..............................................................................................................
Pat-1-1    CAGGUGGUUUAACUC.CGAGCCU.UC.AU.ACCUAAAUC.......................AGAA.............................................................................................................
Pat-1-2    CAGGUGGUUUAACUC.CGAGCCU.UC.AU.ACCUAAAUC.......................AGAA.............................................................................................................
Swo-1-2    UCAAGAGCUUAACUC.CGAGCUA.UC.CC.GCCUAAAGCG......................AAA..............................................................................................................
env-6      AACUGAUAAUUUCUC.CGAUUCA.UUCAU.GCCUAAUGCG......................AC...............................................................................................................
env-7      AACUGAUAAUUUCUC.CGAUUCA.UUCAU.GCCUAAUGCG......................AC...............................................................................................................
Swo-1-3    AAGCGUAAUUAACUC.CGACUCA.CA.CCAGCCUAAGGU.......................AAUG.............................................................................................................
Swo-1-4    AAGCGUAAUUAACUC.CGACUCA.CA.CCAGCCUAAGGU.......................AAUG.............................................................................................................
Chy-1-1    AAAAAUAAGCUGCUC.CGAGUAU.UU.UC.ACCUAAAGCU......................UUA..............................................................................................................
Swo-1-5    AAUAGUUAAUGUCUU.CGAGCCU.UU.UA.UCCUAUAGCUU.....................U................................................................................................................
Mth-1-3    UUGAAUAGUCAUCUC.CGAGCCG.GU.GC.UCCUAAGGGC......................UGUC.............................................................................................................
Swo-1-6    UUUACAUACUUUUUC.CGAACUU.GA.GG.ACCUAAAGC.......................AAAU.............................................................................................................
Csa-1-1    UUUUGAAAAUCUUUC.CGAGCCA.UU.GCAACCUAAAAAGAGGGG.................UUUU.............................................................................................................
Dac-1-1    CAGCCCUUAAAUCUC.CGAGCCGCUA.CC.ACCUACGAGC......................GU...............................................................................................................
Dac-1-2    AAAUAGUUACAACUC.CGAGUCU.GU.UC.ACCUACCGG.......................CACAU............................................................................................................
Hne-1-1    GUUGGAAAUUCGCUC.CGAGCCG.UU.CC.GCCUAAAGCGCCG...................UUUCC............................................................................................................
Swo-1-7    UAACUGAAUAAUCUC.CGAGCUG.UA.CU.GCCUU.CAGUC.....................AC...............................................................................................................
Dac-1-3    UACUAUCAAGAUCUC.CGAGCCU.GC.CCCCCCUACGGAGG.....................UUG..............................................................................................................
Gsp-1-1    GACAAAACAAGUCUC.CGAGCCU.CC.AUUGCCUAAAGGG......................CGUCAAA..........................................................................................................
Gsp-1-2    UGAUUCUCAACUCUC.CGAGCCA.CG.CC.GCCUAAAGGG......................UAGUUU...........................................................................................................
Gsp-1-3    UAAAAACUAAAUCUC.CGAGUCG.UAAUU.GCCUACCGGGGAUGACA...............AAGC.............................................................................................................
Pca-1-2    AUGCGCGGCAGUCUC.CGAGCCA.GG.UCCGCCUAAGGGGA.....................UGG..............................................................................................................
Gsp-1-4    UGCUCGAACUUUCUC.CGAGCCU.GU.CC.GCCUACCGGGAAU....................................................................................................................................
Ppr-1-1    GGAGCAAUCUGUCUC.CGAGCCU.GC.CC.UCCUACCGGG......................ACAC.............................................................................................................
Gur-1-1    ACACCUGUUUUUCUC.CGAGCCU.CC.UC.ACCUACAGGG......................CAUCC............................................................................................................
Gur-1-2    AAAUUGUCGGCUCUC.CGAGCCG.CC.GU.ACCCACCAGGG.....................CAACC............................................................................................................
Gsu-1-1    AUAUUUUGUUAUCUC.CGAGCCC.AU.CC.GCCUACCAGGA.....................ACA..............................................................................................................
Gme-1-1    UACUAGUUUUUUCUC.CGAUCCG.UC.AU.ACCUACCAGGC.....................GCAGA............................................................................................................
Gur-1-3    GAAACUGACGCUCUC.CGAGCUG.UA.GC.GCCUAAAAGGU.....................UUGU.............................................................................................................
Pca-1-3    AAUACUACCAAUCUC.CGAGCCU.CG.CU.GCCUACUGGGA.....................AGG..............................................................................................................
Gme-1-2    ACAACCAACACUCUC.CGAGCCU.GC.AA.GCCUAACAGG......................GAAA.............................................................................................................
Pca-1-4    UCAAGAAUGCGCCUC.CGAGCCU.GA.UG.CCCUAAAAGAGAU...................UUU..............................................................................................................
Ppr-1-2    AUGCCAUGUUAUCUC.CGAGCCU.UG.CC.ACCUAAAAGG......................AAAA.............................................................................................................
Pca-1-5    UACAGAACACGUCUC.CGAGUCU.U..AA.GCCUAAAAGG......................AUGGA............................................................................................................
Gur-1-4    CAAACACAUCAUCUC.CGAGCCU.UU.CA.GCCUAAAAGG......................AUAAA............................................................................................................
Gsp-1-5    UUCAUGUAUCAUCUC.CGAGCCG.UG.CA.GCCUAACAGG......................UUC..............................................................................................................
Gme-1-3    AUUUAAAACCGUCUC.CGAGCCG.CG.GC.GCCUAAAGGGA.....................ACA..............................................................................................................
Gme-1-4    AUUUAAAACCGUCUC.CGAGCUG.CG.GC.GCCUAAAGGGA.....................ACA..............................................................................................................
Csp-2-1    CGAUGAAACUCGCUC.CGAGCGU.CC.CG.GCCUAACGCGC.....................AUCC.............................................................................................................
env-8      UAAUUAUGACUUCUC.CGAGCCU.UU.UU.GCCUAAAG........................GAAA.............................................................................................................
Tet-1-1    AGGAGAAUAACUCUC.CGAGCCU.UU.AU.GUCUAAGGGGC.....................GAAGA............................................................................................................
env-9      AAAUUUGUAAAUCUC.CGAUUUG.CC.GUGACCUAAAGUG......................UC...............................................................................................................
env-10     AAUCUACUUAAUCUC.CGAUUCG.CU.UACACCUAAAGU.......................AUAA.............................................................................................................
Swo-1-8    AAGAAUAUAUAUCUC.CGAGCUG.GG.GA.GUCUAAGGC.......................GAA..............................................................................................................

           ..<<<<<<<<<<<<<.<..<<<<.<<.<<.<<<...<<<<<<<<<<<<<<<<<<<<<<<<<<.................................................................................................................
           ..??????????2?2.1..222?.??.??.?21...?2222??????????2?222??0000.................................................................................................................
           ●●●●●●●●●●●●CUC-CGAGCY●-●●-●●-RCCUA●R●●○----------------------●●●○-------------------------------------------------------------------------------------------------------------
                                               -TR-
```

```
Swo-1-9     GAAUAUCAAGUUCUC.CGAGCUU.UU.AG.AUCUAAGGU.......................UU................................................................................................
Swo-1-10    UUGAAUAUUUGUUUC.CAAGCCU.UU.GA.ACCUAAGGUU......................UAU...............................................................................................
Swo-1-11    ACAAUUGUAUAGCUC.CAAGCCU.UU.AG.ACCUAAAGC.......................AAA...............................................................................................
Dha-1-6     AAUAUAAUAUAUCUC.CAAGCUG.GU.GAGACCUAAGGG.......................AAU...............................................................................................
Dha-2-5     CUGAAAAACGAUCUC.CGAGCCG.GAAUGAACCUAAAGAG......................CAA...............................................................................................
Dha-1-7     ACUGAAAAAGAUCUC.CGAGCCG.GAAUGAACCUAAAGAG......................CAA...............................................................................................
Mth-1-4     AAAUAUUGACAUCUC.CGAGCCG.GU.UUAACCUAAGGA.......................GCGA..............................................................................................
Dha-2-6     ..........AUCUC.CGAGCCG.GAGCA.ACCUAAAGA.......................GUCA..............................................................................................
Dha-2-7     CUUCAUAAUGAUCUC.CGAGCCG.GAGCA.ACCUAAAGA.......................GUCA..............................................................................................
Mth-1-5     AAUAUUUACAUCCUC.UGAGCCA.UC.CUGGCCUAAAGG.......................AGCAA.............................................................................................
Cpe-2-1     AUUAGUAUAACUCUC.CAAGCUU.AA.AG.AUCUAAGGAG......................AAGG..............................................................................................
Cpe-1-1     AUUAGUAUAAAUCUC.CAAGCUU.AA.AG.AUCUAAGGAG......................AAGG..............................................................................................
Cpe-3-1     AUUAGUAUAACUCUC.CAAGCUU.AA.AG.AUCUAAGGAG......................AAGG..............................................................................................
Dre-1-3     AAAUUAAUAGUACUC.CGAGCUU.AG.GC.ACCUAAGAU.......................AGUA..............................................................................................
Swo-1-12    AAACUAAAUAUACUC.CGAGCCU.GA.AA.ACCU..GGUA......................AUAA..............................................................................................
Tet-1-2     UUAUUACUUAUUCUC.CGAGUCU.AU.AA.UCCUAAGGC.......................AAGA..............................................................................................
Swo-1-13    ACUUAAUAUGUAUUC.CGAGCCC.UC.UUAACCUGCAGA.......................CAAA..............................................................................................
Swo-1-14    AAAAGAAUAGAGCUC.CGAGCCU.UAUUA.AUCUAAAGAC......................UGCG..............................................................................................
Swo-1-15    GAAUAGAACCGGCUC.CAAGCCU.UU.GA.GCCUAAUGACUGAA.ACUCA............UUUGUCAGGGAGAAUCGGACUUUUAUGGUGGAAAAAGCCUGAAUCCCUGAGCCCGUUAAAAAAGUAUAAAAAUUAUGGACGAUUAAUGCAUUUUAUAC
Sfu-1-1     AGAACGGUCGCUCCC.CGAGCCG.CC.G........GGUC......................UAA...............................................................................................
            ..<<<<<<<<<<<<<.<..<<<<.<<.<<.<<<...<<<<<<<<<<<<<<<<<<<<<<<<<<..................................................................................................
            ..??????????2?2.1..222?.??.??.?21...?2222??????????2?222??0000..................................................................................................
            ●●●●●●●●●●●●CUC-CGAGCY●-●●-●●-RCCUA●R●●○----------------------●●●○----------------------------------------------------------------------------------------------
                                             -TR-
```

alignment positions 161 · · · 320

```
[1]Mth-1-1  ..........................................G...GGAAG.GGCA.AAC...................................................................................................G
Pca-1-1     ........................................UCCUGUGAGCG.UGGC.ACC...................................................................................................G
Tth-1-1     ........................................GGGC...CU.C.CCGG.GCC...GGGCC.................................CACG...............................................GGCCC...
Tth-2-1     ........................................GGGC...CU.C.CCGG.GCC...GGGCC.................................CACG...............................................GGCCC...
Sth-1-1     ........................................GGGUAUGGUCG.CCUG.ACCG..GUCCCGGU..............................CGU.............................................ACC.GGGC...
Ama-1-1     ........................................AACUAUGGUAC.UGGA.GCC...GCAACU................................GAGA..............................................AGUUGC...
Dha-2-1     .........................................GCUAUGCGGG.GGCG.AUC...UGAGC.................................AAAG...............................................GCUCA...
Dha-1-1     .........................................GCUAUGCGGG.GGCG.AUC...UGAGC.................................AAAG...............................................GCUCA...
Dha-1-2     .........................................GCUAUGCGGG.GGCG.AUC...UGAGC.................................AAAG...............................................GCUCA...
Aba-1-1     ..........................................CUAAGGCAU.CGGG.CGCG..GUAUCCAUCU............................UUGU..........................................AGAUGGAU.C...
Dha-2-2     ........................................UCUCAUGACGG.AGGA.GCC...UGAGC.................................AAACC..............................................GCUCA...
Dha-1-3     ........................................UCUCAUGACGG.AGGA.GCC...UGAGC.................................AAACC..............................................GCUCA...
Dre-1-1     ........................................GGGA...AU.U.AGUA.AAC....................................................................................................
Dha-2-3     .........................................UCUACGGAAU.UUUG.GCC...GUUUCAGA..............................AAUA............................................UCUGAAAC...
Pin-1-1     ....................................AGGUAAAUAAGUCUG.UUAA.GCCG..CUUGU.CUGAC...........................AUUUUG.......................................GUCAGCGCAAG...
Rsp-1-1     ........................................CCAUAAGCCCU.UGUC.ACC...UCGC..................................AGUA................................................GCGA...
Cps-1-1     .......................AGCCAACUAUUCGAAGCAUUCAUGUUGU.GACAUGCC...GUGACUAAAA.CCA.UG.....................UAUU...................................UAUUGGCUUUUAGUUGC...
Hdu-1-1     .........................................GAUAAGGC.A.GCAAAGCC...UGCAAUAUAAGCGGUCG..AGUUGA.UG..........AAA.........................UAUUUAGCUAUUAAUCGCUUAUUUUGCA...
Apl-1-1     .........................................GAUAAGGUGU.GCAA.GCC...UGUGGCAAGCGGUU..........AAAAUU........CACAA.....................AAUUUUUGCAAAAUCAGACCGCUU.ACACA...
[2]Hdu-1-2  ...........................................UAUUAAAU.UUGA.GCAAAU..............................................................................................CAU
Mha-1-1     .................................AAAUUUUUGAUAUGGAAU.UUAU.GCCA..GUAACAGC..............................AUAAA............................................GCGUU.C...
Apl-1-2     ..................UAAAUUCGACCGCUUUUUGUAAGAAUAUGGU.A.GCAAUGCCU..GUAAUUAGCUUA..........................UACG........................................U.AGCUGAUU.C...
Msu-1-1     ...................................UUUGAAAAUAAGGCG..GCAAUGCC...AGCAAUGA..............................AUU.............................................UUAUUGCU...
Hso-1-1     .........................................AAUAAGGA.A.ACAAUGCC...AGCAA.................................AUAUAA.............................................UUGCU...
Hso-2-1     .........................................AAUAAGGA.A.ACAAUGCC...AGCAA.................................AUAUAA.............................................UUGCU...
Hin-3-1     ........................................AAAUACGGU.G.GCAA.GCC...AAUAACG...............................CGGUA............................................CGUUAUU...
Hin-4-1     .......................................GAAAUACGGU.G.GCAA.GCC...AAUAACG...............................CGCUA............................................CGUUAUU...
Hin-2-1     .......................................GAAAUACGGC.G.ACAA.GCC...AAUAACGC..............................CGU.............................................GUGUUAUU...
Hin-1-1     .......................................GAAAUACGGU.G.GCAA.GCC...AAUAACGC..............................CGU.............................................GUGUUAUU...
Pmu-1-1     .....................................UAGAAAUAAGGU.G.GUAAUGCC...AAUAGCAUAA............................AAC...........................................UUGUGCUAUU...
Asu-1-1     ................................CCGGCGCGGAAUAAGGC.A.GCAACGCC...AGCGAUG...............................CGGUU............................................UAUCGCU...
Hso-2-2     .........................................AAUAAGGU.U.GCAA.GCC...UGUAACAUU.............................GUCA...........................................AAUGUUGCA...
Hso-1-2     .........................................AAUAAGGU.U.GCAA.GCC...UGUAACAUU.............................GUCA...........................................AAUGUUGCA...

            ..................>>>>>>>>>>>>>>>>>>>>>>>>>>..>>>>>.>>>>.>>....<<<<<<<<<<<<<<<<<<<<<<<<<<<<<<<<<<<<<<..................>>>>>>>>>>>>>>>>>>>>>>>>>>>>>>>>>>>>>>...
            ..................0000??222?2??????????2222?..12???.???2.22....22222?22????????????????2?22??00???122..................221???00??22?2????????????????22?22222...
            ----------------------------------------○●●YA●GR●○●-●●●R-GCC----------------------------------------------------------------------------------------------------
                                                       -TR
```

```
Mha-1-2     ...................................AGCAGAGAUAUGGU.A.GCAA.GCC....GUAAAG.CACCUACUACAAGCGGUCAUUUUUUCCUAACAUU..............UUG..CAAAAAUUACCGUC.......GGUGCUUUUACC...
Dha-2-4     .........................................GCUAUGGUUUACCAG..UC...GUGCA.................................UUC................................................UGCAC...
Dha-1-5     .........................................GCUAUGGUUUACCAG..UC...GUGCA.................................UUC................................................UGCAC...
Csp-1-1     .........................................GUAAUGGUCU.AUGA.GCC..................................................................................................UG
Dps-1-1     .......................................GGAUCAUGGUCU.GC.A.GCC..................................................................................................GU
Mth-1-2     ........................................GGCUAUGGC.U.GCUG.GCC...UC.CUGCCUG............................CUUGC.........................................CAGGCAGCGA...
Ame-1-1     .........................................GCUACGAU.G.ACUU.ACC...................................................................................................U
Ame-1-2     .......................................UGUCUAAGGU.U.AGUG.GCC...................................................................................................G
Ahy-1-1     .........................................GAUAGGGGGA.AACC.GCCG..UCAGC.CGA.............................UGU............................................UCGAGCUGU...
env-1       .........................................GAUAGGGGGA.AACC.GCCG..UCAGC.CGA.............................UGU............................................UCGAGCUGU...
Plu-1-1     ......................................AGCGAUAUGAUGU.UUUG.GCC...GUGAC.................................CACUGA.............................................GUCAC...
Sgl-1-1     .....................................GAGCCAUAGGGGGU.UCUG.GCC...GUGGC.................................GAA................................................GCCAC...
Ymo-1-1     .......................UGCGUUUCGUUUAUGGACAAUAGGGUGC.UCUG.GCC...GUGGCU................................UUA...............................................AGCCAC...
Yps-2-1     ......................................GGCAAUAUGAUGU.UCUG.GCC...GUGGC.................................CUUAA..............................................GCCAC...
Yps-1-1     ......................................GGCAAUAUGAUGU.UCUG.GCC...GUGGC.................................CUUAA..............................................GCCAC...
Yfr-1-1     ......................................AACAAUAUGAUGC.UCUG.GCC...GUGGCU................................UUA...............................................AGCCAC...
Yin-1-1     ...................................CGA.ACAAUAUGAUGC.UCUG.GCC...GUGGCU................................UUA...............................................AGCCAC...
Ybe-1-1     ..........................UUCGUU.UACC.GGCAAUAUGAUGC.UCUG.GCC...GUGGCU................................UUA...............................................AGCCAC...
Spr-1-1     .....................................GAACAAUAUGGCGC.UUGG.GCC...GUGGC.G...............................AAGU.............................................CAGCCAC...
Eca-1-1     ...................................GCGCGUGAUACGGUGC.CUUG.GCC...GUGGCC................................UUCGU.............................................GGUCAC...
Esp-1-1     .........................................GAUAUGGCGC.CCUG.GCC...GUGGCG................................GUUU..............................................CGCCAC...
env-2       .........................................GAUACGGUGC.UUUG.GCC...GUGACAAGACUU..........................GAAA........................................AAG.UUUGUCAC...
Eco-17-1    .........................................GAUACGGUGC.UUUG.GCC...GUGACAAUGCU...........................CGUAA........................................AG.AUUGCCAC...
Eco-6-1     .........................................GAUACGGUGC.UUUG.GCC...GUGACAAUGCU...........................CGUAA........................................AG.AUUGCCAC...
Eco-3-1     .........................................GAUACGGUGC.UUUG.GCC...GUGACAAUGCU...........................CGUAA........................................AG.AUUGCCAC...
Eco-1-1     .........................................GAUACGGUGC.UUUG.GCC...GUGACAAUGCU...........................CGUAA........................................AG.AUUGCCAC...
Sbo-2-1     .........................................GAUACGGUGC.UUUG.GCC...GUGACAAUGCU...........................CGUAA........................................AG.AUUGCCAC...
Sso-1-1     .........................................GAUACGGUGC.UUUG.GCC...GUGACAAUGCU...........................CGUAA........................................AG.AUUGCCAC...
Sfl-3-1     .........................................GAUACGGUGC.UUUG.GCC...GUGACAAUGCU...........................CGUAA........................................AG.AUUGCCAC...
Eco-2-1     .........................................GAUACGGUGC.UUUG.GCC...GUGACAAUGCU...........................CGUAA........................................AG.AUUGCCAC...
Sfl-2-1     .........................................GAUACGGUGC.UUUG.GCC...GUGACAAUGCU...........................CGUAA........................................AG.AUUGCCAC...
Sdy-1-1     .........................................GAUACGGUGC.UUUG.GCC...GUGACAAUGCU...........................CGUAA........................................AG.AUUGCCAC...
Sbo-1-1     .........................................GAUACGGUGC.UUUG.GCC...GUGACAAUGCU...........................CGUAA........................................AG.AUUGCCAC...
Eco-15-1    .........................................GAUAUGGUGC.UUUG.GCC...GUGACAAUGCU...........................CGUAA........................................AG.AUUGCCAC...
Eco-14-1    .........................................GAUAUGGUGC.UUUG.GCC...GUGACAAUGCU...........................CGUAA........................................AG.AUUGCCAC...
Sty-1-1     ........................................UGAUACGGUGC.UUUG.GCC...GUGACAA.CGU...........................UUA..........................................ACGUUUGCCAC...
Sen-1-1     ........................................UGAUAUGGUGC.UUUG.GCC...GUGACAA.CGU...........................UUA..........................................ACGUUUGCCAC...
Sen-4-1     ........................................UGAUAUGGUGC.UUUG.GCC...GUGACAA.CGU...........................UUA..........................................ACGUUUGCCAC...
Sen-2-1     ........................................UGAUAUGGUGC.UUUG.GCC...GUGACAA.CGU...........................UUA..........................................ACGUUUGCCAC...
Sam-1-1     .........................................GAUAUGGUGU.UUGA.GCC...GUGGC.................................GCGGU..............................................GCCAC...
Spe-1-1     .......................................CAGAUAAGGGUU.UUUA.GCC...GUGGC.................................AAUAAC.............................................GCCAC...
Ssp-4-1     .........................................GAUAGGGAGA.UUAA.GCC...GUGGCA................................AUAG..............................................UGCCAC...
Swo-2-1     .........................................GAUAUGGACG.CUAA.GCC...GUGGC.................................AAUAUC.............................................GCCAC...
Ssp-5-1     .........................................GAUAUGGAGG.UGUA.GCC...GUGGUA................................AUAA..............................................UGCCAC...
Ssp-5-2     .........................................GAUAUGGAGG.UGUA.GCC...GUGGUA................................AUAA..............................................UGCCAC...
Spu-1-1     .........................................GAUAUGGAGG.UGUA.GCC...GUGGUA................................AUAA..............................................UGCCAC...
Sba-1-1     .........................................GAUAUGGAGG.UGUA.GCC...GUGGUA................................AUAA..............................................UGCCAC...
Sba-2-1     .........................................GAUAUGGAGG.UGUA.GCC...GUGGUA................................AUAA..............................................UGCCAC...
Ssp-1-1     .........................................GAUAUGGGGG.UGUA.GCC...GUGGU.................................AAUAAG.............................................GCCAC...
env-4       .........................................GAUAUGGGGG.UGUA.GCC...GUGGU.................................AAUAAG.............................................GCCAC...
Ssp-2-1     .........................................GAUAUGGGGG.UGUA.GCC...GUGGC.................................AAUAAG.............................................GCCAC...
Ssp-3-1     .........................................GAUAUGGGGG.UGUA.GCC...GUGGC.................................AAUAAG.............................................GCCAC...
Son-1-1     .........................................GAUAUGGGGG.UGUA.GCC...GUGGC.................................AAUAAG.............................................GCCAC...
env-5       .........................................GAUAUGGUGG.UGUA.GCC...GUGGC.................................AAUAAG.............................................GCCAC...
Sfr-1-1     .........................................GAUAUGGGCA.GUUA.GCC...GUGGUA................................AUUU..............................................UGCCAC...
Sde-1-1     .........................................GAUACGGGUC.AUUA.GCC...GUGGUA................................AUAA..............................................UGCCAC...
Vch-3-1     .......................................UAAAUAUGCGUU.GUAA.GCCA..GUGGCA................................GAA...............................................UGUCAC...
Vch-1-1     ......................................UUAAAUAUGCGUU.GUAA.GCCA..GUGAC.................................CGCUU..............................................GUCAC...
Vch-8-1     ......................................UUAAAUAUGCGUU.GUAA.GCCA..GUGAC.................................CGCUU..............................................GUCAC...
Vch-10-1    ......................................UUAAAUAUGCGUU.GUAA.GCCA..GUGAC.................................CGCUU..............................................GUCAC...
Vch-13-1    ......................................UUAAAUAUGCGUU.GUAA.GCCA..GUGAC.................................CGCUU..............................................GUCAC...

            ..................>>>>>>>>>>>>>>>>>>>>>>>>>>..>>>>>.>>>>.>>....<<<<<<<<<<<<<<<<<<<<<<<<<<<<<<<<<<<<<<..................>>>>>>>>>>>>>>>>>>>>>>>>>>>>>>>>>>>>>>...
            ..................0000??222?2??????????2222?..12???.???2.22....22222?22????????????????2?22??00???122..................221???00??22?2????????????????22?22222...
            ----------------------------------------○●●YA●GR●○●-●●●R-GCC----------------------------------------------------------------------------------------------------
                                                       -TR
```

```
Vch-7-1     ......................................UUAAAUAUGCGUU.GUAA.GCCA..GUGAC.................................CGCUU...............................................GUCAC...
Vch-12-1    ......................................UUAAAUAUGCGUU.GUAA.GCCA..GUGAC.................................CGCUU...............................................GUCAC...
Vvu-1-1     .................................GAUGGAAUGAUAAGCAGC.UUAA.GCCA..GUGACA................................UGA................................................UGUCAC...
Vvu-2-1     .................................GAUGGAAUGAUAAGCAGC.UUAA.GCCA..GUGACA................................UGA................................................UGUCAC...
Vsp-3-1     ................................UGCUGUACCGAUACGCAUC.AAGA.GCCA..GUGACA................................UGU................................................UGUCAC...
Vsp-2-1     ................................UGCUGUACCGAUACGCAUC.AAGA.GCCA..GUGACA................................UGU................................................UGUCAC...
Vpa-1-1     ........................................GGAUAAGCAUU.UUGA.GCCA..GUGACA................................UAC................................................UGUCAC...
Vsp-1-1     ........................................GGAUAAGCGUU.UUGA.GCCA..GUGGCG................................UAC................................................UGUCAC...
Val-1-1     ........................................GGAUAAGCGUU.UUGA.GCCA..GUGGCA................................UAC................................................UGUCAC...
Vfi-1-1     ................................GAAGCACUCAAUAUGUAGC.GUGA.GCCA..GUGAC.................................AAUUCG..............................................GUCAC...
Psp-1-1     ............................ACGUUCAAAAGUUGAUAGGUGAU.UAAU.GCCA..GUGACG................................AUU................................................UGUCAC...
Van-1-1     ............................ACGUUCAAAAGUUGAUAGGUGAU.UAAU.GCCA..GUGACG................................AUU................................................UGUCAC...
Ppr-3-1     ...............................AAAUUCAGAAUAUAUGUGUU.CAGA.GCCA..GUGACA................................UAC................................................UGUCAC...
Ppr-2-1     ................................AAAUCAGAAUAUAUGUGUU.CAGA.GCCA..GUGACA................................UAC................................................UGUCAC...
Dre-1-2     .........................................GCUAUGAUCU.CCCG.GCC...................................................................................................GU
Swo-1-1     ......................................AAAGCUAUGACUG.AAAA.GCC....................................................................................................G
Pat-1-1     .........................................GACAAGGUAU.GUUG.GCC...GCGAAGUG...CGUUC......................GCAA.....................................GAAUGUGUCA.UUCGC...
Pat-1-2     .........................................GACAAGGUAU.GUUG.GCC...GCGAAGUG...CGUUC......................GCAA.....................................GAAUGUGUCA.UUCGC...
Swo-1-2     ........................................UGCCAUGGUGG.CUUA.GCC...................................................................................................GU
env-6       ........................................CGCGAAGGCAG.GAUG.AAC...................................................................................................GA
env-7       ........................................CGCGAAGGCAG.GAUG.AAC...................................................................................................GA
Swo-1-3     .........................................GCUAUGGC.G.GUUG.AGC...................................................................................................GA
Swo-1-4     .........................................GCUAUGGC.G.GUUG.AGC...................................................................................................GA
Chy-1-1     ........................................GGCCAUGGUGA.AAAU.ACC....................................................................................................A
Swo-1-5     .......................................AAGCCACGGAUA.AUUG.GACGAAAGGAGC................................AUCAAAC............................................GCUCCC...
Mth-1-3     ........................................GCCUAUGGAAC.ACCG.GCC...UGGGU.................................UUCGU...............................................ACCCA...
Swo-1-6     .........................................GCCAUGGU.A.UCAA.GUC....................................................................................................A
Csa-1-1     ...................................UCCCUCUUCAUGGUGC.UUUG.GCC...................................................................................................GA
Dac-1-1     ........................................GAUCAUGGUGG.UCCG.GCC...................................................................................................GC
Dac-1-2     .........................................CCCAAGGU.G.GCAG.GCC....................................................................................................U
Hne-1-1     .....................................CGGUGUCAUGGCGG.CCCG.GCC....................................................................................................G
Swo-1-7     .......................................GGCUAAUGGU.A.UACA.GUC....................................................................................................G
Dac-1-3     .......................................CCUUUAAGGG.G.ACAG.GCC....................................................................................................U
Gsp-1-1     ........................................CCCCACGGCAU.GGAG.GCC...................................................................................................UG
Gsp-1-2     ........................................UCCCAUGGC.G.ACCG.GCU.....................................................................................................
Gsp-1-3     .................................UGUCA.UUCCCAUGGCAA..CCG.ACC....................................................................................................A
Pca-1-2     .......................................UCUUCAGGGC.G.UCUG.GCC....................................................................................................A
Gsp-1-4     .....................................AUUCCCCAUGGU.G.UCAG.GCC....................................................................................................U
Ppr-1-1     ........................................CCCCAUGGC.G.GCAG.GCC....................................................................................................A
Gur-1-1     ........................................CCCCAAGGA.A.AGAG.GCC....................................................................................................A
Gur-1-2     .......................................CUCUCACGGU.G.CGCG.GCC....................................................................................................A
Gsu-1-1     .......................................UUCCCACGGC.G.GCCG.GCC....................................................................................................G
Gme-1-1     .......................................GCCUCACGGUA..UGCG.GUC....................................................................................................A
Gur-1-3     .......................................ACCUGAUGGCGC..ACG.GCC....................................................................................................U
Pca-1-3     .......................................UCCUUAUGGC.G.ACAG.GCC....................................................................................................G
Gme-1-2     ........................................CCUCACGGC.C.CAGG.GCC....................................................................................................A
Pca-1-4     .....................................AUCUCUCAGGGGCG.UCGG.GCC....................................................................................................G
Ppr-1-2     ........................................CCUCAUGGU.G.CACG.GCC....................................................................................................A
Pca-1-5     ........................................CCUCAUGGCUG.ACGG.GCC...................................................................................................GG
Gur-1-4     ........................................CCUCAUGGC.G.CCCG.GCC....................................................................................................A
Gsp-1-5     ........................................CCUCAUGGU.G.CCCG.GCC....................................................................................................U
Gme-1-3     .......................................UCCCCAUGGCGG.UCCG.GCC....................................................................................................U
Gme-1-4     .......................................UCCCCAAGGCGG.UCCG.GCC....................................................................................................U
Csp-2-1     .......................................GCGCCAAGGCCG.GGCC.GCC.GCGCCGG.................................UACG................................................CCGGC...
env-8       ..........................................CCAUGGU.A.AAGG.GCC.....................................................................................................
Tet-1-1     .......................................GCCCUAUGAGAU.AAAG.GCC...................................................................................................GA
env-9       ........................................UACUAUGGUUC.GGUG.AGC...................................................................................................GU
env-10      .........................................ACCAUGGUUU.GGCG.AAC...................................................................................................GA
Swo-1-8     .........................................GCUAUGAUUC.ACCAAGCC....................................................................................................U
Swo-1-9     .........................................ACUAUGGUUU.AAAA.GCC....................................................................................................U
Swo-1-10    ........................................AACUAUGGUUC.CCAG.GCC....................................................................................................G

            ..................>>>>>>>>>>>>>>>>>>>>>>>>>>..>>>>>.>>>>.>>....<<<<<<<<<<<<<<<<<<<<<<<<<<<<<<<<<<<<<<...................>>>>>>>>>>>>>>>>>>>>>>>>>>>>>>>>>>>>>>...
            ..................0000??222?2??????????2222?..12???.???2.22....22222?22????????????????2?22??00???122...................221???00??22?2????????????????22?22222...
            ----------------------------------------○●●YA●GR●○●-●●●R-GCC-----------------------------------------------------------------------------------------------------
                                                       -TR
```

```
Swo-1-11   .........................................GCCAUGGUUU.CUAG.GCC...................................................................................................U
Dha-1-6    .........................................UCUACGGU.U.GCCG.GCC...AGGAC.................................GAAA...............................................GUCCU...
Dha-2-5    ........................................UUCUAUGGACA..CCG.GAC...AGGGC.................................CCAG...............................................GCCCU...
Dha-1-7    ........................................UUCUAUGGACA..CCG.GAC...AGGGC.................................CCAG...............................................GCCCU...
Mth-1-4    .........................................UCUAUGGU.G.GCCG.GCC...UGGGCU................................GAGA..............................................GGCCCA...
Dha-2-6    .........................................UCCAUGGUUG.GUCG.GCC...AGGGC.................................UGUAC..............................................GCCCU...
Dha-2-7    .........................................UCCAUGGUUG.GUCG.GCC...AGGGC.................................UGUAC..............................................GCCCU...
Mth-1-5    .........................................CCCAUGGCAG.GUUG.GCC...UGGACC................................GCU...............................................GGUCCA...
Cpe-2-1    ........................................UUCUAUGAUUU.UUAA.GCC...UGGAUUACUAAAUUA.......................UUUAACAU.................................UAAGUUGGUAAUCCA...
Cpe-1-1    ........................................UUCUAUGAUUU.UUAA.GCC...UGGAUUAC.....UAAAUUAUUUAACA..UUAAGUUUAAUAA..............GAACUUAAAUUGUUAAUUGGUUUAAUUGGGGUAAUCCA...
Cpe-3-1    ........................................UUCUAUGAUUU.UUAA.GCC...UGGAUUAC.....UAAAUUAUUUAACA..UUAAGUUUAAUAA..............GAACUUAAAUUGUUAAUUGGUUUAAUUGGGGUAAUCCA...
Dre-1-3    .........................................AUUAUGGUUC.UUAA.GCC..................................................................................................GU
Swo-1-12   ........................................UACUAAGGGUU.UCAG.GCC..................................................................................................UG
Tet-1-2    .........................................GCUAUGGAUU.AUAG.GCC..................................................................................................GA
Swo-1-13   .........................................UCCAGGUU.A.CAGG.GCC...................................................................................................U
Swo-1-14   ........................................GUUCAUGAUUA.UAGG.GCC...................................................................................................U
Swo-1-15   UUUCAUUUUUGCUGGGAU............UGAGUCUUUAAUCCAUGGUUU.AACG.GCC....................................................................................................
Sfu-1-1    ........................................GGCU..UUUGC.GGCG.GCC..................................................................................................GG
           ..................>>>>>>>>>>>>>>>>>>>>>>>>>>..>>>>>.>>>>.>>....<<<<<<<<<<<<<<<<<<<<<<<<<<<<<<<<<<<<<<..................>>>>>>>>>>>>>>>>>>>>>>>>>>>>>>>>>>>>>>...
           ..................0000??222?2??????????2222?..12???.???2.22....22222?22????????????????2?22??00???122..................221???00??22?2????????????????22?22222...
           ----------------------------------------○●●YA●GR●○●-●●●R-GCC----------------------------------------------------------------------------------------------------
                                                      -TR
```

alignment positions 321 · · · 480

```
[1]Mth-1-1 UCAGGGCC.GG...GG.AGCAAUCCCUUGACCUGCUAUUG...................................GG.CAAAGGAA.G.GU.AC.U.CUUUGUAGGGGAGUACCUUUUUCUUUUUAACUGUAAUAAUAAACUGGCAGCCCAUAAGUUAGC
Pca-1-1    AUAGGGGA.CU...UC.GGAAACGAAGUCCCCUUCCGCG....................................UG.UCAUGAUGGG.AA.AA.U.GUCAGAGCUCGGUUCACCUGCGGGAACACCUUUGCAAUGGGAGGGAAAAAUGCGCAACAAAAU
Tth-1-1    CGGGGUGG.CG...GG.GGCAACCCCGCCGCCUGCCGCGGU..................................CG.CAAAGGAG.G.UG.CGCA.UGGGAAGACGUUUGGUGGCCCUUCUUCUCCUCUUCCUCCCCGGCUUCGCCCAGAAGGUGCGGG
Tth-2-1    CGGGGUGG.CG...GG.GGCAACCCCGCCGCCUGCCGCGGU..................................CG.CAAAGGAG.G.UG.CGCA.UGGGAAGACGUUUGGUGGCCCUUCUUCUCCUCUUCCUCCCCGGCUUCGCCCAGAAGGUGCGGG
Sth-1-1    CAGGGUUG.UC...GG.GGAAACCUGGCGGCCUCCCGU.....................................UG.GAAAGGGG.C.GG.AA.C.UGCGGGAGAUGCCCGCGUCGUUCUGCUAUCUUCCGUUCGGGAGAUAGCUUUUUUGCUCCAGGC
Ama-1-1    CC.GGUAG.CC...AG.AGAAAUCAACCUGCCGCCCACG.......AGCUUUAACGCUCGAUAUU..........UG.GAAAGGUG.U.UA.GA.U.GAACUUACAAGACUCAUUCGGAAGAAGAUUCAAUUACCUGAGACUCUCAGUAACUGAGCUGUG
Dha-2-1    CAGGGUCG.GC...AU.AGAAAUGUACCGGCCUCCCGUAAAUGCGGCAUCCUGUUCAGGAUACGGC.........AG.GAAAGAAA.G.GU.GC.A.AAGACCCAGUAUGAUGGGAUUUUAGGCGCAUUUUCGAUUGUGAGCGAAAGUGCGCUUUUGCUG
Dha-1-1    CAGGGUCG.GC...AU.AGAAAUGUACCGGCCUCCCGCAAAGG.GGUAUCCUGUCUGGGAUGCCUC.........AG.GAAAGAAA.G.GU.GC.A.AAGACCCAGAAUGAUGGGAUUUUAGGCGCAUUUUCGAUUGUGAGCGAAAGUGCGCUUUUGCUA
Dha-1-2    CAGGGUCG.GC...AU.AGAAAUGUACCGGCCUCCCGCAA...................................AG.G...GGUA.UCCU.GU.C.UGGGAUGCCUCAGGAAAGAAAGGUGCAAAGACCCAGAAUGAUGGGAUUUUAGGCGCAUUUUCG
Aba-1-1    GAGGGUGA.CG...AA.GGAAACUUCGCCGCCUCCCGUU....................................UG.GAAAGGAA.G.CACCU.C.GGCGCAUGCUGCCGCGGUGUGUUCUGCGCGGCAUGACACUGACCGAUAAAUUCGGACGCACUA
Dha-2-2    CGGGGUCA.GU...CA.GGAAACCAGCUGGCCUUCACUGA...................................UG.AAAAGGAA.G.CA.GA.A.AAUACAGGAUUGACAGAUCGGAUGCUGGCUUUUGCUGUUUUCGUCAUCAGGCGGUUUUGCUUU
Dha-1-3    CGGGGUCA.GU...CA.GGAAACCAGCUGGCCUUCACUGA...................................UG.AAAAGGAA.G.CA.GA.A.AAUACAGGAUUGACAGAUCGGAUGCUGGCUUUUGCUGUUUUCGUCAUCAGGCGGUUUUGCUUU
Dre-1-1    .GGGGUGC.GA...UG.AGCAAUUGUCGUGCCUGCCACUG...................................CG.CAAAGGAA.G.GG.UA.C.UAGUGAUGAACUGGUACCCUUUUUAUAUUGCCUAAAAUAGCAAGAAAGUAUUGAGGGGGUAUU
Dha-2-3    CAGGGUAU.GC...UU.AGAAAUGAACAUGCCUUCCGUAA...................................AG.AAAAGGAGGA.AC.CC.C.UAUGCAGAAGAUUAAAGUACAGGAUGCGAUUGGAUCGGUGCUUAUGCACGAUCUGACACAAAU
Pin-1-1    AAGGGUAA.AG...AG.AGAAAUGUCUUUGCCUCCCGUAUU..................................UG.GAAAGGAA.C.UU.AA.C.AUGAGCCAACAACUUAUUGAUAACUUCGAUCGGAAGUUUGAAUACUUACGCCUGUCGAUAACC
Rsp-1-1    CAGGGUAG.CGA..UU.GGAAACGGCGUUGCCUCCCGUGUU..................................UG.GAAAGGUG.A.AU.AA.U.GUUACAGGAUUCUUUUGGACGCCGGUUCUAUUACCUGCGUCUGUCGGUGACAGAUGUCUGCAA
Cps-1-1    CAGGGUUA.AC...AA.GGAAACGCGUUAGCCUCCCUACAUU.................................UG.GAAAGGUG.ACAU.GU.U.AACAGACAACUUUGGCCGUAGGUUCUCUUACCUGCGUCUCUCUAUUACCGACGUUUGCAAUUU
Hdu-1-1    CAG.GAUA.GU...AA.AGAAAUGCACUAUCCUCUCAUAUU..................................CA.GAAAGGUG.U.CA.AA.U.UUUCAUUCACUUUCAGAGGACUAUCCAGAUGAAAAAAACAGAAAAUAUUAAUACGAGCCGUCG
Apl-1-1    CC.GGAUA.GU...AA.AGAAAUGCACUAUCCCCUCGUGUU..................................UA.GAAAGGUG.U.CA.AA.U.UCAACUUAACUUUCAGAGGGUUAUGCAUAUGAAAACUCAAGAAAAAAUUAAUGCAAGUCGUCG
[2]Hdu-1-2 AAAGGAU..UU...UG.AGAAAUCAAACCUCCUCUCGUAUU..................................UA.GAAAGGUG.U.CC.UA.U.GCAAGCCAUCCCAAUUAAAACUAUUGGCGAUCCAUCCUUAUGCGAUCUUGUUGAUCGAUUCCA
Mha-1-1    AAAGGAUA.AC...UU.AGAAAUGGGUUGUCCUCUCGUGUU..................................UA.GAAAGGUG.U.CA.AA.U.GCAGUCUAUUAUCCCAAUUAAAAACGUGGGUGAUGAUGUCGUUUCAGGUUUAGUUGAUCGCUU
Apl-1-2    AAGGGAAU.UU...CA.AGAAAUUGUUGUUCCUCUCGUGUU..................................UA.GAAAGGUG.U.CC.AA.U.GCAAUCCAUUCCAAUUAAACAUGUUGGGGCGGAUAAUGUUUCUCAGCUUAUUGAUCGCUUUGA
Msu-1-1    CAGGGAUAAUG...UG.GGAAACGACUUAUCCUCUCAUCUU..................................CA.GAAAGGUA.U.CA.AA.U.GCAAUCUAUUCCUAUCAAAAACGUAGGAACCAAUCGAUUAGUCGAUACGUUCCAACGCGAAUA
Hso-1-1    CAGGGAUAAUA...UU.GGAAACGACUUAUCCUUUCUUAUU..................................UA.AAAAGGUG.UCUA.AU.G.CAAAGAUCUAUUUCUAUUCAUAAUGUUAUUGAAAAUCAUCUUAUUGAUGCGUUUCAACGUAAA
Hso-2-1    CAGGGAUAAUA...UU.GGAAACGACUUAUCCUUUCUUAUU..................................UA.AAAAGGUG.UCUA.AU.G.CAAAGAUCUAUUUCUAUUCAUAAUGUUAUUGAAAAUCAUCUUAUUGAUGCGUUUCAACGUAAA
Hin-3-1    CAGGGAUA.CG...CU.GGAAACGGUUUAUCCUCUCCAUAUU.................................UA.GAAAGGUG.U.AA.AA.U.GCAAUCCAUUCCUAUUAAAAACGUAGGAGAGUCUCGCUUAGUCGAUCCUUUCCAACGUCAAUA
Hin-4-1    CAGGGAUA.CG...CU.GGAAACGGUUUAUCCUCUCCGUAUU.................................UA.GAAAGGUG.U.AA.AA.U.GCAAUCCAUUCCUAUUAAAAACGUAGGAGAGUCUCGCUUAGUCGAUCCUUUCCAACGUCAAUA
Hin-2-1    CAGGGAUA.CG...CU.GGAAACGGUUUAUCCUCUCCAUAUU.................................UA.GAAAGGUG.U.AA.AA.U.GCAAUCCAUUCCUAUCAAGAACGUAGGAGAGUCUCGUUUAGUCGAUCCUUUCCAACGCCAAUA
Hin-1-1    CAGGGAUA.CG...CU.GGAAACGGUUUAUCCUCUCCAUAUU.................................UA.GAAAGGUG.U.AA.AA.U.GCAAUCCAUUCCUAUCAAGAACGUAGGAGAGUCUCGCUUAGUCGAUCCUUUCCAACGUCAAUA
Pmu-1-1    CAGGGAUA.CG...CU.GGAAACGGUUUAUCCUCUCGUAUUU.................................UA.GAAAGGUG.U.GA.CA.U.GCAAACAAUUCCAAUUAAACAUGUUGGUAAUGAUCCUUUAGUGGAUGCGUUCCAACGUCGCUA
Asu-1-1    CAGGGAUC.CG...UU.GGAAACAGUUGGUCCUACCGAUUUU.................................UG.UAAAGGUA.U.UC.AA.U.GCAAUCUAUUCCUGUGAAAAAUGCCGGCGAUCGUCUUGUGGAUGCGUACCGGCGUACCUAUUA
Hso-2-2    CAUGGUAU.AA...CU.AGAAAUGGUUGUGCCACUCCCUU...................................UA.GAAAGGUG.U.CG.AA.A.AUAAAACUUUCACAAUGUGUUGGAGAAAGUGCGGUGUGUUUUCUAACCGUUUCGAUGUCUUUU
Hso-1-2    CAUGGUAC.AA...CU.AGAAAUGGUUGUGCCACUCCCUU...................................UA.GAAAGGUG.U.CG.AA.A.AUAAAACUUUCACAAUGUGUUGGAGAAAGUGCGGUGUGUUUUCUAACCGUUUCGAUGUCUUUU
Mha-1-2    AAUGGAUA.CA...CG.AGAAAUGGUUUAUCCACUCGACUU..................................UA.GAAAGGUG.U.CU.AU.U.UUUAACUUAACUUCAAACGAGGUUUAUUAUGAAAAAACAAGACAAUAUUGACGUAAGUCGUCG
Dha-2-4    CGG.GUAU.UA...UU.GGAAACAGUAAUGCCUCCCAUU....................................UG.GAAAGGAGUC.UG.UA.G.GGUAUGCGGGAGCUCCGCGGUUUUACAGACCGUGGGGCUUAUUUAUUUUGUGGAAUAAUGAAC

           ..<<<<<<.<<...<<.<....>>>>>>>>>>><<.........................................>.>...>>>>.>.>>.>>.>.>>>>...........................................................
           ..202222.??...??.2....2????22220222.........................................2.2...12?2.?.??.??.?.????...........................................................
           ●●GGGU●●-●●---●●-RGAAAYR●●●●●GCCUCCC●●●○○----------------------------------UG-GAAAGG●G-●-●●-●●-●-●●●●●●●●●●●●●●●●●●●●●●●●●●●●●●●●●●●●●●●●●●●●●●●●●●●●●●●●●●●●●●●
                            GNRA                                                           SD+++
```

```
Dha-1-5    CGG.GUAU.UA...UU.GGAAACAGUAAUGCCUCCCAUU...................................UG.GAAAGGAGUC.UG.UA.G.GGUAUGCGGGAGCUCCGCGGUUUUACAGACCGUGGGGCUUAUUUAUUUUGUGGAAUAAUGAAC
Csp-1-1    UAGGGUAU.UG...UG.GGAAACUUCAAUGCCUCCCAUUG..................................UG.GAAAGGAG.U.AC.UA.U.GCAGAUCCCUAUUUCUAUCACUCUAUUAACUUGCGCCUUUUUUAGGCGCUUUUUAUUUUUCUG
Dps-1-1    UAGGGUUG.UA...CU.GGAAACGGUUCAGCCUUCCACCACU................................UG.AAAAGGAG.UCUG.UA.U.GAAUGUCUCACCGGGGCCCAGUUCCCCGCAGUCUAAGUUGGUUGAUACGCAUGGCCGUUCAAU
Mth-1-2    CGGGGUAG.GC...GG.GGAAACCGGCCUGCCUCCCGGAAU.................................GG.GAAAGGAG.U.AU.UU.G.ACCCGGUGGUCAACCGGGCCGGAUAGCUUCAGCACCCUUUCCGGGUGCCUUUAAUUUUUUGCG
Ame-1-1    CUGGGUAA.AU...GA.AGAAAUUCUCUUGCCUCCCGAACU.................................UG.AAAAGGAA.A.UU.UG.C.UUUACAUAAGAAUUCUAACUAACGAAUUCAUAUUUGUGUUAAAGCUAAUCCUUUUGGAUUAGC
Ame-1-2    AUAGGUAC.CU...AG.AGAAAUUUUUGUGCCUCCCAACAU.................................UG.GAAAGGAA.A.UA.UA.C.UAGCUAUUUAAGCUAGUCCUUUCGAGGACUAGUUUUUGUUUUAUAUAUUCAUAUUUAUCCGAA
Ahy-1-1    AAGGGUCA.GG...AA.AGAAAUGCCUGGCCUCCCGUAUU..................................UG.GAAAGGUG.U.UU.AU.G.UCGCCACUGCAAGAUGGUUUUUCCCGUCGUUUCUACUAUUUGCGCCUGUCGAUCACCGACGUG
env-1      AAGGGUCA.GG...AA.AGAAAUGCCUGGCCUCCCGUAUU..................................UG.GAAAGGUG.U.UU.AU.G.UCGCCACUGCAAGAUGGUUUUUCCCGUCGUUUCUACUAUUUGCGCCUGUCGAUCACCGACGUG
Plu-1-1    CAGGAUGUGAG...GC.AGAAAUGCCCACGUCUCCCAUGUU.................................UG.GAAAGGUG.U.UA.UG.G.UGAAACAACUCACAGAUGCUUUUGCCCGCAAAUUUUACUAUUUGCGGCUAUCAAUCACCGAUG
Sgl-1-1    CAGGGUGCAGG...GU.AGAAAUGCCGGCAUCUCCCGUAUU.................................UG.GAAAGGUG.U.UACUG.U.GUCACAACUCAUCGAUGCGUUUGAGCGCAGGUUUUAUUAUCUGCGCUUGUCAGUGACGGACGU
Ymo-1-1    CAGGGUGCGAG...GU.AGAAAUGCCUGCAUCUCCCGUAUU.................................UG.GAAAGGUG.U.UA.UG.G.UACAACUGACUGACGCAUUUGCGCGCAAGUUCUAUUACUUGCGCCUAUCGAUCACCGAUGUGU
Yps-2-1    CAGGGUGCAAG...GU.AGAAAUGCCUGCAUCUCCCGUAUU.................................UG.GAAAGGUG.U.UA.UG.G.UACAACUCACUGACGCAUUUGCGCGUAAAUUCUACUAUUUGCGCCUAUCGAUCACUGAUGUGU
Yps-1-1    CAGGGUGCAAG...GU.AGAAAUGCCUGCAUCUCCCGUAUU.................................UG.GAAAGGUG.U.UA.UG.G.UACAACUCACUGACGCAUUUGCGCGUAAAUUCUACUAUUUGCGCCUAUCGAUCACUGAUGUGU
Yfr-1-1    CAGGGUGCGAG...GU.AGAAAUGCCUGCAUCUCCCGUAUU.................................UG.GAAAGGUG.U.UA.UG.G.UACAACUGACUGAUGCGUUUGCGCGCAAGUUCUAUUAUUUGCGCCUAUCAAUUACCGAUGUGU
Yin-1-1    CAGGGUGCGAG...GU.AGAAAUGCCUGCAUCUCCCGUAUU.................................UG.GAAAGGUG.U.UA.UG.G.UACAACUCACUGACGCAUUUGCGCGCAAGUUCUAUUAUUUGCGCCUAUCAAUUACCGAUGUGU
Ybe-1-1    CAGGGUGCGAG...GU.AGAAAUGCCUGCAUCUCCCGUAUU.................................UG.GAAAGGUG.U.UA.UG.G.UACAACUUACUGACGCAUUUGCGCGCAAGUUCUAUUAUUUGCGCCUAUCGAUCACCGACGUGU
Spr-1-1    CAGGGUGCAGA...GC.GGAAACGCUCGCAUCUCCCGACUU.................................UG.G.AAGGUG.U.UA.UG.G.UGCCGCAACUCAUUGAUGCUUAUGAGCGCAAAUUCUAUUACUUGCGCCUGUCGAUCACCGACG
Eca-1-1    CAGGGUGCGAG...GU.AGAAAUGCCUGCAUCUCCCGUAUU.................................UG.GAAAGGUG.U.UU.AU.GGUGAGUCAACUGACUGAUGCGUUUGCGCGCAAGUUUUACUAUUUGCGUCUGUCUAUCACAGACG
Esp-1-1    CAGGGUAC.AG...AA.AGAAAUGACUGUGCCUCCCGUAUC.................................UG.GAAAGGUG.U.AC.AU.G.GCUUCACAACUCACUGAUGCUUUCGCGCGUAAGUUUUUCUAUUUACGUCUGUCGAUUACCGAU
env-2      CAGGGCGC.AG...GA.AGAAAUGACUACGUCUCCCGUAUU.................................UG.GAAAGGUG.U.AC.AU.G.GGCUCUCAACUCACCGAUGCUUUCGCACGUAAGUUUUACUACUUACGUUUGUCGAUUACCGAU
Eco-17-1   CAGGGCGA.AG...GA.AGAAAUGACUUCGCCUCCCGUAUC.................................UG.GAAAGGUG.U.AC.AU.G.GCUUCACAACUGACUGAUGCAUUUGCGCGUAAGUUUUACUACUUGCGCCUGUCGAUUACCGAU
Eco-6-1    CAGGGCGA.AG...GA.AGAAAUGACUUCGCCUCCCGUAUC.................................UG.GAAAGGUG.U.AC.AU.G.GCUUCACAACUGACUGAUGCAUUUGCGCGUAAGUUUUACUACUUGCGCCUGUCGAUUACCGAU
Eco-3-1    CAGGGCGA.AG...GA.AGAAAUGACUUCGCCUCCCGUAUC.................................UG.GAAAGGUG.U.AC.AU.G.GCUUCACAACUGACUGAUGCAUUUGCGCGUAAGUUUUACUACUUGCGCCUGUCGAUUACCGAU
Eco-1-1    CAGGGCGA.AG...GA.AGAAAUGACUUCGCCUCCCGUAUC.................................UG.GAAAGGUG.U.AC.AU.G.GCUUCACAACUGACUGAUGCAUUUGCGCGUAAGUUUUACUACUUGCGCCUGUCGAUUACCGAU
Sbo-2-1    CAGGGCGA.AG...GA.AGAAAUGACUUCGCCUCCCGUAUC.................................UG.GAAAGGUG.U.AC.AU.G.GCUUCACAACUGACUGAUGCAUUUGCGCGUAAGUUUUACUACUUGCGCCUGUCGAUUACCGAU
Sso-1-1    CAGGGCGA.AG...GA.AGAAAUGACUUCGCCUCCCGUAUC.................................UG.GAAAGGUG.U.AC.AU.G.GCUUCACAACUGACUGAUGCAUUUGCGCGUAAGUUUUACUACUUGCGCCUGUCGAUUACCGAU
Sfl-3-1    CAGGGCGA.AG...GAAAGAAAUGACUUCGCCUCCCGUAUC.................................UG.GAAAGGUG.U.AC.AU.G.GCUUCACAACUGACUGAUGCAUUUGCGCGUAAGUUUUACUACUUGCGCCUGUCGAUUACCGAU
Eco-2-1    CAGGGCGA.AG...GA.AGAAAUGACUUCGCCUCCCGUAUC.................................UG.GAAAGGUG.U.AC.AU.G.GCUUCACAACUGACUGAUGCAUUUGCGCGUAAGUUUUACUACUUGCGCCUGUCGAUUACUGAU
Sfl-2-1    CAGGGCGA.AG...GA.AGAAAUGACUUCGCCUCCCGUAUC.................................UG.GAAAGGUG.U.AC.AU.G.GCUUCACAACUGACUGAUGUAUUUGCGCGUAAGUUUUACUACUUGCGCCUGUCGAUUACCGAU
Sdy-1-1    CAGGGCGA.AG...GA.AGAAAUGACUUCGCCUCCCGUAUC.................................UG.GAAAGGUG.U.AC.AU.G.GCUUCACAACUGACUGAUGCAUUUGCGCGUAAGUUUUACUACUUGCGCCUGUCGAUUACCGAU
Sbo-1-1    CAGGGCGA.AG...GA.AGAAAUGACUUCGCCUCCCGUAUC.................................UG.GAAAGGUG.U.AC.AU.G.GCUUCACAACUGACUGAUGCAUUUGCGCGUAAGUUUUACUACUUGCGCCUGUCGAUUACCGAU
Eco-15-1   CAGGGCGA.AG...GA.AGAAAUGACUUCGCCUCCCGUAUC.................................UG.GAAAGGUG.U.AC.AU.G.GCUUCACAACUGACUGAUGCAUUUGCGCGUAAGUUUUACUACUUGCGCCUGUCGAUUACCGAU
Eco-14-1   CAGGGCGA.AG...GA.AGAAAUGACUUCGCCUCCCGUAUC.................................UG.GAAAGGUG.U.AC.AU.G.GCUUCACAACUGACUGAUGCAUUUGCGCGUAAGUUUUACUACUUGCGCCUGUCGAUUACCGAU
Sty-1-1    CAGGGCGC.AG...GA.AGAAAUGACUCCGCCUCCCGUAUU.................................UG.GAAAGGUG.U.AC.AU.G.GCUUCACAACUUACCGAUGCUUUCGCGCGUAAGUUUUACUACUUGCGUUUGUCGAUUACCGAU
Sen-1-1    CAGGGCGC.AG...GA.AGAAAUGACUCCGCCUCCCGUAUU.................................UG.GAAAGGUG.U.AC.AU.G.GCUUCACAACUUACCGAUGCUUUCGCGCGUAAGUUUUACUACUUGCGUUUGUCGAUUACCGAU
Sen-4-1    CAGGGCGC.AG...GA.AGAAAUGACUCCGCCUCCCGUAUU.................................UG.GAAAGGUG.U.AC.AU.G.GCUUCACAACUUACCGAUGCUUUCGCGCGUAAGUUUUACUACUUGCGUUUGUCGAUUACCGAU
Sen-2-1    CAGGGCGC.AG...GA.AGAAAUGACUCCGCCUCCCGUAUU.................................UG.GAAAGGUG.U.AC.AU.G.GCUUCACAACUUACCGAUGCUUUCGCGCGUAAGUUUUACUACUUGCGUUUGUCGAUUACCGAU
Sam-1-1    CGGGGUGG.GG...UGCAGAAAUGGCUUCACCUCCCGAUACU................................UG.GAAAGGUG.A.UC.AU.G.UCUCAACUUCAAGACAGCUUUGGUCGUAAGUUUCACUAUUUGCGGAUGUCGGUUACCGACGUU
Spe-1-1    CGGGGUGA.GA...AA.AGAAAUGAUCUCACCUCCCCUUACU................................UG.GAAAGGUG.A.UC.AU.G.UCCCAACUACAAGACAAUUUUGGUCGACGGUUUCAUUAUUUGCGUAUGUCAGUGACUGACGUA
Ssp-4-1    CGGGGUGA.GG...AA.AGAAAUUGACUCACCUCCCCUUACU................................UG.GAAAGGUG.A.UC.AU.G.UCUCAACUACAAGACAAUUUUGGUCGAAGAUUUCACUAUUUACGGAUGUCGGUAACCGAUGUC
Swo-2-1    CGGGGUGU.GA...UA.AGAAAUUCUCUCACCUCCCCUUACU................................UG.GAAAGGUG.A.UC.AU.G.UCUCAACUACAAGACAAUUUUGGUCGAAGGUUUCACUAUCUGCGCAUGUCAGUCACUGAUGUG
Ssp-5-1    CGGGGUGA.GU...AA.AGAAAUGCUCUCACCUCCCACACU.................................UG.GAAAGGUG.A.UC.AU.G.UCUCAAUUACAAGACAGCUUUGGUCGAAAAUUUCACUAUUUACGGAUGUCUGUUACAGACGUU
Ssp-5-2    CGGGGUGA.GU...AA.AGAAAUGCUCUCACCUCCCACACU.................................UG.GAAAGGUG.A.UC.AU.G.UCUCAAUUACAAGACAGCUUUGGUCGAAAAUUUCACUAUUUACGGAUGUCUGUUACAGACGUU
Spu-1-1    CGGGGUGA.GU...AA.AGAAAUGCUCUCACCUCCCACACU.................................UG.GAAAGGUG.A.UC.AU.G.UCUCAAUUACAAGACAGCUUUGGUCGAAAAUUUCACUAUUUACGGAUGUCUGUUACAGACGUU
Sba-1-1    CGGGGUGG.GA...UA.AGAAAUGCUCUCACCUCCCACACU.................................UG.GAAAGGUG.A.UC.AU.G.UCUCAAUUACAAGACAGCUUUGGUCGAAAAUUUCACUAUUUGCGGAUGUCAGUCACUGACGUU
Sba-2-1    CGGGGUGG.GA...UA.AGAAAUGCUCUCACCUCCCACACU.................................UG.GAAAGGUG.A.UC.AU.G.UCUCAAUUACAAGACAGCUUUGGUCGAAAAUUUCACUAUUUGCGGAUGUCAGUCACUGACGUU
Ssp-1-1    UGGGGUGA.GU...AA.AGAAAUGCACUCGCCUCCCACACU.................................UG.GAAAGGUG.A.UC.AU.G.UCUCAAUUGCAAGACAAUUUUGGUCGUAAAUUUCAUUACUUACGAAUGUCAGUCACUGACGUU
env-4      UGGGGUGA.GU...AA.AGAAAUGCACUCGCCUCCCACACU.................................UG.GAAAGGUG.A.UC.AU.G.UCUCAAUUGCAAGACAAUUUUGGUCGUAAAUUUCAUUACUUACGAAUGUCAGUCACUGACGUU
Ssp-2-1    UGGGGUGA.GU...AA.AGAAAUGCACUCGCCUCCCACACU.................................UG.GAAAGGUG.A.UC.AU.G.UCUCAAUUACAAGACAAUUUUGGUCGUAAAUUUCAUUACUUACGAAUGUCAGUCACUGACGUU
Ssp-3-1    UGGGGUGA.GU...AA.AGAAAUGCACUCGCCUCCCACACU.................................UG.GAAAGGUG.A.UC.AU.G.UCUCAAUUACAAGACAAUUUUGGUCGUAAAUUUCAUUACUUACGAAUGUCAGUCACUGACGUU
Son-1-1    UGGGGUGA.GU...AA.AGAAAUGCUCUCGCCUCCCACACU.................................UG.GAAAGGUG.A.UC.AU.G.UCUCAAUUACAAGACAAUUUUGGUCGUAAAUUUCAUUACUUACGGAUGUCAGUCACUGACGUU
env-5      UGGGGUGA.GU...AA.AGAAAUGCACUCGCCUCCCACACU.................................UG.GAAAGGUG.A.UC.AU.G.UCUCAAUUACAAGACAAUUUUGGUCGUAAAUUUCAUUACUUACGAAUGUCAGUCACUGACGUU
Sfr-1-1    CGGGGUGA.GU...AA.AGAAAUGCACUCACCUCCCACACU.................................UG.GAAAGGUU.A.AC.AU.G.UCCCAACUACAAGACAGCUUUGGUCGAAGAUUUCACUAUUUGCGGAUGUCAGUUACUGAUGUU
Sde-1-1    CGGGGUGA.GU...AA.AGAAAUGCACUCACCUCCCGCACU.................................UG.GAAAGGUU.A.AC.AU.G.UCUCAACUACAAGACAAUUUUGGUCGACGGUUUCACUAUUUGCGUCUGUCUAUUACCGAUGCC
Vch-3-1    AAGGGCAG.GA...UU.GGAAAUGAUUUUGCCUCCCGUAUU.................................UG.GAAAGGUG.U.UC.UG.U.GGCGCAACAAUUCGAAGACAGAUUUCAACGUAAAUUCUACUAUUUACGUCUAUCCAUAACCGA
Vch-1-1    AAGGGCAG.AA...UU.GGAAAUGAUUUUGCCUCCCGUAUU.................................UG.GAAAGGUG.U.UC.UG.U.GGCGCAACAAUUCGAAGACAGAUUUCAGCGUAAAUUUUACUAUUUACGUCUAUCCAUAACCGA
Vch-8-1    AAGGGCAG.AA...UU.GGAAAUGAUUUUGCCUCCCGUAUU.................................UG.GAAAGGUG.U.UC.UG.U.GGCGCAACAAUUCGAAGACAGAUUUCAGCGUAAAUUUUACUAUUUACGUCUAUCCAUAACCGA
Vch-10-1   AAGGGCAG.AA...UU.GGAAAUGAUUUUGCCUCCCGUAUU.................................UG.GAAAGGUG.U.UC.UG.U.GGCGCAACAAUUCGAAGACAGAUUUCAGCGUAAAUUUUACUAUUUACGUCUAUCCAUAACCGA
Vch-13-1   AAGGGCAG.AA...UU.GGAAAUGAUUUUGCCUCCCGUAUU.................................UG.GAAAGGUG.U.UC.UG.U.GGCGCAACAAUUCGAAGACAGAUUUCAGCGUAAAUUUUACUAUUUACGUCUAUCCAUAACCGA
Vch-7-1    AAGGGCAG.AA...UU.GGAAAUGAUUUUGCCUCCCGUAUU.................................UG.GAAAGGUG.U.UC.UG.U.GGCGCAACAAUUCGAAGACAGAUUUCAGCGUAAAUUUUACUAUUUACGUCUAUCCAUAACCGA
Vch-12-1   AAGGGCAG.AA...UU.GGAAAUAAUUUUGCCUCCCGUAUU.................................UG.GAAAGGUG.U.UC.UG.U.GGCGCAACAAUUCGAAGACAGAUUUCAGCGUAAAUUUUACUAUUUACGUCUAUCCAUAACCGA

           ..<<<<<<.<<...<<.<....>>>>>>>>>>><<........................................>.>...>>>>.>.>>.>>.>.>>>>..........................................................
           ..202222.??...??.2....2????22220222........................................2.2...12?2.?.??.??.?.????..........................................................
           ••GGGU••-••---••-RGAAAYR•••••GCCUCCC•••◦◦-----------------------------------UG-GAAAGG•G-•-••-••-•-••••••••••••••••••••••••••••••••••••••••••••••••••••••••••••
                                GNRA                                                        SD+++
```

```
Vvu-1-1   AAGGGCGU.AA...UU.GGAAAUGACUACGCCUCCCGUGUU..................................UG.GAAAGGUG.U.UC.CG.U.GGCGCAACAAUUCGAAGAUAGAUUCCACCGCAAAUUCUAUUACUUGCGUUUGUCUGUGACGGA
Vvu-2-1   AAGGGCGU.AA...UU.GGAAAUGACUACGCCUCCCGUGUU..................................UG.GAAAGGUG.U.UC.CG.U.GGCGCAACAAUUCGAAGAUAGAUUCCACCGCAAAUUCUAUUACUUGCGUUUGUCUGUGACGGA
Vsp-3-1   AAGGGCUU.AA...UU.GGAAAUGAUUAUGCCUCCCGUGUU..................................UG.GAAAGGUG.U.UC.CG.U.GGCGCAACAAUUCGAAGAUAGAUUCCAUCGCAAAUUUAUUAUUUGCGCUUGUCGGUUACAGA
Vsp-2-1   AAGGGCUU.AA...UU.GGAAAUGAUUAUGCCUCCCGUGUU..................................UG.GAAAGGUG.U.UC.CG.U.GGCGCAACAAUUCGAAGAUAGAUUCCAUCGCAAGUUUUAUUACUUGCGCUUGUCGGUUACAGA
Vpa-1-1   AAGGGCAU.GA...UU.GGAAAUGGCCAUGCCUCCCGUGUU..................................UG.GAAAGGUG.U.UC.CG.U.GGCGCAACAAUUCGAAGACAAAUUUCAUCGUAAGUUUUAUUACUUACGUCUGUCUGUGACCGA
Vsp-1-1   AAGGGCAU.GA...UU.GGAAAUGGCCAUGCCUCCCGUGUU..................................UG.GAAAGGUG.U.UC.CG.U.GGCGCAACAAUUCGAAGACAAAUUUCAUCGUAAGUUCUAUUACUUGCGUCUGUCUGUGACCGA
Val-1-1   AAGGGCAU.GA...UU.GGAAAUGGCCAUGCCUCCCGUGUU..................................UG.GAAAGGUG.U.UC.CG.U.GGCGCAACAAUUCGAAGACAAAUUUCAUCGUAAGUUUUAUUACUUGCGUCUGUCUGUGACCGA
Vfi-1-1   AAGGGUUU.GU...UU.GGAAAUGAACAAGCCUCCCGUGUU..................................UG.GAAAGGUG.U.UC.CG.U.GGCGCAACAAUUCGCAGAUAAUUUUGAUCGUAAGUUCUAUUACUUACGAUUAUCAGUUACAGA
Psp-1-1   CAGGGUUU.AU...UU.AGAAAUGAAUUGACCUCCCGUAUU..................................UG.GAAAGGUG.U.UC.CG.U.GGCGAAACAAUUAGAAGAUAGUUUUCAUCGUAAGUUUUACUACUUGCGACUCUCUGUUACAGA
Van-1-1   CAGGGUUU.AU...UU.AGAAAUGAAUUGACCUCCCGUAUU..................................UG.GAAAGGUG.U.UC.CG.U.GGCGAAACAAUUAGAAGAUAGUUUCCAUCGUAAGUUUUAUUACUUGCGACUCUCUGUUACAGA
Ppr-3-1   CAGGGUCA.AU...UU.AGAAAUGAAUCGGCCUCCCGUAUU..................................UG.GAAAGGUG.U.UC.CA.U.GGCGCAACAAUUAGAUAAUGAUCUUCAAUUUGAAGAUCGCUUUCAUCGUAAGUUUUAUUACUU
Ppr-2-1   CAGGGUCA.AU...UU.AGAAAUGAAUCGGCCUCCCGUAUU..................................UG.GAAAGGUG.U.UC.CA.U.GGCGCAACAAUUAGAUAAUGAUCUUCAAUUUGAAGAUCGCUUUCAUCGUAAGUUUUAUUACUU
Dre-1-2   UAGGGUUU.AC...UG.AGAAAUUGGUAGGCCUCCCGUG....................................UG.GAAAGGAG.C.CG.GU.A.UAUGCUUGGUCGUGUUUUUUUGGCCGGGGUCUGCCUGCUUCUUGCAGAGAUCCUGGUUUUUAA
Swo-1-1   AUAGGUAG.GA...GG.GGAAACGCUCAUGCCUCCCGUU....................................UG.GAAAGGAG.A.GC.AA.A.AUGUAUGCAAGUUGACUAAUGCCGGGCUUAUCUUUCUGGAUGGGCCUGGUUUUUAUUUGUGUU
Pat-1-1   CAGGGUUG.UG...AG.AGAAAUCAAACAGCCUCCCCGUAAG.................................UG.CCAAGGCG.A.UA.AG.G.CCCGGCAACUUCAAUUUGGAAAGGUGUUAGUGAAUAUGUUGCAAGAUAAAUUUGGUCGCCGGU
Pat-1-2   CAGGGUUG.UG...AG.AGAAAUCAAACAGCCUCCCCGUAAGUGCCAAGGCGAUAAGGCCCGGCAACUUCAAUU.UG.GAAAGGUG.U.UA.GU.G.AAUAUGUUGCAAGAUAAAUUUGGUCGCCGGUUUCAUUACCUGCGGCUGUCCAUUACUGAUGUG
Swo-1-2   UAGGGUAA.AC...AG.GGAAACAGGUUUGCCUCCAUCAUG..................................AG.GAAAGGAG.C.GG.UA.U.UAUAUUUAUAUCACGCUUUUCCAUACUAACCCCCUACGGCCACAACCUUAGGGGAAGCGCGAU
env-6     CAGGGUCA.GC...AG.GGAAACGUGCUGGCCUCCCGUUU...................................UG.GAAAGGAG.A.CC.UC.C.UAUGUUCGACCUUCAAGGUAGAAAUAUCAACAAACUCCGGGUUUCCGUGAGCGAGGCCU....
env-7     UAGGGUUA.GC...AG.GGAAACGUGCUGGCCUCCCGUUU...................................UG.GAAAGGAG.A.CC.UC.C.UAUGUUGGACCUUCAGGGUAGAAAUAUCAAUAAGCUCCGGGUUUCCGUGAGCGAGGCCUGCAA
Swo-1-3   UUGGGUAU.GG...AG.GGAAACCUCUGUGCCUCCCGUU....................................UG.GAAAGGAG.U.AAGAU.U.AAGUGUAGUGCUUAAUAACACCCCUUGUCCGGGGUGAUUUUUUUUGUGGAGGUGUUGAAGGUG
Swo-1-4   UUGGGUAU.GG...AG.GGAAACCUCUGUGCCUCCCGUU....................................UG.GAAAGGAG.U.AAGAU.U.AAGUGUAGUGCUUAAUAACACCCCUUGUCCGGGGUGAUUUUUUUUGUGGAGGUGUUGAAGGUG
Chy-1-1   AAGGGUACUAC...CG.GGAAACCGGGGUGCCUCCCGUUU...................................UG.GAAAGGAG.U.UA.UC.A.AAGCGCCUUUUUAAAACGGGGCGCUUUUAAUUUUAGGAGGCUAAAGAAAUGCUUGAUUCUUUU
Swo-1-5   CAGGGUAU.AG...CU.GUAAACGGCUGUGCCUCCCGUAUU..................................UG.GAAAGAAG.A.UA.UG.G.AACAUCCUUUCUGAGGGUUAGUCAUGUUGGCUAACAAACUCAGGAGAUUAACAUAAGCAGGAA
Mth-1-3   UGGGGUAG.GU...CG.GGAAACGGGCCUGCCUCCCUUUGCU.................................CG.GAAAGGAG.A.UU.CC.C.CAUGGCUUCUGGACAACGGAGUCCCGGUAUCCUUUGCGAGCCGGGCUUUUUUAUUUAUUCCGG
Swo-1-6   UUGGGUAC.AC...CG.GGAAACGGGUGUGCCUCCCACUG...................................UG.GAAAGGAG.A.AC.CU.A.UAUCCAAACGGUUGCUAUUAAGGGGCAAGUUUGUUCUUUCACAGACUUGCCCUAUUUUAAUAG
Csa-1-1   UAGGGUGC.AGGUUUG.GGAAACCACUGCGCCUCCCGCUU...................................UG.GAAAGGAA.A.GA.UU.U.AAAGGAUACUCUUUCCGAGUUUGUUCAAAGCGGGGGAGUAUCCUUUUUUGUUUCAAAAGAGUG
Dac-1-1   CA.GGUGG.UC...CC.GGAAACGGUAUCGCCCCCCAUGUU..................................UG.GAAAGGAG.A.UU.UC.C.GCCAUGAGCGAUGAUUGUUCACCUGCAACAACGGAGAGAACCCAUGGAUAAAAUUUUUCGCGU
Dac-1-2   AUGGGUUC.UU...CG.GGAAACUGGCGAGCCUCCCACUCU..................................UG.GAAAGGAG.C.UG.AC.G.GACAUUAAUCAUCAAGGUGACUGGUGCCGUUUUUUGUCUUUCCCGGCGAUGGAGGGUCAUUUU
Hne-1-1   AGG.GUGG.CA...GUUGGAAACCCUGCCGCCUCCCGUGCU..................................UG.GAAAGGAG.C.CA.UG.C.GAUGACACAUCCCGGCGCAGUCGCGACGGAUGCCUUCGGGCGCAGGUUCAGUUACCUGCGGCU
Swo-1-7   AUAGGUGC.AA...CU.GAAAACGGCUGUGCCUCCCUUU....................................UG.GAAAGGAG.A.GU.AU.U.UAUCCUGGGUACCGGUGAAAUCACCCGGAGUCUUCUUUCGAGAGUUUAGGGUUUUUUUAAUUC
Dac-1-3   CAGGGUUG.CA...UU.GGAAACGGUGUGGCCUCCCAGUCU..................................UG.GAAAGGAG.A.UU.CU.U.ACCAUGCGCUUAACGUUAAGACACUGCAAAGGAGCCUGACACACAUGGAUAAGAUUUUCCGCG
Gsp-1-1   CAGG..CCGGG...CC.GGAAACGGCCGGACCUCCCUUU....................................CG.GAAAGGAG.A.CUGCA.A.AUAAUCACUGAAAGGGAGACUCCCCAUGAACAAGAUCCUUCGCGUCAACAUGACGACUCUCAC
Gsp-1-2   .AGGGUGC.CG...CU.GGAAACGGCUGUGCCUCCCUUU....................................UG.GAAAGGAG.A.GC.CU.C.AAAGUACCGGACCUGUUCCUGUAUCCAAAGGCCUCUUUCCAUCUGCGGACAGACUCUUUGAUG
Gsp-1-3   UUGGGUCC.UG...CU.GGAAACGGCAGCGCCUCCCUUU....................................UG.GAAAGGAG.A.GU.CA.CCGAAC.GGCAUAAAAAACCGUUACCCGCGGGGUCUCUUUCCAUCAAGAAAGGACCUUUUUUA
Pca-1-2   AAGGGUUU.CG...CU.GGAAACGGCGAUGCCUCCCGCGUU..................................UG.GAAAGGAG.A.UA.AU.C.CCGGAGUUCCGUUCCAUUAAUUUCGCAUUGGGAGGAAAACAAGCUAUGCACAUUGUACGUGUC
Gsp-1-4   GUGGGCUC.UG...CU.GGAAACGGCAGGACCUUCCUU.....................................UG.AAAAGGAG.A.AC.CU.C.UCGCCACGCUAUGAUUGCGUUCUAGGGGCUCCUUUAUCCAAAGGAGCCCUUUUUGUUUGUGGC
Ppr-1-1   ACGGGUUC.UG...GC.GGAAACGCCCGAGCCUCCCUUU....................................UG.GAAAGGAG.A.UG.AC.A.GCAGCAGGACAAACCACACCAAAAGGGAGAAAACAAGAUGGACAAGAUUGUACGCGUAAACAU
Gur-1-1   ACGGGUUU.CG...CG.GGAAACUGCGACGCCUCCCUU.....................................UG.GAAAGGAG.A.AU.UA.A.CGUGGCUUGAAGCCACAACAUUCAAAAAAGGGAAAGGAGUAAACGUAUGGAAAAGAUUGCCAG
Gur-1-2   ACGGGUGC.UG...CC.GGAAACGGCUGCGCCUUCCUUU....................................UG.AAAAGGAG.A.AC.AC.C.CUCCGUCAGAACCGUUUCCGCUGCAGGCCUCUCUCCAGCCGGGGAGGGGCCUUUUUCGUUCCG
Gsu-1-1   ACGGGUGU.UG...CU.GGAAACGGCAGCGCCUCCCCUC....................................UG.GAAAGGAG.A.UU.CC.G.GGCCACGUGCGCCCAGAGACCCUUUCCAGCAGGAAGGGGUUUUGUGUUUUCCGCCCCUAUUCA
Gme-1-1   ACGGGUUC.CG...CU.GGAAACGGCGGUGCCUCCCUUU....................................UG.GAAAGGAG.A.AC.UC.U.UUAUUCCCCUGCGUGGGGUAUGGGGCCUCUUUCCGUUUGGAGAGAGGCCCUUUUUGUUGUGCA
Gur-1-3   AUGGGUUC.UG...CC.GGAAACGGCAGCGCCUCCCUUU....................................UG.GAAAGGAGAA.AC.CG.C.AGCGUUGCAAAACGUUCAGGGGCCUCCUUAUUCGGACAAGGAGGCCCCUUUUUCAUUGUUUCG
Pca-1-3   ACGGGUUC.CG...CU.GGAAACAGUGGCGCCUCCCUUUU...................................UG.GAAAGGAG.A.UU.UU.A.CCCGGAACCAUUCCAGAUAGUAAAAGAUUGGGAGGAAGCAAGAGAAUGGAUAAAAUCUUUCGC
Gme-1-2   CCGGGUGC.CG...CC.GGAAACGGCGGCGCCUCCCUUU....................................CG.GAAAGGAG.A.AC.CA.U.CAAUGAGAAACGCUAUCUAUAGAAAGGGAGCAAUCAGUUCAUGGAUAAGAUAAUCCGCGUAAA
Pca-1-4   AUGGGUUU.CG...CC.GGAAACAGCGGAGCCUCCCUGUUUU.................................CG.GAAAGGAG.A.CC.AU.U.GCCUUGGGCUGUUUGAGGUAAUGUAUCCAAAAAGGUUCUUUCCGAUGAAAGGGGGAACCUUU
Ppr-1-2   ACGGGUUU.CG...CU.GGAAACAGCGGAGCCUCCCUUC....................................AG.GAAAGGAGCU.UG.UC.C.UCGUUAGAGAGGGUACAUAAAAUCAUAGGGAGAAGAUAACCAUGGACAAGAUUCUACGCGUUA
Pca-1-5   CAGGGUUU.CG...CU.GGAAACGGCGGAGCCUCCCUUU....................................CG.GAAAGGAG.A.UA.UU.U.GCAUUUUGAGCAACCUCGUCUGAGGAAGGGAGAACAGCACAUGUUAGGUGGUUAUUGGCAAAA
Gur-1-4   ACGGGUUU.CG...CU.GGAAACAGCGGAGCCUCCCUUU....................................CG.GAAAGGAG.A.CC.UA.U.CCGCUCAGCGGAUUUUAAAACAUGAAAGGGAGCAAUGAACAAUGGACAAAAUUUUACGCGUAA
Gsp-1-5   ACGGGUUU.CG...CU.GGAAACGGCGGGGCCUCCCUUU....................................CG.GAAAGGAGUA.AG.CA.A.GCCCUUGCGGGUAAACACUAUGAUUGGGAGAAGAUAAAGAAAUGGACAAGAUUUUACGCGUCA
Gme-1-3   AUGGGUAU.CG...CU.GGAAACGGCGUUGCCUUCCUUA....................................UG.CAAAGGGG.ACCC.AG.C.GACGCGGAGGUAUGUCCGCGACGUUCAGGGGCCUUUCUAUCCAGAGAGCGCCCCUGUUGUUUU
Gme-1-4   AUGGGUAU.CG...CU.GGAAACGGCGUUGCCUUCCUUA....................................UG.CAAAGGGG.ACCC.AA.C.GGUGCGGAGGUGUGUCCGCGACGUUCAGGGGCCUUUCUAUCCAGGAGAGCGCCCCUUUUGUUU
Csp-2-1   AAGGGUGA.CG...GG.GGAAACGCCGUCGCCUCCCUUGCU..................................UG.GAAAGGAG.C.CC.GC.A.UGGGACGUCUGGUUGACGGCUUCGGCCGCCGCUUUCGCUAUCUGCGCCUCUCGGUCACCGAGG
env-8     .UGGGCCA.CC...UU.GGAAACGGGAUGGCCUUCCGUAUUU.................................UG.AAAAGGGG.U.UG.UU.A.UCAUGCGGGCGGGACCCGGCGCGGCAAUUGCAGCACCGGAUUUGUCCAUAUCGCCCUCAUUAU
Tet-1-1   UAGGGUAU.UG...AU.GGAAACAUCAAUGCCUCCCGUGU...................................UG.GAAAGGAG.A.GG.CA.A.UGCAUUUGCAUUUAUGCAAAGCAAGAAUGCGUAAGGUAAAGUAAUUGUGUCCUUGCUUUGCGA
env-9     UAGGGUUG.AA...AG.GGAAACUUCUCAGCCUCCCGGAAU..................................UG.GAAAGGAGAA.AC.CA.C.AUGUUAGACCGCCAAGGUAGAAAUAUAAACAAACUGAGAGUAUCUUUAACUGAGGUUUGCAAU
env-10    UAGGGUGG.AC...AG.GGAAACUGGUCUGCCUCCCGACUU..................................UG.GAAAGGAG.A.CC.UU.UCAUGAUAGACCAGCACGGUCGAGACAUUAAUAAACUCCGUAUAUCUUUAAGUGAAGUCUGUAAU
Swo-1-8   GUAGGUGU.AGA..CU.GGAAACGGAUACGCCUUCCCGUAUUG................................UG.AAAAGGAG.U.UA.GU.A.UCAACUGUUCGAAAUGCAGACAGAUGUUGUGUGCUCUAAUUCACAUACAUCUGUUUUAUUAUU
Swo-1-9   GUGGGUAU.AU...GC.GGAAACGGAUAUGCCUUCCCGUAUUG................................UG.AAAAGGAG.A.UG.CU.C.AGCAGGCAGGUACUUUAUAAGCAUAUCCAUAAAGGGUUCAAUUGCUUAUUAUUGUGCAUCUGU
Swo-1-10  AUGGGUAU.AGA..UU.GGAAACGGCUAUGCCUCCCCGUAUUG................................UG.GAAAGGAG.A.CG.AC.G.AGGGCUUAUUUUAUAUAGGUUUAAAUGGUCGUGUUCUUUUUCCAGGACACGACCUUCUUGUUU
Swo-1-11  GUGGGUAU.AG...UU.GGAAACGAUUGUGCCUCCCCGUAUUU.U..............................UG.GAAAGGAG.CCUA.AA.A.AGCCUGGUUAAGAGCAGGUAGUACUUGCUGGGCCAUAUUGGGCAUAAAAUCCAAAUAUUUUUU
Dha-1-6   CAGGGUGU.GU...CU.GGAAACGGAUAGGCCUUCACGUU...................................UG.AAAAGGAG.G.GA.AG.A.AAAGUGCUUUGCAAUCAAGGUGAAGCAGGAAGAAGAGUAAGAUUGGUGUUUGAUAAUGACGGG

          ..<<<<<<.<<...<<.<....>>>>>>>>>>><<.........................................>.>...>>>>.>.>>.>>.>.>>>>...........................................................
          ..202222.??...??.2....2????22220222.........................................2.2...12?2.?.??.??.?.????...........................................................
          ●●GGGU●●-●●---●●-RGAAAYR●●●●●GCCUCCC●●●●○-----------------------------------UG-GAAAGG●G-●-●●-●●-●-●●●●●●●●●●●●●●●●●●●●●●●●●●●●●●●●●●●●●●●●●●●●●●●●●●●●●●●●●●●●●
                             GNRA                                                       SD+++
```

```
Dha-2-5   CGGGGUAU.AC...UU.GGAAACGGGUAUGCCUUCACGUU...................................UG.AAAAGGAG.G.UG.GA.U.UAAAGCCAACUGUGUAUGAAAUCAGUUAUGGCUCCGCUUUCUAUUGGCGGAGCCAUUAUUUUU
Dha-1-7   CGGGGUAU.AC...UU.GGAAACGGGUAUGCCUUCACGUU...................................UG.AAAAGGAG.G.UG.GA.U.UAAAGCCAACUGUGUAUGAAAUCAGUUAUGGCUCCGCUUUCUAUUGGCGGAGCCAUUAUUUUU
Mth-1-4   CGGGGUAU.AC...CG.GGAAACUGGUAUGCCUUCCCAGGUUU.G..............................AGUAUAAGGAG.G.UG.GC.U.GAUUAGUGGCUGUUUAUGGCUUUUAUUAGCCCCACCUUCCUACAGGUGGGGUUUUAGUUUUAG
Dha-2-6   CGGGGUAU.AU...CC.AGAAAUGGCUAUGCCUUCACGUU...................................UG.AGAAGGAG.G.U..............GGAUUCCCAAUAUGUUGGGUUUACUAGAGGUUCCACUUUCGUUAAAGUGGAACCUU
Dha-2-7   CGGGGUAU.AU...CC.AGAAAUGGCUAUGCCUUCACGUU...................................UG.AGAAGGAG.G.UGGAU.U.CCCAAUAUGUUGGGUUUACUAGAGGUUCCACUUUCGUUAAAGUGGAACCUUUUAACGUUUAAA
Mth-1-5   CGGGGUGU.AC...CG.GGAAACGGGUAUGCCUUCCAGGUAU.................................UG.AAAAGGAG.G.GG.GU.U.AGCUAUAAGAGAGAGCUCUGGCUUUUUUAGUUAAACCCCGCUCCGCCGUGGAGACGGCGUUUU
Cpe-2-1   CAGGGUAA.GG...UU.UGAAAGAAUUUUGCCUCCACGUAUUU................................UG.GGAAGGAG.A.AU.AA.G.CAAAAGUAAAAAACAUAGAACUUACAUAAUAAUAUUGUUAAUUAUGUUUUUUAGUUUAAUAU
Cpe-1-1   CAGGGUAA.GG...UU.UGAAAGAAUUUUGCCUCCACGUAUUU................................UG.GGAAGGAG.A.AU.AA.G.CAAAAGUAAAAAACAUAGAACUUACAUAAUAAUAUUGUUAAUUAUGUUUUUUAGUUUAAUAU
Cpe-3-1   CAGGGUAA.GG...UU.UGAAAGAAUUUUGCCUCCACGUAUUU................................UG.GGAAGGAG.A.AU.AA.G.CAAAAGUAAAAAACAUAGAACUUACAUAAUAAUAUUGUUAAUUAUGUUUUUUAGUUUAAUAU
Dre-1-3   UAGGGUUU.GU...CU.GGAAACGGGCAGGCCUCCCAAUG...................................CG.GAAAGGAGAA.AC.AC.A.UUAUGCCAAAUGGCGAGUCCGUGUACUGUUUUUUCUUUCUUACAGUACACGGUUUUAUUUUUU
Swo-1-12  UAGGGUAC.GG...CG.GGAAACGGCCGGCCUCCCAUGG...................................UG.GAAAGGAG.A.AA.UC.A.AAUGCGCUUACAUAGCAGGUUAAUUCCAUUUCUAAUUAACCCUGUGCUUGUAUAUUUCUGCGC
Tet-1-2   UAGGGUAG.UG...CU.GGAAACGGUGCUGCCUCCCGCC....................................UG.GAAAGGAG.A.AU.AG.G.AAGGCUGCUAUUCUCCUUUCGCAUACUAAAACGAAAGGAGUUUUUUUAUGAAGGUUCUUUGUC
Swo-1-13  AUAGGUAU.AA...GU.GGAAACACUUAUGCCUCCCCGUAUUG................................UG.GAAAGGAG.U.AG.UG.A.AAGAACUGUUCAGUUAAUUGAACAGGUGUUACUGUGCUUUUUCACAACACACAACACCUGUUU
Swo-1-14  AUAGGUAU.AA...GU.GGAAACGCUUAUGCCUCCCCGUAUUG................................UG.GAAAGGAG.U.AC.UU.A.UUUAGCUGUUCGUCAAAAGAACAGGUGUUAAUGUGCUUUCUUUCAUUAACACCUGUUUUUUAU
Swo-1-15  .GCGGUAU.AA...GU.GGAAACGCUUGUGCCUCCCUUAUUU..................................G.GAAAGGAG.A.CC.AA.A.UUCACUAUUAGUUAGGUUUAGCCAUUUUUAGACCCCGGUUAUAUAGGAUAAGCCUGACAGGUG
Sfu-1-1   CAGGGUGU.GC...CU.GGAAACGGGCAUGCCUCCCGUUG...................................CG.GAAAGGGG.A.GC.AG.G.UCAAACCGGGUUUCCAGUGCCGGAGCCUGCCCUUUUCGCGGGUUCCGGUUUUUUUGCCUCGUU
          ..<<<<<<.<<...<<.<....>>>>>>>>>>><<........................................>.>...>>>>.>.>>.>>.>.>>>>...........................................................
          ..202222.??...??.2....2????22220222........................................2.2...12?2.?.??.??.?.????...........................................................
          ●●GGU●●-●●---●●-RGAAAYR●●●●●GCCUCCC●●●○○-----------------------------------UG-GAAAGG●G-●-●●-●●-●-●●●●●●●●●●●●●●●●●●●●●●●●●●●●●●●●●●●●●●●●●●●●●●●●●●●●●●●●●●●●●●
                          GNRA                                                           SD+++
```

alignment positions 481 · · · 640

```
1 Mth-1-1 UUCCCGUUUUUUACCUGAAUUUUUACCUGGAGGUGGUGGGGCAAAGGCGAGGCAGUAAAUAUAAAUGUCGAAUUUAUAAUGAAAAUCGCGAAAGGGGGAAUUAUCUUGCCUAAGGUACUGGUAGUCGAUCAGGGUAAAUGCACCGCCUGCCGGCGCUGCG
Pca-1-1   CUUUCAAACAAUCUGGCUGGUACUCAUCCUGGCGCUGACGGGCAUCCCCUGUCCCGCAUGGAGCGCUCCGGAGCCUUUGACCAUUUAUUCCGGGGCAGGAUUAAUGAAGCCGAUGGACGAGCUGAAAGCAGGCUUCGAAGCCCGCUAUAACAUCCCGGUU
Tth-1-1   UGGUGGCGGCCUCGGACCUCCAGUACGCCCUCCCCGAGGUCGCCCGGGCCUUUGAGCGGAAAAACCCCGGGGUGAAGGUGGAGCUCGUCUUCGGCUCCUCCGGCAAGCUCUACGCCCAGCUCACCCAGGGCCUCGAGGCCGACCUCUUCUUCUCCGCCGA
Tth-2-1   UGGUGGCGGCCUCGGACCUCCAGUACGCCCUCCCCGAGGUCGCCCGGGCCUUUGAGCGGAAAAACCCCGGGGUGAAGGUGGAGCUCGUCUUCGGCUCCUCCGGCAAGCUCUACGCCCAGCUCACCCAGGGCCUCGAGGCCGACCUCUUCUUCUCCGCCGA
Sth-1-1   AGUAUCGGCGAAGCAGGUGAGGACAUGGACUUUUUCACGCUGAAAUCUGUCGAGGAGGCCCGGGUCGAGUUCCUCGGCGCCGUCACCCGCCGGAUGCCCGCGGAGCGGGUCGCAAGGGAGGAAGCCCUGGGGCGUAUCACCGCCGCCCCCGUGCCCGCUC
Ama-1-1   CAAUUUCCGCUGUAAUUACUGUCUUCCUGAUGGCAACGACUGCAACACACCCAAAGGUGGUUUGUCAGUGAAAGAAAUUCAAACCUUGGUGCAAGUGUUUGCCGGCUUGGGUACAACCAAAGUGCGUAUUACUGGUGGCGAACCAGCACUGCGCAAAGAU
Dha-2-1   UUGUUUUUGCUUUUGCUGGCCAAGGGUUCCGUAGAGGCCUUAGACGAGAGAAGGGGGAAAAAGGAUGGCAGACUGUCUUUCCGCAAUGCGUUCGGUGGAAUUUCUGAGUCACUUUUCCGAUGAUGAGCUCCAGGAGAUUGGCAAAUGCCUGCUGACCAGG
Dha-1-1   UUGUUUUUGCUUUUGGUAACCAAGGGUUCCGUAGAGGCCUUAGAGGAGAAGAAGGGGGAAAAAGGAUGGCAGACUGUCUUUCCGCAAUGCGUUCGGUGGAAUUUCUGAGUCAUUUUUCCGAUGAUGAGCUCCAGGAGAUUGGCAAAUGCCUGCUGACCAG
Dha-1-2   AUUGUGAGCGAAAGUGCGCUUUUGCUAUUGUUUUUGCUUUUGGUAACCAAGGGUUCCGUAGAGGCCUUAGAGGAGAAGAAGGGGGAAAAAGGAUGGCAGACUGUCUUUCCGCAAUGCGUUCGGUGGAAUUUCUGAGUCAUUUUUCCGAUGAUGAGCUCCA
Aba-1-1   UCAACGACCUCCGGCUGUCCAUCACGGACCGCUGUAACUAUCGUUGUGUCUAUUGCCGCUCUGGCAACAAUGGGCCAUCCUUCGCCGAACUUCGCUUCCAAGAUUACGUCCGUAUUGCUCGCGUCUUCGUCUCCCUCGGAAUUACUAAGAUCCGCCUCAC
Dha-2-2   AGAAAGGCAGGCCGCCUUUUCUGUUGGUAAUGAGUUCUAUGAUGUUAUUCAUAUUGUGUCAUUUAUAUUGUUUUUAUAAGGAGAAAACCAUGGCAAACAAUCAGCAUGAUCACAUCAGCUGCCCCUCCUCAUCCUGCAUCGGUUGUGCCACACGCUGUCA
Dha-1-3   AGAAAGGCAGGCCGCUUUUUCUGUUGGUAUUGUUUUUAUAAGGAGAAACACCAUGGCAAAUAAUCAGCAUGAUCACAUCAGCUGCCCCUCCUCAUCCUGCAUCGGUUGUGCCACACGCUGUCAUCGGCAGCAGAAUGAUCCGUUCUCCCGGGAUGAGGCU
Dre-1-1   AAAUAUGACAAAACUUUUUCGUGUGAACAUGACAACCAAAGAAGCCAAGAUUGAAGAGCUUCCCCAGAAGUAUGUUGGGCUCGCUGGCCGUGCCUUGACCUCAAGCUUUGUACUUGAUGAGGUAGAACCUAAAUGCCAUCCCCUGGGACCCAACAACAAA
Dha-2-3   UAUUCCAGGAAAAACAAAAGACGCCCGCUUUAGAAAGGGUCAUGUGAUCAAAGAAGAGGAUAUUCCCACCCUGCUUAGUAUGGGAAAAGAGCAUAUUUUUGUCUGGGAUCAGACUCCGGGUCUUAUUCACGAAGAUGAAGCGGCGGAGCGCCUGGCCAAG
Pin-1-1   GAUGUCUGUAAUUUUAAAUGUAAUUAUUGUCUGCCCGAUGGAUAUCAGCGAACCCAUGAAAAACAGUUUUUGAGCAAGCAGGAGAUAACCAACUUAGUCAAUGCUUUCGCCGAACUGGGUGCUAAAAAAGUACGUAUAACAGGUGGAGAACCCGCUUUAC
Rsp-1-1   UUUUCGUUGUAGCUACUGUCUUCCCGAUGGCUAUCAGGGGAAACCCGAUGAAGCCUUUCUUUCGGCGUCCGAGCUGGAAGCUGCUGUGCGCGGUUUUGCGCAAAUGGGCACGCAAAAAAUUCGCCUGACCGGCGGUGAACCCGGUUUACGUUCGGACCUG
Cps-1-1   UAGUUGCACUUACUGCUUGCCGGAUGGAUAUCAGUGCGAUCAACCGCGUGAUUUUUUAUCACUAUGUGAAAUCAAACGUAUAGCGAAAGCUUUCGCCGAGUUAGGAACUGAAAAGAUCCGCAUAACCGGUGGUGAACCAGCCCUACGUAAAGACUUACCU
Hdu-1-1   AGGUUUUAUUAAAAAUACUUCGUUUGGUCUUGCAGGGACAUCUUUAGCCGGUGGUAGCGUAAAUGCAUUGCUUACAACAGAAGCCGUUGCCGCAGAAAUGCAAACUGUCGUUACUGCCGCACAUUGGGGCGCAUUAGGUGUCGUAGUUGAAAACGGUAAA
Apl-1-1   UGGUUUUAUUAAAAAUACCUCUCUCGGUCUUGCUGGGACAUCUUUAGCGGGCGGUAGUGUAAAUGCCUUAAUUUCAACGGAAGCGGCCGCAGCGGAGAUGCAAACCGUAAUGACAGCAGCACACUGGGGGGCGUUAGGUAUCGUGGUUGAAAACGGUAAA
2 Hdu-1-2 GCGUCAAUACACUUAUCUGAGAUUAUCUAUUACCGAGGUAUGCAAUUUCCGUUGUAAUUAUUGCUUGCCAGAUGGCUACAGACCACCAUCUCAUAAACAACGUUUUCUUAAUUUAGUAGAAAUUAAACGGCUUGCACGCACUUUUGCCAAUCUUGGUACA
Mha-1-1   UCAGCGUCAAUAUACUUAUUUGCGACUAUCCAUUACUGAUGUUUGUAACUUCCGUUGUAAUUAUUGCUUGCCUGAUGGCUAUAAGCCGCCUUCACAUAAGCAAACUUUUCUUAGCGUUGAUGAAAUCCAGCGGGUGGCUAAAGCCUUUGCCAAUUUAGGG
Apl-1-2   ACGACAAUAUGUUUAUUUACGUUUAUCCGUUACCGAUGUCUGCAAUUUUCGCUGUAAUUAUUGUCUGCCGGACGGCUAUAAGCCUCCUUCACAUAGGCAGCAAUUUUUAAGCAUAUCCGAAAUUCAACGUGUCGUGCGUGCUUUUGCUGAUCUCGGCACG
Msu-1-1   CUAUUAUUUGCGCCUGUCCGUAACGGAUGUGUGCAAUUUCAAGUGUACCUAUUGUUUACCUAGUGGGUAUCAGCCUCCUGUGCAAAAAGAAAGUUUUUUGAGCUUGGAUGAAAUACGCCGUAUUGUCGGUGCUUUUGCGGCAAUGGGAACGGAAAAAGUG
Hso-1-1   UACUAUUAUUUACGCUUGUCUGUCACGGAUAUGUGUAAUUUUCGUUGUAACUAUUGUUUACCUCAUGGCUAUCGAUCGGAAAGCACAAAACCCAGUUUUCUGAAUCUGGUUGAAAUUAAACGAUUGGUGAAUGCCUUUGCACAAUUAGGUACAGAAAAAG
Hso-2-1   UACUAUUAUUUACGCUUGUCUGUCACGGAUAUGUGUAAUUUUCGUUGUAACUAUUGUUUACCUCAUGGCUAUCGAGCGGAAAGCACAAAACCCAGUUUUCUGAAUCUGGUUGAAAUUAAACGAUUGGUGAAUGCCUUUGCACAAUUAGGUACAGAAAAAG
Hin-3-1   UUACUAUCUACGGCUGUCGAUUACCGAUCAGUGUAAUUUUCGUUGUACUUAUUGUUUACCUGAUGGUUAUCAACCCGAAGCUAACAAACCAAGUUUCUUAACUUUAAAAGAAAUCACCCAUCUUGCUCAAGCGUUUGCUGAAAUGGGCACAGAGAAAAUC
Hin-4-1   UUACUAUCUACGGCUGUCGAUUACCGAUCAGUGUAAUUUUCGUUGUACUUAUUGUUUACCUGAUGGUUAUCAACCCGAAGCUAACAAACCAAGUUUCUUAACUUUAAAAGAAAUUACCCAUCUUGCUCAAGCCUUUGCUGAAAUGGGCACAGAGAAAAUC
Hin-2-1   UUACUAUCUACGACUGUCGAUUACCGAUCAGUGUAAUUUUCGUUGUACUUAUUGUUUACCUGAUGGUUAUCAACCCGAAGCUAACAAACCAAGUUUCUUAACUUUAAAAGAAAUUACCCAUCUUGCUCAAGCGUUUGCUGAAAUGGGUACAGAAAAAAUC
Hin-1-1   UUACUAUCUACGACUGUCGAUUACUGAUCAGUGUAAUUUUCGUUGUACCUAUUGUUUACCUGAUGGUUAUCAACCCGAAGCUAACAAACCAAGUUUCUUAACCUUAAAAGAAAUUAUCCAUCUUGCUCAAGCGUUUGCUGAAAUGGGCACAGAGAAAAUC
Pmu-1-1   UUACUAUUUGCGACUGUCAAUUACAGAUGUGUGUAAUUUCCGCUGUAAUUACUGUUUGCCGAAUGGUUAUCAACCUGAGUCAAAUAAACCCAGUUUUCUCACCUUAAAUGAAAUUCGUUUAGUGGUAAAAAGUUUUGCUAACAUGGGUACUGAAAAAGUC
Asu-1-1   UUAUCUGCGCCUGUCGAUUACGGACGUGUGUAAUUUCCGUUGUAAUUACUGUCUGCCCGACGGCUAUCAUCCCUCGCACGAGCGCGAUAAAUUUCUCACAGUGGAUGAAAUUCGGCGUGCGGUAUCUGCUUUUGCGGCAAUGGGAGCGCAAAAAGUGCGG
Hso-2-2   CUUUCACCAGUUCAUUGUGAGCCUAGUAAAUAACGAGGUUUAAAAUGAAAACACAAAAUAAAGUUAAUACAAGUCGCCGUGAUUUUUUAAAAAAUUCAUCAUUAGGCGUGGCUGGCGUAUCUCUAAGUGGCGGUGUUGUCGGAACAUUAGCUUCGAAAGC
Hso-1-2   CUUUCACCAGUUCAUUGUGAGCCUAGUAAAUAACGAGGUUUAAAAUGAAAACACAAAAUAAAGUUAAUACAAGUCGCCGUGAUUUUUUAAAAAAUUCAUCAUUAGGCGUGGCUGGCGUAUCUCUAAGUGGCGGUGUUGUCGGAACAUUAGCUUCGAAAGC
Mha-1-2   CGGUUUUCUAAAAAAUUCAUCAUUAAGCUUAGCUGCCGGUGCAGGUUUAGCCAGUGGUACUACCGGUGUAGUGGGGGCAUUAGCUUCCACCAGUGCCAAAGCAGCCGAGAUGAAAACAGUAGUAACUGCCGCCCACUGGGGGCCUUUAGGUGUAGUGGUA
Dha-2-4   GUGAAGGUGAGGGGAAUCUAUGCAAGAUCAAUAUCGGCGCCAGAUCGAAUACCUGAGGAUUUCCAUUACAGAUCGCUGCAAUUUACGAUGUAAAUAUUGCAUGCCCGCUGAAGGAGUCCAAUGGAUUCCUCAUGAGCGCAUCUUAACCUAUGAGGAAAUA
Dha-1-5   GUGAAGGUGAGGGGAAUCUAUGCAAGAUCAAUAUCGGCGCCAGAUCGAAUACCUGAGGAUUUCCAUUACAGAUCGCUGCAAUUUACGAUGUAAAUAUUGCAUGCCCGCUGAAGGAGUUCAAUGGAUUCCUCAUGAGCGCAUCUUAACCUAUGAGGAAAUA
Csp-1-1   UAUAAAAUCUCAAACUGGCAAGAAGUCCCUUAUCUAAAGUUAUAGUUGAAAUUUAAACUAAAGGGAGAGAUUGGUUAUGAAAAGAAGGUUUAGAGAAUUAUCGCUUAUUUUCCUACUAUUCCUGUGUAUUUUUGCUGUUGCUUGUUCUAAUCCAGCAACU
          ................................................................................................................................................................
          ................................................................................................................................................................
          ●●●●●●●●●●●●●●●●●●●●●●●●●●●●●●●●●●●●●●●●●●●●●●●●●●●●●●●●●●●●●●●●●●●●●●●●●●●●●●●●●●●●●●●●●●●●●●●●●●●●●●●●●●●●●●●●●●●●●●●●●●●●●●●●●●●●●●●●●●●●●●●●●●●●●●●●●
```

Dps-1-1	UACUUAUCUUAGGCUUUCCAUAACCGAUCGUUGUAACCUCCGUUGUCGUUACUGUAUGCCGGAAGAGGGUGUCCAGUUCCUUGAUCACUCUGAAAUUCUCUCCUAUCAGGAACUCCUCCGUAUCAGCCGUAUUUUUGUCGGAUUCGGGGUGAAUAAAAUU
Mth-1-2	AGCCGUUUAAACGGCCCUGCUACGAACGAAAACCCCGGGCUGGUAUGUGCUGGUAGGAGGCGAUCCCGGCUCGAAGGCAAACAGGGCCAGCCAAGGAGGAGUUAUUUUAGAGGAGGCUUUUUAUCAUGGUCAACCAGGACGCCAUUCGAGCGGCGGUAAA
Ame-1-1	UUUUUUGUAUUUAAGUGGGAUAUGAGUCGACGCCAUAGCGUCAGACGAAUUUGACGGGGGGAUAUUCAAAUGAUUGAGUUAUUAGAUACUGUAACACUAGAAGAGGCAAAACAGAUUGUCAUUGAAAAUUUCAAAGAAACAUCCUUGGAUAGUGAAAAG
Ame-1-2	AAAAAGUUAAUCCAAACACCAGCACAUCAUUAAGUAGAUGAUUAAGGGGAAAAUACAUGAAAGACACAUUCCAAAGAAGCAUUAACUACAUGAGGAUUUCUAUCACGGAUCUAUGUAAUUUGCGUUGUCAAUAUUGUAUGCCUGAAAAGGGAAUCUAUA
Ahy-1-1	UGCAAUUUUCGCUGUACCUAUUGCCUGCCCGAUGGUUACCGUCCACCGGCCGGUGGCAAGGUGGGGCGUCAGCCCUUCCUCUCUCUGGAAGAGAUACGCCGUGUUGUCUCCGGAUUUGCCGCCAUGGGCACCCGCAAGGUGCGCCUGACCGGGGGGGAGC
env-1	UGCAAUUUUCGCUGUACCUACUGCCUGCCCGAUGGUUACCGCCCACCGGCCGGUGGCAAGGCGGGGCGUCAGCCCUUUCUCUCUUUGGAAGAGAUCCGCCGUGUCGUGUCCGGAUUUGCCGCCAUGGGCACCCGCAAGGUGCGCCUGACCGGUGGGGAGC
Plu-1-1	UAUGUAAUUUUCGGUGCACUUAUUGUUUGCCGGAUGGUUAUAAACCCCGUGGACAUCACUCCUUUUUGACAUUACCCGAAAUCCGUCUUGUUGGCCGGGCAUUUGCCGAUCUGGGUACCGAGAAAAUCCGUUUGACGGGGGGUGAACCGACCAUGCGCCG
Sgl-1-1	CUGUAAUUUCCGCUGCACCUAUUGCCUGCCCGACGGUUAUCAUCCGCAGGGAACCCGCAAUACCGGCUUUCUCUCCUUGGCCGAGAUCCGUCGCGUCACCCGCGCCUUUGCCGAACUGGGGACAGAAAAAGUCCGCCUCACCGGCGGCGAACCGUCGCUG
Ymo-1-1	GCAACUUUCGCUGCACCUACUGUCUGCCGGAUGGCUAUCGGCCCGAUGGACUAAAAAGCUUUCUGAGCCUCGACGAAAUAAGCCGCGUUAGCCGCGCCUUUGCCCUGUUAGGUACUGAAAAAAUCCGCCUGACGGGCGGUGAACCCUCUAUGCGGCGUGA
Yps-2-1	GCAAUUUCCGUUGCACAUAUUGCUUGCCAGAGGGCUACCGCCCAGAUGGUGUCAAAAGUUUUUUGAGCCUUGACGAAAUCAAUCGCGUCAGCCGCGCCUUUGCCUUGUUGGGAACGGAAAAAAUCCGCCUGACGGGGGGCGAGCCUUCGAUGCGGCGCGA
Yps-1-1	GCAAUUUCCGUUGCACAUAUUGCUUGCCAGAGGGCUACCGCCCAGAUGGUGUCAAAAGUUUUUUGAGCCUUGACGAAAUCAAUCGCGUCAGCCGCGCCUUUGCCUUGUUGGGAACGGAAAAAAUCCGCCUGACGGGGGGCGAGCCUUCGAUGCGGCGCGA
Yfr-1-1	GCAAUUUUCGUUGCACUUAUUGCCUGCCCGAUGGCUAUCGCCCGGACGGAUUAAAAAGUUUUCUAAGCCUUGAUGAGAUAAGCCGCAUCAGCCGCGCUUUUGCUUUGCUGGGUACAGAAAAGAUCCGUCUGACCGGUGGUGAGCCCUCCAUGCGCCGCGA
Yin-1-1	GCAAUUUUCGUUGCACCUAUUGCUUGCCGGAUGGCUAUCGCCCCGACGGAUUAAAAAGCUUUCUGAGUCUGGACGAAAUAAGCCGCGUCAGCCGCGCCUUUGCUCUAUUGGGAACAGAAAAAAUUCGUCUGACGGGCGGCGAACCCUCUAUGCGGCGCGA
Ybe-1-1	GCAACUUUCGCUGCACCUACUGUCUGCCCGAUGGCUAUCGCCCCGACGGGCUAAAAAGUUUUCUGAACCUUGACGAGAUAAGCCGCGUUAGCCGCGCCUUUGCUCUGUUGGGAACGGAAAAAAUCCGCCUGACCGGUGGUGAACCCUCUAUGCGGCGUGA
Spr-1-1	UGUGUAACUUUCGUUGCACCUAUUGUUUGCCGGAUGGCUAUCGCCCUAACGGCAGCCCGAAAACCUUCUUGUCGCUCGAUGAGAUCCGACGUGUCAGCCGCGCCUUCGCCGAGCUGGGCACUGAAAAGGUGCGCCUGACCGGCGGUGAGCCUUCGCUGCG
Eca-1-1	UCUGCAAUUUUCGCUGUACCUACUGCCUGCCGGAUGGUUAUCAAGCCAAUGGCACCAAUCCACAUCGCUUUUUAUCACUCGAUGAGAUUCGUCGCGUCAGCCGCGCUUUUGCUGAAUUGGGGACGGAAAAAGUCCGUCUUACCGGCGGCGAACCGUCUCU
Esp-1-1	GUGUGCAACUUCCGUUGCACCUAUUGCCUGCCGAACGGUUACAAGCCCGGCAGCGUCACCAAUAACGGAUUUCUCUCCGUGGCAGAAGUGCGGCGCAUCACGCGCGCAUUCUCUGAACUCGGCACCGAAAAAGUGCGCCUGACGGGCGGAGAACCCUCAC
env-2	GUGUGUAACUUUCGUUGCACCUACUGCCUGCCGGAUGGCUAUAAGCCCGGUGGGGUCACCAAUAACGGCUUUCUGACCGUUGAUGAAAUUCGUCGCGUCACGCGCGCGUUCGCCAGUCUGGGAACGGAAAAAGUGCGUCUGACAGGGGGCGAACCUUCAC
Eco-17-1	GUGUGUAACUUUCGUUGCACCUACUGCCUGCCGGAUGGCUACAAACCGAGCGGCGUCACCAAUAAAGGCUUUCUUACCGUCGAUGAAAUUCGCCGGGUUACGCGCGCCUUCGCCAGACUGGGCACCGAAAAAGUGCGCCUGACAGGAGGAGAGCCGUCUU
Eco-6-1	GUGUGUAACUUUCGUUGCACCUACUGCCUGCCGGAUGGCUACAAACCGAGCGGCGUCACCAAUAAAGGCUUUCUUACCGUCGAUGAAAUUCGCCGGGUUACGCGCGCCUUCGCCAGUCUGGGCACCGAAAAAGUGCGCCUGACAGGCGGUGAGCCGUCUU
Eco-3-1	GUGUGUAACUUUCGUUGCACCUACUGCCUGCCGGAUGGCUACAAACCGAGCGGCGUCACCAAUAAAGGCUUUCUUACCGUCGAUGAAAUUCGCCGGGUUACGCGCGCCUUCGCCAGUCUGGGCACCGAAAAAGUGCGCCUGACAGGAGGAGAGCCGUCUU
Eco-1-1	GUGUGUAACUUUCGUUGCACCUACUGCCUGCCGGAUGGGUACAAGCCGAGCGGCGUCACCAAUAAAGGCUUUCUAACCGUCGAUGAAAUUCGCCGGGUUACGCGCGCCUUCGCCAGUCUGGGCACCGAAAAAGUGCGCCUGACAGGCGGUGAGCCGUCUU
Sbo-2-1	GUGUGUAACUUUCGUUGCACCUACUGCCUGCCGGAUGGCUACAAACCGAGCGGCGUCACCAAUAAAGGCUUUCUUACCGUCGAUGAAAUUCGCCGGGUUACGCGCGCCUUCGCCAGUCUGGGCACCGAAAAAGUGCGCCUGACAGGAGGUGAGCCGUCUU
Sso-1-1	GUGUGUAACUUUCGUUGCACCUACUGCCUGCCGGAUGGCUACAAACCGAGCGGCGUCACCAAUAAAGGCUUUCUUACCGUCGAUGAAAUUCGCCGGGUUACGCGCGCCUUCGCCAGUCUGGGCACCGAAAAAGUGCGCCUGACAGGAGGAGAGCCGUCUU
Sfl-3-1	GUGUGUAACUUUCGUUGCACCUACUGCCUGCCGGAUGGCUACAAACCGAGCGGCGUCACCAAUAAAGGCUUUCUUACCGUCGAUGAAAUUCGCCGGGUUACGCGCGCCUUCGCCAGUCUGGGCACCGAAAAAGUGCGCCUGACAGGAGGAGAGCCGUCUU
Eco-2-1	GUGUGUAACUUUCGUUGCACCUACUGCCUGCCGGAUGGUUACAAGCCGAGCGGCGUCACCAAUAAAGGCUUUCUGACCGUCGAUGAAAUUCGCCGGGUUACGCGCGCCUUCGCCAGUCUGGGCACCGAAAAAGUCCGUCUGACAGGUGGUGAGCCAUCUU
Sfl-2-1	GUGUGUAACUUUCGUUGCACCUACUGCCUGCCGGAUGGCUACAAACCGAGCGGCGUCACCAAUAAAGGCUUUCUUACCGUCGAUGAAAUUCGCCGGGUUACGCGCGCCUUCGCCAGUCUGGGCACCGAAAAAGUGCGCCUGACAGGAGGAGAGCCGUCUU
Sdy-1-1	GUGUGUAACUUUCGUUGCACCUACUGCCUGCCGGAUGGCUACAAACCGAGCGGCGUCACCAAUAAAGGCUUUCUGACCGUCGAUGAAAUUCGCCGGGUUACGCGCGCCUUCGCCAGUCUGGGCACCGAAAAAGUCCGUCUGACAGGUGGUGAGCCGUCUU
Sbo-1-1	GUGUGUAACUUUCGUUGCACCUACUGCCUGCCGGAUGGCUACAAACCGAGCGGCGUCACCAAUAAAGGCUUUCUUACCGUCGAUGAAAUUCGCCGGGUUACGCGCGCCUUCGCCAGUCUGGGCACCGAAAAAGUGCGCCUGACAGGAGGUGAGCCGUCUU
Eco-15-1	GUGUGUAACUUUCGUUGCACCUACUGCCUGCCGGAUGGCUACAAACCGAGCGGCGUCACCAAUAAAGGCUUUCUGACCGUCGAUGAAAUUCGCCGGGUUACGCGCGCCUUCGCCAGUCUGGGCACCGAAAAAGUGCGCCUGACAGGCGGUGAGCCGUCUU
Eco-14-1	GUGUGUAACUUUCGUUGCACCUACUGCCUGCCGGAUGGCUACAAACCGAGCGGCGUCACCAAUAAAGGCUUUCUGACCGUCGAUGAAAUUCGCCGGGUUACGCGCGCCUUCGCCAGUCUGGGCACCGAAAAAGUGCGCCUGACAGGCGGUGAGCCGUCUU
Sty-1-1	GUGUGUAACUUUCGUUGCACCUACUGUCUGCCGGAUGGCUACAAACCUGGCGGCGUCACUAACAACGGUUUCCUUACCGUCGAUGAAAUUCGUCGCGUUACGCGCGCUUUUGCCAGUCUUGGCACGGAGAAGGUGCGUCUUACCGGCGGCGAGCCUUCAU
Sen-1-1	GUGUGUAACUUUCGUUGCACCUACUGUCUGCCGGAUGGCUACAAACCUGGCGGCGUCACUAACAACGGUUUCCUUACCGUCGAUGAAAUUCGUCGCGUUACGCGCGCUUUUGCCAGUCUUGGCACGGAAAAGGUGCGUCUUACCGGCGGCGAGCCUUCAU
Sen-4-1	GUGUGUAACUUUCGUUGCACCUACUGCCUGCCGGAUGGCUACAAACCUGGCGGCGUCACUAACAACGGUUUCCUUACCGUCGAUGAAAUUCGUCGCGUUACGCGCGCUUUUGCCAGUCUUGGCACGGAGAAGGUGCGUCUUACCGGCGGCGAGCCUUCAU
Sen-2-1	GUGUGUAACUUUCGUUGCACCUACUGUCUGCCGGAUGGCUACAAACCUGGCGGCGUCACUAACAACGGUUUCCUUACCGUCGAUGAAAUUCGUCGCGUUACGCGCGCUUUUGCCAGUCUUGGCACGGAAAAGGUGCGUCUUACCGGCGGCGAGCCUUCAU
Sam-1-1	UGCAACUUCAAAUGCAGCUAUUGUCUGCCCGACGGCUACCACCCGGAUGGUAGGCCGAGUUUCUGACCCUCAGUGAAAUCGAAAAUCUGAUUGCCGCCUUUGCCAAGGCCGGUACCCGCAAGGUGCGCAUCACUGGCGGUGAGCCAACCCUGCGCAAAG
Spe-1-1	UGUAACUUUAAGUGCACGUAUUGCCUCCCAGAUGGCUACCGCCCAGACGGUAAGCCUAAAUUCCUCGAUCUCAAUGAAAUCGAGCAUCUGGUUUCGGCUUUUUCACAAGUAGGCACCCAAAAAAUCCGUAUUACCGGUGGUGAGCCUUCUUUGCGUAAAG
Ssp-4-1	UGUAACUUCAAAUGCACAUACUGCCUCCCAGAUGGUUAUCGCCCCGACGGUAAGCCUAAGUUUUUAGAUCUCAACGAGAUCGAGAAUCUAGUGGGCGCCUUUGCCGAGGUCGGCACCCAGAAGAUCCGCAUCACGGGCGGCGAGCCCACAUUGCGUAAAG
Swo-2-1	UGUAAUUUCAAAUGUACUUACUGCCUCCCCGAUGGCUAUCGACCUAAUGGUCGUUCUAAGUUUUUGGCGCUUAGCGAGAUUGAAAACUUAGUCGCAGCUUUUGCUGAAGUCGGUACUCAAAAAAUUCGUAUCACUGGCGGCGAACCGACGCUGCGUAAAG
Ssp-5-1	UGUAACUUCAAAUGCAGCUACUGUCUCCCCGAUGGUUAUCAUCCUGAUGGUAAACCAAAAUUCUUAUCUCUUAAUGAGAUAGAAAACCUCGUGUCUGCAUUU..
Ssp-5-2	UGUAACUUCAAAUGCAGCUACUGUCUCCCCGAUGGUUAUCAUCCUGAUGGUAAACCAAAAUUCUUAUCUCUUAAUGAGAUAGAAAACCUCGUGUCUGCAUUUAGCCAAGUGGGUACUCAAAAAAUCCGCAUAACGGGCGGUGAGCCUACCCUACGUAAAG
Spu-1-1	UGUAACUUCAAAUGUAGCUACUGUCUCCCCGAUGGUUAUCAUCCUGAUGGUAAACCAAAAUUCUUAUCUCUUAAUGAGAUAGAAAACCUCGUGUCUGCAUUUAGCCAAGUGGGUACUCAAAAAAUCCGCAUAACGGGCGGUGAGCCUACCCUACGUAAAG
Sba-1-1	UGUAACUUCAAAUGCAGUUAUUGCCUCCCCGACGGUUAUCAUCCUGACGGUAAGCCAAAAUUUUUAUCUCUCAAUGAGAUAGCAAACUUAGUCUCUGCAUUUAGCCAAGUCGGCACCCAAAAAAUCCGCAUUACAGGCGGUGAACCCACCCUGCGUAAAG
Sba-2-1	UGUAACUUCAAAUGCAGUUAUUGCCUCCCCGACGGUUAUCAUCCUGACGGCAAGCCAAAAUUCUUAUCUCUCAAUGAGAUAGAAAACUUAGUCUCUGCAUUUAGCCAAGUCGGCACCCAAAAAAUCCGCAUUACAGGCGGUGAACCCACCCUGCGUAAAG
Ssp-1-1	UGUAACUUCAAAUGCAGUUAUUGCCUCCCCGAUGGUUAUCAUCCGAAUGGCAAACAACAGUUUUUAUCUCUCAGUGAGAUAGAAAACCUUGUGUCCGCAUUUAGCCAGGUUGGCACUCAAAAAAUCCGUAUCACUGGCGGCGAACCCACGCUGCGCAAAG
env-4	UGUAACUUCAAAUGCAGCUAUUGCCUCCCGGAUGGUUAUCAUCCGAAUGGCAAACAACAGUUUCUAUCUCUCAGUGAGAUAGAAAACCUUGUGUCCGCAUUUAGCCAGGUUGGCACUCAAAAAAUCCGUAUCACUGGCGGCGAACCCACGCUGCGUAAAG
Ssp-2-1	UGUAACUUCAAAUGCAGUUAUUGUCUCCCCGAUGGUUAUCAUCCGAAUGGUAGACAACAGUUCUUAUCUCUCAGUGAGAUAGAAAAUCUUGUGUCCGCAUUUAGCCAGGUUGGCACUCAAAAAAUCCGUAUCACUGGCGGCGAACCCACGCUGCGUAAAG
Ssp-3-1	UGUAACUUCAAAUGCAGUUAUUGUCUCCCCGAUGGUUAUCAUCCGAAUGGUAGACAACAGUUCUUAUCUCUCAGUGAGAUAGAAAAUCUUGUGUCCGCAUUUAGCCAGGUUGGCACUCAAAAAAUCCGUAUCACUGGCGGCGAACCAACGCUGCGUAAAG
Son-1-1	UGUAACUUCAAAUGCAGUUAUUGCCUCCCCGAUGGUUAUCAUCCCAAUGGCAAGCAGCAGUUUUUAUCUCUCAGUGAGAUAGAAAACUUGGUGUCCGCAUUUAGCCUCGUUGGCACUCAAAAAAUCCGUAUCACUGGCGGCGAACCCACGCUGCGUAAAG
env-5	UGUAACUUCAAAUGCAGCUAUUGCCUCCCCGAUGGUUAUCAUCCGAAUGGCAAGCAACAGUUUUUAUCUCUCAGUGAGAUAGAAAACUUGGUGUCCGCAUUUAGCCUCGUUGGCACUCAAAAAAUCCGUAUUACUGGCGGCGAACCCACCCUGCGUAAAG
Sfr-1-1	UGCAAUUUUAAAUGUACUUAUUGUUUACCCGACGGAUAUCGUCCAGAUGGUAAACCUACCUUCUUAACGCUCAACGAGAUUGAAAAUCUGGUUGCCGGUUUCGGUGAAGUAGGCACACAAAAAGUUCGUAUCACUGGUGGCGAGCCCACCUUACGCAAGG
Sde-1-1	UGUAAUUUUAAAUGUACCUAUUGUUUACCCGAUGGCUAUCAAAGCCAAGGAAAUAGCCCGUUUUUGUCCUUAAGUGAGAUAGAGCUGCUAUUGGGGGCAUUUUCUCAAAUGGGCACUCAAAAAGUGCGCAUUACCGGCGGUGAACCUAGCCUGCGCAAAG
Vch-3-1	CGUUUGUAAUUUCAAAUGUACGUAUUGCUUACCUGAUGGCUAUAAGCCGUCAGCGGAUCGUCCUGCCUCUUUUCUGACCGUCAACGAAAUUCGUCGUGUGGUGAAUGCGUUUGCCCAUUGUGGCACUUCAAAGGUGAGAAUCACCGGCGGUGAACCUUCG
Vch-1-1	CGUUUGUAAUUUCAAAUGUACGUAUUGCUUACCUGAUGGUUACAAGCCAUCAGCGGAUCGCCCUGCCUCUUUUCUGACGGUGAAUGAGAUUCGCCGAGUGGUGAGUGCGUUUGCCCAUUGUGGCACUUCCAAGGUCAGAAUUACCGGCGGUGAACCUUCA
Vch-8-1	CGUUUGUAAUUUCAAAUGUACGUAUUGCUUACCUGAUGGUUACAAGUCAUCAGCGGAUCGCCCUGCCUCUUUUCUGACGGUGAAUGAGAUUCGCCGAGUGGUGAGUGCGUUUGCCCAUUGUGGCACUUCCAAGGUCAGAAUUACCGGCGGUGAACCUUCA
Vch-10-1	CGUUUGUAAUUUCAAAUGUACGUAUUGCUUACCUGAUGGUUACAAGCCAUCAGCGGGUCGCCCUGCCUCUUUUCUGACGGUGAAUGAGAUUCGCCGAGUGGUGAGUGCGUUUGCCCAUUGUGGUACUUCCAAGGUCAGAAUUACCGGCGGUGAACCUUCA
Vch-13-1	CGUUUGUAAUUUCAAAUGUACGUAUUGCUUACCUGAUGGUUACAAGCCAUCAGCGGAUCGCCCUGCCUCUUUUCUGACGAUGAAUGAGAUUCGCCGAGUGGUGAGUGCGUUUGCCCAUUGUGGCACUUCCAAGGUCAGAAUUACCGGCGGUGAACCUUCA
Vch-7-1	CGUUUGUAAUUUCAAAUGUACGUAUUGCUUACCUGAUGGUUACAAGCCAUCAGCGGAUCGCCCUGCCUCUUUUCUGACGGUGAAUGAGAUUCGCCGAGUGGUGAGUGCGUUUGCCCAUUGUGGCACUUCCAAGGUCAGAAUUACCGGCGGUGAACCUUCA
Vch-12-1	CGUUUGUAAUUUCAAAUGUACGUAUUGCUUACCUGAUGGUUACAAGCCAUCAGCGGAUCGCCCUGCCUCUUUUCUGACGGUGAAUGAGAUUCGCCGAGUGGUGAGUGCGUUUGCCCAUUGUGGCACUUCCAAGGUCAGAAUUACCGGCGGUGAACCUUCA
Vvu-1-1	CGUGUGUAAUUUCAAAUGUACCUAUUGUCUGCCUGAUGGUUACCAACCUUCGGGGCAGAAAAACUCGUCUUUUUUAAACCUCUCUGAAAUCCGUCGAGUCGUUAAGGCUUUUGCUGACUGUGGCACAAGUAAAGUGCGCAUUACUGGCGGAGAGCCAAGU
Vvu-2-1	CGUGUGUAAUUUCAAAUGUACCUAUUGUCUGCCUGAUGGUUACCAACCUUCGGGGCAGAAAAACUCGUCUUUUUUAAACCUCUCUGAAAUCCGUCGAGUCGUUAAGGCUUUUGCUGACUGUGGCACAAGUAAAGUGCGUAUUACUGGCGGAGAGCCAAGU
	....
	....
	••

```
Vsp-3-1   CGUCUGUAACUUUAAAUGUACUUACUGCUUGCCAGAUGGUUAUAAACCGUCAGGGCAAAAAAACUCGUCUUUUUUAAGUGUUCCCGAGAUCAAACGUAUUGUAAAAGCGUUUGCAGAUUGUGGUACCUCAAAGAUCCGCAUUACAGGCGGAGAGCCGAGC
Vsp-2-1   CGUCUGUAACUUUAAAUGUACUUACUGCUUGCCAGAUGGUUAUAAACCGUCAGGGCAAAAAAACUCGUCUUUUUUAAGUGUUCCUGAGAUCAAACGCGUUGUUAAAGCGUUUGCUGAUUGUGGUACCUCAAAGAUCCGCAUUACUGGCGGAGAGCCGAGU
Vpa-1-1   CGUAUGUAAUUUCAAAUGUACCUAUUGCCUUCCUGAUGGUUACAAACCAUCGGGUACAAAAAACUCGUCUUUUUUAUCUCUUCCUGAAAUCAAACGGGUCGUAAAAGCGUUUGCUGAUUGUGGUACGUCAAAAGUGCGCAUUACUGGCGGCGAGCCAAGC
Vsp-1-1   CGUAUGUAAUUUCAAAUGUACCUAUUGCCUUCCUGAUGGCUAUAAGCCUUCGGGCAAUAAAAACUCGUCUUUUUUAUCUCUUCCUGAAAUCAAACGGGUCGUAAAAGCGUUUGCUGAUUGUGGCACGUCAAAAGUGCGCAUCACUGGCGGUGAACCAAGU
Val-1-1   CGUAUGUAAUUUCAAAUGUACCUAUUGCCUUCCUGAUGGCUAUAAGCCUUCGGGCAAUAAAAACUCGUCUUUUUUAUCUCUCCCUGAAAUCAAACGGGUCGUAAAAGCAUUCGCUGAUUGUGGCACGUCAAAAGUGCGCAUCACUGGUGGUGAGCCUAGC
Vfi-1-1   UGUCUGUAACUUUAAAUGUACUUAUUGCUUACCUGAUGGUUAUAAACCAGAAAAUGGAAAGCGUGAGUCCUUCCUUGAACUUGAUGAAAUCGAACGUACUGUUAGUGCGUUUGCUCGUUGUGGUACGUCUAAAGUACGUAUUACAGGGGGAGAACCAAGU
Psp-1-1   UGUCUGUAACUUCAAAUGUACCUAUUGCUUGCCAGAUGGCUAUCAUCCAACAGAAAAGAAAAAACCUUCCUUUCUAACAUUGGGUGAGAUUCAGCGAGUUGCCUUAGCAUUUGCUGAGUGUGGCACUAGUAAAAUCCGUAUCACAGGUGGUGAGCCGAGU
Van-1-1   UGUCUGUAACUUCAAAUGUACCUAUUGCUUGCCAGAUGGCUAUCAUCCAACAGAAAAGAAAAAACCUUCCUUUUUAACAUUGGAUGAGAUUCAGCGAGUUGCCUCAGCGUUUGCUGAGUGUGGCACCAGUAAAAUACGUAUCACAGGUGGUGAGCCGAGC
Ppr-3-1   GCGACUAUCAGUCACAGAUGUCUGUAAUUUUAAAUGUACUUACUGCUUGCCUGAUGGCUAUAAGCCGACAGAAAAAAAGAAACCUUCUUUUCUUACAUUGAAUGAGAUUGAGCGCGUAACAAGGGCUUUUGCUGAAUGUGGAACGUCAAAAGUACGUAUC
Ppr-2-1   ACGACUAUCAGUCACAGAUGUCUGUAAUUUUAAAUGUACUUACUGCCUGCCUGAUGGCUAUAAGCCGACAGAAAAAAAGAAACCUUCUUUUCUUACAUUGGAUGAAAUUGAGCGCGUAACAAGGGCUUUUGCUGAAUGUGGAACGUCAAAAGUACGUAUC
Dre-1-2   UUUUUUAUGAGGAGGCAGUCAUGACUAAGGUAAAAGAAUUGGUUAAUAAUAAACAAGUGGAAUUGGACAGGUUAACCGAUGUGUGUGAAAGUUAUCUUGAUGAUAUAAAUAUUCUGUCUCAAGCUGUUGACUAUCUAUUACAGCAACGUAGCGAAGGAAA
Swo-1-1   UAACGAGGUGAAUUAAGUCUCCCACUUCUGCCGGGUUCCUAUUAACAAACAGGUGGCGGGUUCUAACCCCCGCCCUCGUUGCAGCGGUGUGUUGAAAGUGCUCCGCAGUUUCUUCCGAUGCUUAAAGGGUUGUUUAUUGAUUUAUUAAGUGGCCUCGUAU
Pat-1-1   UUCAUUACCUGCGGCUGUCCAUUACUGAUGUGUGUAAUUUUAGUUGUGAGUACUGUCUACCUGAUGGGUAUCAGUGCGAUACCGACAGGGAUUUCUUGUCUCUGAUUGAAAUUACACGCAUUGCAGGUGCCUUUGCGAAACUAGGCACCAGCAAAAUUCG
Pat-1-2   UGUAAUUUUAGUUGUGAGUACUGUCUACCUGAUGGGUAUCAGUGCGAUACCGACAGGGAUUUCUUGUCUCUGAUUGAAAUUACACGCAUUGCAGGUGCCUUUGCGAAACUAGGCACCAGCAAAAUUCGUAUAACGGGUGGCGAGCCUUCGCUACGAAAAG
Swo-1-2   UUUUAUUUCUUGUCGUUAAACCUUGGAAAGGGGUUCGGCGGUGAAAGUAUCAAGCAUUGCCGGGUUUCCGCAAAGGAAUCUGGUUAAGCCUGAUAUUUAUGCCCAAAAUGGGGCCCUGAAUCAAGGAGAAGUUGCCUUAUAGUUCAAUUCCAAAUUAUCU
env-6     .......................................................................................................
env-7     CAUGGCCUGUAGUUAUUGUGUCACCGGAAUCGAGGAUCAUCAGAUCGCCCCGGACCAGUUGGUCAUGCCGGAUUUGCUCCGGCUGGUGGAGUUACUUCAUCGUCACGCGGGGAUCGAAAAAAUCCGCAUCACCGGUGGCGAACCCCUGCUUUAUCGUGAU
Swo-1-3   ACCGAUCAGAAGGAACUGGUGGUAAAGCUAAAGGAAUUAUUUCCCCAGGGGAGAAUCACUUGUACCGAAGCUCAUGAUCUGGCCCGGGAGUGGGAUAUGGAUCCUCGGGAGCUGGGAGAAGCCUGUAAUGAGGCAGGCAUCAAGAUUUCUGGUUGUGAAU
Swo-1-4   ACCGAUCAGAAGGAACUGGUGGUAAAGCUAAAGGAAUUAUUUCCCCAGGGGAGAAUCACUUGUACCGAAGCUCAUGAUCUGGCCCGGGAGUGGGAUAUGGAUCCUCGGGAGCUGGGAGAAGCCUGUAAUGAGGCAGGCAUCAAGAUUUCUGGUUGUGAAU
Chy-1-1   AACCGGGAAAUAAACUAUUUGCGGAUUUCGGUUACCGACAAAUGUAACCUUAACUGUUUUUACUGUAAACCCGGAAAUUUUCAAGAAUUUUCGCCGGGAGAUAUUUUAACCUUUGAGGAAAUUUUAGAAGUAGUCCGGGCCUUUUUACCCUUGGGUUUAA
Swo-1-5   GAGCUGUUUCCUGCCUGAAGAUGAGGAUAAAGCCAGCAUCAACUUUUCGAUGCUGGCUUUUAUUUCCCCGUUGUUCAUAGAAUCACCCUCAUAAAAGCGAGACGAGGUCUGUUAUGAAAUUUACGGAAAGGAGGUGCUGAUGAAAGACCUCGCUGAAAUA
Mth-1-3   CGGCGCAGGGGGCCGUAAUGGCAAUUUAAGGCGGAUAGGGCCUCACAGUGGCUGACGCCCCCGCCAGUGAAGAAUGAAAAUGUGGGCUGAGGACCAUUUUGCCAGAAGUAUAUUUGGGAGGAGCCCAAGAGUAUGAAAUUACGGCGCGCAUCAAUAUU
Swo-1-6   UUAUUCCAGCCCGGUACUGCUUUCUGCCGGGUUUUAGUAUUUCUAGGAUAAUUAACGUUCUAACUGUAAUAAUGGAGGAGGUUAUAGAUAGUAAAGGAUUAAUUAAGUCGAUAAUAUGAGGCGAGAAACUUAUAUAUAGAUAGAGAUAUUAAGAUAGAGA
Csa-1-1   ACUAUGUUAAAAAAAAGGAGUGUUAAAAGAUGAAAAAAGUUAUAUUUAAAGUUGUUUUAGUAUGCAUUCUUUUAACUUUUGUCUCUACUUUCUCACUGUAUUCUUUUUCAAAACCUGUUAAAACAUAUAAAAUAGCAACAACUACAAGUAUAUAUGACAG
Dac-1-1   UAACAUGACCGACCUGACCACCACUGUUGAAGAUGUCCCUGAAGCAUGGCUCGGCCUCGGUGGUCGUGCUCUGACUUCAACCAUCGUUGCUACCGAGGUUGAUCCUGAAUGCCAUCCCCUUGGCCAGUUCAAUAAGUUGGUUUUUGCCCCCGGCCUGCUC
Dac-1-2   AUUUCAACCCCUUUGUUUGAAGGAAAAAUUGUUAUGAAACAACUGCGAAUGGUACUCUUUUCAUUGAUGAUCGUCCUGCUGCUGGCUGGGGCCGCCUUGGCUCAGGAGCACCUGAAGCUGGCCACGACCACAUCGACGGAAAACUCCGGUCUGCUCAGUG
Hne-1-1   GUCGGUUACAGAAGUCUGCAAUUUUCAGGCGGAUACCUACUGCUUGCCGGAUGGAUACCGAAAAACCGGUUCCAUGGACUUUCUCGCCGCAGACGAGAUUGAACGGCUCGGUCGCGCCUUCACCGGGCUUGGCAUCCGUAAGGUCCGGCUGACAGGCGGCGAA
Swo-1-7   AAAUUGAGUGUCGGUUCUGCGUCUACAAAUAAUAUUUAGGAGGUAUUAUUGUGUUGAAGUUCAGGCAGGAAUCCAGGUCUUCUUUGGUAUUAGCGCUGGUUGUUCUUUUUUGCUUAUUCCUAACCUUACCGGUACUGGCUGAAGGUGGUAGUGGUGACGG
Dac-1-3   UUAACAUGACCGACCUGACCACCACCGUUGAAGACGUCCCGGCUGAAUGGGCUGGUCUCGGUGGUCGUGCCCUGACCUCAACCAUCGUAGCUACCGAGGUUGAUCCUGAAUGCCAUCCUCUGGGAGAAAAAAACAAACUCGUUUUUGCCCCUGGCCUGCU
Gsp-1-1   CACCGGCACCGAAUCGGUACCAAAGGAAUGGGCAGCUCUCGGCGGUCGCGCCCUGACUUCCACCAUCGUGGCCAAGGAGGUUCCCCCCACCUGCCACCCCCUUGGGGAAAACAACAAGCUGGUCUUUGCCCCUGGACUGUUGAGCGGCACCACUGCCGCU
Gsp-1-2   CAUUUGGGGGAGACCUUUUCCCCUGUUAUUAGAAAACAGGAGGGUACGAUGAAGAAACGCAGCUUAGCAGCAACAGUGGUGGCAAUGGGCCUGGUGCUCAUCCCGGGUGUUCAUGCCAAAACGCUUGAGGAUGUGCUCAAGGAGAAAGGGGUCAUCACCG
Gsp-1-3   UUUCUGAAGCACGGGGUAAGGAGCCUUCAAUGUCGCUGGCAAAGAGGCCCUUAAGUACAUUCUGUAUCCACAGGAGGGGAUGAACUAGGUGGGUUUUUUUCCGAAUCACUGCAGACAGCUUUCAGCCUCAUCAUGUCCAUGGACCGGGAGGUCUAUACG
Pca-1-2   AACAUGUCUGAUCUGACCGUCAAGACCGAAGAGGUACCCGCCGAAUGGAAAACCCUCGGUGGCCGUGCCCUGACCACCACGAUUGUGGCGGCCGAGGUAAACCCCACCUGUCACCCGCUCGGUCCCAAUAAUAAACUCAUUUUUGCCCCGGAAUGCUGU
Gsp-1-4   UGAUCACGUGCUUCGAACAUGACUUCAAAACCAGAUAAAUGAUUCUCAACUCUCCGAGCCACGCCGCCUAAAGGGUAGUUUUCCCAUGGCGACCGGCUAGGGUGCCGCUGGAAACGGCUGUGCCUCCCUUUUGGAAAGGAGAGCCUCAAAGUACCGGACC
Ppr-1-1   GACCGACCUGACCAUGAAGACCGAGGCGGUGCCGGAAGCCUGGGCAGCCUAUGGCGGGCGCGCCCUGACCUCCACCAUCGUGGCAGCCGAAGUCCCCCCCACCUGCCACCCCCUGGGCCCGAACAACAAACUGGUAUUCGCUCCCGGCCUGCUAUCCGGA
Gur-1-1   GAUCGACAUGGGGGCGGCAGGCGGCCCCAUGGUAACGUUUGAAGAAGUCGGCGAGUAUGCCGGACUCGGCGGCAGGGCGAUGACGUCCCUCAUCAUCGCCAAGGAAGUGCACCCCCUCUGUCAUCCGCUGGGAGCGGAGAACAAGCUGGUCAUCGCCCCC
Gur-1-2   GCUGCGCCGCUGCUGGUCCCUUUUGCACAGCCGCUGGUCGAGCACCCCGCAAAAUCCCCGGGAAUUUGCAUCCUGGCCAAUCCAAGAUACACUUGAAAACGGCAGUCAGGCAGUCAUUCCAUGUUAUUUAGGGGACGCUUGAAAUGUUCAAGAAAGGGGA
Gsu-1-1   AUCCCGGGAGGAAGCAUCCAUGGUCAUCAUUACCGACACCCCCAUCGAUCCCACAUCUGCCUACGGGCUUCUCUCAAAAGGGACUUCAGGCUCGAUUGUCCUUCACUACGCCGUGGUGAAACAGGACGCCGGCAACAGCGCGCCUACCGCCUGCAUCGAC
Gme-1-1   CCGGCAGGCCUGGCCUGUUCCGGCAUGGUGAGCGUGACUCACAACUAUACCCCCCUAGGAGGAACACAUGAAACACAGAAACAUCGCGGCAGUUUCGGCCAUUCUUGCGGCCACUAUGGCCUGCACCACAGCGGAGGCCAAGACCUUGGAGGACAUCCUC
Gur-1-3   CCCAAUUCCGCAUGAUGACAAGGAAGGAAAUAAGCAAUGGUCAUAGUGUCAACAGAACCCAUCAAUCCAUCGGAAGCCUACGGGUUGAUCGAAAAGAGCAGGUCGGGAUCGGUCGUUCUUCAUUACGCAGUGGUCAAAGAGGAUAUCGGCGUCGGCAAAG
Pca-1-3   GUUAACAUGACGGACCUCACCUGCAAAGUUGAGGAAGUCCCCGCCGAAUUCGCAACCUUGGGUGGUCGCGCCCUGACCUCUACCAUCGUUGCGGCGGAAGUACCCCCCAGCUGUCAUGCCCUCGGCCCCAACAACAAGCUGGUUUUCGCCCCCGGCCAGC
Gme-1-2   CAUGGCCGACCUGAGCACCAGGAUCGAGGCGGUCCCCGCUGAAUGGGCGGGGCUUGGCGGCCGCGGCCUGACCUCGACCAUCGUUGCCGCAGAGGUCCCCCCCACCUGUCACCCCUGGGCCCCAGCAACAAACUUGUCUUCGCCCCGGGACUCCUGACC
Pca-1-4   UUGCGUCGGGUGUUAUUGUAGCAGAGAUUUUUUGACUUGCAGGGUCAGGUCAAAAAAAAUUGAGCAAUACGCUGUUUUAUCGAUUGCGCAAUGUUCGAUCCGGGGGUAGGACUAAAAGGAAAAGUCCUUGUCUUGAAAAAUAUUUAACGGUGCCGUGCCG
Ppr-1-2   ACAUGACAGACAUGACCACCAAGGUCGAAGAAGUACCGGCUGAAUGGGCUGGUCACGGUGGACGUGGCCUGACCUCAACCAUCGUUGCCGCAGAAGUACCCCCCACAUGCCAUCCGCUGGGACCGAACAACAAGCUGGUAUUCGCACCUGGCCUGCUCUC
Pca-1-5   GAUCGCGGUCAUUGAUUUGACCAACGAAACAGUGGAAUACAUCGAGCCCAGUGAAGAUGACCUGAAAAAAAUAUAUUGGCGGCAGCGGCUGGGUGCUAAGUUGCUGUAUGAAAACACCGCGCCGGUAUCGACCCGUUGGGCCGGAAAACGUCCUGAUC
Gur-1-4   ACAUGACCGACCUGACCACCAAGCUUGAGACAGUGCCCACCGAAUGGGCCGGUCUCGGCGGCCGCGGUCUGACCUCCACCAUCGUCGCCGCAGAAGUGCCGGCCGACUGUCACCCCCUCGGACCCAACAACAAGCUGGUAUUUGCUCCGGGACUCCUUAC
Gsp-1-5   ACAUGACCGACCUCACCACCAGGACCGAGCCGGUACCGGCCGCCUGGGCCGGUUUGGGGGGCCGUGCCCUUACCUCCACCAUUGUUGCGGCCGAAGUGCCUGCCACCUGCCAUGCCCUGGGUGAGAACAACAAGCUGGUCUUUGCCCCCGGGCUUCUCAC
Gme-1-3   UUUGCGGGAUAGACCGCGUACAACGUGUCCGGUGUGAUGCCGGAUUACGACAACAAGUGGAGAGAGGAGGAGGAUAUGAAAUACGAAGAGGUCGGAUGUUAUCUGGAAAUCAAUCUGGCAACGGGAACUAUCGAACGGGUGGCGCUGGAACCCGAGG
Gme-1-4   UUUCCCUCCGAGGCUCUAGUGCGGGCUGUCGGCGGAAAGCGGGUCUUGAAAAAAUUUAAAACCGUCUCCGAGCCGCGGCGCCUAAAGGGAACAUCCCCAUGGCGGUCCGGCCUAUGGGUAUCGCUGGAAACGGCGUUGCCUUCCUUAUGCAAAGGGGACC
Csp-2-1   UGUGCAACUACCGCUGCACCUAUUGCCUGCCCGACGGUUCAAGAAGUCGGGGACGAUGGCCUUCCUGACGCCCGAUGAAAUCGACCGCGUGGUGCGCCUUCGCCAGCUUCGGCGUCGCCAAGCUGCGUCUGACCGGCGGCGAGCCCAGCGUGCGGCG
env-8     UUCACGCUCCCGAUCCUGGGCAAAAAGCCGGAAGUCAUUUGACUUCCGGCUUUAAUCCAUCAAAGGAGGGAAAUUACCUUGAAAAACUAUGACUUACGAACAAUUAUUUUUGUGGCUUUGUGCUGUGACCUGGGUCUGUUCGCCAAGCGCGUCAUCGCGC
Tet-1-1   AAACGAUGCAGUGCCUUCUCCGGAAGGCAUUUUUUUGUACCUUUGUUAGGACUAAUUUCAAUGGUAAAAGCUAAAGAAUGGAAUCUUUCCAGUUUUAAUGGGUAAGGUUUAAACAAAAACUAAUAGUGAUGAUGGAGGAUGAAUACCAUGGCAAGAAUA
env-9     AUGGCCUGUACUUAUUGUGUAUCCAGUUUAAAUGACCAUAGACGCUCACCAGGAGAAUUAAGCACAGAAAAAUUAUUGAAACUAGUUAGUAUGUUAGUGAAACAUGCUGGUAUUGAAAAAGUUCGUUUGACUGGGGGAGAGCCUCUAUUACAUGCUAAUC
env-10    AUGGCUUGCACGUAUUGCGUGACAAGCCUGAGCGAUCAUAAACGUUCUCCGGAAGAAUUAAAUACGGACCAGAUACUAUAUCUUGUCCGAUUGUUAAAAGAUCAUGCAGGAAUUGAGAAAGUACGCCUAACUGG.........................
Swo-1-8   UUAAUUGAAUAUUUGUUUCCAAGCCUUUGAACCUAAGGUUUAUAACUAUGGUUCCCAGGCCGAUGGGUAUAGAUUGGAAACGGCUAUGCCUCCCCGUAUUGUGGAAAGGAGACGACGAGGGCUUAUUUUAUAUAGGUUUAAAUGGUCGUGUUCUUUUUCC
Swo-1-9   GUCCAGCAUUUCUUUAGCUGGGCUUUUUUAAUGCAGAUUUAAAUUAUCUCAAAGUAAAUAAAAUUAGCCGGAAGUAUCGCUGAACUAGGAGUAAUGAAUAUCGUUCGGGCCAGUGCUUUUUAGUUAUCAUCAAAAAAGCCUCACAACUAUAAGGGUGU
Swo-1-10  AAGAAGGAAAAAAGCGGGAUUCGUUCUGGUUCUCCCUAAGGUUUUGGGAAGCAAACGGGUACAGGUAGGCAUAAAAGCUAACGUCUCAAUUUAAUCAGGUAAUAUGCUGCAAGGCUAUUACAUACAAUCCCAAUCUUUUUCUCCCCGCUUGCAAACAAGC
Swo-1-11  UAUGCCCCUGGCCUUUGCUGGAGGCAUUUUUGUUUAUCCCAUAAAUGCAAUCAGGUACUCUGGCAAAAGAGUAUCUGCUGGUAGUAGUAGUUUUUAAGGGAGGUAGAGAUUGGUUAUGAAAUUUACUCGUCGUGGUUUCCUGAAGCUUUCAGGUCUCUCC
Dha-1-6   GAUAAGCCUAUCAGUAGGACCCGGUGGAGUGCUCCUGCUCUGAUUUUAAAGGAGGGUAAAUUAUGAUGAAAGAAUUCUUAAGCCGUAAAAGAACGCAGUUUUUUUCAGUUCUCAUGGUGGCGGUUUUGAUGAUCGCUUCCUUUGUUCCUUCAGCCUUCGC
Dha-2-5   UUUAAAUUUUAAAACUGUUAGGUGGUGAAUUUCAUUGGCAAGUAUAAAAAUGAAACAAGUCAUGGAAUUUUUUGCGGAGAACUAUGAUAUAAACCUCAAUGAUGCUGAAGAAUAUUUCAUGGCCAUUGUUUCUGUCGGAUUGUCUUUAAAUAAGGAAAUG
Dha-1-7   UUAAAUUUUAAAACUGUUAGGUGGUGAAUUUCAUUGGCAAGUAUAAAAAUGAAACAAGUCAUGGAAUUUUUUGCGGAGAACUAUGAUAUAAACCUCAAUGAUGCUGAAGAAUAUUUCAUGGCCAUUGUUUCUGUCGGAUUGUCUUUAAAUAAGGAAAUGC

          .......................................................................................................
          .......................................................................................................
          .......................................................................................................
```

```
Mth-1-4    GUGGUGCUAAAUGGCAGAGCGAGGAUAAGGAGGCAAAGCUAUGUGGGAAGUCUAUGAUGAAUUAAUCGCGGCGGUGCCGCCGGAUCUGGAGGUAGAAGAUUGCAUCGUCGGCCUUAACUGGAUCCUGGUCCGUUCCCGGGCUACCGGCCUGGUGAUGACG
Dha-2-6    UUAACGUUUAAAGAUCGGUUGGACUGGAGCCCGCCAACAUGGAGAAAGAAGGUUGGUCAACAAUGGGAUUGAAUUGUAUGAAAAUGAUGGGGAUACCGACUUUUGCGGAAAGACCCGAUGCCGAAUAUUUGUUUUUUCCCGGAUGUAAUCCCUUUGCUGA
Dha-2-7    GAUCGGUUGGACUGGAGCCCGCCAACAUGGAGAAAGAAGGUUGGUCAACAAUGGGAUUGAAUUGUAUGAAAAUGAUGGGGAUACCGACUUUUGCGGAAAGACCCGAUGCCGAAUAUUUGUUUUUUCCCGGAUGUAAUCCCUUUGCUGACAGGUCAAAAGG
Mth-1-5    UAAUUUGUUGCUGAUUAGCCAGGAAAGGGGAUAGCUUUAGAUGCAGUUCUUAGAAGGCAUCAGAGAAAAAUUUUACAGGCUGGUAAAAGACAGGCACCUGAGCGGUGAGGAAGAAAUCCAGGUGGUCAGCGCCCGGCCCCUGACCCCCGAAGAAGCCAUU
Cpe-2-1    UAAGGUCUAAAAGGACUAUUUAAUUUAAUGCUUAUUAUUCUUGUUGGUAUAACAAAAAUAAUAAGCAUUUUAUUUUAUAAAGGGAUGGUGAUAUUUUGAAAAAAUUUAUAGCUUUAGAAGAAGCUCUUGAAAUAUUAAAUAAGAACACUAAGGCUUUAAA
Cpe-1-1    UAAGGUCUAAAAAGACUAUUUAAUUUAAUGCUUAUUAUUCUUGUUGUUAUAACAAAAAUAAUAAGCAUUUUAUUUUAUAAAGGGAUGGUGAUAUUUUGAAAAAAUUUAUAGCUUUAGAAGAAGCUCUUGAAAUAUUAAAUAAGAACACUAAGGCUUUAAA
Cpe-3-1    UAAGGUCUAAAAGGACUAUUUAAUUUAAUGCUUAUUAUUCUUGUUGGUAUAACAAAAAUAAUAAGCAUUUUAUUUUAUAAAGGGAUGGUGAUAUUUUGAAAAAAUUUAUAGCUUUAGAAGAAGCUCUUGAAAUAUUAAAUAAGAACACUAAGGCUUUAAA
Dre-1-3    GUUUUGGGGGGUGAAAUACUAGCACAGCAAAAAAUGUUACAAAUUCAACACCGUAUAUUAAAUACAUAGGAGGUUUUAUCAUGAAGAAUCGGUCAUUAAAAUAUCUUGCCUUGCUGUUAACAAUGCUUAUGCUGGUAGUUGUCGGCUGUGCUCAGCAACA
Swo-1-12   UUAAUAUAAAACCAAACCUGGUAAACCUUUUUGCCAGGUAUUUUGUUUUUUGCAGCCAGUUUUAUUAUACAUUGCUGCGUUAAAAGCAAUAUUGUUACUUGAAAAACCGAACCCUUCAAAACCUUAGAAGGCCUCUGUGAAGAUCACUUUGAAAAGAGAU
Tet-1-2    AAUAGUUGGGGUGGGUAUUUUUUCUGAAUGUUGUUAUUGUAGGUCCUAAGAGUGAUCCUGCAAAGGUACAUCAAGCGGGUUCCCGCGGCGAGGGCUAAAGGUGGCUUGUUUCCGAAGGUAAUAUGAGGUUUCAAAUGCUUGUAGAAGAGCUGUGAGAAAC
Swo-1-13   UUUGUUUAAUUGAAUAGAACCGGCUCCAAGCCUUUGAGCCUAAUGACUGAAACUCAUUUGUCAGGGAGAAUCGGACUUUUAUGGUGGAAAAAGCCUGAAUCCCUGAGCCCGUUAAAAAAGUAUAAAAAAUUAUGGACGAUUAAUGCAUUUUAUACUUUCAU
Swo-1-14   UUAACUUAAUAUGUAUUCCGAGCCCUCUUAACCUGCAGACAAAUCCAGGUUACAGGGCCUAUAGGUAUAAGUGGAAACACUUAUGCCUCCCCGUAUUGUGGAAAGGAGUAGUGAAAGAACUGUUCAGUUAAUUGAACAGGUGUUACUGUGCUUUUUCACA
Swo-1-15   AUUAUUGGACUUUCUUCAAAUACGGGAGGCUCUUUUUGUCUGUAGGUUUGAUUUCUUGGGAGUUGUUAGAAUUUGAAAGGAGUGAUGGGAGUGGAACUGAUCAAAAUCACUAUUGAUGGAAAGGAGGUUGAAGUCCCGGCGGGUACAACAAUAAUGAAGG
Sfu-1-1    GGCCCCGCAGGGGCCGGAGCGGCGGGGUGGAUGGUCCGGGCGGGGAGGUUUGGGUGCCGCGCGGAGUCCCGCGGGUUUCCGCCGGGGAUGAAACGGCGUGCCGAUUGCGGCGGCGGUUCACGCGGAUCGCCGAGUGUCCUGAGACAGCCUGCGGAAUGCG
           ................................................................................................................................................................
           ................................................................................................................................................................
           ●●●●●●●●●●●●●●●●●●●●●●●●●●●●●●●●●●●●●●●●●●●●●●●●●●●●●●●●●●●●●●●●●●●●●●●●●●●●●●●●●●●●●●●●●●●●●●●●●●●●●●●●●●●●●●●●●●●●●●●●●●●●●●●●●●●●●●●●●●●●●●●●●●●●●●●●●●●
```

alignment positions 641 · · · 724

```
[1] Mth-1-1    AGCUGGCCUGCUCUUUCAAACAUACCGGCGAAUUCAAUCCUGCGGCAGCCCGGCUGACAGUGGCUGUUUUCCUUGAUGACGAUU
    Pca-1-1    CAGCUUAUCUACGGCGGUUCCGGCGAACUGUUCGGCAUGAUCGCCACCCGCAAAGCGGGUGACGUUUUCAUCCCCGGAGCCGA.
    Tth-1-1    CAAGGCCUACCCCGAGCUCUUGGAGCAGAGAGGCCUCGCCGAGCCCGGCACGCGCAAGCCCUACGCCCUCGGGCGGGUGGUCC.
    Tth-2-1    CAAGGCCUACCCCGAGCUCUUGGAGCAGAGAGGCCUCGCCGAGCCCGGCACGCGCAAGCCCUACGCCCUCGGGCGGGUGGUCC.
    Sth-1-1    CCGAGGAUCUGCCCCCCUUUCCCCGCAGCUCAGUGGACGGCUAUGCCGUUCUGUCGGCCGACACCUUCGGCGCGUCUGAAGGGC
    Ama-1-1    UUGUGUGAAAUCAUCGAAACCUGCAAGAAAGUCGAUGGCAUUAAAAAAGUCGCACUGACAACCAACGGUUUCAAACUCAAUCAG
    Dha-2-1    GUCCAGAGGUACAAAAAGGGUGAAAAGGUUUUUGAGGCGGGUCAGCCCGCUGUUUCCAUGGGUGUUAUUCUGCAGGGAAGGGUG
    Dha-1-1    GGUGCAGAGGUACAAAAAGGGUGAAAAAGUUUUUGAGGCGGGUCAACCCGCUGUUUCCAUGGGCGUUAUUCUGCAGGGAAGGGU
    Dha-1-2    GGAGAUUGGCAAAUGCCUGCUGACCAGGGUGCAGAGGUACAAAAAGGGUGAAAAAGUUUUUGAGGCGGGUCAACCCGCUGUUU.
    Aba-1-1    CGGUGGCGAGCCGCUUCUCCGCCGGGACCUGCUUGAACUCGUCCGCGAACUUCGUGCCUUGAAAACGCUCGAUGGUGAGCCCC.
    Dha-2-2    UCGGCAGCAGAAUGAUCCGUUCUCCCGGGAUGAGGCUUUGGCGAUCCUUUUCGACCAGGCCAAAUUUGAACUGGCCAAGGAAUU
    Dha-1-3    UUGGCGAUCCUUUUCGACCAGGCCAAAUUUGAACUGGCCAAGGAAUUCGUACCACUGAAUGAAGCUUUGGGCCGGGUGACUUCG
    Dre-1-1    CUAAUUUUUGCGCCUGGUCUGCUGGGAGGUACCAAUUGUGCUAACUCCGGACGUCUUUCCGUAGGAAGUAAGAGUCCUUUAACC
    Dha-2-3    GCUGUGGCCGGUCCGGGGCUUUCUUUCAGUGAAACGAAAGAGGGAAAGGUCAAUCUGAUUGCUGAAGAGGAUGGUUUAAUCUA.
    Pin-1-1    GCAAAGAUUUUCCGAAAAUAAUUGAAGCCAUUGCCACUAUCCCUGGCAUCCAAAAAGUAGCCACUACGACUAACGGUUACAAUU
    Rsp-1-1    CCCGAAAUCAUUUACCGAUUGAAUCGCAUAGACGGCAUCAACAACAUCGCUGUGACCACCAAUGGCUACAAGUUGCCACAGCGU
    Cps-1-1    GAAAUCAUUCGAAUUUGUAAAGAAACAGCUGGCAUCAAAAAAGUUGCCAUUACAAGUAAUGGUUUUAAGUUACCAGAUCACCU.
    Hdu-1-1    GUGGUUAAAUCCGGUCCGGCAAUGCCAGUUUCUGUUGAAAAUGAAUUACAAACAGUUGUGCCAGAUCAAGUGCAUGGCGAAACA
    Apl-1-1    GUGGUUAGAUCCGGUCCUGCUAUGCCUCCGUCAAUCGAAAACGAAUUACAAACUGUCGUGCCUGAUCAAGUACACGGUGAAACU
[2] Hdu-1-2    GAAAAAAUCCGAAUCACGGGUGGGGAGCCUACUUUACGUAAAGAUUUUCUAGAAAUAGUCGAAACCAUUUCACAAACAGAAGGC
    Mha-1-1    ACGGAAAAAAUCCGUAUCACCGGUGGUGAGCCAACUUUACGCAAAGAUUUCCUCGAAAUCGCUCACACUAUUUCACAAACACCC
    Apl-1-2    GAAAAAGUGCGCAUUACCGGUGGCGAACCGACUUUACGUAAAGAUUUUUUAGAAAUUGCGCAUACCGUUUCGCAAACGAACGGC
    Msu-1-1    CGUUUAACCGGCGGCGAACCGACGUUACGCAAAGAUUUUUUAGCUAUUGUUGAAACCAUCAGCGCCCUAGAAGGCAUUAAAAAA
    Hso-1-1    UUCGUAUUACCGGUGGUGAGCCGACUCUUCGUAAAGAUUUCCUGUCCAUUGUUGAAAAUAUUCGUGCUAUUGAAACAAUAAAA.
    Hso-2-1    UUCGUAUUACCGGUGGUGAGCCAACUCUUCGUAAAGAUUUCCUGUCCAUUGUUGAAAAUAUUCGUGCUAUUGAAACAAUAAAA.
    Hin-3-1    CGUUUAACGGGUGGCGAACCGACUUUACGCAAAGAUUUUAUUUCUAUUGCUGAAAGCAUUGCUAAUAUUGAUGGCAUUCGUCAA
    Hin-4-1    CGUUUAACGGGUGGCGAACCGACUUUACGCAAAGAUUUUAUUUCUAUUGCUGAAAGCAUUACUAACAUUGAUGGCAUUCGUCAA
    Hin-2-1    CGUUUAACAGGUGGCGAACCAACUUUACGUAAAGAUUUUAUUUCCAUUGCUGAAAGCAUUGCUAACAUUGAGGGCAUUCGUCAA
    Hin-1-1    CGUUUAACGGGUGGCGAACCGACUUUACGCAAAGAUUUUAUUUCUAUUGCCGAAAGCAUUGCUAACAUUGAUGGCAUUCGUCAA
    Pmu-1-1    CGUAUUACGGGGGGAGAACCUACCUUACGUAAAGAUUUUUUACCUAUUGUUGAAACCAUUGCUCAAAAUCCUACUAUUAAACAG
    Asu-1-1    AUUACCGGCGGCGAGCCUACUUUACGCAAAGAUUUCCUGCAAAUUACGGAAAAUAUCACCGCACUUGACGGUAUUCGACAUGUC
    Hso-2-2    UGUUGCGGCUGAACAACAUACCGUUGUAACAGCUGCUCAUUGGGGGGCUUUAGGUGUAGUUGUAGAAAAUGGCAAGGUAGUUAA
    Hso-1-2    UGUGGCGGCUGAACAACAUACCGUUGUAACAGCUGCUCAUUGGGGGGCUUUAGGUGUAGUUGUAGAAAAUGGCAAGGUAGUUAA
    Mha-1-2    GAAAAUGGCAAAGUGGUGAAAUCUGAUUCUGUUUUUGAGAUUUCUGUGCCAAAUGAAUUGCAACACGUGGUAGCAGACCAAGUA
    Dha-2-4    UUGCGUGUCAUGCGGAUUAGCACCACAUUGGGCUUUAGACGGUUCCGCAUCACCGGCGGGGAACCCUUAAUCAGGGCCGGAGU.
    Dha-1-5    UUGCGUGUCAUGCGCAUUAGCACCACAUUGGGCUUUAGACGGUUCCGCAUCACCGGCGGGGAACCCUUAAUCAGGUCCGGAGU.
    Csp-1-1    ACCACACAGGAAUCAAAUGGAAGCAAUGUACCAAGUGCCACUGUUGAAACGACCCCUAUAAAAAAUAAUGUAUUGAUUCUUUCA
    Dps-1-1    AGGAUAACCGGUGGAGAACCCUUUGUUCGCCGGGACUGUGUUGAUUUUAUGCAGGCCCUGAUGGAGCAAACAGCGCAUUUGGA.
    Mth-1-2    AGAAGCAGCGGACGGCCACGGCGGCAAGCUGCCCUGCGCCGUAGCCCAGGAGGUUGCCCGCCGCCUUAAGGUACCAAUGCGGGA

               ....................................................................................
               ....................................................................................
               ●●●●●●●●●●●●●●●●●●●●●●●●●●●●●●●●●●●●●●●●●●●●●●●●●●●●●●●●●●●●●●●●●●●●●●●●●●●●●●●●●●●○
```

```
Ame-1-1    GUACCGUUAUUUAAAGGGGGGGGGAGGGUCCUUGCUACGGACAUCACAGCACCUAUCGAUGUGCCGGGAUUUGACCGCUCUACU
Ame-1-2    AAAAAACACAUCAAGAUAUAUUAACCUUGGAAGAAAUCGAACAAAUCGUCAGAAUAGGUGCGGAAAAUGGUAUUAAUAAAGUAC
Ahy-1-1    CCUCCCUGCGCCGGGACUUCACCGCCAUCAUAGAGACGGUGGCCAAUACCCCCGGCAUCGAGAAGGUGGCCAUGACCACCAAUG
env-1      CCUCCCUGCGGCGGGACUUCACCGCCAUCAUCGAGACGGUGGCCAAUACCCCCGGCAUCGAGAAGGUGGCCAUGACCACCAAUG
Plu-1-1    CGAUUUCACCGAUAUUAUUUCCGUCAUACGUGAAAAUAAGUUGAUUAAAACCCUCGCGGUAACCACUAACGGUUAUCGAAUGGA
Sgl-1-1    CGCCGUGAUUUCAUCAACAUCAUUGCGGCGGUGCGCGCCAAUCUGGCUAUCCGCACCGUCGCGGUCACCACCAACGGAUAUCG.
Ymo-1-1    UUUCACCGACAUCAUCGCCACCAUUCGGCAAAAUCCCGCGAUCCGCACAUUGGCGGUGACCACCAAUGGUUACCGUUUAGCGCG
Yps-2-1    CUUCACUGAUAUUAUUGCCACUAUCCGGCAAAAUCCCGCUAUCCGUACCUUGGCCGUUACAACUAAUGGUUAUCGUUUAGUUCG
Yps-1-1    CUUCACUGAUAUUAUUGCCACUAUCCGGCAAAAUCCCGCUAUCCGUACCUUGGCCGUUACAACUAAUGGUUAUCGUUUAGUUCG
Yfr-1-1    UUUUACCGAUAUUAUUGCCACAAUCCGGCAAAAUCCUGCGAUCCGUACCUUGGCAGUGACUACCAAUGGCUACCGUUUAGCGCG
Yin-1-1    CUUCAGCGAUAUCAUUGCCACCAUCAGACAAAAUCCAGCGAUCCGCACUCUGGCAGUCACCACCAAUGGCUAUCGUUUAGCCCG
Ybe-1-1    UUUUACCGAUAUUAUCGCCACUAUCCGGCAAAAUCCCGCGAUCCGCACAUUAGCGGUGACCACCAAUGGUUAUCGUUUAGCGCG
Spr-1-1    UCGCGAUUUUACCGAGAUCAUUGCCGCCGUGCGUGAAAAUCCGGCCGUUCGCCAGCUUGCCGUGACCACCAAUGGCUACCGCCU
Eca-1-1    GCGUCGUGACUUCGUCGAUAUCAUUGCUGCGAUCCGUGAAAACCCAGCUAUUCGUACGCUGGCGGUCACCACCAACGGUUACC.
Esp-1-1    UUCGUCGUGACUUUCCUGACAUUAUUGCGGCUAUUCGUGAAAACGAGAGCAUUCGUCAAAUCGCCGUCACCACCAAUGGCUAUC
env-2      UGCGUCGCGACUUUACUGACAUUAUCGCCGCCGUGCGCGAAAACGACGCCAUCCGUCAGAUUGCGGUAACCACCAAUGGUUAUC
Eco-17-1   UACGCCGCGACUUUACCGAUAUCAUCGCCGCUGUGCGGGAAAACGACGCUAUCCGCCAGAUUGCGGUCACAACCAAUGGUUACC
Eco-6-1    UACGCCGCGACUUUACCGAUAUCAUCGCCGCUGUGCGGGAAAACGACGCUAUCCGCCAGAUUGCGGUCACCACCAAUGGUUACC
Eco-3-1    UACGCCGCGACUUUACCGAUAUCAUCGCCGCUGUGCGGGAAAACGACGCUAUCCGCCAGAUUGCGGUCACAACCAAUGGUUACC
Eco-1-1    UACGCCGCGACUUUACCGAUAUCAUCGCCGCUGUGCGAGAAAAUGACGCUAUCCGCCAGAUUGCGGUCACCACCAAUGGUUACC
Sbo-2-1    UACGCCGCGACUUUACCGAUAUCAUCGCCGCUGUGCGGGAAAACGACGCUAUCCGCCAGAUUGCGGUCACCACCAAUGGUUACC
Sso-1-1    UACGCCGCGACUUUACCGAUAUCAUCGCCGCUGUGCGGGAAAACGACGCUAUCCGCCAGAUUGCGGUCACCACCAAUGGUUACC
Sfl-3-1    UACGCCGCGACUUUACCGAUAUCAUCGCCGCUGUGCGGGAAAACGACGCUAUCCGCCAGAUUGCGGUCACCACCAAUGGUUACC
Eco-2-1    UACGCCGCGACUUUACCGAUAUCAUCGCCGCUGUGCGAGAAAACGACGCUAUUCGCCAGAUAGCGGUCACCACCAAUGGUUACC
Sfl-2-1    UACGCCGCGACUUUACCGAUAUCAUCGCCGCUGUGCGGGAAAACGACGCUAUCCGCCAGAUUGCGGUCACCACCAAUGGUUACC
Sdy-1-1    UACGCCGCGACUUUACCGAUAUCAUCGCCGCUGUGCGAGAAAACGACGCUAUCCGCCAGAUUGCGGUCACCACCAAUGGUUACC
Sbo-1-1    UACGCCGCGACUUUACCGAUAUCAUCGCCGCUGUGCGGGAAAACGACGCUAUCCGCCAGAUUGCGGUCACCACCAAUGGUUACC
Eco-15-1   UACGCCGCGACUUUACCGAUAUCAUCGCCGCUGUGCGAGAAAACGACGCUAUUCGCCAGAUAGCGGUCACCACCAAUGGUUACC
Eco-14-1   UACGCCGCGACUUUACCGAUAUCAUCGCCGCUGUGCGAGAAAACGACGCUAUUCGCCAGAUAGCGGUCACCACCAAUGGUUACC
Sty-1-1    UACGCCGCGAUUUUACCGACAUCAUUGCCGCCGUGGGUGAAAAUGAUGCUAUUCGCCAGAUCGCGGUGACCACUAACGGUUAUC
Sen-1-1    UACGCCGCGAUUUUACCGACAUCAUUGCCGCCGUGGGUGAAAAUGAUGCUAUUCGCCAGAUCGCGGUGACCACUAACGGUUAUC
Sen-4-1    UACGCCGCGAUUUUACCGACAUCAUUGCCGCCGUGGGUGAAAAUGAUGCUAUUCGCCAGAUCGCGGUGACCACUAACGGUUAUC
Sen-2-1    UACGCCGCGAUUUUACCGACAUCAUUGCCGCCGUGGGUGAAAAUGAUGCUAUUCGCCAGAUCGCGGUGACCACUAACGGUUAUC
Sam-1-1    AUUUUACCGAUAUCAUCCGGGUCAUCGCAGACAACCAGGCCAUCAGUACCCUGGCCACCACCACCAACGGUUAUCGCCUGGAGC
Spe-1-1    AUUUCACCGACAUCAUUCGCAUCGUUAAAGACAACGAUAAGAUUAAAACCAUUGCCACGACUACUAACGGUUACCGUUUAGCUA
Ssp-4-1    AUUUUACCGACAUCGUGCGUAUCGUCGCGGACAACGACAAGAUCAAGACGCUGGCGACCACCACCAAUGGCUAUCGCCUCGAGA
Swo-2-1    ACUUUACCGAUAUUAUUCGUGCGGUUGCUGAUAACGAUAAGAUUAAAACCAUCGCGACAACGACAAACGGUUACCGUUUAGAGA
Ssp-5-1    ................................................................................
Ssp-5-2    AUUUUACCGACAUUAUUCGUGUUGUGGCCGAUAACCCGCGCAUUCACACUGUUGCGACUACCACUAAUGGGUAUAGACUCGAAA
Spu-1-1    AUUUUACCGACAUUAUUCGUGUUGUGGCCGAUAACCCGCGUAUUCACACUGUUGCGACUACCACUAAUGGGUAUAGGCUCGAAA
Sba-1-1    ACUUUACCGACAUCAUCCGUGUCGUCGCCGAUAACCCGCGCAUUCACACCAUAGCGACAACCACUAAUGGCUAUAGACUCGAAA
Sba-2-1    ACUUUACCGACAUCAUCCGUGUCGUCGCCGAUAACCCGCGCAUUCACACCAUAGCGACAACCACUAAUGGCUAUAGACUCGAAA
Ssp-1-1    AUUUUACCGAUAUCAUUCGAGUGGUCGCCGACAAUCCUCGUAUUCAUACCGUAGCAACUACCACUAAUGGGUAUAGGCUCGAGA
env-4      AUUUUACCGAUAUCAUUCGAGUGGUCGCCGACAAUCCUCGUAUUCAUACCGUAGCCACUACCACUAAUGGGUAUAGGCUCGAGA
Ssp-2-1    AUUUUACCGAUAUCAUUCGAGUGGUCGCCGACAAUCCUCGAAUUCAUACCGUAGCCACUACUACUAAUGGGUAUAGGCUCGAGA
Ssp-3-1    AUUUUACCGAUAUCAUUCGAGUGGUCGCCGACAAUCCUCGAAUUCAUACCGUAGCCACUACUACUAAUGGGUAUAGGCUCGAGA
Son-1-1    AUUUUACCGAUAUUAUUCGAGUCGUUGCCGAUAAUCCGCGUAUUCAUACUGUUGCUACUACCACUAAUGGAUAUAGGCUCGAGA
env-5      AUUUUACCGAUAUCAUUCGAGUGGUCGCCGAUAAUCCGCGUAUUCAUACCGUAGCCACUACCACUAAUGGGUAUAGGCUCGAGA
Sfr-1-1    AUUUCACAGAUAUUGUGCGGAUCGUUGCCGACAAUCCUAAUAUUAAUACCGUAGCGAUGACCACCAAUGGGUAUCGUUUACAGC
Sde-1-1    ACUUUAUCGACAUUAUCGGCCUUGCUGCUAAUACCCCCAAUAUCAAGACUGUCGCCACCACCACCAAUGGUUACCGCUUAGCGA
Vch-3-1    CUGCGCAAAGAUUUUGGCGAAAUCAUUCAUACCAUUGCGCAAACUCCCGGCAUUCAGAAAGUCGCUACCACCACCAACGGCUAU
Vch-1-1    CUGCGCAAAGAUUUUGGCGAAAUCAUUCAUACCAUAGCGCAAACUCCCGGCAUUCAAAAAGUUGCGACGACCACCAAUGGCUAC
Vch-8-1    CUGCGCAAAGAUUUUGGCGAAAUCAUUCAUACCAUAGCGCAAACUCCCGGCAUUCAAAAAGUCGCGACGACCACCAAUGGCUAC
Vch-10-1   CUGCGCAAAGAUUUUGGCGAAAUCAUUCAUACCAUAGCGCAAACUCCCGGCAUUCAAAAAGUUGCGACGACCACCAAUGGCUAC
Vch-13-1   CUGCGCAAAGAUUUUGGCGAAAUCAUUCAUACCAUAGCGCAAACUCCCGGCAUUCAAAAAGUUGCGACGACCACCAAUGGCUAC
Vch-7-1    CUGCGCAAAGAUUUUGGCGAGAUUAUUCAUGCCAUAGCGCAAACUCCCGGCAUUCAAAAAGUUGCGACGACCACCAAUGGCUAC
Vch-12-1   CUGCGCAAAGAUUUUGGCGAAAUCAUUCAUACCAUAGCGCAAACUCCCGGCAUUCAAAAAGUUGCGACGACCACCAAUGGCUAC
Vvu-1-1    CUGCGUAAAGAUUUUACUGACAUUAUCCACACGGUCGCAUCAACUCAAGGCAUUAAGCGAGUUGCCACUACCACCAAUGGCUAU
Vvu-2-1    CUGCGUAAAGAUUUUACUGACAUUAUCCACACGGUCGCGUCAACUCAAGGCAUUAAACGAGUUGCCACUACCACCAAUGGCUAU
Vsp-3-1    CUGCGUAAAGACUUUCCUGAAAUCAUACAUACUGUUGCUUCUACUCCAGGCAUUGAAAAAGUAGCCACCACAACGAAUGGCUAC
Vsp-2-1    CUGCGUAAAGACUUUCCUGAAAUCAUACAUACCGUUGCUUCUACUCCAGGCAUUGAAAAAGUAGCCACCACAACGAAUGGCUAC

           ................................................................................
           ................................................................................
```

```
Vpa-1-1     UUGCGCAAAGAUUUCACUGACAUCAUUCAUUCUGUCGCAACGACGCCAGGCAUCAAAAAGGUGGCGACGACGACCAACGGCUAC
Vsp-1-1     UUACGCAAAGAUUUUACCGACAUUAUCCACUCAGUGGCGACGACGCCUGGUAUCCAAAAAGUUGCAACUACGACCAAUGGUUAC
Val-1-1     UUACGCAAAGAUUUUACCGACAUUAUCCAUUCAGUAGCGGCGACGCCUGGCAUUCAAAAAGUUGCAACGACAACCAAUGGCUAU
Vfi-1-1     CUUCGCAAAGAUUUCACUGAUAUUAUUCGUACUGUCGCCUCUCAGCCUGGUAUUACUAAGGUUGCAACUACUACCAACGGUUAU
Psp-1-1     UUACGUCGUGAUUUUACUGAUAUCAUCAAAACGGUGGCUGAGAUCCCUGGGAUUAAUAAAGUAGCGACAACCACUAAUGGUUAU
Van-1-1     UUACGCCGUGAUUUUACUGAUAUUAUCAAAACGGUAGCUGAAAUCCCUGGGAUCAAUAAAGUAGCGACAACCACUAAUGGUUAC
Ppr-3-1     ACGGGUGGCGAACCUAGUUUACGUCGUGAUUAUACGGAUAUUAUCCGUAUAGUCGCGGCUCAGCCCGGCAUAACUAAAGUGGCU
Ppr-2-1     ACUGGGGGCGAACCUAGUUUACGUCGUGACUAUACGGAUAUUAUCCGUACUGUAGCAGCUCAGCCCGGUAUAACUAAAGUGGCU
Dre-1-2     GUUAUCUCAAGUCCAUGGCGGACAGUUUCGCCUGGAAGGUGAGGAUAUUUUGUUAAAGGAAGGUUACCCCCUAGAAGUUCUCAU
Swo-1-1     GAGGAGAAGCAAAAGGGUUUUCGGGCAAGAAUGAUUUUGAAACCAUGGGAGGGUUUAUUGCUAUGGAAGCAGCCGUAAGCAGUU
Pat-1-1     UAUAACGGGUGGCGAGCCUUCGCUACGAAAAGAUUUGCCAGACGCUAUUCGUGCCUGCGCAAAUACCCCGGGCAUCAAGCAGGU
Pat-1-2     AUUUGCCAGACGCUAUUCGUGCCUGCGCAAAUACCCCGGGCAUCAAGCAGGUCGCGAUCACGACGAAUGGUUAUAAAUUACCUG
Swo-1-2     UGGAAGGCACCCCCUGUUUCAGUGUGGGGGUUUUUUUGUUGCCUAAAAAAAAUGGAAGGAGUGAUGAUGGUGGAUUUGGUAAAAC
env-6       ....................................................................................
env-7       UUGAUUCCCUUCAUUGAGGGGCUGACUAAAGCCGGUUUGGAAGACAUCGGUCUGACCAGCAACGGACUUUUGCUAGCAAAAUCU
Swo-1-3     UGGGCUGCUUUUAAGCUGGACAAGCUUUUUCCCUGCAGGGAGGUGGGAAAAUAUGCUGGAUAAACACGAAAGAAAUAUAAACU.
Swo-1-4     UGGGCUGCUUUUAAGCUGGACAAGCUUUUUCCCUGCAGGGAGGUGGGAAAAUAUGCUGGAUAAACACGAAAGAAAUAUAAACUA
Chy-1-1     AAAAAGUACGGAUUACCGGCGGCGAGCCAUUGGUUAGAAAAAAUCUUUUAUAUUUAAUUGAAAAUAUUGCAGCUUUACCGGGUA
Swo-1-5     AGAUACUUUGAGAUAGGAGGAAGAUAUAUGAAAAAGAAGGAGCUUGUAAUUCUGGCGAUGCUUGCCCUGCUGAUCAUCAGUGGC
Mth-1-3     AAUAGCGAGUACCCUGAUUGCCGCCCUGGCCCUGGCCGGCUGCCGCCAGGUUGCAACACCGCAGCCCCGGCAACAGGGGCAACA
Swo-1-6     AAGAUUGGGGGAUUAAAAUAUGCUUUGUAUAUUUCAAAAUGGGGAAUAUUAGUUCCUCUUUUUUAGUUUAUAGCCAUAUCGUA
Csa-1-1     UGGAUUUUUAAGUUUUGUUGUUCCUGAGUUUGAAAAAAAGAAUAAUAUGAAACUUAAUUUUAUCUCUGUUGGCAGUGGUCAGGC
Dac-1-1     AGCGGCACCGCUGCGGCCCAAUCCGGCCGGAUGUCCUGCGGCGCUAAAAGUCCGCUGACCGGCGGCAUCAAAGAGAGUAAUGCC
Dac-1-2     CUCUGCUGCCGACCUUUGAAGCGCAGAACAACUGCAAGGUCGACGUGAUUGCCGUGGGUACCGGCAAGGCAAUCAAGCUGGGUA
Hne-1-1     CCGACUGUCCGGAAGGAUCUGACGCAACUGAUCGCCCGCAUUUCAGCGACGCCGGGGGUUGAGAAAGUCGCCCUGACGACAAAU
Swo-1-7     GUCUGGAGGCGGGCACGAUGCCCCUCUGGGGUUAGCUUCUUCCAACCCUGCUGAUGGGGCAUCAAAUGUAGCAGUUAAUACUCA
Dac-1-3     UAGUGGUACCGCUGCGGCCCAAUCCGGUCGUAUGUCCUGCGGCGCUAAAAGUCCGCUGACCGGCGGCAUCAAAGAGAGUAAUGC
Gsp-1-1     AACAGCGGGCGCUUUUCCGCAGGCGCCAAAAGCCCCCUCACCGGCACUAUCAAGGAAAGCAAUGCCGGCGGGUUGGCCGCCCA.
Gsp-1-2     AAGAGGAUUACAAGGAAGUAACCAGGAGCAAGGCGCUGAAUUACAAGCCGGGCAAGGGGUUCACCCUGACCUCUGCCGACGAAA
Gsp-1-3     ACCGUCUGGACUUCCAUUUACGUAUCCAUAUGGUCAACGCUUUUUGCAUCGGUCAUCGGCAUCCCUGCCGGUAUUACUAUCGGC
Pca-1-2     CGGGCACCCCGGCCGCAAACUCCGGUCGCAUGUCCUGCGGCGGUAAAAGCCCUCUGACCUACGGCAUCAAAGAGUCCAAUGCCG
Gsp-1-4     UGUUCCUGUAUCCAAAGGCCUCUUUCCAUCUGCGGACAGACUCUUUGAUGCAUUUGGGGGAGACCUUUUCCCCUGUUAUUAGAA
Ppr-1-1     ACUCCGGCCGCCAACUCCGGUCGCCUCUCCGCCGGCGCCAAGAGCCCCCUCACCUACACCAUCAAGGAGAGUAACGCCGGUGGG
Gur-1-1     GGCAUGCUGAGCGGCACCACCGGCUCCAUGACCGGCCGGCUGUCGGUCGGCUGCAAGAGCCCGCUGACCGGCACCAUCAAGGAA
Gur-1-2     UCUGGUAAGGCAUGCGGCCAUGCCGUCGUGGGGGAUAGGAAGCGUAGUGAAUCCGGCCAAGGAGGGAAACCUCCUGAUUCGGUU
Gsu-1-1     UACCGGUUCGACGCUGGUGCGAUCGAGGAACUGGAGGCAAUCUCCGCCGAGCUGCGCCAGCAGUGGCAACUGGAAGACGUCCUU
Gme-1-1     AAAGAAAAGGGUGUCAUCACCGAAGCCGAUUACAAGGAAGUGACCAAGAGCAAAGCGUUCGAUUACAAGGUUGGAAACGGCUUC
Gur-1-3     CCACAUCAUGUAUCGAUUAUCGCUCGGAAGGCGACACCAAGGCCGAGUUGGGGAUUAUUUCGGGGAACUGAAGGAAAAAUGG.
Pca-1-3     UGUCCGGCACCCCGGCCGCUAACUCCGGCCGUAUGUCCGCCGGCGCCAAGAGCCCCCUGACCGGUACCAUCAAGGAGUCCAACU
Gme-1-2     GGAACGCCGGCCGCCAACUCGGGCCGCCUCUCGGCCGGGGCCAAGAGCCCCCUGACCGGCACCAUCAAGGAAAGCAACGCCGGA
Pca-1-4     GUGGCUUGGAUACCUGUUGUCAGCCCGGGGCUCGCCGUUUUCGAAUUUUCCGCUUAGUUUUUUUGUGGGUUAUGGAAUAUCCU
Ppr-1-2     CGGAACCCCGGCCGCCAACUCCGGUCGCCUCUCCGCCGGUGCCAAGAGUCCGCUCACCGGCACCAUCAAGGAGAGUAAUGCCG.
Pca-1-5     UGCAUGACCGGUCCUUAUGUCGGUACCAAGGUGGCUCAGUCGGGUCGUUGGGAGCUGAUCACUAAAGGUCCUCUGACCGGGAUU
Gur-1-4     CGGCACCGCCGCUGCCAACUCCGGCCGUCUCUCCGCCGGGGCCAAGAGCCCGCUCACCGGUGGCAUCAAGGAAAGCAACGCCGG
Gsp-1-5     CGGUACCCCUGCCGCCAACUCAGGCCGCCUCUCUGCAGGGGCCAAGAGCCCGCUUACGGGCACCAUCAAAGAAUCGAACGCCG.
Gme-1-3     UAAUGAGAGAGCAUCUGGGACGUCCCGGGGCCUGCAUCGGGGAAGCGUGCGAGGGAUUCUGCCAGCAUUCGCUACAGACCUGC.
Gme-1-4     CAGCGACGCGGAGGUAUGUCCGCGACGUUCAGGGGCCUUUCUAUCCAGAGAGCGCCCCUGUUGUUUUUUGCGGGAUAGACCG.
Csp-2-1     CGACCUGGUCGAGAUCAUCGCCCGCGCCGCCGCCACGCCGGGAAUCGACAAGGUGGCGGUCACCACCAAUGGCUGGAACCUCAA
env-8       CCUUUGCAAAUAUCAUCACCGAUACACUUCAUAUUCCCGGCGGCAUUGGUACCAGCUUCUCCCUGAUGUUUUUGGUAAUUGCGG
Tet-1-1     UCAAAGAAGUUUUUGAUGAUCCUUUUAUAUUCUAUUUUGUUGUUUAGUUUAUUUUCUCUGAACGGUUGUACAGGUUCUAAAACU
env-9       UAACCGAAGU..........................................................................
env-10      ....................................................................................
Swo-1-8     AGGACACGACCUUCUUGUUUAAGAAGGAAAAAAGCGGGAUUCGUUCUGGUUCUCCCUAAGGUUUUGGGAAGCAAACGGGUACAG
Swo-1-9     GAGGCUUUAAUUCUGUUACUAUAGCUCUAUUAUUAUUCUAUUCGCUGGGAAUCCUCAUGCCGUAGAAGGAACGCCAUACGAAGA
Swo-1-10    GGGUUUUUUUUUAAAUCAACCCAUGAAGGUUUAAUAUAUUUUCUUUGUCUUACGAUAGAACCAUCCAUAGUUACUUAAGUGCUG
Swo-1-11    GUUGCCGUAGUUGCUUCCGGCUUGGGGUUUGACUUGGAAGCAACUGCCGCACACGGGCACGCACUGAAGCUGGCAGGAUGCAC.
Dha-1-6     GCUGAAUUACAACGGCACAGGCUCCGGUACGGGCAAUAAGUCCUUGGAUUUUAACGGGUAUGAAUAUACCAAAACCGGCGGUAC
Dha-2-5     AGAAAAGAUGUAGCAGCAAUCUAUAAUCAGAACAAAUUAAUGUUUUUUGAAAGCGCUAAAGAAUUUUCAGGAUAUAAUCAUGUC
Dha-1-7     GAAAAGAUGUAGCUGCAAUCUAUAAUCAGAACAAAUUAAUGUUUUUUGAAAGCACUAAAGAAUUUUCAGGAUAUAAUCAUGUUU
Mth-1-4     CCCCUGGAGGGCCAUCCGCGCAUCAAGCUGGCCGGGGAAAUAAAGGGCAUGCCCGUGCGGGAUCUGGCGGAGUACAUUAAGUCC
Dha-2-6     CAGGUCAAAAGGGGAAUGGAAUAUGGUCUGUAUGCAGUUGCUUAGGGAUAGAAAAAUUGAUUUUGCCGUUCUGGGUAAAGAUCA

            ....................................................................................
            ....................................................................................
            ●●●●●●●●●●●●●●●●●●●●●●●●●●●●●●●●●●●●●●●●●●●●●●●●●●●●●●●●●●●●●●●●●●●●●●●●●●●●●●●●●○
```

```
Dha-2-7    GGAAUGGAAUAUGGUCUGUAUGCAGUUGCUUAGGGAUAGAAAAAUUGAUUUUGCCGUUCUGGGUAAAGAUCAGUGGUGUUGUG.
Mth-1-5    GGCCGGCCGGAACGUCAGGACUUCCCUCUCUUGAAAGGUAAAGAAGUGAUGCUCCAGGCCGCCUUUGCUGGCGCGGUGGGGCAG
Cpe-2-1    AAGUGAGGUUGUUAGCAUAAAGGAUUCUUUAAAGAGAGUUUUGUAUGGUGAUGUUAAAUCUAAGAUUAAUAAUCCUCCCUUUAA
Cpe-1-1    AAGUGAGGUUGUUAGCAUAAAGGAUUCUUUAAAGAGAGUUUUGUAUGGUGAUGUUAAAUCUAAGAUUAAUAAUCCUCCCUUUAA
Cpe-3-1    AAGUGAGGUUGUUAGCAUAAAGGAUUCUUUAAAGAGAGUUUUGUAUGGUGAUGUUAAAUCUAAGAUUAAUAAUCCUCCCUUUAA
Dre-1-3    AACCCCUAAGGAAGAAGUGAAGAAGGAAGCCGCAGAACCUGCAAUAAAAGAUGUUAUUCUAGCCACAACCACCAGUACCCAAG.
Swo-1-12   GUUUUACGUAGAAUAAUAUGGAAAAGAGUAAAGCGAGUAUUAUAGCGAGUAUUAUUUAGAAAAAAGAUAGGAGGGAUCAUGCUG
Tet-1-2    CUGAACUUGCGAGAAUGUUUUAUAAUAGUAAAAGUGGCUUUCUACAGAUUUAGAGCAUAGUCGCAGAAAGGGCAACGAUAAGUA
Swo-1-13   UUUUGCUGGGAUUGAGUCUUUAAUCCAUGGUUUAACGGCCGCGGUAUAAGUGGAAACGCUUGUGCCUCCCUUAUUUGGAAAGGA
Swo-1-14   ACACACAACACCUGUUUUUUGUUUAAUUGAAUAGAACCGGCUCCAAGCCUUUGAGCCUAAUGACUGAAACUCAUUUGUCAGGGA
Swo-1-15   CCGCUGACAGUGUUGGGAUUAAUAUUCCAAGGCUAUGUUAUGACCCUGACCUCAGCGCCCUGGGAGCUUGUCGCCUGUGCGUGG
Sfu-1-1    AUACGGAAGGUCACCAUUCACCAUAAAGGAGAGGAGCCAUGUUGAGAAAAUCGGUUUUGGCUGGUGCGGUGCUCGUUGCCCUGG
           ....................................................................................
           ....................................................................................
           ●●●●●●●●●●●●●●●●●●●●●●●●●●●●●●●●●●●●●●●●●●●●●●●●●●●●●●●●●●●●●●●●●●●●●●●●●●●●●●●●●○
```

Supplementary Figure S5: alignment of sequences containing P3

The format of this figure is identical to Supplementary Figure S4.

alignment positions 1 · · · 160

```
Aba-1-1   CUAGGAACGUGUUUC.CGACGUU.CG.CU.GCCUACGG........................AAUA..............................................................................................
Tth-1-1   GGAUGGCCUCGCCUC.CGAGCCC.GG.GU.AG....ACCC......................CCU...............................................................................................
Tth-2-1   GGAUGGCCUUGCCUC.CGAGCCC.GG.GU.AG....ACCC......................CCU...............................................................................................
Sth-1-1   GAUGUGAUCACGUCC.CGAGUCA.UG.CG.ACCUGUACCC......................GAUG..............................................................................................
Csp-1-1   CGAUGAAACUCGCUC.CGAGCGU.CC.CG.GCCUAACGCGC.....................AUCC..............................................................................................
Rsp-1-1   CAUUAAGCAAUACAC.CGAGUGA.CA.GGGCGCUAAGUGG......................UCU...............................................................................................
Ama-1-1   AGGUAACAAAAACUC.CGAGCUC.UA.AU.ACCUAAAGUU......................CGAAC.............................................................................................
Cps-1-1   UUUAUUUUAUUACAC.CGAGCUA.UC.AC.AGCUAACAUUGUUUU.AUUUUUUGGCU.....AAAUUA............................................................................................
Dha-2-1   UAUGUUUAUGUGCUC.CGAAACU.GG.AA.ACCUAAAGU.......................UA................................................................................................
Dha-1-1   UAUGUUUAUGUGCUC.CGAAACU.GG.AA.ACCUAAAGU.......................UA................................................................................................
Dha-2-2   AUAAUAGGGAAUGGC.CGAGCCA.UA.UU.UCCUACUGA.......................CUGAAGCAAGUCUGACGCUA..............................................................................
Pat-1-1   CAGGUGGUUUAACUC.CGAGCCU.UC.AU.ACCUAAAUC.......................AGAA..............................................................................................
Pat-1-2   CAGGUGGUUUAACUC.CGAGCCU.UC.AU.ACCUAAAUC.......................AGAA..............................................................................................
Swo-1-1   AAUAGUUAAUGUCUU.CGAGCCU.UU.UA.UCCUAUAGCUU.....................U.................................................................................................
Mth-1-1   AACUCUAUAAACCUC.CGAGCCA.GU.GG.GCCUAAGGUU......................UUC...............................................................................................
Mth-1-2   UUGAAUAGUCAUCUC.CGAGCCG.GU.GC.UCCUAAGGGC......................UGUC..............................................................................................
Dha-1-3   AAUAUAAUAUAUCUC.CAAGCUG.GU.GAGACCUAAGGG.......................AAU...............................................................................................
Mth-1-3   AAAUAUUGACAUCUC.CGAGCCG.GU.UUAACCUAAGGA.......................GCGA..............................................................................................
Dha-1-4   ACUGAAAAAGAUCUC.CGAGCCG.GAAUGAACCUAAAGAG......................CAA...............................................................................................
Dha-2-3   CUGAAAAACGAUCUC.CGAGCCG.GAAUGAACCUAAAGAG......................CAA...............................................................................................
Dha-2-4   ..........AUCUC.CGAGCCG.GAGCA.ACCUAAAGA.......................GUCA..............................................................................................
Dha-2-5   CUUCAUAAUGAUCUC.CGAGCCG.GAGCA.ACCUAAAGA.......................GUCA..............................................................................................
Mth-1-4   AAUAUUUACAUCCUC.UGAGCCA.UC.CUGGCCUAAAGG.......................AGCAA.............................................................................................
Cpe-2-1   AUUAGUAUAACUCUC.CAAGCUU.AA.AG.AUCUAAGGAG......................AAGG..............................................................................................
Cpe-1-1   AUUAGUAUAAAUCUC.CAAGCUU.AA.AG.AUCUAAGGAG......................AAGG..............................................................................................
Cpe-3-1   AUUAGUAUAACUCUC.CAAGCUU.AA.AG.AUCUAAGGAG......................AAGG..............................................................................................
Pin-1-1   UUUUAAAACCAGCUC.CGAGCUU.AA.UAAAACUAAGUUUCCUU..................UUUA..............................................................................................
Apl-1-1   UAACUUACAGGAUAC.CGAGCUU.GU.AU.GCCUAAGUC.......................UUA...............................................................................................
Hdu-1-1   UAACUUAAACGAUAC.CGAGCUU.GU.UA.GCCUAAGUC.......................UAUU..............................................................................................
Mha-1-1   CAACUUAGAGGAUAC.CGAGCUU.GU.GA.GCCUAAGUGUCUGCU.................GAG...............................................................................................
Apl-1-2   UUUAUCAACAGACUC.CGAGCUU.GU.GA.ACCUAACUUCU.........CGGUCG.UUUUACAAAUUUUCGU.......................................................................................
Hso-2-1   AAAAACUGACGACUC.CGAGCUU.GU.UG.ACCUAAGUU.......................GCAA..............................................................................................
Hso-1-1   AAAAACUGACGACUC.CGAGCUU.GU.UG.ACCUAAGUU.......................GCAA..............................................................................................
Asu-1-1   UCAAUUUUCGUUUAC.CGAGCUU.GU.GU.GCCUAAGUUCUU..CCGG..............UUAU..............................................................................................
Msu-1-1   AGAAAUUUUAUAUAC.CGAGCUU.GU.AC.GCCUAAGUUUUUAAA.................UUUA..............................................................................................
Hso-1-2   GUUAUCGCAUAACUC.CGAGCUU.GU.UU.ACCUAAGUU.......................GAAA..............................................................................................
Hso-2-2   GUUAUCGCAUAACUC.CGAGCUU.GU.UU.ACCUAAGUU.......................GAAA..............................................................................................
Hin-3-1   UUUAACGCAUAACUC.CGAGCUU.GU.UUAGCCUAAGUUU......................CACAAA............................................................................................
Hin-4-1   UUUAACGCAUAACUC.CGAGCUU.GU.UUAGCCUAAGUUUC.....................AUA...............................................................................................
Hin-1-1   UUUAACGCAUAACUC.CGAGCUU.GU.UUAGCCUAAGUUUC.....................AGAA..............................................................................................
Hin-2-1   UUUAACGCAUAACUC.CGAGCUU.GU.UUAGCCUAAGUUUC.....................AUC...............................................................................................
Pmu-1-1   AUUAUCGCAUAACUC.CGAGCUU.AU.UUAACCUACGUUUCUA...................CAA...............................................................................................
Mha-1-2   AUUUAUUGAAGACUC.CGAGCAU.UA.UU.UCCUAACUCUAAAAAUU...............AAAGAC............................................................................................
Plu-1-1   CUAUGGAAUUAACUC.CUAGCCU.CU.GC.AUCUAAGUCACU....................UUUUACAC..........................................................................................
Eca-1-1   AUGAGAAUCAAACUC.CUAGCCU.UG.GU.GCCUAAGUGAUGCGC.................UUAUU.............................................................................................
Spr-1-1   CUGAAACCAAAACUC.CUAGCCU.CU.GG.GCCUACGUUGUUC...................UUGU..............................................................................................
Sgl-1-1   AGGUAUACAUAACUC.CUAGCCU.UU.AC.GCCUAAGUGGCCC...................GUCAU.............................................................................................
Ymo-1-1   ACUGAACAUAAACUC.CUAGCCU.UG.GU.GCCUACGUUGUCC.....CGAGCCGCG.....AAUA..............................................................................................
Yps-2-1   GCUGAACAUAAACUC.CUAGCCU.UA.GC.GUCUACGUUGUC....................UUUUUUGUAUU.......................................................................................
Yps-1-1   ACUGAACAUAAACUC.CUAGCCU.UA.GC.GUCUACGUUGUC....................UUUUUUGUAUU.......................................................................................
Yfr-1-1   ACUGAACAUAAACUC.CUAGCCU.UA.GU.GUCUACGUUGUU....................GUGUUUUCU.........................................................................................
Yin-1-1   ACUGAACAUAAACUC.CUAGCCU.UG.GC.GUCUACGUUGUCUCG.................UUCG..............................................................................................

          ..<<<<<<<<<<<<<.<..<<<<.<<.<<.<<<...<<<<<<<<<<<<<<<<<<<<<<<<<<..................................................................................................
          ..??????????2?2.1..222?.??.??.?21...?222???????????2?222??0000..................................................................................................
          ●●●●●●●●●●●●CUC-CGAGCY●-●●-●●-●YCUARR●●○----------------------●●●○------------------------------------------------------------------------------------------------
                                        -TR-
```

```
Ybe-1-1    ACUGAACAUAAACUC.CUAGCCU.UG.GU.GUCUACGUUGUCCGGUGUCGCGAA.......UACGUUU.........................................................................................
Spe-1-1    AGGUUGACGGUUCUC.CGAGCUU.CU.UA.CCCUACGUC.G....................AGA.............................................................................................
Swo-2-1    UCACUGACGGUUCUC.CGAGCUU.UU.UG.UCCUACGUC......................AACA............................................................................................
Ssp-4-1    GAAACGACGGUUCUC.CGAGCUU.UC.UC.UCCUACGUC......................CACA............................................................................................
Ssp-5-1    CCAGCGACGGUUCUC.CGAGCUU.GC.CU.ACCUACGUC......................AAUA............................................................................................
Ssp-5-2    CCAGCGACGGUUCUC.CGAGCUU.GC.CU.ACCUACGUC......................AAUA............................................................................................
Spu-1-1    CCAGCGACGGUUCUC.CGAGCUU.GC.CU.ACCUACGUC......................AAUA............................................................................................
Sba-1-1    CCGGUGACGGUUCUC.CGAGCUU.GC.CU.ACCUAAGUC......................GAAA............................................................................................
Sba-2-1    CCGGUGACGGUUCUC.CGAGCUU.GC.CU.ACCUAAGUC......................GAAA............................................................................................
Ssp-1-1    CGAAUGACGGUUCUC.CGAGCUU.GC.CU.ACCUAAGUC......................GACA............................................................................................
env-2      CGAAUGACGGUUCUC.CGAGCUU.GC.CU.ACCUAAGUC......................GACA............................................................................................
Ssp-2-1    CGAAUGACGGUUCUC.CGAGCUU.GC.CU.ACCUAAGUC......................GACA............................................................................................
Ssp-3-1    CGAAUGACGGUUCUC.CGAGCUU.GC.CU.ACCUAAGUC......................GACA............................................................................................
Son-1-1    CAGCUGACGGUUCUC.CGAGCUU.GC.CU.ACCUAAGUC......................AACA............................................................................................
env-3      CGGCUGACGGUUCUC.CGAGCUU.GC.CU.ACCUAAGUC......................AACA............................................................................................
Sfr-1-1    GAUCUGACGGUUCUC.CGAGCUU.UU.UG.CCCUACGUC......................AAU.............................................................................................
Sde-1-1    GCAUUGACGGUUCUC.CGAGCUU.CU.UA.UCCUAAGUC......................UUUA............................................................................................
Sam-1-1    UCAAUGACGGUUCUC.CGAGCUC.CU.GC.ACCUAAGUC......................AAUG............................................................................................
Esp-1-1    GAUGAAAACAUACACUCUAGCCU.UG.AC.GCCUGUGUC......................ACAU............................................................................................
env-4      GACGAGUAUAAACACUCUAGCCU.CU.GC.ACCUGGGUC......................AAAU............................................................................................
Eco-15-1   UUAACCACUAAACACUCUAGCCU.CU.GC.ACCUGGGUC......................AACU............................................................................................
Eco-14-1   UUAACCACUAAACACUYUAGCCU.CU.GC.ACCUGGGUC......................AACU............................................................................................
Eco-3-1    AUAACCACUAAACACUCUAGCCU.CU.GC.ACCUGGGUC......................AACU............................................................................................
Eco-1-1    AUAACCACUAAACACUCUAGCCU.CU.GC.ACCUGGGUC......................AACU............................................................................................
Eco-17-1   AUAACCACUAAACACUCUAGCCU.CU.GC.ACCUGGGUC......................AACU............................................................................................
Eco-6-1    AUAACCACUAAACACUCUAGCCU.CU.GC.ACCUGGGUC......................AACU............................................................................................
Eco-2-1    UUAACCACUAAACACUCUAGCCU.CU.GC.ACCUGGGUC......................AACU............................................................................................
Sfl-2-1    UUAACCACUAAACACUCUAGCCU.CU.GC.ACCUGGGUC......................AACU............................................................................................
Sbo-2-1    UUAACCACUAAACACUCUAGCCU.CU.GC.ACCUGGGUC......................AACU............................................................................................
Sso-1-1    UUAACCACUAAACACUCUAGCCU.CU.GC.ACCUGGGUC......................AACU............................................................................................
Sfl-3-1    UUAACCACUAAACACUCUAGCCU.CU.GC.ACCUGGGUC......................AACU............................................................................................
Sdy-1-1    UUAACCACUAAACACUCUAGCCU.CU.GC.ACCUAGGUC......................AACU............................................................................................
Sbo-1-1    UUAACCACUAAACACUCUAGCCU.CU.GC.ACCUAGGUC......................AACU............................................................................................
Sty-1-1    AACCACAGUAAACACUCUAGCCU.CU.GC.ACCUGGGUCA.....................UUUU............................................................................................
Sen-1-1    AACCACAAUAAACACUCUAGCCU.CU.GC.ACUUGGGUCA.....................UUUU............................................................................................
Sen-4-1    AACCACAGUAAACACUCUAGCCU.CU.GC.ACCUGGGUCA.....................UUUU............................................................................................
Sen-2-1    AACCACAAUAAACACUCUAGCCU.CU.GC.ACCUGGGUCA.....................UUUU............................................................................................
Ahy-1-1    AAACUAGAUGAACUC.CGAGCGG.UU.UU.GCCUAAGUC......................CUCAU...........................................................................................
env-5      AAACUAGAUGAACUC.CGAGCGG.UU.UU.GCCUAAGUC......................CUCAU...........................................................................................
Vch-1-1    GCUUGGCCUUAACUC.CGAGCUU.AC.CG.CGCUAAGUUUAA...................ACCU............................................................................................
Vch-8-1    GCUUGGCCUUAACUC.CGAGCUU.AC.CG.CGCUAAGUUUAA...................ACCU............................................................................................
Vch-10-1   GCUUGGCCUUAACUC.CGAGCUU.AC.CG.CGCUAAGUUUAA...................ACCU............................................................................................
Vch-13-1   GCUUGGCCUUAACUC.CGAGCUU.AC.CG.CGCUAAGUUUAA...................ACCU............................................................................................
Vch-7-1    GCUUGGCCUUAACUC.CGAGCUU.AC.CG.CGCUAAGUUUAA...................ACCU............................................................................................
Vch-12-1   GCUUGGCCUUAACUC.CGAGCUU.AC.CG.CGCUAAGUUUAA...................ACCU............................................................................................
Vch-3-1    GCUUGGCCUUAACUC.CGAGCUU.AC.CG.CGCUAAGUUUA....................AACCUG..........................................................................................
Vfi-1-1    UGGCUGCCAUAACUC.CGAGCUC.AA.GC.UACUAAGUUG....UUUU.............UUAA............................................................................................
Vvu-1-1    GGGUUGCCAUAACUC.CGAGCUU.AA.CU.UGCUAAGUUAUUUUAUU..............UCAAU...........................................................................................
Vvu-2-1    GGGUUGCCAUAACUC.CGAGCUU.AA.CU.UGCUAAGUUAUUUUAUU..............UCAAU...........................................................................................
Vsp-2-1    CAGUUGCCAUAACUC.CGAGCUC.UC.GCAUGCUAAGUCG.CACAGUG.............GUAUU...........................................................................................
Vsp-3-1    CAGUUGCCAUAACUC.CGAGCUC.UC.GCAUGCUAAGUCG.CACAGUG.............GUAUU...........................................................................................
Vpa-1-1    UGGUUGCCAUAACUC.CGAGCUC.AC.UA.UGCUAAGUCC.....................GA..............................................................................................
Val-1-1    UGGUUGCCAUAACUC.CGAGCUC.AC.UAUUGCUAAGUCC.....................UU..............................................................................................
Vsp-1-1    UGGUUGCCAUAACUC.CGAGCUC.AC.UAUUGCUAAGUCC.....................GA..............................................................................................
Psp-1-1    UAACUGCCAUAACUC.CGAGCAU.UC.UU.CACUAAGUUA.CUAAGCGAUGU.........UGGACUUAUAUAUAUAU...............................................................................
Van-1-1    UAACUGCCAUAACUC.CGAGCAU.UU.UU.CACUAAGUUA.CUAAGCGAUGU.........UGGACAUUAAUAUAU.................................................................................
Ppr-2-1    UAGCUGCCAUAACUC.CGAGCUU.UA.GU.CACUACGUUUUCUGC.UUU............UCUU............................................................................................
Ppr-1-1    UAGCUGCCAUAACUC.CGAGCUU.UG.GU.CACUACGUUUUCUGUUUU.............UCUU............................................................................................
Dha-2-6    AAUUGAAUAGUUUUC.CGAGCUC.CU.UU.GUCUAAAAGA.....................CUA.............................................................................................
Dha-1-6    AAUUGAAUAGUUUUC.CGAGCUC.CU.UU.GUCUAAAAGA.....................CUA.............................................................................................
Dha-1-7    AAUAGUGAAAUCUUU.CGAAUCG.UUUCC.CGCUAAAGC......................CAU.............................................................................................

           ..<<<<<<<<<<<<<.<..<<<<.<<.<<.<<<...<<<<<<<<<<<<<<<<<<<<<<<<<................................................................................................
           ..??????????2?2.1..222?.??.??.?21...?222??????????2?222??0000................................................................................................
           ●●●●●●●●●●●●CUC-CGAGCY●-●●-●●-●YCUARR●●○---------------------●●●○--------------------------------------------------------------------------------------------
                                           -TR-
```

```
Dha-1-8   AAUAGUGAAAUCUUU.CGAAUCG.UUUCC.CGCUAAAGC.......................CAU...............................................................................................
Dha-2-7   AAUAGUGAAAUCUUU.CGAAUCG.UUUCC.CGCUAAAGC.......................CAU...............................................................................................
          ..<<<<<<<<<<<<<.<..<<<<.<<.<<.<<<...<<<<<<<<<<<<<<<<<<<<<<<<<<..................................................................................................
          ..??????????2?2.1..222?.??.??.?21...?222???????????2?222??0000..................................................................................................
          ●●●●●●●●●●●CUC-CGAGCY●-●●-●●-●YCUARR●●○-----------------------●●●○-----------------------------------------------------------------------------------------------
                                        -TR-
```

alignment positions 161 · · · 320

```
Aba-1-1   ..........................................CUAAGGCAU.CGGG.CGCG..GUAUCCAUCU............................UUGU..........................................AGAUGGAU.C...
Tth-1-1   ........................................GGGC...CU.C.CCGG.GCC...GGGCC.................................CACG...............................................GGCCC...
Tth-2-1   ........................................GGGC...CU.C.CCGG.GCC...GGGCC.................................CACG...............................................GGCCC...
Sth-1-1   ........................................GGGUAUGGUCG.CCUG.ACCG..GUCCCGGU..............................CGU.............................................ACC.GGGC...
Csp-1-1   .......................................GCGCCAAGGCCG.GGCC.GCC.GCGCCGG.................................UACG...............................................CCGGC...
Rsp-1-1   ........................................CCAUAAGCCCU.UGUC.ACC...UCGC..................................AGUA................................................GCGA...
Ama-1-1   ........................................AACUAUGGUAC.UGGA.GCC...GCAACU................................GAGA..............................................AGUUGC...
Cps-1-1   .......................AGCCAACUAUUCGAAGCAUUCAUGUUGU.GACAUGCC...GUGACUAAAA.CCA.UG.....................UAUU...................................UAUUGGCUUUUAGUUGC...
Dha-2-1   .........................................GCUAUGGUUUACCAG..UC...GUGCA.................................UUC................................................UGCAC...
Dha-1-1   .........................................GCUAUGGUUUACCAG..UC...GUGCA.................................UUC................................................UGCAC...
Dha-2-2   .........................................UCUACGGAAU.UUUG.GCC...GUUUCAGA..............................AAUA............................................UCUGAAAC...
Pat-1-1   .........................................GACAAGGUAU.GUUG.GCC...GCGAAGUG...CGUUC......................GCAA....................................GAAUGUGUCA.UUCGC...
Pat-1-2   .........................................GACAAGGUAU.GUUG.GCC...GCGAAGUG...CGUUC......................GCAA....................................GAAUGUGUCA.UUCGC...
Swo-1-1   .......................................AAGCCACGGAUA.AUUG.GACGAAAGGAGC................................AUCAAAC...........................................GCUCCC...
Mth-1-1   ........................................GGCUAUGGC.U.GCUG.GCC...UC.CUGCCUG............................CUUGC.........................................CAGGCAGCGA...
Mth-1-2   ........................................GCCUAUGGAAC.ACCG.GCC...UGGGU.................................UUCGU..............................................ACCCA...
Dha-1-3   .........................................UCUACGGU.U.GCCG.GCC...AGGAC.................................GAAA...............................................GUCCU...
Mth-1-3   .........................................UCUAUGGU.G.GCCG.GCC...UGGGCU................................GAGA..............................................GGCCCA...
Dha-1-4   ........................................UUCUAUGGACA..CCG.GAC...AGGGC.................................CCAG...............................................GCCCU...
Dha-2-3   ........................................UUCUAUGGACA..CCG.GAC...AGGGC.................................CCAG...............................................GCCCU...
Dha-2-4   .........................................UCCAUGGUUG.GUCG.GCC...AGGGC.................................UGUAC..............................................GCCCU...
Dha-2-5   .........................................UCCAUGGUUG.GUCG.GCC...AGGGC.................................UGUAC..............................................GCCCU...
Mth-1-4   .........................................CCCAUGGCAG.GUUG.GCC...UGGACC................................GCU...............................................GGUCCA...
Cpe-2-1   ........................................UUCUAUGAUUU.UUAA.GCC...UGGAUUACUAAAUUA.......................UUUAACAU.................................UAAGUUGGUAAUCCA...
Cpe-1-1   ........................................UUCUAUGAUUU.UUAA.GCC...UGGAUUAC......UAAAUUAUUUAACA..UUAAGUUUAAUAA.............GAACUUAAAUUGUUAAUUGGUUUAAUUGGGGUAAUCCA...
Cpe-3-1   ........................................UUCUAUGAUUU.UUAA.GCC...UGGAUUAC......UAAAUUAUUUAACA..UUAAGUUUAAUAA.............GAACUUAAAUUGUUAAUUGGUUUAAUUGGGGUAAUCCA...
Pin-1-1   ....................................AGGUAAAUAAGUCUG.UUAA.GCCG..CUUGU.CUGAC...........................AUUUUG.......................................GUCAGCGCAAG...
Apl-1-1   .........................................GAUAAGGUGU.GCAA.GCC...UGUGGCAAGCGGUU..........AAAAUU........CACAA......................AAUUUUGCAAAAUCAGACCGCUU.ACACA...
Hdu-1-1   .........................................GAUAAGGC.A.GCAAAGCC...UGCAAUAUAAGCGGUCG..AGUUGA.UG..........AAA.........................UAUUUAGCUAUUAAUCGCUUAUUUUGCA...
Mha-1-1   ...................................AGCAGAGAUAUGGU.A.GCAA.GCC....GUAAAG.CACCUACUACAAGCGGUCAUUUUUUCCUAACAUU...............UUG..CAAAAAUUACCGUC......GGUGCUUUUACC...
Apl-1-2   ..................UAAAUUCGACCGCUUUUUGUAAGAAUAUGGU.A.GCAAUGCCU..GUAAUUAGCUUA..........................UACG........................................U.AGCUGAUU.C...
Hso-2-1   .........................................AAUAAGGU.U.GCAA.GCC...UGUAACAUU.............................GUCA...........................................AAUGUUGCA...
Hso-1-1   .........................................AAUAAGGU.U.GCAA.GCC...UGUAACAUU.............................GUCA...........................................AAUGUUGCA...
Asu-1-1   ................................CCGGCGCGGAAUAAGGC.A.GCAACGCC...AGCGAUG...............................CGGUU............................................UAUCGCU...
Msu-1-1   ...................................UUUGAAAAUAAGGCG..GCAAUGCC...AGCAAUGA..............................AUU.............................................UUAUUGCU...
Hso-1-2   .........................................AAUAAGGA.A.ACAAUGCC...AGCAA.................................AUAUAA.............................................UUGCU...
Hso-2-2   .........................................AAUAAGGA.A.ACAAUGCC...AGCAA.................................AUAUAA.............................................UUGCU...
Hin-3-1   ........................................AAAUACGGU.G.GCAA.GCC...AAUAACG...............................CGGUA............................................CGUUAUU...
Hin-4-1   .......................................GAAAUACGGU.G.GCAA.GCC...AAUAACG...............................CGCUA............................................CGUUAUU...
Hin-1-1   .......................................GAAAUACGGU.G.GCAA.GCC...AAUAACGC..............................CGU.............................................GUGUUAUU...
Hin-2-1   .......................................GAAAUACGGC.G.ACAA.GCC...AAUAACGC..............................CGU.............................................GUGUUAUU...
Pmu-1-1   .....................................UAGAAAUAAGGU.G.GUAAUGCC...AAUAGCAUAA............................AAC...........................................UUGUGCUAUU...
Mha-1-2   ..................................AAAUUUUGAUAUGGAAU.UUAU.GCCA..GUAACAGC..............................AUAAA...........................................GCUGUU.C...
Plu-1-1   ......................................AGCGAUAUGAUGU.UUUG.GCC...GUGAC.................................CACUGA.............................................GUCAC...
Eca-1-1   ...................................GCGCGUGAUACGGUGC.CUUG.GCC...GUGGCC................................UUCGU.............................................GGUCAC...
Spr-1-1   .....................................GAACAAUAUGGCGC.UUGG.GCC...GUGGC.G...............................AAGU.............................................CAGCCAC...
Sgl-1-1   .....................................GAGCCAUAGGGGGU.UCUG.GCC...GUGGC.................................GAA................................................GCCAC...
Ymo-1-1   .......................UGCGUUUCGUUUAUGGACAAUAGGGUGC.UCUG.GCC...GUGGCU................................UUA...............................................AGCCAC...
Yps-2-1   ......................................GGCAAUAUGAUGU.UCUG.GCC...GUGGC.................................CUUAA..............................................GCCAC...
Yps-1-1   ......................................GGCAAUAUGAUGU.UCUG.GCC...GUGGC.................................CUUAA..............................................GCCAC...
Yfr-1-1   ......................................AACAAUAUGAUGC.UCUG.GCC...GUGGCU................................UUA...............................................AGCCAC...

          ..................>>>>>>>>>>>>>>>>>>>>>>>>>>..>>>>>.>>>>.>>....<<<<<<<<<<<<<<<<<<<<<<<<<<<<<<<<<<<<<<..................>>>>>>>>>>>>>>>>>>>>>>>>>>>>>>>>>>>>>>...
          ..................0000??222?2???????????222?..12???.???2.22....22222?22????????????????2?22??00???122..................221???00??22?2????????????????22?22222...
          ----------------------------------------○●●UA●GR●○●-●●●R-GCC---●●RRY○---------------------------------●●●○---------------------------------------------○●YY●●---
                                                   -TR
```

```
Yin-1-1    ...................................CGA.ACAAUAUGAUGC.UCUG.GCC...GUGGCU................................UUA................................................AGCCAC...
Ybe-1-1    ..........................UUCGUU.UACC.GGCAAUAUGAUGC.UCUG.GCC...GUGGCU................................UUA................................................AGCCAC...
Spe-1-1    .......................................CAGAUAAGGGUU.UUUA.GCC...GUGGC.................................AAUAAC..............................................GCCAC...
Swo-2-1    .........................................GAUAUGGACG.CUAA.GCC...GUGGC.................................AAUAUC..............................................GCCAC...
Ssp-4-1    .........................................GAUAGGGAGA.UUAA.GCC...GUGGCA................................AUAG...............................................UGCCAC...
Ssp-5-1    .........................................GAUAUGGAGG.UGUA.GCC...GUGGUA................................AUAA...............................................UGCCAC...
Ssp-5-2    .........................................GAUAUGGAGG.UGUA.GCC...GUGGUA................................AUAA...............................................UGCCAC...
Spu-1-1    .........................................GAUAUGGAGG.UGUA.GCC...GUGGUA................................AUAA...............................................UGCCAC...
Sba-1-1    .........................................GAUAUGGAGG.UGUA.GCC...GUGGUA................................AUAA...............................................UGCCAC...
Sba-2-1    .........................................GAUAUGGAGG.UGUA.GCC...GUGGUA................................AUAA...............................................UGCCAC...
Ssp-1-1    .........................................GAUAUGGGGG.UGUA.GCC...GUGGU.................................AAUAAG..............................................GCCAC...
env-2      .........................................GAUAUGGGGG.UGUA.GCC...GUGGU.................................AAUAAG..............................................GCCAC...
Ssp-2-1    .........................................GAUAUGGGGG.UGUA.GCC...GUGGC.................................AAUAAG..............................................GCCAC...
Ssp-3-1    .........................................GAUAUGGGGG.UGUA.GCC...GUGGC.................................AAUAAG..............................................GCCAC...
Son-1-1    .........................................GAUAUGGGGG.UGUA.GCC...GUGGC.................................AAUAAG..............................................GCCAC...
env-3      .........................................GAUAUGGUGG.UGUA.GCC...GUGGC.................................AAUAAG..............................................GCCAC...
Sfr-1-1    .........................................GAUAUGGGCA.GUUA.GCC...GUGGUA................................AUUU...............................................UGCCAC...
Sde-1-1    .........................................GAUACGGGUC.AUUA.GCC...GUGGUA................................AUAA...............................................UGCCAC...
Sam-1-1    .........................................GAUAUGGUGU.UUGA.GCC...GUGGC.................................GCGGU...............................................GCCAC...
Esp-1-1    .........................................GAUAUGGCGC.CCUG.GCC...GUGGCG................................GUUU...............................................CGCCAC...
env-4      .........................................GAUACGGUGC.UUUG.GCC...GUGACAAGACUU..........................GAAA.........................................AAG.UUUGUCAC...
Eco-15-1   .........................................GAUAUGGUGC.UUUG.GCC...GUGACAAUGCU...........................CGUAA.........................................AG.AUUGCCAC...
Eco-14-1   .........................................GAUAUGGUGC.UUUG.GCC...GUGACAAUGCU...........................CGUAA.........................................AG.AUUGCCAC...
Eco-3-1    .........................................GAUACGGUGC.UUUG.GCC...GUGACAAUGCU...........................CGUAA.........................................AG.AUUGCCAC...
Eco-1-1    .........................................GAUACGGUGC.UUUG.GCC...GUGACAAUGCU...........................CGUAA.........................................AG.AUUGCCAC...
Eco-17-1   .........................................GAUACGGUGC.UUUG.GCC...GUGACAAUGCU...........................CGUAA.........................................AG.AUUGCCAC...
Eco-6-1    .........................................GAUACGGUGC.UUUG.GCC...GUGACAAUGCU...........................CGUAA.........................................AG.AUUGCCAC...
Eco-2-1    .........................................GAUACGGUGC.UUUG.GCC...GUGACAAUGCU...........................CGUAA.........................................AG.AUUGCCAC...
Sfl-2-1    .........................................GAUACGGUGC.UUUG.GCC...GUGACAAUGCU...........................CGUAA.........................................AG.AUUGCCAC...
Sbo-2-1    .........................................GAUACGGUGC.UUUG.GCC...GUGACAAUGCU...........................CGUAA.........................................AG.AUUGCCAC...
Sso-1-1    .........................................GAUACGGUGC.UUUG.GCC...GUGACAAUGCU...........................CGUAA.........................................AG.AUUGCCAC...
Sfl-3-1    .........................................GAUACGGUGC.UUUG.GCC...GUGACAAUGCU...........................CGUAA.........................................AG.AUUGCCAC...
Sdy-1-1    .........................................GAUACGGUGC.UUUG.GCC...GUGACAAUGCU...........................CGUAA.........................................AG.AUUGCCAC...
Sbo-1-1    .........................................GAUACGGUGC.UUUG.GCC...GUGACAAUGCU...........................CGUAA.........................................AG.AUUGCCAC...
Sty-1-1    ........................................UGAUACGGUGC.UUUG.GCC...GUGACAA.CGU...........................UUA...........................................ACGUUUGCCAC...
Sen-1-1    ........................................UGAUAUGGUGC.UUUG.GCC...GUGACAA.CGU...........................UUA...........................................ACGUUUGCCAC...
Sen-4-1    ........................................UGAUAUGGUGC.UUUG.GCC...GUGACAA.CGU...........................UUA...........................................ACGUUUGCCAC...
Sen-2-1    ........................................UGAUAUGGUGC.UUUG.GCC...GUGACAA.CGU...........................UUA...........................................ACGUUUGCCAC...
Ahy-1-1    .........................................GAUAGGGGGA.AACC.GCCG..UCAGC.CGA.............................UGU.............................................UCGAGCUGU...
env-5      .........................................GAUAGGGGGA.AACC.GCCG..UCAGC.CGA.............................UGU.............................................UCGAGCUGU...
Vch-1-1    ......................................UUAAAUAUGCGUU.GUAA.GCCA..GUGAC.................................CGCUU...............................................GUCAC...
Vch-8-1    ......................................UUAAAUAUGCGUU.GUAA.GCCA..GUGAC.................................CGCUU...............................................GUCAC...
Vch-10-1   ......................................UUAAAUAUGCGUU.GUAA.GCCA..GUGAC.................................CGCUU...............................................GUCAC...
Vch-13-1   ......................................UUAAAUAUGCGUU.GUAA.GCCA..GUGAC.................................CGCUU...............................................GUCAC...
Vch-7-1    ......................................UUAAAUAUGCGUU.GUAA.GCCA..GUGAC.................................CGCUU...............................................GUCAC...
Vch-12-1   ......................................UUAAAUAUGCGUU.GUAA.GCCA..GUGAC.................................CGCUU...............................................GUCAC...
Vch-3-1    .......................................UAAAUAUGCGUU.GUAA.GCCA..GUGGCA................................GAA................................................UGUCAC...
Vfi-1-1    ................................GAAGCACUCAAUAUGUAGC.GUGA.GCCA..GUGAC.................................AAUUCG..............................................GUCAC...
Vvu-1-1    .................................GAUGGAAUGAUAAGCAGC.UUAA.GCCA..GUGACA................................UGA................................................UGUCAC...
Vvu-2-1    .................................GAUGGAAUGAUAAGCAGC.UUAA.GCCA..GUGACA................................UGA................................................UGUCAC...
Vsp-2-1    ................................UGCUGUACCGAUACGCAUC.AAGA.GCCA..GUGACA................................UGU................................................UGUCAC...
Vsp-3-1    ................................UGCUGUACCGAUACGCAUC.AAGA.GCCA..GUGACA................................UGU................................................UGUCAC...
Vpa-1-1    ........................................GGAUAAGCAUU.UUGA.GCCA..GUGACA................................UAC................................................UGUCAC...
Val-1-1    ........................................GGAUAAGCGUU.UUGA.GCCA..GUGGCA................................UAC................................................UGUCAC...
Vsp-1-1    ........................................GGAUAAGCGUU.UUGA.GCCA..GUGGCG................................UAC................................................UGUCAC...
Psp-1-1    ............................ACGUUCAAAAGUUGAUAGGUGAU.UAAU.GCCA..GUGACG................................AUU................................................UGUCAC...
Van-1-1    ............................ACGUUCAAAAGUUGAUAGGUGAU.UAAU.GCCA..GUGACG................................AUU................................................UGUCAC...
Ppr-2-1    ...............................AAAUUCAGAAUAUAUGUGUU.CAGA.GCCA..GUGACA................................UAC................................................UGUCAC...
Ppr-1-1    ................................AAAUCAGAAUAUAUGUGUU.CAGA.GCCA..GUGACA................................UAC................................................UGUCAC...
Dha-2-6    ........................................UCUCAUGACGG.AGGA.GCC...UGAGC.................................AAACC...............................................GCUCA...
Dha-1-6    ........................................UCUCAUGACGG.AGGA.GCC...UGAGC.................................AAACC...............................................GCUCA...

           ..................>>>>>>>>>>>>>>>>>>>>>>>>>>..>>>>>.>>>>.>>....<<<<<<<<<<<<<<<<<<<<<<<<<<<<<<<<<<<<<<...................>>>>>>>>>>>>>>>>>>>>>>>>>>>>>>>>>>>>>>...
           ..................0000??222?2???????????222?..12???.???2.22....22222?22????????????????2?22??00???122...................221???00??22?2????????????????22?22222...
           ----------------------------------------○●●UA●GR●●●-●●●R-GCC---●●RRY○--------------------------------●●●○-----------------------------------------------○●YY●●---
                                                      -TR
```

```
Dha-1-7   .........................................GCUAUGCGGG.GGCG.AUC...UGAGC.................................AAAG...............................................GCUCA...
Dha-1-8   .........................................GCUAUGCGGG.GGCG.AUC...UGAGC.................................AAAG...............................................GCUCA...
Dha-2-7   .........................................GCUAUGCGGG.GGCG.AUC...UGAGC.................................AAAG...............................................GCUCA...
          ..................>>>>>>>>>>>>>>>>>>>>>>>>>>..>>>>>.>>>>.>>....<<<<<<<<<<<<<<<<<<<<<<<<<<<<<<<<<<<<<<..................>>>>>>>>>>>>>>>>>>>>>>>>>>>>>>>>>>>>>>...
          ..................0000??222?2???????????222?..12???.???2.22....22222?22????????????????2?22??00???122..................221??00??22?2?????????????????22?22222...
          -----------------------------------------○●●UA●GR●○●-●●●R-GCC---●●RRY○--------------------------------●●●○----------------------------------------------○●YY●●---
                                                     -TR
```

alignment positions 321 · · · 480

```
Aba-1-1   GAGGGUGA.CG...AA.GGAAACUUCGCCGCCUCCCGUU....................................UG.GAAAGGAA.G.CACCU.C.GGCGCAUGCUGCCGCGGUGUGUUCUGCGCGGCAUGACACUGACCGAUAAAUUCGGACGCACUA
Tth-1-1   CGGGGUGG.CG...GG.GGCAACCCCGCCGCCUGCCGCGGU..................................CG.CAAAGGAG.G.UG.CGCA.UGGGAAGACGUUUGGUGGCCCUUCUUCUCCUCUUCCUCCCCGGCUUCGCCCAGAAGGUGCGGG
Tth-2-1   CGGGGUGG.CG...GG.GGCAACCCCGCCGCCUGCCGCGGU..................................CG.CAAAGGAG.G.UG.CGCA.UGGGAAGACGUUUGGUGGCCCUUCUUCUCCUCUUCCUCCCCGGCUUCGCCCAGAAGGUGCGGG
Sth-1-1   CAGGGUUG.UC...GG.GGAAACCUGGCGGCCUCCCGU.....................................UG.GAAAGGGG.C.GG.AA.C.UGCGGGAGAUGCCCGCGUCGUUCUGCUAUCUUCCGUUCGGGAGAUAGCUUUUUUGCUCCAGGC
Csp-1-1   AAGGGUGA.CG...GG.GGAAACGCCGUCGCCUCCCUUGCU..................................UG.GAAAGGAG.C.CC.GC.A.UGGGACGUCUGGUUGACGGCUUCGGCCGCCGCUUUCGCUAUCUGCGCCUCUCGGUCACCGAGG
Rsp-1-1   CAGGGUAG.CGA..UU.GGAAACGGCGUUGCCUCCCGUGUU..................................UG.GAAAGGUG.A.AU.AA.U.GUUACAGGAUUCUUUUGGACGCCGGUUCUAUUACCUGCGUCUGUCGGUGACAGAUGUCUGCAA
Ama-1-1   CC.GGUAG.CC...AG.AGAAAUCAACCUGCCGCCCACG........AGCUUUAACGCUCGAUAUU.........UG.GAAAGGUG.U.UA.GA.U.GAACUUACAAGACUCAUUCGGAAGAAGAUUCAAUUACCUGAGACUCUCAGUAACUGAGCUGUG
Cps-1-1   CAGGGUUA.AC...AA.GGAAACGCGUUAGCCUCCCUACAUU.................................UG.GAAAGGUG.ACAU.GU.U.AACAGACAACUUUGGCCGUAGGUUCUCUUACCUGCGUCUCUCUAUUACCGACGUUUGCAAUUU
Dha-2-1   CGG.GUAU.UA...UU.GGAAACAGUAAUGCCUCCCAUU....................................UG.GAAAGGAGUC.UG.UA.G.GGUAUGCGGGAGCUCCGCGGUUUUACAGACCGUGGGGCUUAUUUAUUUUGUGGAAUAAUGAAC
Dha-1-1   CGG.GUAU.UA...UU.GGAAACAGUAAUGCCUCCCAUU....................................UG.GAAAGGAGUC.UG.UA.G.GGUAUGCGGGAGCUCCGCGGUUUUACAGACCGUGGGGCUUAUUUAUUUUGUGGAAUAAUGAAC
Dha-2-2   CAGGGUAU.GC...UU.AGAAAUGAACAUGCCUUCCGUAA...................................AG.AAAAGGAGGA.AC.CC.C.UAUGCAGAAGAUUAAAGUACAGGAUGCGAUUGGAUCGGUGCUUAUGCACGAUCUGACACAAAU
Pat-1-1   CAGGGUUG.UG...AG.AGAAAUCAAACAGCCUCCCCGUAAG.................................UG.CCAAGGCG.A.UA.AG.G.CCCGGCAACUUCAAUUUGGAAAGGUGUUAGUGAAUAUGUUGCAAGAUAAAUUUGGUCGCCGGU
Pat-1-2   CAGGGUUG.UG...AG.AGAAAUCAAACAGCCUCCCCGUAAGUGCCAAGGCGAUAAGGCCCGGCAACUUCAAUU.UG.GAAAGGUG.U.UA.GU.G.AAUAUGUUGCAAGAUAAAUUUGGUCGCCGGUUUCAUUACCUGCGGCUGUCCAUUACUGAUGUG
Swo-1-1   CAGGGUAU.AG...CU.GUAAACGGCUGUGCCUCCCGUAUU..................................UG.GAAAGAAG.A.UA.UG.G.AACAUCCUUUCUGAGGGUUAGUCAUGUUGGCUAACAAACUCAGGAGAUUAACAUAAGCAGGAA
Mth-1-1   CGGGGUAG.GC...GG.GGAAACCGGCCUGCCUCCCGGAAU..................................GG.GAAAGGAG.U.AU.UU.G.ACCCGGUGGUCAACCGGGCCGGAUAGCUUCAGCACCCUUUCCGGGUGCCUUUAAUUUUUUGCG
Mth-1-2   UGGGGUAG.GU...CG.GGAAACGGGCCUGCCUCCCUUUGCU.................................CG.GAAAGGAG.A.UU.CC.C.CAUGGCUUCUGGACAACGGAGUCCCGGUAUCCUUUGCGAGCCGGGCUUUUUUAUUUAUUCCGG
Dha-1-3   CAGGGUGU.GU...CU.GGAAACGGAUAGGCCUUCACGUU...................................UG.AAAAGGAG.G.GA.AG.A.AAAGUGCUUUGCAAUCAAGGUGAAGCAGGAAGAAGAGUAAGAUUGGUGUUUGAUAAUGACGGG
Mth-1-3   CGGGGUAU.AC...CG.GGAAACUGGUAUGCCUUCCCAGGUUU.G..............................AGUAUAAGGAG.G.UG.GC.U.GAUUAGUGGCUGUUUAUGGCUUUUAUUAGCCCCACCUUCCUACAGGUGGGGUUUUAGUUUUAG
Dha-1-4   CGGGGUAU.AC...UU.GGAAACGGGUAUGCCUUCACGUU...................................UG.AAAAGGAG.G.UG.GA.U.UAAAGCCAACUGUGUAUGAAAUCAGUUAUGGCUCCGCUUUCUAUUGGCGGAGCCAUUAUUUUU
Dha-2-3   CGGGGUAU.AC...UU.GGAAACGGGUAUGCCUUCACGUU...................................UG.AAAAGGAG.G.UG.GA.U.UAAAGCCAACUGUGUAUGAAAUCAGUUAUGGCUCCGCUUUCUAUUGGCGGAGCCAUUAUUUUU
Dha-2-4   CGGGGUAU.AU...CC.AGAAAUGGCUAUGCCUUCACGUU...................................UG.AGAAGGAG.G.U..............GGAUUCCCAAUAUGUUGGGUUUACUAGAGGUUCCACUUUCGUUAAAGUGGAACCUU
Dha-2-5   CGGGGUAU.AU...CC.AGAAAUGGCUAUGCCUUCACGUU...................................UG.AGAAGGAG.G.UGGAU.U.CCCAAUAUGUUGGGUUUACUAGAGGUUCCACUUUCGUUAAAGUGGAACCUUUUAACGUUUAAA
Mth-1-4   CGGGGUGU.AC...CG.GGAAACGGGUAUGCCUUCCAGGUAU.................................UG.AAAAGGAG.G.GG.GU.U.AGCUAUAAGAGAGAGCUCUGGCUUUUUUAGUUAAACCCCGCUCCGCCGUGGAGACGGCGUUUU
Cpe-2-1   CAGGGUAA.GG...UU.UGAAAGAAUUUUGCCUCCACGUAUUU................................UG.GGAAGGAG.A.AU.AA.G.CAAAAGUAAAAAACAUAGAACUUACAUAAUAAUAUUGUUAAUUAUGUUUUUUAGUUUUAAUAU
Cpe-1-1   CAGGGUAA.GG...UU.UGAAAGAAUUUUGCCUCCACGUAUUU................................UG.GGAAGGAG.A.AU.AA.G.CAAAAGUAAAAAACAUAGAACUUACAUAAUAAUAUUGUUAAUUAUGUUUUUUAGUUUUAAUAU
Cpe-3-1   CAGGGUAA.GG...UU.UGAAAGAAUUUUGCCUCCACGUAUUU................................UG.GGAAGGAG.A.AU.AA.G.CAAAAGUAAAAAACAUAGAACUUACAUAAUAAUAUUGUUAAUUAUGUUUUUUAGUUUUAAUAU
Pin-1-1   AAGGGUAA.AG...AG.AGAAAUGUCUUUGCCUCCCGUAUU..................................UG.GAAAGGAA.C.UU.AA.C.AUGAGCCAACAACUUAUUGAUAACUUCGAUCGGAAGUUUGAAUACUUACGCCUGUCGAUAACC
Apl-1-1   CC.GGAUA.GU...AA.AGAAAUGCACUAUCCCCUCGUGUU..................................UA.GAAAGGUG.U.CA.AA.U.UCAACUUAACUUUCAGAGGGUUAUGCAUAUGAAAACUCAAGAAAAAAUUAAUGCAAGUCGUCG
Hdu-1-1   CAG.GAUA.GU...AA.AGAAAUGCACUAUCCUCUCAUAUU..................................CA.GAAAGGUG.U.CA.AA.U.UUUCAUUCACUUUCAGAGGACUAUCCAGAUGAAAAAAACAGAAAAUAUUAAUACGAGCCGUCG
Mha-1-1   AAUGGAUA.CA...CG.AGAAAUGGUUUAUCCACUCGACUU..................................UA.GAAAGGUG.U.CU.AU.U.UUUAACUUAACUUCAAACGAGGUUUAUUAUGAAAAAACAAGACAAUAUUGACGUAAGUCGUCG
Apl-1-2   AAGGGAAU.UU...CA.AGAAAUUGUUGUUCCUCUCGUGUU..................................UA.GAAAGGUG.U.CC.AA.U.GCAAUCCAUUCCAAUUAAACAUGUUGGGGCGGAUAAUGUUUCUCAGCUUAUUGAUCGCUUUGA
Hso-2-1   CAUGGUAU.AA...CU.AGAAAUGGUUGUGCCACUCCCUU...................................UA.GAAAGGUG.U.CG.AA.A.AUAAAACUUUCACAAUGUGUUGGAGAAAGUGCGGUGUGUUUUCUAACCGUUUCGAUGUCUUUU
Hso-1-1   CAUGGUAC.AA...CU.AGAAAUGGUUGUGCCACUCCCUU...................................UA.GAAAGGUG.U.CG.AA.A.AUAAAACUUUCACAAUGUGUUGGAGAAAGUGCGGUGUGUUUUCUAACCGUUUCGAUGUCUUUU
Asu-1-1   CAGGGAUC.CG...UU.GGAAACAGUUGGUCCUACCGAUUUU.................................UG.UAAAGGUA.U.UC.AA.U.GCAAUCUAUUCCUGUGAAAAAUGCCGGCGAUCGUCUUGUGGAUGCGUACCGGCGUACCUAUUA
Msu-1-1   CAGGGAUAAUG...UG.GGAAACGACUUAUCCUCUCAUCUU..................................CA.GAAAGGUA.U.CA.AA.U.GCAAUCUAUUCCUAUCAAAAACGUAGGAACCAAUCGAUUAGUCGAUACGUUCCAACGCGAAUA
Hso-1-2   CAGGGAUAAUA...UU.GGAAACGACUUAUCCUUUCUUAUU..................................UA.AAAAGGUG.UCUA.AU.G.CAAAGAUCUAUUUCUAUUCAUAAUGUUAUUGAAAAUCAUCUUAUUGAUGCGUUUCAACGUAAA
Hso-2-2   CAGGGAUAAUA...UU.GGAAACGACUUAUCCUUUCUUAUU..................................UA.AAAAGGUG.UCUA.AU.G.CAAAGAUCUAUUUCUAUUCAUAAUGUUAUUGAAAAUCAUCUUAUUGAUGCGUUUCAACGUAAA
Hin-3-1   CAGGGAUA.CG...CU.GGAAACGGUUUAUCCUCUCCAUAUU.................................UA.GAAAGGUG.U.AA.AA.U.GCAAUCCAUUCCUAUUAAAAACGUAGGAGAGUCUCGCUUAGUCGAUCCUUUCCAACGUCAAUA
Hin-4-1   CAGGGAUA.CG...CU.GGAAACGGUUUAUCCUCUCCGUAUU.................................UA.GAAAGGUG.U.AA.AA.U.GCAAUCCAUUCCUAUUAAAAACGUAGGAGAGUCUCGCUUAGUCGAUCCUUUCCAACGUCAAUA
Hin-1-1   CAGGGAUA.CG...CU.GGAAACGGUUUAUCCUCUCCAUAUU.................................UA.GAAAGGUG.U.AA.AA.U.GCAAUCCAUUCCUAUCAAGAACGUAGGAGAGUCUCGCUUAGUCGAUCCUUUCCAACGUCAAUA
Hin-2-1   CAGGGAUA.CG...CU.GGAAACGGUUUAUCCUCUCCAUAUU.................................UA.GAAAGGUG.U.AA.AA.U.GCAAUCCAUUCCUAUCAAGAACGUAGGAGAGUCUCGUUUAGUCGAUCCUUUCCAACGCCAAUA
Pmu-1-1   CAGGGAUA.CG...CU.GGAAACGGUUUAUCCUCUCGUAUUU.................................UA.GAAAGGUG.U.GA.CA.U.GCAAACAAUUCCAAUUAAACAUGUUGGUAAUGAUCCUUUAGUGGAUGCGUUCCAACGUCGCUA
Mha-1-2   AAAGGAUA.AC...UU.AGAAAUGGGUUGUCCUCUCGUGUU..................................UA.GAAAGGUG.U.CA.AA.U.GCAGUCUAUUAUCCCAAUUAAAAACGUGGGUGAUGAUGUCGUUUCAGGUUUAGUUGAUCGCUU
Plu-1-1   CAGGAUGUGAG...GC.AGAAAUGCCCACGUCUCCCAUGUU..................................UG.GAAAGGUG.U.UA.UG.G.UGAAACAACUCACAGAUGCUUUUGCCCGCAAAUUUUACUAUUUGCGGCUAUCAAUCACCGAUG
Eca-1-1   CAGGGUGCGAG...GU.AGAAAUGCCUGCAUCUCCCGUAUU..................................UG.GAAAGGUG.U.UU.AU.GGUGAGUCAACUGACUGAUGCGUUUGCGCGCAAGUUUUACUAUUUGCGUCUGUCUAUCACAGACG
Spr-1-1   CAGGGUGCAGA...GC.GGAAACGCUCGCAUCUCCCGACUU..................................UG.G.AAGGUG.U.UA.UG.G.UGCCGCAACUCAUUGAUGCUUAUGAGCGCAAAUUCUAUUACUUGCGCCUGUCGAUCACCGACG
Sgl-1-1   CAGGGUGCAGG...GU.AGAAAUGCCGGCAUCUCCCGUAUU..................................UG.GAAAGGUG.U.UACUG.U.GUCACAACUCAUCGAUGCGUUUGAGCGCAGGUUUUAUUAUCUGCGCUUGUCAGUGACGGACGU
Ymo-1-1   CAGGGUGCGAG...GU.AGAAAUGCCUGCAUCUCCCGUAUU..................................UG.GAAAGGUG.U.UA.UG.G.UACAACUGACUGACGCAUUUGCGCGCAAGUUCUAUUACUUGCGCCUAUCGAUCACCGAUGUGU
Yps-2-1   CAGGGUGCAAG...GU.AGAAAUGCCUGCAUCUCCCGUAUU..................................UG.GAAAGGUG.U.UA.UG.G.UACAACUCACUGACGCAUUUGCGCGUAAAUUCUACUAUUUGCGCCUAUCGAUCACUGAUGUGU
Yps-1-1   CAGGGUGCAAG...GU.AGAAAUGCCUGCAUCUCCCGUAUU..................................UG.GAAAGGUG.U.UA.UG.G.UACAACUCACUGACGCAUUUGCGCGUAAAUUCUACUAUUUGCGCCUAUCGAUCACUGAUGUGU

          ..<<<<<<.<<...<<.<....>>>>>>>>>>><<.........................................>.>...>>>>.>.>>.>>.>.>>>>...........................................................
          ..202222.??...??.2....2????22220222.........................................2.2...12?2.?.??.??.?.????...........................................................
          CRGGGU●●-●●---●●-RGAAAYR●●Y●●RCCUCCC●●●●○----------------------------------UG-RAAAGG●G-●-●●-●●-●-●●●R●●●●●●●●●●●●●●●●●●●●●●●●●●●●●●●●●●Y●●●●●●●●●●●●●●●●●●●●●●●●
                          GNRA                                                            SD+++
```

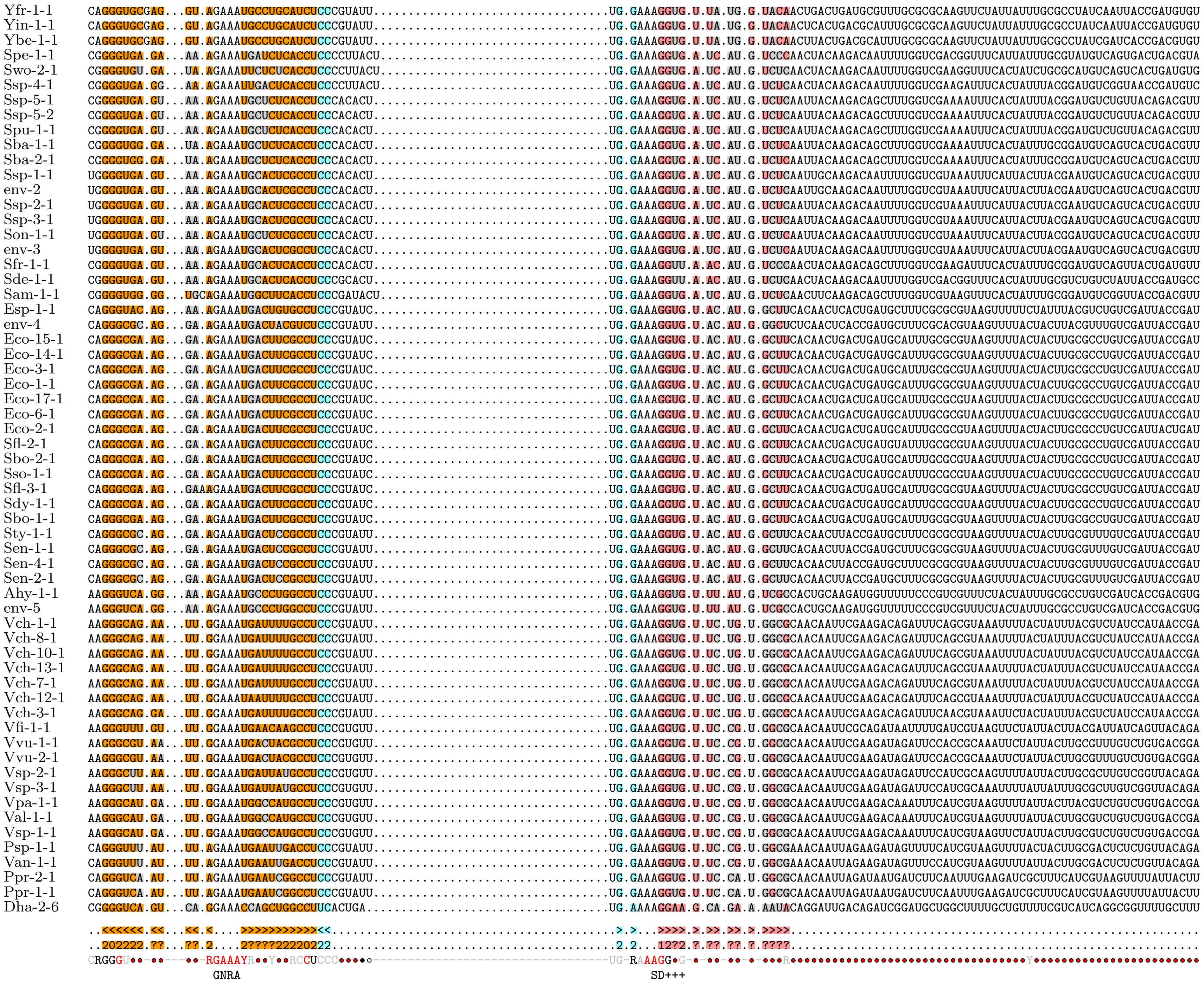

Yfr-1-1
Yin-1-1
Ybe-1-1
Spe-1-1
Swo-2-1
Ssp-4-1
Ssp-5-1
Ssp-5-2
Spu-1-1
Sba-1-1
Sba-2-1
Ssp-1-1
env-2
Ssp-2-1
Ssp-3-1
Son-1-1
env-3
Sfr-1-1
Sde-1-1
Sam-1-1
Esp-1-1
env-4
Eco-15-1
Eco-14-1
Eco-3-1
Eco-1-1
Eco-17-1
Eco-6-1
Eco-2-1
Sfl-2-1
Sbo-2-1
Sso-1-1
Sfl-3-1
Sdy-1-1
Sbo-1-1
Sty-1-1
Sen-1-1
Sen-4-1
Sen-2-1
Ahy-1-1
env-5
Vch-1-1
Vch-8-1
Vch-10-1
Vch-13-1
Vch-7-1
Vch-12-1
Vch-3-1
Vfi-1-1
Vvu-1-1
Vvu-2-1
Vsp-2-1
Vsp-3-1
Vpa-1-1
Val-1-1
Vsp-1-1
Psp-1-1
Van-1-1
Ppr-2-1
Ppr-1-1
Dha-2-6
GNRA
SD+++

```
Dha-1-6   CGGGGUCA.GU...CA.GGAAACCAGCUGGCCUUCACUGA.................................UG.AAAAGGAA.G.CA.GA.A.AAUACAGGAUUGACAGAUCGGAUGCUGGCUUUUGCUGUUUUCGUCAUCAGGCGGUUUUGCUUU
Dha-1-7   CAGGGUCG.GC...AU.AGAAAUGUACCGGCCUCCCGCAA.................................AG.G...GGUA.UCCU.GU.C.UGGGAUGCCUCAGGAAAGAAAGGUGCAAAGACCCAGAAUGAUGGGAUUUUAGGCGCAUUUUCG
Dha-1-8   CAGGGUCG.GC...AU.AGAAAUGUACCGGCCUCCCGCAAAGG.GGUAUCCUGUCUGGGAUGCCUC.........AG.GAAAGAAA.G.GU.GC.A.AAGACCCAGAAUGAUGGGAUUUUAGGCGCAUUUUCGAUUGUGAGCGAAAGUGCGCUUUUGCUA
Dha-2-7   CAGGGUCG.GC...AU.AGAAAUGUACCGGCCUCCCGUAAAUGCGGCAUCCUGUUCAGGAUACGGC.........AG.GAAAGAAA.G.GU.GC.A.AAGACCCAGUAUGAUGGGAUUUUAGGCGCAUUUUCGAUUGUGAGCGAAAGUGCGCUUUUGCUG
          ..<<<<<<.<<...<<.<....>>>>>>>>>>><<.....................................>.>...>>>>.>.>>.>>.>.>>>>.......................................................
          ..202222.??...??.2....2????22220222.....................................2.2...12?2.?.??.??.?.????.......................................................
          CRGGGU●●-●●---●●-RGAAAYR●●Y●●RCCUCCC●●●●○-------------------------------UG-RAAAGG●G-●-●●-●●-●-●●●R●●●●●●●●●●●●●●●●●●●●●●●●●●●●●●●●●●●●●●Y●●●●●●●●●●●●●●●●●●●●●●●●
                   GNRA                                                              SD+++
```

alignment positions 481 ··· 640

```
Aba-1-1   UCAACGACCUCCGGCUGUCCAUCACGGACCGCUGUAACUAUCGUUGUGUCUAUUGCCGCUCUGGCAACAAUGGGCCAUCCUUCGCCGAACUUCGCUUCCAAGAUUACGUCCGUAUUGCUCGCGUCUUCGUCUCCCUCGGAAUUACUAAGAUCCGCCUCAC
Tth-1-1   UGGUGGCGGCCUCGGACCUCCAGUACGCCCUCCCCGAGGUCGCCCGGGCCUUUGAGCGGAAAAACCCCGGGGUGAAGGUGGAGCUCGUCUUCGGCUCCUCCGGCAAGCUCUACGCCCAGCUCACCCAGGGCCUCGAGGCCGACCUCUUCUUCUCCGCCGA
Tth-2-1   UGGUGGCGGCCUCGGACCUCCAGUACGCCCUCCCCGAGGUCGCCCGGGCCUUUGAGCGGGAAAACCCCGGGGUGAAGGUGGAGCUCGUCUUCGGCUCCUCCGGCAAGCUCUACGCCCAGCUCACCCAGGGCCUCGAGGCCGACCUCUUCUUCUCCGCCGA
Sth-1-1   AGUAUCGGCGAAGCAGGUGAGGACAUGGACUUUUUCACGCUGAAAUCUGUCGAGGAGGCCCGGGUCGAGUUCCUCGGCGCCGUCACCCGCCGGAUGCCCGCGGAGCGGGUCGCAAGGGAGGAAGCCCUGGGGCGUAUCACCGCCGCCCCCGUGCCCGCUC
Csp-1-1   UGUGCAACUACCGCUGCACCUAUUGCCUGCCCGACGGCUUCAAGAAGUCGGGGACGAUGGCCUUCCUGACGCCCGAUGAAAUCGACCGCGUGGUGCGCGCCUUCGCCAGCUUCGGCGUCGCCAAGCUGCGUCUGACCGGCGGCGAGCCCAGCGUGCGGCG
Rsp-1-1   UUUUCGUUGUAGCUACUGUCUUCCCGAUGGCUAUCAGGGGAAACCCGAUGAAGCCUUUCUUUCGGCGUCCGAGCUGGAAGCUGCUGUGCGCGGUUUUGCGCAAAUGGGCACGCAAAAAAUUCGCCUGACCGGCGGUGAACCCGGUUUACGUUCGGACCUG
Ama-1-1   CAAUUUCCGCUGUAAUUACUGUCUUCCUGAUGGCAACGACUGCAACACACCCAAAGGUGGUUUGUCAGUGAAAGAAAUUCAAACCUUGGUGCAAGUGUUUGCCGGCUUGGGUACAACCAAAGUGCGUAUUACUGGUGGCGAACCAGCACUGCGCAAAGAU
Cps-1-1   UAGUUGCACUUACUGCUUGCCGGAUGGAUAUCAGUGCGAUCAACCGCGUGAUUUUUUAUCACUAUGUGAAAUCAAACGUAUAGCGAAAGCUUUCGCCGAGUUAGGAACUGAAAAGAUCCGCAUAACCGGUGGUGAACCAGCCCUACGUAAAGACUUACCU
Dha-2-1   GUGAAGGUGAGGGGAAUCUAUGCAAGAUCAAUAUCGGCGCCAGAUCGAAUACCUGAGGAUUUCCAUUACAGAUCGCUGCAAUUUACGAUGUAAAUAUUGCAUGCCCGCUGAAGGAGUCCAAUGGAUUCCUCAUGAGCGCAUCUUAACCUAUGAGGAAAUA
Dha-1-1   GUGAAGGUGAGGGGAAUCUAUGCAAGAUCAAUAUCGGCGCCAGAUCGAAUACCUGAGGAUUUCCAUUACAGAUCGCUGCAAUUUACGAUGUAAAUAUUGCAUGCCCGCUGAAGGAGUUCAAUGGAUUCCUCAUGAGCGCAUCUUAACCUAUGAGGAAAUA
Dha-2-2   UAUUCCAGGAAAAACAAAAGACGCCCGCUUUAGAAAGGGUCAUGUGAUCAAAGAAGAGGAUAUUCCCACCCUGCUUAGUAUGGGAAAAGAGCAUAUUUUUGUCUGGGAUCAGACUCCGGGUCUUAUUCACGAAGAUGAAGCGGCGGAGCGCCUGGCCAAG
Pat-1-1   UUCAUUACCUGCGGCUGUCCAUUACUGAUGUGUGUAAUUUUAGUUGUGAGUACUGUCUACCUGAUGGGUAUCAGUGCGAUACCGACAGGGAUUUCUUGUCUCUGAUUGAAAUUACACGCAUUGCAGGUGCCUUUGCGAAACUAGGCACCAGCAAAAUUCG
Pat-1-2   UGUAAUUUUAGUUGUGAGUACUGUCUACCUGAUGGGUAUCAGUGCGAUACCGACAGGGAUUUCUUGUCUCUGAUUGAAAUUACACGCAUUGCAGGUGCCUUUGCGAAACUAGGCACCAGCAAAAUUCGUAUAACGGGUGGCGAGCCUUCGCUACGAAAAG
Swo-1-1   GAGCUGUUUCCUGCCUGAAGAUGAGGAUAAAGCCAGCAUCAACUUUUCGAUGCUGGCUUUUAUUUCCCCGUUGUUCAUAGAAUCACCCUCAUAAAAGCGAGACGAGGUCUGUUAUGAAAUUUACGGAAAGGAGGUGCUGAUGAAAGACCUCGCUGAAAUA
Mth-1-1   AGCCGUUUAAACGGCCCUGCUACGAACGAAAACCCCGGGCUGGUAUGUGCUGGUAGGAGGCGAUCCCGGCUCGAAGGCAAACAGGGCCAGCCAAGGAGGAGUUAUUUUAGAGGAGGCUUUUUAUCAUGGUCAACCAGGACGCCAUUCGAGCGGCGGUAAA
Mth-1-2   CGGCGCCAGGGGGCCGUAAUGGCAAUUUAAGGCGGAUAGGGCCUCACAGUGGCUGACGCCCCCGCCAGUGAAGAAUGAAAAUGUGGGCUGAGGACCAUUUUGCCAGAAGUAUAUUUUGGGAGGAGCCCAAGAGUAUGAAAUUACGGCGCGCAUCAAUAUU
Dha-1-3   GAUAAGCCUAUCAGUAGGACCCGGUGGAGUGCUCCUGCUCUGAUUUUAAAGGAGGGUAAAUUAUGAUGAAAGAAUUCUUAAGCCGUAAAAGAACGCAGUUUUUUUCAGUUCUCAUGGUGGCGGUUUUGAUGAUCGCUUCCUUUGUUCCUUCAGCCUUCGC
Mth-1-3   GUGGUGCUAAAUGGCAGAGCGAGGAUAAGGAGGCAAAGCUAUGUGGGAAGUCUAUGAUGAAUUAAUCGCGGCGGUGCCGCCGGAUCUGGAGGUAGAAGAUUGCAUCGUCGGCCUUAACUGGAUCCUGGUCCGUUCCCGGGCUACCGGCCUGGUGAUGACG
Dha-1-4   UUAAAUUUUAAAACUGUUAGGUGGUGAAUUUCAUUGGCAAGUAUAAAAAUGAAACAAGUCAUGGAAUUUUUUGCGGAGAACUAUGAUAUAAACCUCAAUGAUGCUGAAGAAUAUUUCAUGGCCAUUGUUUCUGUCGGAUUGUCUUUAAAUAAGGAAAUGC
Dha-2-3   UUUAAAUUUUAAAACUGUUAGGUGGUGAAUUUCAUUGGCAAGUAUAAAAAUGAAACAAGUCAUGGAAUUUUUUGCGGAGAACUAUGAUAUAAACCUCAAUGAUGCUGAAGAAUAUUUCAUGGCCAUUGUUUCUGUCGGAUUGUCUUUAAAUAAGGAAAUG
Dha-2-4   UUAACGUUUAAAGAUCGGUUGGACUGGAGCCCGCCAACAUGGAGAAAGAAGGUUGGUCAACAAUGGGAUUGAAUUGUAUGAAAAUGAUGGGGAUACCGACUUUUGCGGAAAGACCCGAUGCCGAAUAUUUGUUUUUUCCCGGAUGUAAUCCCUUUGCUGA
Dha-2-5   GAUCGGUUGGACUGGAGCCCGCCAACAUGGAGAAAGAAGGUUGGUCAACAAUGGGAUUGAAUUGUAUGAAAAUGAUGGGGAUACCGACUUUUGCGGAAAGACCCGAUGCCGAAUAUUUGUUUUUUCCCGGAUGUAAUCCCUUUGCUGACAGGUCAAAAGG
Mth-1-4   UAAUUUGUUGCUGAUUAGCCAGGAAAGGGGAUAGCUUUAGAUGCAGUUCUUAGAAGGCAUCAGAGAAAAAUUUUACAGGCUGGUAAAAGACAGGCACCUGAGCGGUGAGGAAGAAAUCCAGGUGGUCAGCGCCCGGCCCCUGACCCCCGAAGAAGCCAUU
Cpe-2-1   UAAGGUCUAAAAGGACUAUUUAAUUUAAUGCUUAUUAUUCUUGUUGGUAUAACAAAAAUAAUAAGCAUUUUAUUUUAUAAAGGGAUGGUGAUAUUUUGAAAAAAUUUAUAGCUUUAGAAGAAGCUCUUGAAAUAUUAAAUAAGAACACUAAGGCUUUAAA
Cpe-1-1   UAAGGUCUAAAAAGACUAUUUAAUUUAAUGCUUAUUAUUCUUGUUGUUAUAACAAAAAUAAUAAGCAUUUUAUUUUAUAAAGGGAUGGUGAUAUUUUGAAAAAAUUUAUAGCUUUAGAAGAAGCUCUUGAAAUAUUAAAUAAGAACACUAAGGCUUUAAA
Cpe-3-1   UAAGGUCUAAAAGGACUAUUUAAUUUAAUGCUUAUUAUUCUUGUUGGUAUAACAAAAAUAAUAAGCAUUUUAUUUUAUAAAGGGAUGGUGAUAUUUUGAAAAAAUUUAUAGCUUUAGAAGAAGCUCUUGAAAUAUUAAAUAAGAACACUAAGGCUUUAAA
Pin-1-1   GAUGUCUGUAAUUUUAAAUGUAAUUAUUGUCUGCCCGAUGGAUAUCAGCGAACCCAUGAAAAACAGUUUUUGAGCAAGCAGGAGAUAACCAACUUAGUCAAUGCUUUCGCCGAACUGGGUGCUAAAAAAGUACGUAUAACAGGUGGAGAACCCGCUUUAC
Apl-1-1   UGGUUUUAUUAAAAAUACCUCUCUCGGUCUUGCUGGGACAUCUUUAGCGGGCGGUAGUGUAAAUGCCUUAAUUUCAACGGAAGCGGCCGCAGCGGAGAUGCAAACCGUAAUGACAGCAGCACACUGGGGGGCGUUAGGUAUCGUGGUUGAAAACGGUAAA
Hdu-1-1   AGGUUUUAUUAAAAAUACUUCGUUUGGUCUUGCAGGGACAUCUUUAGCCGGUGGUAGCGUAAAUGCAUUGCUUACAACAGAAGCCGUUGCCGCAGAAAUGCAAACUGUCGUUACUGCCGCACAUUGGGGCGCAUUAGGUGUCGUAGUUGAAAACGGUAAA
Mha-1-1   CGGUUUUCUAAAAAAUUCAUCAUUAAGCUUAGCUGCCGGUGCAGGUUUAGCCAGUGGUACUACCGGUGUAGUGGGGGCAUUAGCUUCCACCAGUGCCAAAGCAGCCGAGAUGAAAACAGUAGUAACUGCCGCCCACUGGGGGCCUUUAGGUGUAGUGGUA
Apl-1-2   ACGACAAUAUGUUUAUUUACGUUUAUCCGUUACCGAUGUCUGCAAUUUUCGCUGUAAUUAUUGUCUGCCGGACGGCUAUAAGCCUCCUUCACAUAGGCAGCAAUUUUUAAGCAUAUCCGAAAUUCAACGUGUCGUGCGUGCUUUUGCUGAUCUCGGCACG
Hso-2-1   CUUUCACCAGUUCAUUGUGAGCCUAGUAAAUAACGAGGUUUAAAAUGAAAACACAAAAUAAAGUUAAUACAAGUCGCCGUGAUUUUUUAAAAAAUUCAUCAUUAGGCGUGGCUGGCGUAUCUCUAAGUGGCGGUGUUGUCGGAACAUUAGCUUCGAAAGC
Hso-1-1   CUUUCACCAGUUCAUUGUGAGCCUAGUAAAUAACGAGGUUUAAAAUGAAAACACAAAAUAAAGUUAAUACAAGUCGCCGUGAUUUUUUAAAAAAUUCAUCAUUAGGCGUGGCUGGCGUAUCUCUAAGUGGCGGUGUUGUCGGAACAUUAGCUUCGAAAGC
Asu-1-1   UUAUCUGCGCCUGUCGAUUACGGACGUGUGUAAUUUCCGUUGUAAUUACUGUCUGCCCGACGGCUAUCAUCCCUCGCACGAGCGCGAUAAAUUUCUCACAGUGGAUGAAAUUCGGCGUGCGGUAUCUGCUUUUGCGGCAAUGGGAGCGCAAAAAGUGCGG
Msu-1-1   CUAUUAUUUGCGCCUGUCCGUAACGGAUGUGUGCAAUUUCAAGUGUACCUAUUGUUUACCUAGUGGGUAUCAGCCUCCUGUGCAAAAAGAAAGUUUUUUGAGCUUGGAUGAAAUACGCCGUAUUGUCGGUGCUUUUGCGGCAAUGGGAACGGAAAAAGUG
Hso-1-2   UACUAUUAUUUACGCUUGUCUGUCACGGAUAUGUGUAAUUUUCGUUGUAACUAUUGUUUACCUCAUGGCUAUCGAUCGGAAAGCACAAAACCCAGUUUUCUGAAUCUGGUUGAAAUUAAACGAUUGGUGAAUGCCUUUGCACAAUUAGGUACAGAAAAAG
Hso-2-2   UACUAUUAUUUACGCUUGUCUGUCACGGAUAUGUGUAAUUUUCGUUGUAACUAUUGUUUACCUCAUGGCUAUCGAGCGGAAAGCACAAAACCCAGUUUUCUGAAUCUGGUUGAAAUUAAACGAUUGGUGAAUGCCUUUGCACAAUUAGGUACAGAAAAAG
Hin-3-1   UUACUAUCUACGGCUGUCGAUUACCGAUCAGUGUAAUUUUCGUUGUACUUAUUGUUUACCUGAUGGUUAUCAACCCGAAGCUAACAAACCAAGUUUCUUAACUUUAAAAGAAAUCACCCAUCUUGCUCAAGCGUUUGCUGAAAUGGGCACAGAGAAAAUC
Hin-4-1   UUACUAUCUACGGCUGUCGAUUACCGAUCAGUGUAAUUUUCGUUGUACUUAUUGUUUACCUGAUGGUUAUCAACCCGAAGCUAACAAACCAAGUUUCUUAACUUUAAAAGAAAUUACCCAUCUUGCUCAAGCCUUUGCUGAAAUGGGCACAGAGAAAAUC
Hin-1-1   UUACUAUCUACGACUGUCGAUUACUGAUCAGUGUAAUUUUCGUUGUACCUAUUGUUUACCUGAUGGUUAUCAACCCGAAGCUAACAAACCAAGUUUCUUAACCUUAAAAGAAAUUAUCCAUCUUGCUCAAGCGUUUGCUGAAAUGGGCACAGAGAAAAUC
Hin-2-1   UUACUAUCUACGACUGUCGAUUACCGAUCAGUGUAAUUUUCGUUGUACUUAUUGUUUACCUGAUGGUUAUCAACCCGAAGCUAACAAACCAAGUUUCUUAACUUUAAAAGAAAUUACCCAUCUUGCUCAAGCGUUUGCUGAAAUGGGUACAGAAAAAAUC
Pmu-1-1   UUACUAUUUGCGACUGUCAAUUACAGAUGUGUGUAAUUUCCGCUGUAAUUACUGUUUGCCGAAUGGUUAUCAACCUGAGUCAAAUAAACCCAGUUUUCUCACCUUAAAUGAAAUUCGUUUAGUGGUAAAAAGUUUUGCUAACAUGGGUACUGAAAAAGUC
Mha-1-2   UCAGCGUCAAUAUACUUAUUUGCGACUAUCCAUUACUGAUGUUUGUAACUUCCGUUGUAAUUAUUGCUUGCCUGAUGGCUAUAAGCCGCCUUCACAUAAGCAAACUUUUCUUAGCGUUGAUGAAAUCCAGCGGGUGGCUAAAGCCUUUGCCAAUUUAGGG
Plu-1-1   UAUGUAAUUUUCGGUGCACUUAUUGUUUGCCGGAUGGUUAUAAACCCCGUGGACAUCACUCCUUUUUGACAUUACCCGAAAUCCGUCUUGUUGGCCGGGCAUUUGCCGAUCUGGGUACCGAGAAAAUCCGUUUGACGGGGGGUGAACCGACCAUGCGCCG
Eca-1-1   UCUGCAAUUUUCGCUGUACCUACUGCCUGCCGGAUGGUUAUCAAGCCAAUGGCACCAAUCCACAUCGCUUUUUAUCACUCGAUGAGAUUCGUCGCGUCAGCCGCGCUUUUGCUGAAUUGGGGACGGAAAAAGUCCGUCUUACCGGCGGCGAACCGUCUCU
Spr-1-1   UGUGUAACUUUCGUUGCACCUAUUGUUUGCCGGAUGGCUAUCGCCCUAACGGCAGCCCGAAAACCUUCUUGUCGCUCGAUGAGAUCCGACGUGUCAGCCGCGCCUUCGCCGAGCUGGGCACUGAAAAGGUGCGCCUGACCGGCGGUGAGCCUUCGCUGCG
Sgl-1-1   CUGUAAUUUCCGCUGCACCUAUUGCCUGCCCGACGGUUAUCAUCCGCAGGGAACCCGCAAUACCGGCUUUCUCUCCUUGGCCGAGAUCCGUCGCGUCACCCGCGCCUUUGCCGAACUGGGGACAGAAAAAGUCCGCCUCACCGGCGGCGAACCGUCGCUG
Ymo-1-1   GCAACUUUCGCUGCACCUACUGUCUGCCGGAUGGCUAUCGGCCCGAUGGACUAAAAAGCUUUCUGAGCCUCGACGAAAUAAGCCGCGUUAGCCGCGCCUUUGCCCUGUUAGGUACUGAAAAAAUCCGCCUGACGGGCGGUGAACCCUCUAUGCGGCGUGA
Yps-2-1   GCAAUUUCCGUUGCACAUAUUGCUUGCCAGAGGGCUACCGCCCAGAUGGUGUCAAAAGUUUUUUGAGCCUUGACGAAAUCAAUCGCGUCAGCCGCGCCUUUGCCUUGUUGGGAACGGAAAAAAUCCGCCUGACGGGGGGCGAGCCUUCGAUGCGGCGCGA

          ................................................................................................................................................................
          ................................................................................................................................................................
          ●●●●●●●●●●●●●●●●●●●●●●●●●●●●●●●●●●●●●●●●●●●●●●●●●●●●●●●●●●●●●●●●●●●●●●●●●●●●●●●●●●●●●●●●●●●●●●●●●●●●●●●●●●●●●●●●●●●●●●●●●●●●●●●●●●●●●●●●●●●●●●●●●●●●●●●●●●
```

Yps-1-1	GCAAUUUCCGUUGCACAUAUUGCUUGCCAGAGGGCUACCGCCCAGAUGGUGUCAAAAGUUUUUGAGCCUUGACGAAAUCAAUCGCGUCAGCCGCGCCUUUGCCUUGUUGGGAACGGAAAAAAUCCGCCUGACGGGGGGCGAGCCUUCGAUGCGGCGCGA
Yfr-1-1	GCAAUUUUCGUUGCACUUAUUGCCUGCCCGAUGGCUAUCGCCCGGACGGAUUAAAAAGUUUUCUAAGCCUUGAUGAGAUAAGCCGCAUCAGCCGCGCUUUUGCUUUGCUGGGUACAGAAAAGAUCCGUCUGACCGGUGGUGAGCCCUCCAUGCGCCGCGA
Yin-1-1	GCAAUUUUCGUUGCACCUAUUGCUUGCCGGAUGGCUAUCGCCCCGACGGAUUAAAAAGCUUUCUGAGUCUGGACGAAAUAAGCCGCGUCAGCCGCGCCUUUGCUCUAUUGGGAACAGAAAAAAUUCGUCUGACGGGCGGCGAACCCUCUAUGCGGCGCGA
Ybe-1-1	GCAACUUUCGCUGCACCUACUGUCUGCCCGAUGGCUAUCGCCCCGACGGGCUAAAAAGUUUUCUGAACCUUGACGAGAUAAGCCGCGUUAGCCGCGCCUUUGCUCUGUUGGGAACGGAAAAAAUCCGCCUGACCGGUGGUGAACCCUCUAUGCGGCGUGA
Spe-1-1	UGUAACUUUAAGUGCACGUAUUGCCUCCCAGAUGGCUACCGCCCAGACGGUAAGCCUAAAUUCCUCGAUCUCAAUGAAAUCGAGCAUCUGGUUUCGGCUUUUUCACAAGUAGGCACCCAAAAAAUCCGUAUUACCGGUGGUGAGCCUUCUUUGCGUAAAG
Swo-2-1	UGUAAUUUCAAAUGUACUUACUGCCUCCCCGAUGGCUAUCGACCUAAUGGUCGUUCUAAGUUUUUGGCGCUUAGCGAGAUUGAAAACUUAGUCGCAGCUUUUGCUGAAGUCGGUACUCAAAAAAUUCGUAUCACUGGCGGCGAACCGACGCUGCGUAAAG
Ssp-4-1	UGUAACUUCAAAUGCACAUACUGCCUCCCAGAUGGUUAUCGCCCCGACGGUAAGCCUAAGUUUUUAGAUCUCAACGAGAUCGAGAAUCUAGUGGGCGCCUUUGCCGAGGUCGGCACCCAGAAGAUCCGCAUCACGGGCGGCGAGCCCACAUUGCGUAAAG
Ssp-5-1	UGUAACUUCAAAUGCAGCUACUGUCUCCCCGAUGGUUAUCAUCCUGAUGGUAAACCAAAAUUCUUAUCUCUUAAUGAGAUAGAAAACCUCGUGUCUGCAUUU...
Ssp-5-2	UGUAACUUCAAAUGCAGCUACUGUCUCCCCGAUGGUUAUCAUCCUGAUGGUAAACCAAAAUUCUUAUCUCUUAAUGAGAUAGAAAACCUCGUGUCUGCAUUUAGCCAAGUGGGUACUCAAAAAAUCCGCAUAACGGGCGGUGAGCCUACCCUACGUAAAG
Spu-1-1	UGUAACUUCAAAUGUAGCUACUGUCUCCCCGAUGGUUAUCAUCCUGAUGGUAAACCAAAAUUCUUAUCUCUUAAUGAGAUAGAAAACCUCGUGUCUGCAUUUAGCCAAGUGGGUACUCAAAAAAUCCGCAUAACGGGCGGUGAGCCUACCCUACGUAAAG
Sba-1-1	UGUAACUUCAAAUGCAGUUAUUGCCUCCCCGACGGUUAUCAUCCUGACGGUAAGCCAAAAUUUUUAUCUCUCAAUGAGAUAGCAAACUUAGUCUCUGCAUUUAGCCAAGUCGGCACCCAAAAAAUCCGCAUUACAGGCGGUGAACCCACCCUGCGUAAAG
Sba-2-1	UGUAACUUCAAAUGCAGUUAUUGCCUCCCCGACGGUUAUCAUCCUGACGGCAAGCCAAAAUUCUUAUCUCUCAAUGAGAUAGAAAACUUAGUCUCUGCAUUUAGCCAAGUCGGCACCCAAAAAAUCCGCAUUACAGGCGGUGAACCCACCCUGCGUAAAG
Ssp-1-1	UGUAACUUCAAAUGCAGUUAUUGCCUCCCCGAUGGUUAUCAUCCGAAUGGCAAACAACAGUUUUUAUCUCUCAGUGAGAUAGAAAACCUUGUGUCCGCAUUUAGCCAGGUUGGCACUCAAAAAAUCCGUAUCACUGGCGGCGAACCCACGCUGCGCAAAG
env-2	UGUAACUUCAAAUGCAGCUAUUGCCUCCCGGAUGGUUAUCAUCCGAAUGGCAAACAACAGUUUCUAUCUCUCAGUGAGAUAGAAAACCUUGUGUCCGCAUUUAGCCAGGUUGGCACUCAAAAAAUCCGUAUCACUGGCGGCGAACCCACGCUGCGUAAAG
Ssp-2-1	UGUAACUUCAAAUGCAGUUAUUGUCUCCCCGAUGGUUAUCAUCCGAAUGGUAGACAACAGUUCUUAUCUCUCAGUGAGAUAGAAAAUCUUGUGUCCGCAUUUAGCCAGGUUGGCACUCAAAAAAUCCGUAUCACUGGCGGCGAACCCACGCUGCGUAAAG
Ssp-3-1	UGUAACUUCAAAUGCAGUUAUUGUCUCCCCGAUGGUUAUCAUCCGAAUGGUAGACAACAGUUCUUAUCUCUCAGUGAGAUAGAAAAUCUUGUGUCCGCAUUUAGCCAGGUUGGCACUCAAAAAAUCCGUAUCACUGGCGGCGAACCAACGCUGCGUAAAG
Son-1-1	UGUAACUUCAAAUGCAGUUAUUGCCUCCCCGAUGGUUAUCAUCCCAAUGGCAAGCAGCAGUUUUUAUCUCUCAGUGAGAUAGAAAACUUGGUGUCCGCAUUUAGCCUCGUUGGCACUCAAAAAAUCCGUAUCACUGGCGGCGAACCCACGCUGCGUAAAG
env-3	UGUAACUUCAAAUGCAGCUAUUGCCUCCCCGAUGGUUAUCAUCCGAAUGGCAAGCAACAGUUUUUAUCUCUCAGUGAGAUAGAAAACUUGGUGUCCGCAUUUAGCCUCGUUGGCACUCAAAAAAUCCGUAUUACUGGCGGCGAACCCACCCUGCGUAAAG
Sfr-1-1	UGCAAUUUUAAAUGUACUUAUUGUUUACCCGACGGAUAUCGUCCAGAUGGUAAACCUACCUUCUUAACGCUCAACGAGAUUGAAAAUCUGGUUGCCGGUUUCGGUGAAGUAGGCACACAAAAAGUUCGUAUCACUGGUGGCGAGCCCACCUUACGCAAGG
Sde-1-1	UGUAAUUUUAAAUGUACCUAUUGUUUACCCGAUGGCUAUCAAAGCCAAGGAAAUAGCCCGUUUUUGUCCUUAAGUGAGAUAGAGCUGCUAUUGGGGGCAUUUUCUCAAAUGGGCACUCAAAAAGUGCGCAUUACCGGCGGUGAACCUAGCCUGCGCAAAG
Sam-1-1	UGCAACUUCAAAUGCAGCUAUUGUCUGCCCGACGGCUACCACCCGGAUGGUAGGCCGAGUUUUCUGACCCUCAGUGAAAUCGAAAAUCUGAUUGCCGCCUUUGCCAAGGCCGGUACCCGCAAGGUGCGCAUCACUGGCGGUGAGCCAACCCUGCGCAAAG
Esp-1-1	GUGUGCAACUUCCGUUGCACCUAUUGCCUGCCGAACGGUUACAAGCCCGGCAGCGUCACCAAUAACGGAUUUCUCUCCGUGGCAGAAGUGCGGCGCAUCACGCGCGCAUUCUCUGAACUCGGCACCGAAAAAGUGCGCCUGACGGGCGGAGAACCCUCAC
env-4	GUGUGUAACUUUCGUUGCACCUACUGCCUGCCGGAUGGCUAUAAGCCCGGUGGGGUCACCAAUAACGGCUUUCUGACCGUUGAUGAAAUUCGUCGCGUCACGCGCGCGUUCGCCAGUCUGGGAACGGAAAAAGUGCGUCUGACAGGGGGCGAACCUUCAC
Eco-15-1	GUGUGUAACUUUCGUUGCACCUACUGCCUGCCGGAUGGCUACAAACCGAGCGGCGUCACCAAUAAAGGCUUUCUGACCGUCGAUGAAAUUCGCCGGGUUACGCGCGCCUUCGCCAGUCUGGGCACCGAAAAAGUGCGCCUGACAGGCGGUGAGCCGUCUU
Eco-14-1	GUGUGUAACUUUCGUUGCACCUACUGCCUGCCGGAUGGCUACAAACCGAGCGGCGUCACCAAUAAAGGCUUUCUGACCGUCGAUGAAAUUCGCCGGGUUACGCGCGCCUUCGCCAGUCUGGGCACCGAAAAAGUGCGCCUGACAGGCGGUGAGCCGUCUU
Eco-3-1	GUGUGUAACUUUCGUUGCACCUACUGCCUGCCGGAUGGCUACAAACCGAGCGGCGUCACCAAUAAAGGCUUUCUUACCGUCGAUGAAAUUCGCCGGGUUACGCGCGCCUUCGCCAGUCUGGGCACCGAAAAAGUGCGCCUGACAGGAGGAGAGCCGUCUU
Eco-1-1	GUGUGUAACUUUCGUUGCACCUACUGCCUGCCGGAUGGGUACAAGCCGAGCGGCGUCACCAAUAAAGGCUUUCUAACCGUCGAUGAAAUUCGCCGGGUUACGCGCGCCUUCGCCAGUCUGGGCACCGAAAAAGUGCGCCUGACAGGCGGUGAGCCGUCUU
Eco-17-1	GUGUGUAACUUUCGUUGCACCUACUGCCUGCCGGAUGGCUACAAACCGAGCGGCGUCACCAAUAAAGGCUUUCUUACCGUCGAUGAAAUUCGCCGGGUUACGCGCGCCUUCGCCAGACUGGGCACCGAAAAAGUGCGCCUGACAGGAGGAGAGCCGUCUU
Eco-6-1	GUGUGUAACUUUCGUUGCACCUACUGCCUGCCGGAUGGCUACAAACCGAGCGGCGUCACCAAUAAAGGCUUUCUUACCGUCGAUGAAAUUCGCCGGGUUACGCGCGCCUUCGCCAGUCUGGGCACCGAAAAAGUGCGCCUGACAGGCGGUGAGCCGUCUU
Eco-2-1	GUGUGUAACUUUCGUUGCACCUACUGCCUGCCGGAUGGUUACAAGCCGAGCGGCGUCACCAAUAAAGGCUUUCUGACCGUCGAUGAAAUUCGCCGGGUUACGCGCGCCUUCGCCAGUCUGGGCACCGAAAAAGUCCGUCUGACAGGUGGUGAGCCAUCUU
Sfl-2-1	GUGUGUAACUUUCGUUGCACCUACUGCCUGCCGGAUGGCUACAAACCGAGCGGCGUCACCAAUAAAGGCUUUCUUACCGUCGAUGAAAUUCGCCGGGUUACGCGCGCCUUCGCCAGUCUGGGCACCGAAAAAGUGCGCCUGACAGGAGGAGAGCCGUCUU
Sbo-2-1	GUGUGUAACUUUCGUUGCACCUACUGCCUGCCGGAUGGCUACAAACCGAGCGGCGUCACCAAUAAAGGCUUUCUUACCGUCGAUGAAAUUCGCCGGGUUACGCGCGCCUUCGCCAGUCUGGGCACCGAAAAAGUGCGCCUGACAGGAGGUGAGCCGUCUU
Sso-1-1	GUGUGUAACUUUCGUUGCACCUACUGCCUGCCGGAUGGCUACAAACCGAGCGGCGUCACCAAUAAAGGCUUUCUUACCGUCGAUGAAAUUCGCCGGGUUACGCGCGCCUUCGCCAGUCUGGGCACCGAAAAAGUGCGCCUGACAGGAGGAGAGCCGUCUU
Sfl-3-1	GUGUGUAACUUUCGUUGCACCUACUGCCUGCCGGAUGGCUACAAACCGAGCGGCGUCACCAAUAAAGGCUUUCUUACCGUCGAUGAAAUUCGCCGGGUUACGCGCGCCUUCGCCAGUCUGGGCACCGAAAAAGUGCGCCUGACAGGAGGAGAGCCGUCUU
Sdy-1-1	GUGUGUAACUUUCGUUGCACCUACUGCCUGCCGGAUGGCUACAAACCGAGCGGCGUCACCAAUAAAGGCUUUCUGACCGUCGAUGAAAUUCGCCGGGUUACGCGCGCCUUCGCCAGUCUGGGCACCGAAAAAGUCCGUCUGACAGGUGGUGAGCCGUCUU
Sbo-1-1	GUGUGUAACUUUCGUUGCACCUACUGCCUGCCGGAUGGCUACAAACCGAGCGGCGUCACCAAUAAAGGCUUUCUUACCGUCGAUGAAAUUCGCCGGGUUACGCGCGCCUUCGCCAGUCUGGGCACCGAAAAAGUGCGCCUGACAGGAGGUGAGCCGUCUU
Sty-1-1	GUGUGUAACUUUCGUUGCACCUACUGUCUGCCGGAUGGCUACAAACCUGGCGGCGUCACUAACAACGGUUUCCUUACCGUCGAUGAAAUUCGUCGCGUUACGCGCGCUUUUGCCAGUCUUGGCACGGAGAAGGUGCGUCUUACCGGCGGCGAGCCUUCAU
Sen-1-1	GUGUGUAACUUUCGUUGCACCUACUGUCUGCCGGAUGGCUACAAACCUGGCGGCGUCACUAACAACGGUUUCCUUACCGUCGAUGAAAUUCGUCGCGUUACGCGCGCUUUUGCCAGUCUUGGCACGGAAAAGGUGCGUCUUACCGGCGGCGAGCCUUCAU
Sen-4-1	GUGUGUAACUUUCGUUGCACCUACUGCCUGCCGGAUGGCUACAAACCUGGCGGCGUCACUAACAACGGUUUCCUUACCGUCGAUGAAAUUCGUCGCGUUACGCGCGCUUUUGCCAGUCUUGGCACGGAGAAGGUGCGUCUUACCGGCGGCGAGCCUUCAU
Sen-2-1	GUGUGUAACUUUCGUUGCACCUACUGUCUGCCGGAUGGCUACAAACCUGGCGGCGUCACUAACAACGGUUUCCUUACCGUCGAUGAAAUUCGUCGCGUUACGCGCGCUUUUGCCAGUCUUGGCACGGAAAAGGUGCGUCUUACCGGCGGCGAGCCUUCAU
Ahy-1-1	UGCAAUUUUCGCUGUACCUAUUGCCUGCCCGAUGGUUACCGUCCACCGGCCGGUGGCAAGGUGGGGCGUCAGCCCUUCCUCUCUCUGGAAGAGAUACGCCGUGUUGUCUCCGGAUUUGCCGCCAUGGGCACCCGCAAGGUGCGCCUGACCGGGGGGGAGC
env-5	UGCAAUUUUCGCUGUACCUACUGCCUGCCCGAUGGUUACCGCCCACCGGCCGGUGGCAAGGCGGGGCGUCAGCCCUUUCUCUCUUUGGAAGAGAUCCGCCGUGUCGUGUCCGGAUUUGCCGCCAUGGGCACCCGCAAGGUGCGCCUGACCGGUGGGGAGC
Vch-1-1	CGUUUGUAAUUUCAAAUGUACGUAUUGCUUACCUGAUGGUUACAAGCCAUCAGCGGAUCGCCCUGCCUCUUUUCUGACGGUGAAUGAGAUUCGCCGAGUGGUGAGUGCGUUUGCCCAUUGUGGCACUUCCAAGGUCAGAAUUACCGGCGGUGAACCUUCA
Vch-8-1	CGUUUGUAAUUUCAAAUGUACGUAUUGCUUACCUGAUGGUUACAAGUCAUCAGCGGAUCGCCCUGCCUCUUUUCUGACGGUGAAUGAGAUUCGCCGAGUGGUGAGUGCGUUUGCCCAUUGUGGCACUUCCAAGGUCAGAAUUACCGGCGGUGAACCUUCA
Vch-10-1	CGUUUGUAAUUUCAAAUGUACGUAUUGCUUACCUGAUGGUUACAAGCCAUCAGCGGGUCGCCCUGCCUCUUUUCUGACGGUGAAUGAGAUUCGCCGAGUGGUGAGUGCGUUUGCCCAUUGUGGUACUUCCAAGGUCAGAAUUACCGGCGGUGAACCUUCA
Vch-13-1	CGUUUGUAAUUUCAAAUGUACGUAUUGCUUACCUGAUGGUUACAAGCCAUCAGCGGAUCGCCCUGCCUCUUUUCUGACGAUGAAUGAGAUUCGCCGAGUGGUGAGUGCGUUUGCCCAUUGUGGCACUUCCAAGGUCAGAAUUACCGGCGGUGAACCUUCA
Vch-7-1	CGUUUGUAAUUUCAAAUGUACGUAUUGCUUACCUGAUGGUUACAAGCCAUCAGCGGAUCGCCCUGCCUCUUUUCUGACGGUGAAUGAGAUUCGCCGAGUGGUGAGUGCGUUUGCCCAUUGUGGCACUUCCAAGGUCAGAAUUACCGGCGGUGAACCUUCA
Vch-12-1	CGUUUGUAAUUUCAAAUGUACGUAUUGCUUACCUGAUGGUUACAAGCCAUCAGCGGAUCGCCCUGCCUCUUUUCUGACGGUGAAUGAGAUUCGCCGAGUGGUGAGUGCGUUUGCCCAUUGUGGCACUUCCAAGGUCAGAAUUACCGGCGGUGAACCUUCA
Vch-3-1	CGUUUGUAAUUUCAAAUGUACGUAUUGCUUACCUGAUGGCUAUAAGCCGUCAGCGGAUCGUCCUGCCUCUUUUCUGACCGUCAACGAAAUUCGUCGUGUGGUGAAUGCGUUUGCCCAUUGUGGCACUUCAAAGGUGAGAAUCACCGGCGGUGAACCUUCG
Vfi-1-1	UGUCUGUAACUUUAAAUGUACUUAUUGCUUACCUGAUGGUUAUAAACCAGAAAAUGGAAAGCGUGAGUCCUUCCUUGAACUUGAUGAAAUCGAACGUACUGUUAGUGCGUUUGCUCGUUGUGGUACGUCUAAAGUACGUAUUACAGGGGGAGAACCAAGU
Vvu-1-1	CGUGUGUAAUUUCAAAUGUACCUAUUGUCUGCCUGAUGGUUACCAACCUUCGGGGCAGAAAAACUCGUCUUUUUUAAACCUCUCUGAAAUCCGUCGAGUCGUUAAGGCUUUUGCUGACUGUGGCACAAGUAAAGUGCGCAUUACUGGCGGAGAGCCAAGU
Vvu-2-1	CGUGUGUAAUUUCAAAUGUACCUAUUGUCUGCCUGAUGGUUACCAACCUUCGGGGCAGAAAAACUCGUCUUUUUUAAACCUCUCUGAAAUCCGUCGAGUCGUUAAGGCUUUUGCUGACUGUGGCACAAGUAAAGUGCGUAUUACUGGCGGAGAGCCAAGU
Vsp-2-1	CGUCUGUAACUUUAAAUGUACUUACUGCUUGCCAGAUGGUUAUAAACCGUCAGGGCAAAAAAACUCGUCUUUUUUAAGUGUUCCUGAGAUCAAACGCGUUGUUAAAGCGUUUGCUGAUUGUGGUACCUCAAAGAUCCGCAUUACUGGCGGAGAGCCGAGU
Vsp-3-1	CGUCUGUAACUUUAAAUGUACUUACUGCUUGCCAGAUGGUUAUAAACCGUCAGGGCAAAAAAACUCGUCUUUUUUAAGUGUUCCCGAGAUCAAACGUAUUGUAAAAGCGUUUGCAGAUUGUGGUACCUCAAAGAUCCGCAUUACAGGCGGAGAGCCGAGC
Vpa-1-1	CGUAUGUAAUUUCAAAUGUACCUAUUGCCUUCCUGAUGGUUACAAACCAUCGGGUAACAAAAACUCGUCUUUUUUAUCUCUUCCUGAAAUCAAACGGGUCGUAAAAGCGUUUGCUGAUUGUGGUACGUCAAAAGUGCGCAUUACUGGCGGCGAGCCAAGC
Val-1-1	CGUAUGUAAUUUCAAAUGUACCUAUUGCCUUCCUGAUGGCUAUAAGCCUUCGGGCAAUAAAAACUCGUCUUUUUUAUCUCUCCCUGAAAUCAAACGGGUCGUAAAAGCAUUCGCUGAUUGUGGCACGUCAAAAGUGCGCAUCACUGGUGGUGAGCCUAGC
Vsp-1-1	CGUAUGUAAUUUCAAAUGUACCUAUUGCCUUCCUGAUGGCUAUAAGCCUUCGGGCAAUAAAAACUCGUCUUUUUUAUCUCUUCCUGAAAUCAAACGGGUCGUAAAAGCGUUUGCUGAUUGUGGCACGUCAAAAGUGCGCAUCACUGGCGGUGAACCAAGU
Psp-1-1	UGUCUGUAACUUCAAAUGUACCUAUUGCUUGCCAGAUGGCUAUCAUCCAACAGAAAAGAAAAAACCUUCCUUUCUAACAUUGGGUGAGAUUCAGCGAGUUGCCUUAGCAUUUGCUGAGUGUGGCACUAGUAAAAUCCGUAUCACAGGUGGUGAGCCGAGU
Van-1-1	UGUCUGUAACUUCAAAUGUACCUAUUGCUUGCCAGAUGGCUAUCAUCCAACAGAAAAGAAAAAACCUUCCUUUUUAACAUUGGAUGAGAUUCAGCGAGUUGCCUCAGCGUUUGCUGAGUGUGGCACCAGUAAAAUACGUAUCACAGGUGGUGAGCCGAGC
Ppr-2-1	GCGACUAUCAGUCACAGAUGUCUGUAAUUUUAAAUGUACUUACUGCUUGCCUGAUGGCUAUAAGCCGACAGAAAAAAAGAAACCUUCUUUUCUUACAUUGAAUGAGAUUGAGCGCGUAACAAGGGCUUUUGCUGAAUGUGGAACGUCAAAAGUACGUAUC
Ppr-1-1	ACGACUAUCAGUCACAGAUGUCUGUAAUUUUAAAUGUACUUACUGCCUGCCUGAUGGCUAUAAGCCGACAGAAAAAAAGAAACCUUCUUUUCUUACAUUGGAUGAAAUUGAGCGCGUAACAAGGGCUUUUGCUGAAUGUGGAACGUCAAAAGUACGUAUC
	....
	....
	●●●

```
Dha-2-6   AGAAAGGCAGGCCGCCUUUUCUGUUGGUAAUGAGUUCUAUGAUGUUAUUCAUAUUGUGUCAUUUAUAUUGUUUUUAUAAGGAGAAAACCAUGGCAAACAAUCAGCAUGAUCACAUCAGCUGCCCCUCCUCAUCCUGCAUCGGUUGUGCCACACGCUGUCA
Dha-1-6   AGAAAGGCAGGCCGCUUUUUCUGUUGGUAUUGUUUUUAUAAGGAGAAACACCAUGGCAAAUAAUCAGCAUGAUCACAUCAGCUGCCCCUCCUCAUCCUGCAUCGGUUGUGCCACACGCUGUCAUCGGCAGCAGAAUGAUCCGUUCUCCCGGGAUGAGGCU
Dha-1-7   AUUGUGAGCGAAAGUGCGCUUUUGCUAUUGUUUUUGCUUUUGGUAACCAAGGGUUCCGUAGAGGCCUUAGAGGAGAAGAAGGGGGAAAAAGGAUGGCAGACUGUCUUUCCGCAAUGCGUUCGGUGGAAUUUCUGAGUCAUUUUUCCGAUGAUGAGCUCCA
Dha-1-8   UUGUUUUUGCUUUUGGUAACCAAGGGUUCCGUAGAGGCCUUAGAGGAGAAGAAGGGGGAAAAAGGAUGGCAGACUGUCUUUCCGCAAUGCGUUCGGUGGAAUUUCUGAGUCAUUUUUCCGAUGAUGAGCUCCAGGAGAUUGGCAAAUGCCUGCUGACCAG
Dha-2-7   UUGUUUUUGCUUUUGCUGGCCAAGGGUUCCGUAGAGGCCUUAGACGAGAGAAGGGGGAAAAAGGAUGGCAGACUGUCUUUCCGCAAUGCGUUCGGUGGAAUUUCUGAGUCACUUUUCCGAUGAUGAGCUCCAGGAGAUUGGCAAAUGCCUGCUGACCAGG
          ................................................................................................................................................................
          ................................................................................................................................................................
          ●●●●●●●●●●●●●●●●●●●●●●●●●●●●●●●●●●●●●●●●●●●●●●●●●●●●●●●●●●●●●●●●●●●●●●●●●●●●●●●●●●●●●●●●●●●●●●●●●●●●●●●●●●●●●●●●●●●●●●●●●●●●●●●●●●●●●●●●●●●●●●●●●●●●●●●●
```

alignment positions 641 · · · 724

```
Aba-1-1   CGGUGGCGAGCCGCUUCUCCGCCGGGACCUGCUUGAACUCGUCCGCGAACUUCGUGCCUUGAAAACGCUCGAUGGUGAGCCCC.
Tth-1-1   CAAGGCCUACCCCGAGCUCUUGGAGCAGAGAGGCCUCGCCGAGCCCGGCACGCGCAAGCCCUACGCCCUCGGGCGGGUGGUCC.
Tth-2-1   CAAGGCCUACCCCGAGCUCUUGGAGCAGAGAGGCCUCGCCGAGCCCGGCACGCGCAAGCCCUACGCCCUCGGGCGGGUGGUCC.
Sth-1-1   CCGAGGAUCUGCCCCCCUUUCCCCGCAGCUCAGUGGACGGCUAUGCCGUUCUGUCGGCCGACACCUUCGGCGCGUCUGAAGGGC
Csp-1-1   CGACCUGGUCGAGAUCAUCGCCCGCGCCGCCGCCACGCCGGGAAUCGACAAGGUGGCGGUCACCACCAAUGGCUGGAACCUCAA
Rsp-1-1   CCCGAAAUCAUUUACCGAUUGAAUCGCAUAGACGGCAUCAACAACAUCGCUGUGACCACCAAUGGCUACAAGUUGCCACAGCGU
Ama-1-1   UUGUGUGAAAUCAUCGAAACCUGCAAGAAAGUCGAUGGCAUUAAAAAAGUCGCACUGACAACCAACGGUUUCAAACUCAAUCAG
Cps-1-1   GAAAUCAUUCGAAUUUGUAAAGAAACAGCUGGCAUCAAAAAAGUUGCCAUUACAAGUAAUGGUUUUAAGUUACCAGAUCACCU.
Dha-2-1   UUGCGUGUCAUGCGGAUUAGCACCACAUUGGGCUUUAGACGGUUCCGCAUCACCGGCGGGGAACCCUUAAUCAGGGCCGGAGU.
Dha-1-1   UUGCGUGUCAUGCGCAUUAGCACCACAUUGGGCUUUAGACGGUUCCGCAUCACCGGCGGGGAACCCUUAAUCAGGUCCGGAGU.
Dha-2-2   GCUGUGGCCGGUCCGGGGCUUUCUUUCAGUGAAACGAAAGAGGGAAAGGUCAAUCUGAUUGCUGAAGAGGAUGGUUUAAUCUA.
Pat-1-1   UAUAACGGGUGGCGAGCCUUCGCUACGAAAAGAUUUGCCAGACGCUAUUCGUGCCUGCGCAAAUACCCCGGGCAUCAAGCAGGU
Pat-1-2   AUUUGCCAGACGCUAUUCGUGCCUGCGCAAAUACCCCGGGCAUCAAGCAGGUCGCGAUCACGACGAAUGGUUAUAAAUUACCUG
Swo-1-1   AGAUACUUUGAGAUAGGAGGAAGAUAUAUGAAAAAGAAGGAGCUUGUAAUUCUGGCGAUGCUUGCCCUGCUGAUCAUCAGUGGC
Mth-1-1   AGAAGCAGCGGACGGCCACGGCGGCAAGCUGCCCUGCGCCGUAGCCCAGGAGGUUGCCCGCCGCCUUAAGGUACCAAUGCGGGA
Mth-1-2   AAUAGCGAGUACCCUGAUUGCCGCCCUGGCCCUGGCCGGCUGCCGCCAGGUUGCAACACCGCAGCCCCGGCAACAGGGGCAACA
Dha-1-3   GCUGAAUUACAACGGCACAGGCUCCGGUACGGGCAAUAAGUCCUUGGAUUUUAACGGGUAUGAAUAUACCAAAACCGGCGGUAC
Mth-1-3   CCCCUGGAGGGCCAUCCGCGCAUCAAGCUGGCCGGGGAAAUAAAGGGCAUGCCCGUGCGGGAUCUGGCGGAGUACAUUAAGUCC
Dha-1-4   GAAAAGAUGUAGCUGCAAUCUAUAAUCAGAACAAAUUAAUGUUUUUUGAAAGCACUAAAGAAUUUUCAGGAUAUAAUCAUGUUU
Dha-2-3   AGAAAAGAUGUAGCAGCAAUCUAUAAUCAGAACAAAUUAAUGUUUUUUGAAAGCGCUAAAGAAUUUUCAGGAUAUAAUCAUGUC
Dha-2-4   CAGGUCAAAAGGGGAAUGGAAUAUGGUCUGUAUGCAGUUGCUUAGGGAUAGAAAAAUUGAUUUUGCCGUUCUGGGUAAAGAUCA
Dha-2-5   GGAAUGGAAUAUGGUCUGUAUGCAGUUGCUUAGGGAUAGAAAAAUUGAUUUUGCCGUUCUGGGUAAAGAUCAGUGGUGUUGUG.
Mth-1-4   GGCCGGCCGGAACGUCAGGACUUCCCUCUCUUGAAAGGUAAAGAAGUGAUGCUCCAGGCCGCCUUUGCUGGCGCGGUGGGGCAG
Cpe-2-1   AAGUGAGGUUGUUAGCAUAAAGGAUUCUUUAAAGAGAGUUUUGUAUGGUGAUGUUAAAUCUAAGAUUAAUAAUCCUCCCUUUAA
Cpe-1-1   AAGUGAGGUUGUUAGCAUAAAGGAUUCUUUAAAGAGAGUUUUGUAUGGUGAUGUUAAAUCUAAGAUUAAUAAUCCUCCCUUUAA
Cpe-3-1   AAGUGAGGUUGUUAGCAUAAAGGAUUCUUUAAAGAGAGUUUUGUAUGGUGAUGUUAAAUCUAAGAUUAAUAAUCCUCCCUUUAA
Pin-1-1   GCAAAGAUUUUCCGAAAAUAAUUGAAGCCAUUGCCACUAUCCCUGGCAUCCAAAAAGUAGCCACUACGACUAACGGUUACAAUU
Apl-1-1   GUGGUUAGAUCCGGUCCUGCUAUGCCUCCGUCAAUCGAAAACGAAUUACAAACUGUCGUGCCUGAUCAAGUACACGGUGAAACU
Hdu-1-1   GUGGUUAAAUCCGGUCCGGCAAUGCCAGUUUCUGUUGAAAAUGAAUUACAAACAGUUGUGCCAGAUCAAGUGCAUGGCGAAACA
Mha-1-1   GAAAAUGGCAAAGUGGUGAAAUCUGAUUCUGUUUUUGAGAUUUCUGUGCCAAAUGAAUUGCAACACGUGGUAGCAGACCAAGUA
Apl-1-2   GAAAAAGUGCGCAUUACCGGUGGCGAACCGACUUUACGUAAAGAUUUUUUAGAAAUUGCGCAUACCGUUUCGCAAACGAACGGC
Hso-2-1   UGUUGCGGCUGAACAACAUACCGUUGUAACAGCUGCUCAUUGGGGGGCUUUAGGUGUAGUUGUAGAAAAUGGCAAGGUAGUUAA
Hso-1-1   UGUGGCGGCUGAACAACAUACCGUUGUAACAGCUGCUCAUUGGGGGGCUUUAGGUGUAGUUGUAGAAAAUGGCAAGGUAGUUAA
Asu-1-1   AUUACCGGCGGCGAGCCUACUUUACGCAAAGAUUUCCUGCAAAUUACGGAAAAUAUCACCGCACUUGACGGUAUUCGACAUGUC
Msu-1-1   CGUUUAACCGGCGGCGAACCGACGUUACGCAAAGAUUUUUUAGCUAUUGUUGAAACCAUCAGCGCCCUAGAAGGCAUUAAAAAA
Hso-1-2   UUCGUAUUACCGGUGGUGAGCCGACUCUUCGUAAAGAUUUCCUGUCCAUUGUUGAAAAUAUUCGUGCUAUUGAAACAAUAAAA.
Hso-2-2   UUCGUAUUACCGGUGGUGAGCCAACUCUUCGUAAAGAUUUCCUGUCCAUUGUUGAAAAUAUUCGUGCUAUUGAAACAAUAAAA.
Hin-3-1   CGUUUAACGGGUGGCGAACCGACUUUACGCAAAGAUUUUAUUUCUAUUGCUGAAAGCAUUGCUAAUAUUGAUGGCAUUCGUCAA
Hin-4-1   CGUUUAACGGGUGGCGAACCGACUUUACGCAAAGAUUUUAUUUCUAUUGCUGAAAGCAUUACUAACAUUGAUGGCAUUCGUCAA
Hin-1-1   CGUUUAACGGGUGGCGAACCGACUUUACGCAAAGAUUUUAUUUCUAUUGCCGAAAGCAUUGCUAACAUUGAUGGCAUUCGUCAA
Hin-2-1   CGUUUAACAGGUGGCGAACCAACUUUACGUAAAGAUUUUAUUUCCAUUGCUGAAAGCAUUGCUAACAUUGAGGGCAUUCGUCAA
Pmu-1-1   CGUAUUACGGGGGGAGAACCUACCUUACGUAAAGAUUUUUUACCUAUUGUUGAAACCAUUGCUCAAAAUCCUACUAUUAAACAG
Mha-1-2   ACGGAAAAAAUCCGUAUCACCGGUGGUGAGCCAACUUUACGCAAAGAUUUCCUCGAAAUCGCUCACACUAUUUCACAAACACCC
Plu-1-1   CGAUUUCACCGAUAUUAUUUCCGUCAUACGUGAAAAUAAGUUGAUUAAAACCCUCGCGGUAACCACUAACGGUUAUCGAAUGGA
Eca-1-1   GCGUCGUGACUUCGUCGAUAUCAUUGCUGCGAUCCGUGAAAACCCAGCUAUUCGUACGCUGGCGGUCACCACCAACGGUUACC.
Spr-1-1   UCGCGAUUUUACCGAGAUCAUUGCCGCCGUGCGUGAAAAUCCGGCCGUUCGCCAGCUUGCCGUGACCACCAAUGGCUACCGCCU
Sgl-1-1   CGCCGUGAUUUCAUCAACAUCAUUGCGGCGGUGCGCGCCAAUCUGGCUAUCCGCACCGUCGCGGUCACCACCAACGGAUAUCG.
Ymo-1-1   UUUCACCGACAUCAUCGCCACCAUUCGGCAAAAUCCCGCGAUCCGCACAUUGGCGGUGACCACCAAUGGUUACCGUUUAGCGCG

          ....................................................................................
          ....................................................................................
          ●●●●●●●●●●●●●●●●●●●●●●●●●●●●●●●●●●●●●●●●●●●●●●●●●●●●●●●●●●●●●●●●●●●●●●●●●●●●●●●●●○
```

```
Yps-2-1    CUUCACUGAUAUUAUUGCCACUAUCCGGCAAAAUCCCGCUAUCCGUACCUUGGCCGUUACAACUAAUGGUUAUCGUUUAGUUCG
Yps-1-1    CUUCACUGAUAUUAUUGCCACUAUCCGGCAAAAUCCCGCUAUCCGUACCUUGGCCGUUACAACUAAUGGUUAUCGUUUAGUUCG
Yfr-1-1    UUUUACCGAUAUUAUUGCCACAAUCCGGCAAAAUCCUGCGAUCCGUACCUUGGCAGUGACUACCAAUGGCUACCGUUUAGCGCG
Yin-1-1    CUUCAGCGAUAUCAUUGCCACCAUCAGACAAAAUCCAGCGAUCCGCACUCUGGCAGUCACCACCAAUGGCUAUCGUUUAGCCCG
Ybe-1-1    UUUUACCGAUAUUAUCGCCACUAUCCGGCAAAAUCCCGCGAUCCGCACAUUAGCGGUGACCACCAAUGGUUAUCGUUUAGCGCG
Spe-1-1    AUUUCACCGACAUCAUUCGCAUCGUUAAAGACAACGAUAAGAUUAAAACCAUUGCCACGACUACUAACGGUUACCGUUUAGCUA
Swo-2-1    ACUUUACCGAUAUUAUUCGUGCGGUUGCUGAUAACGAUAAGAUUAAAACCAUCGCGACAACGACAAACGGUUACCGUUUAGAGA
Ssp-4-1    AUUUUACCGACAUCGUGCGUAUCGUCGCGGACAACGACAAGAUCAAGACGCUGGCGACCACCACCAAUGGCUAUCGCCUCGAGA
Ssp-5-1    ....................................................................................
Ssp-5-2    AUUUUACCGACAUUAUUCGUGUUGUGGCCGAUAACCCGCGCAUUCACACUGUUGCGACUACCACUAAUGGGUAUAGACUCGAAA
Spu-1-1    AUUUUACCGACAUUAUUCGUGUUGUGGCCGAUAACCCGCGUAUUCACACUGUUGCGACUACCACUAAUGGGUAUAGGCUCGAAA
Sba-1-1    ACUUUACCGACAUCAUCCGUGUCGUCGCCGAUAACCCGCGCAUUCACACCAUAGCGACAACCACUAAUGGCUAUAGACUCGAAA
Sba-2-1    ACUUUACCGACAUCAUCCGUGUCGUCGCCGAUAACCCGCGCAUUCACACCAUAGCGACAACCACUAAUGGCUAUAGACUCGAAA
Ssp-1-1    AUUUUACCGAUAUCAUUCGAGUGGUCGCCGACAAUCCUCGUAUUCAUACCGUAGCAACUACCACUAAUGGGUAUAGGCUCGAGA
env-2      AUUUUACCGAUAUCAUUCGAGUGGUCGCCGACAAUCCUCGUAUUCAUACCGUAGCCACUACCACUAAUGGGUAUAGGCUCGAGA
Ssp-2-1    AUUUUACCGAUAUCAUUCGAGUGGUCGCCGACAAUCCUCGAAUUCAUACCGUAGCCACUACUACUAAUGGGUAUAGGCUCGAGA
Ssp-3-1    AUUUUACCGAUAUCAUUCGAGUGGUCGCCGACAAUCCUCGAAUUCAUACCGUAGCCACUACUACUAAUGGGUAUAGGCUCGAGA
Son-1-1    AUUUUACCGAUAUUAUUCGAGUCGUUGCCGAUAAUCCGCGUAUUCAUACUGUUGCUACUACCACUAAUGGAUAUAGGCUCGAGA
env-3      AUUUUACCGAUAUCAUUCGAGUGGUCGCCGAUAAUCCGCGUAUUCAUACCGUAGCCACUACCACUAAUGGGUAUAGGCUCGAGA
Sfr-1-1    AUUUCACAGAUAUUGUGCGGAUCGUUGCCGACAAUCCUAAUAUUAAUACCGUAGCGAUGACCACCAAUGGGUAUCGUUUACAGC
Sde-1-1    ACUUUAUCGACAUUAUCGGCCUUGCUGCUAAUACCCCCAAUAUCAAGACUGUCGCCACCACCACCAAUGGUUACCGCUUAGCGA
Sam-1-1    AUUUUACCGAUAUCAUCCGGGUCAUCGCAGACAACCAGGCCAUCAGUACCCUGGCCACCACCACCAACGGUUAUCGCCUGGAGC
Esp-1-1    UUCGUCGUGACUUUCCUGACAUUAUUGCGGCUAUUCGUGAAAACGAGAGCAUUCGUCAAAUCGCCGUCACCACCAAUGGCUAUC
env-4      UGCGUCGCGACUUUACUGACAUUAUCGCCGCCGUGCGCGAAAACGACGCCAUCCGUCAGAUUGCGGUAACCACCAAUGGUUAUC
Eco-15-1   UACGCCGCGACUUUACCGAUAUCAUCGCCGCUGUGCGAGAAAACGACGCUAUUCGCCAGAUAGCGGUCACCACCAAUGGUUACC
Eco-14-1   UACGCCGCGACUUUACCGAUAUCAUCGCCGCUGUGCGAGAAAACGACGCUAUUCGCCAGAUAGCGGUCACCACCAAUGGUUACC
Eco-3-1    UACGCCGCGACUUUACCGAUAUCAUCGCCGCUGUGCGGGAAAACGACGCUAUCCGCCAGAUUGCGGUCACAACCAAUGGUUACC
Eco-1-1    UACGCCGCGACUUUACCGAUAUCAUCGCCGCUGUGCGAGAAAAUGACGCUAUCCGCCAGAUUGCGGUCACCACCAAUGGUUACC
Eco-17-1   UACGCCGCGACUUUACCGAUAUCAUCGCCGCUGUGCGGGAAAACGACGCUAUCCGCCAGAUUGCGGUCACAACCAAUGGUUACC
Eco-6-1    UACGCCGCGACUUUACCGAUAUCAUCGCCGCUGUGCGGGAAAACGACGCUAUCCGCCAGAUUGCGGUCACCACCAAUGGUUACC
Eco-2-1    UACGCCGCGACUUUACCGAUAUCAUCGCCGCUGUGCGAGAAAACGACGCUAUUCGCCAGAUAGCGGUCACCACCAAUGGUUACC
Sfl-2-1    UACGCCGCGACUUUACCGAUAUCAUCGCCGCUGUGCGGGAAAACGACGCUAUCCGCCAGAUUGCGGUCACCACCAAUGGUUACC
Sbo-2-1    UACGCCGCGACUUUACCGAUAUCAUCGCCGCUGUGCGGGAAAACGACGCUAUCCGCCAGAUUGCGGUCACCACCAAUGGUUACC
Sso-1-1    UACGCCGCGACUUUACCGAUAUCAUCGCCGCUGUGCGGGAAAACGACGCUAUCCGCCAGAUUGCGGUCACCACCAAUGGUUACC
Sfl-3-1    UACGCCGCGACUUUACCGAUAUCAUCGCCGCUGUGCGGGAAAACGACGCUAUCCGCCAGAUUGCGGUCACCACCAAUGGUUACC
Sdy-1-1    UACGCCGCGACUUUACCGAUAUCAUCGCCGCUGUGCGAGAAAACGACGCUAUCCGCCAGAUUGCGGUCACCACCAAUGGUUACC
Sbo-1-1    UACGCCGCGACUUUACCGAUAUCAUCGCCGCUGUGCGGGAAAACGACGCUAUCCGCCAGAUUGCGGUCACCACCAAUGGUUACC
Sty-1-1    UACGCCGCGAUUUUACCGACAUCAUUGCCGCCGUGGGUGAAAAUGAUGCUAUUCGCCAGAUCGCGGUGACCACUAACGGUUAUC
Sen-1-1    UACGCCGCGAUUUUACCGACAUCAUUGCCGCCGUGGGUGAAAAUGAUGCUAUUCGCCAGAUCGCGGUGACCACUAACGGUUAUC
Sen-4-1    UACGCCGCGAUUUUACCGACAUCAUUGCCGCCGUGGGUGAAAAUGAUGCUAUUCGCCAGAUCGCGGUGACCACUAACGGUUAUC
Sen-2-1    UACGCCGCGAUUUUACCGACAUCAUUGCCGCCGUGGGUGAAAAUGAUGCUAUUCGCCAGAUCGCGGUGACCACUAACGGUUAUC
Ahy-1-1    CCUCCCUGCGCCGGGACUUCACCGCCAUCAUAGAGACGGUGGCCAAUACCCCCGGCAUCGAGAAGGUGGCCAUGACCACCAAUG
env-5      CCUCCCUGCGGCGGGACUUCACCGCCAUCAUCGAGACGGUGGCCAAUACCCCCGGCAUCGAGAAGGUGGCCAUGACCACCAAUG
Vch-1-1    CUGCGCAAAGAUUUUGGCGAAAUCAUUCAUACCAUAGCGCAAACUCCCGGCAUUCAAAAAGUUGCGACGACCACCAAUGGCUAC
Vch-8-1    CUGCGCAAAGAUUUUGGCGAAAUCAUUCAUACCAUAGCGCAAACUCCCGGCAUUCAAAAAGUCGCGACGACCACCAAUGGCUAC
Vch-10-1   CUGCGCAAAGAUUUUGGCGAAAUCAUUCAUACCAUAGCGCAAACUCCCGGCAUUCAAAAAGUUGCGACGACCACCAAUGGCUAC
Vch-13-1   CUGCGCAAAGAUUUUGGCGAAAUCAUUCAUACCAUAGCGCAAACUCCCGGCAUUCAAAAAGUUGCGACGACCACCAAUGGCUAC
Vch-7-1    CUGCGCAAAGAUUUUGGCGAGAUUAUUCAUGCCAUAGCGCAAACUCCCGGCAUUCAAAAAGUUGCGACGACCACCAAUGGCUAC
Vch-12-1   CUGCGCAAAGAUUUUGGCGAAAUCAUUCAUACCAUAGCGCAAACUCCCGGCAUUCAAAAAGUUGCGACGACCACCAAUGGCUAC
Vch-3-1    CUGCGCAAAGAUUUUGGCGAAAUCAUUCAUACCAUUGCGCAAACUCCCGGCAUUCAGAAAGUCGCUACCACCACCAACGGCUAU
Vfi-1-1    CUUCGCAAAGAUUUCACUGAUAUUAUUCGUACUGUCGCCUCUCAGCCUGGUAUUACUAAGGUUGCAACUACUACCAACGGUUAU
Vvu-1-1    CUGCGUAAAGAUUUUACUGACAUUAUCCACACGGUCGCAUCAACUCAAGGCAUUAAGCGAGUUGCCACUACCACCAAUGGCUAU
Vvu-2-1    CUGCGUAAAGAUUUUACUGACAUUAUCCACACGGUCGCGUCAACUCAAGGCAUUAAACGAGUUGCCACUACCACCAAUGGCUAU
Vsp-2-1    CUGCGUAAAGACUUUCCUGAAAUCAUACAUACCGUUGCUUCUACUCCAGGCAUUGAAAAAGUAGCCACCACAACGAAUGGCUAC
Vsp-3-1    CUGCGUAAAGACUUUCCUGAAAUCAUACAUACUGUUGCUUCUACUCCAGGCAUUGAAAAAGUAGCCACCACAACGAAUGGCUAC
Vpa-1-1    UUGCGCAAAGAUUUCACUGACAUCAUUCAUUCUGUCGCAACGACGCCAGGCAUCAAAAAGGUGGCGACGACGACCAACGGCUAC
Val-1-1    UUACGCAAAGAUUUUACCGACAUUAUCCAUUCAGUAGCGGCGACGCCUGGCAUUCAAAAAGUUGCAACGACAACCAAUGGCUAU
Vsp-1-1    UUACGCAAAGAUUUUACCGACAUUAUCCACUCAGUGGCGACGACGCCUGGUAUCCAAAAAGUUGCAACUACGACCAAUGGUUAC
Psp-1-1    UUACGUCGUGAUUUUACUGAUAUCAUCAAAACGGUGGCUGAGAUCCCUGGGAUUAAUAAAGUAGCGACAACCACUAAUGGUUAU
Van-1-1    UUACGCCGUGAUUUUACUGAUAUUAUCAAAACGGUAGCUGAAAUCCCUGGGAUCAAUAAAGUAGCGACAACCACUAAUGGUUAC
Ppr-2-1    ACGGGUGGCGAACCUAGUUUACGUCGUGAUUAUACGGAUAUUAUCCGUAUAGUCGCGGCUCAGCCCGGCAUAACUAAAGUGGCU

           ....................................................................................
           ....................................................................................
```

```
Ppr-1-1   ACUGGGGGCGAACCUAGUUUACGUCGUGACUAUACGGAUAUUAUCCGUACUGUAGCAGCUCAGCCCGGUAUAACUAAAGUGGCU
Dha-2-6   UCGGCAGCAGAAUGAUCCGUUCUCCCGGGAUGAGGCUUUGGCGAUCCUUUUCGACCAGGCCAAAUUUGAACUGGCCAAGGAAUU
Dha-1-6   UUGGCGAUCCUUUUCGACCAGGCCAAAUUUGAACUGGCCAAGGAAUUCGUACCACUGAAUGAAGCUUUGGGCCGGGUGACUUCG
Dha-1-7   GGAGAUUGGCAAAUGCCUGCUGACCAGGGUGCAGAGGUACAAAAAGGGUGAAAAAGUUUUUGAGGCGGGUCAACCCGCUGUUU.
Dha-1-8   GGUGCAGAGGUACAAAAAGGGUGAAAAAGUUUUUGAGGCGGGUCAACCCGCUGUUUCCAUGGGCGUUAUUCUGCAGGGAAGGGU
Dha-2-7   GUCCAGAGGUACAAAAAGGGUGAAAAGGUUUUUGAGGCGGGUCAGCCCGCUGUUUCCAUGGGUGUUAUUCUGCAGGGAAGGGUG
          ....................................................................................
          ....................................................................................
```

Supplementary Figure S6: alignment of sequences lacking P3

The format of this figure is identical to Supplementary Figure S4.

alignment positions 1 · · · 160

```
Pca-1-1     ACCUAUGUGUUCCUC.CAUGUGC.UG.CG.UUUUGGCGGA......................UAC...............................................................................................
Mth-1-1     UCCAUAAGCGACUUC.CGAGUUG.UCCUC.UCC....C........................ACC...............................................................................................
Hdu-1-1     AUUAUUUUAGACUC.CGAGCCU.AC.UU.UCCUAAG.........................UCU...............................................................................................
Dre-1-1     UGAUAUUUACACUUC.CGAUUUA.CU.AU.AU....UCCU......................ACU...............................................................................................
Ame-1-1     AAUGUUACGAAUUUC.CGAGUGA.GA.UU.AUCUAAGGC.......................GAU...............................................................................................
Ame-1-2     AAUAAAAUUUAUUUC.CGAGUUA.CU.UU.UCCUAAGGGCA.....................AAC...............................................................................................
Csp-1-1     AUAACUAAAUAUCUC.CGAGUUC.AU.CGAUCCUAAUAC.......................CUGUUUC...........................................................................................
Dps-1-1     UUAAGAAUAUAACUC.CGAGCUA.AA.AG.GCCUUCGGUUU.....................CUGCAU............................................................................................
Sfu-1-1     AGAACGGUCGCUCCC.CGAGCCG.CC.G........GGUC......................UAA...............................................................................................
env-1       UAAUUAUGACUUCUC.CGAGCCU.UU.UU.GCCUAAAG........................GAAA..............................................................................................
Swo-1-1     AUAAUUAUGGCUCGC.CGAGUUU.UU.CC.GUCUAAGGUUUU....................UUA...............................................................................................
Dre-1-2     GUAAUAACUUGUCUC.CGAGCCG.CU.UG.GUCUAAGGC.......................ACUU..............................................................................................
Hne-1-1     GUUGGAAAUUCGCUC.CGAGCCG.UU.CC.GCCUAAAGCGCCG...................UUUCC.............................................................................................
Swo-1-2     UCAAGAGCUUAACUC.CGAGCUA.UC.CC.GCCUAAAGCG......................AAA...............................................................................................
Swo-1-3     AAGCGUAAUUAACUC.CGACUCA.CA.CCAGCCUAAGGU.......................AAUG..............................................................................................
Swo-1-4     AAGCGUAAUUAACUC.CGACUCA.CA.CCAGCCUAAGGU.......................AAUG..............................................................................................
env-2       AACUGAUAAUUUCUC.CGAUUCA.UUCAU.GCCUAAUGCG......................AC................................................................................................
env-3       AACUGAUAAUUUCUC.CGAUUCA.UUCAU.GCCUAAUGCG......................AC................................................................................................
Chy-1-1     AAAAAUAAGCUGCUC.CGAGUAU.UU.UC.ACCUAAAGCU......................UUA...............................................................................................
Tet-1-1     AGGAGAAUAACUCUC.CGAGCCU.UU.AU.GUCUAAGGGGC.....................GAAGA.............................................................................................
env-4       AAAUUUGUAAAUCUC.CGAUUUG.CC.GUGACCUAAAGUG......................UC................................................................................................
env-5       AAUCUACUUAAUCUC.CGAUUCG.CU.UACACCUAAAGU.......................AUAA..............................................................................................
Swo-1-5     GAAUAGAACCGGCUC.CAAGCCU.UU.GA.GCCUAAUGACUGAA.ACUCA............UUUGUCAGGGAGAAUCGGACUUUUAUGGUGGAAAAAGCCUGAAUCCCUGAGCCCGUUAAAAAAGUAUAAAAAUUAUGGACGAUUAAUGCAUUUUAUAC
Swo-1-6     ACUUAAUAUGUAUUC.CGAGCCC.UC.UUAACCUGCAGA.......................CAAA..............................................................................................
Swo-1-7     AAAAGAAUAGAGCUC.CGAGCCU.UAUUA.AUCUAAAGAC......................UGCG..............................................................................................
Dre-1-3     AAAUUAAUAGUACUC.CGAGCUU.AG.GC.ACCUAAGAU.......................AGUA..............................................................................................
Tet-1-2     UUAUUACUUAUUCUC.CGAGUCU.AU.AA.UCCUAAGGC.......................AAGA..............................................................................................
Swo-1-8     AAACUAAAUAUACUC.CGAGCCU.GA.AA.ACCU..GGUA......................AUAA..............................................................................................
Swo-1-9     UUGAAUAUUUGUUUC.CAAGCCU.UU.GA.ACCUAAGGUU......................UAU...............................................................................................
Swo-1-10    ACAAUUGUAUAGCUC.CAAGCCU.UU.AG.ACCUAAAGC.......................AAA...............................................................................................
Swo-1-11    GAAUAUCAAGUUCUC.CGAGCUU.UU.AG.AUCUAAGGU.......................UU................................................................................................
Swo-1-12    AAGAAUAUAUAUCUC.CGAGCUG.GG.GA.GUCUAAGGC.......................GAA...............................................................................................
Swo-1-13    UUUACAUACUUUUUC.CGAACUU.GA.GG.ACCUAAAGC.......................AAAU..............................................................................................
Csa-1-1     UUUUGAAAAUCUUUC.CGAGCCA.UU.GCAACCUAAAAAGAGGGG.................UUUU..............................................................................................
Dac-1-1     CAGCCCUUAAAUCUC.CGAGCCGCUA.CC.ACCUACGAGC......................GU................................................................................................
Dac-1-2     AAAUAGUUACAACUC.CGAGUCU.GU.UC.ACCUACCGG.......................CACAU.............................................................................................
Swo-1-14    UAACUGAAUAAUCUC.CGAGCUG.UA.CU.GCCUU.CAGUC.....................AC................................................................................................
Dac-1-3     UACUAUCAAGAUCUC.CGAGCCU.GC.CCCCCCUACGGAGG.....................UUG...............................................................................................
Gsp-1-1     GACAAAACAAGUCUC.CGAGCCU.CC.AUUGCCUAAAGGG......................CGUCAAA...........................................................................................
Gsp-1-2     UGAUUCUCAACUCUC.CGAGCCA.CG.CC.GCCUAAAGGG......................UAGUUU............................................................................................
Gsp-1-3     UAAAAACUAAAUCUC.CGAGUCG.UAAUU.GCCUACCGGGGAUGACA...............AAGC..............................................................................................
Pca-1-2     AUGCGCGGCAGUCUC.CGAGCCA.GG.UCCGCCUAAGGGGA.....................UGG...............................................................................................
Ppr-1-1     GGAGCAAUCUGUCUC.CGAGCCU.GC.CC.UCCUACCGGG......................ACAC..............................................................................................
Gsp-1-4     UGCUCGAACUUUCUC.CGAGCCU.GU.CC.GCCUACCGGGAAU.....................................................................................................................
Gsu-1-1     AUAUUUUGUUAUCUC.CGAGCCC.AU.CC.GCCUACCAGGA.....................ACA...............................................................................................
Gme-1-1     UACUAGUUUUUUCUC.CGAUCCG.UC.AU.ACCUACCAGGC.....................GCAGA.............................................................................................
Gur-1-1     AAAUUGUCGGCUCUC.CGAGCCG.CC.GU.ACCCACCAGGG.....................CAACC.............................................................................................
Gur-1-2     GAAACUGACGCUCUC.CGAGCUG.UA.GC.GCCUAAAAGGU.....................UUGU..............................................................................................
Pca-1-3     AAUACUACCAAUCUC.CGAGCCU.CG.CU.GCCUACUGGGA.....................AGG...............................................................................................
Gme-1-2     ACAACCAACACUCUC.CGAGCCU.GC.AA.GCCUAACAGG......................GAAA..............................................................................................
Ppr-1-2     AUGCCAUGUUAUCUC.CGAGCCU.UG.CC.ACCUAAAAGG......................AAAA..............................................................................................
Pca-1-4     UACAGAACACGUCUC.CGAGUCU.U..AA.GCCUAAAAGG......................AUGGA.............................................................................................

            ..<<<<<<<<<<<<<.<..<<<<.<<.<<.<<<...<<<<<<<<<<<<<<<<<<<<<<<<<<..................................................................................................
            ..??????????220.0..2222.??.??.?21...?2222?22?22000????????????..................................................................................................
            •••••••••••YCUC-CGAGYY•-••-••-RCCUA••R•○----------------------••○○----------------------------------------------------------------------------------------------
                                          -TR-
```

```
Gur-1-3    CAAACACAUCAUCUC.CGAGCCU.UU.CA.GCCUAAAAGG......................AUAAA.............................................................................................
Gsp-1-5    UUCAUGUAUCAUCUC.CGAGCCG.UG.CA.GCCUAACAGG......................UUC...............................................................................................
Pca-1-5    UCAAGAAUGCGCCUC.CGAGCCU.GA.UG.CCCUAAAAGAGAU...................UUU...............................................................................................
Gur-1-4    ACACCUGUUUUUCUC.CGAGCCU.CC.UC.ACCUACAGGG......................CAUCC.............................................................................................
Gme-1-3    AUUUAAAACCGUCUC.CGAGCCG.CG.GC.GCCUAAAGGGA.....................ACA...............................................................................................
Gme-1-4    AUUUAAAACCGUCUC.CGAGCUG.CG.GC.GCCUAAAGGGA.....................ACA...............................................................................................
           ..<<<<<<<<<<<<<.<..<<<<.<<.<<.<<<...<<<<<<<<<<<<<<<<<<<<<<<<<<..................................................................................................
           ..??????????220.0..2222.??.??.?21...?2222?22?22000????????????..................................................................................................
           ●●●●●●●●●●●YCUC-CGAGYY●-●●-●●-RCCUA●●R●○----------------------●●○○----------------------------------------------------------------------------------------------
                                         -TR-
```

alignment positions 161 · · · 320

```
Pca-1-1    ........................................UCCUGUGAGCG.UGGC.ACC...................................................................................................G
Mth-1-1    ..........................................G...GGAAG.GGCA.AAC...................................................................................................G
Hdu-1-1    ...........................................UAUUAAAU.UUGA.GCAAAU...........................................................................................CAU
Dre-1-1    ........................................GGGA...AU.U.AGUA.AAC....................................................................................................
Ame-1-1    .........................................GCUACGAU.G.ACUU.ACC...................................................................................................U
Ame-1-2    .......................................UGUCUAAGGU.U.AGUG.GCC...................................................................................................G
Csp-1-1    .........................................GUAAUGGUCU.AUGA.GCC..................................................................................................UG
Dps-1-1    .......................................GGAUCAUGGUCU.GC.A.GCC..................................................................................................GU
Sfu-1-1    ........................................GGCU..UUUGC.GGCG.GCC..................................................................................................GG
env-1      ..........................................CCAUGGU.A.AAGG.GCC....................................................................................................
Swo-1-1    ......................................AAAGCUAUGACUG.AAAA.GCC...................................................................................................G
Dre-1-2    .........................................GCUAUGAUCU.CCCG.GCC..................................................................................................GU
Hne-1-1    .....................................CGGUGUCAUGGCGG.CCCG.GCC...................................................................................................G
Swo-1-2    ........................................UGCCAUGGUGG.CUUA.GCC..................................................................................................GU
Swo-1-3    .........................................GCUAUGGC.G.GUUG.AGC..................................................................................................GA
Swo-1-4    .........................................GCUAUGGC.G.GUUG.AGC..................................................................................................GA
env-2      ........................................CGCGAAGGCAG.GAUG.AAC..................................................................................................GA
env-3      ........................................CGCGAAGGCAG.GAUG.AAC..................................................................................................GA
Chy-1-1    ........................................GGCCAUGGUGA.AAAU.ACC...................................................................................................A
Tet-1-1    .......................................GCCCUAUGAGAU.AAAG.GCC..................................................................................................GA
env-4      ........................................UACUAUGGUUC.GGUG.AGC..................................................................................................GU
env-5      .........................................ACCAUGGUUU.GGCG.AAC..................................................................................................GA
Swo-1-5    UUUCAUUUUUGCUGGGAU............UGAGUCUUUAAUCCAUGGUUU.AACG.GCC....................................................................................................
Swo-1-6    .........................................UCCAGGUU.A.CAGG.GCC...................................................................................................U
Swo-1-7    ........................................GUUCAUGAUUA.UAGG.GCC...................................................................................................U
Dre-1-3    .........................................AUUAUGGUUC.UUAA.GCC..................................................................................................GU
Tet-1-2    .........................................GCUAUGGAUU.AUAG.GCC..................................................................................................GA
Swo-1-8    ........................................UACUAAGGGUU.UCAG.GCC..................................................................................................UG
Swo-1-9    ........................................AACUAUGGUUC.CCAG.GCC...................................................................................................G
Swo-1-10   .........................................GCCAUGGUUU.CUAG.GCC...................................................................................................U
Swo-1-11   .........................................ACUAUGGUUU.AAAA.GCC...................................................................................................U
Swo-1-12   .........................................GCUAUGAUUC.ACCAAGCC...................................................................................................U
Swo-1-13   .........................................GCCAUGGU.A.UCAA.GUC...................................................................................................A
Csa-1-1    ...................................UCCCUCUUCAUGGUGC.UUUG.GCC..................................................................................................GA
Dac-1-1    ........................................GAUCAUGGUGG.UCCG.GCC..................................................................................................GC
Dac-1-2    .........................................CCCAAGGU.G.GCAG.GCC...................................................................................................U
Swo-1-14   .......................................GGCUAAUGGU.A.UACA.GUC...................................................................................................G
Dac-1-3    .......................................CCUUUAAGGG.G.ACAG.GCC...................................................................................................U
Gsp-1-1    ........................................CCCCACGGCAU.GGAG.GCC..................................................................................................UG
Gsp-1-2    ........................................UCCCAUGGC.G.ACCG.GCU....................................................................................................
Gsp-1-3    .................................UGUCA.UUCCCAUGGCAA..CCG.ACC...................................................................................................A
Pca-1-2    .......................................UCUUCAGGGC.G.UCUG.GCC...................................................................................................A
Ppr-1-1    ........................................CCCCAUGGC.G.GCAG.GCC...................................................................................................A
Gsp-1-4    .....................................AUUCCCCAUGGU.G.UCAG.GCC...................................................................................................U
Gsu-1-1    .......................................UUCCCACGGC.G.GCCG.GCC...................................................................................................G
Gme-1-1    .......................................GCCUCACGGUA..UGCG.GUC...................................................................................................A
Gur-1-1    .......................................CUCUCACGGU.G.CGCG.GCC...................................................................................................A

           ..................>>>>>>>>>>>>>>>>>>>>>>>>>>..>>>>>.>>>>.>>....<<<<<<<<<<<<<<<<<<<<<<<<<<<<<<<<<<<<<<..................>>>>>>>>>>>>>>>>>>>>>>>>>>>>>>>>>>>>>>...
           ..................????????????00022?22?2222?..12???.??22.22....??????????????????????????????????????..................??????????????????????????????????????...
           ----------------------------------------○●YYAYGGY○●-●●●R-GCC---------------------------------------------------------------------------------------------------●
                                                     -TR
```

```
Gur-1-2   .......................................ACCUGAUGGCGC..ACG.GCC...................................................................................................U
Pca-1-3   .......................................UCCUUAUGGC.G.ACAG.GCC...................................................................................................G
Gme-1-2   ........................................CCUCACGGC.C.CAGG.GCC...................................................................................................A
Ppr-1-2   ........................................CCUCAUGGU.G.CACG.GCC...................................................................................................A
Pca-1-4   ........................................CCUCAUGGCUG.ACGG.GCC..................................................................................................GG
Gur-1-3   ........................................CCUCAUGGC.G.CCCG.GCC...................................................................................................A
Gsp-1-5   ........................................CCUCAUGGU.G.CCCG.GCC...................................................................................................U
Pca-1-5   .....................................AUCUCUCAGGGGCG.UCGG.GCC...................................................................................................G
Gur-1-4   ........................................CCCCAAGGA.A.AGAG.GCC...................................................................................................A
Gme-1-3   .......................................UCCCCAUGGCGG.UCCG.GCC...................................................................................................U
Gme-1-4   .......................................UCCCCAAGGCGG.UCCG.GCC...................................................................................................U
          ..................>>>>>>>>>>>>>>>>>>>>>>>>>..>>>>>.>>>>.>>....<<<<<<<<<<<<<<<<<<<<<<<<<<<<<<<<<<<<<<..................>>>>>>>>>>>>>>>>>>>>>>>>>>>>>>>>>>>>>>...
          ..................???????????00022?22?2222?..12???.??22.22....??????????????????????????????????????..................??????????????????????????????????????...
          ---------------------------------------○●YYAYGGY○●-●●●R-GCC--------------------------------------------------------------------------------------------------●
                                                   -TR
```

alignment positions 321 · · · 480

```
Pca-1-1   AUAGGGGA.CU...UC.GGAAACGAAGUCCCCUUCCGCG....................................UG.UCAUGAUGGG.AA.AA.U.GUCAGAGCUCGGUUCACCUGCGGGAACACCUUUGCAAUGGGAGGGAAAAAUGCGCAACAAAAU
Mth-1-1   UCAGGGCC.GG...GG.AGCAAUCCCUUGACCUGCUAUUG...................................GG.CAAAGGAA.G.GU.AC.U.CUUUGUAGGGGAGUACCUUUUUCUUUUUAACUGUAAUAAUAAACUGGCAGCCCAUAAGUUAGC
Hdu-1-1   AAAGGAU..UU...UG.AGAAAUCAAACCUCCUCUCGUAUU..................................UA.GAAAGGUG.U.CC.UA.U.GCAAGCCAUCCCAAUUAAAACUAUUGGCGAUCCAUCCUUAUGCGAUCUUGUUGAUCGAUUCCA
Dre-1-1   .GGGGUGC.GA...UG.AGCAAUUGUCGUGCCUGCCACUG...................................CG.CAAAGGAA.G.GG.UA.C.UAGUGAUGAACUGGUACCCUUUUUAUAUUGCCUAAAAUAGCAAGAAAGUAUUGAGGGGGUAUU
Ame-1-1   CUGGGUAA.AU...GA.AGAAAUUCUCUUGCCUCCCGAACU..................................UG.AAAAGGAA.A.UU.UG.C.UUUACAUAAGAAUUCUAACUAACGAAUUCAUAUUUGUGUUAAAGCUAAUCCUUUUGGAUUAGC
Ame-1-2   AUAGGUAC.CU...AG.AGAAAUUUUUGUGCCUCCCAACAU..................................UG.GAAAGGAA.A.UA.UA.C.UAGCUAUUUAAGCUAGUCCUUUCGAGGACUAGUUUUUGUUUUAUAUAUUCAUAUUUAUCCGAA
Csp-1-1   UAGGGUAU.UG...UG.GGAAACUUCAAUGCCUCCCAUUG...................................UG.GAAAGGAG.U.AC.UA.U.GCAGAUCCCUAUUUCUAUCACUCUAUUAACUUGCGCCUUUUUUAGGCGCUUUUUAUUUUUCUG
Dps-1-1   UAGGGUUG.UA...CU.GGAAACGGUUCAGCCUUCCACCACU.................................UG.AAAAGGAG.UCUG.UA.U.GAAUGUCUCACCGGGGCCCAGUUCCCCGCAGUCUAAGUUGGUUGAUACGCAUGGCCGUUCAAU
Sfu-1-1   CAGGGUGU.GC...CU.GGAAACGGGCAUGCCUCCCGUUG...................................CG.GAAAGGGG.A.GC.AG.G.UCAAACCGGGUUUCCAGUGCCGGAGCCUGCCCUUUUCGCGGGUUCCGGUUUUUUUGCCUCGUU
env-1     .UGGGCCA.CC...UU.GGAAACGGGAUGGCCUUCCGUAUUU.................................UG.AAAAGGGG.U.UG.UU.A.UCAUGCGGGCGGGACCCGGCGCGGCAAUUGCAGCACCGGAUUUGUCCAUAUCGCCCUCAUUAU
Swo-1-1   AUAGGUAG.GA...GG.GGAAACGCUCAUGCCUCCCGUU....................................UG.GAAAGGAG.A.GC.AA.A.AUGUAUGCAAGUUGACUAAUGCCGGGCUUAUCUUUCUGGAUGGGCCUGGUUUUUAUUUGUGUU
Dre-1-2   UAGGGUUU.AC...UG.AGAAAUUGGUAGGCCUCCCGUG....................................UG.GAAAGGAG.C.CG.GU.A.UAUGCUUGGUCGUGUUUUUUUGGCCGGGGUCUGCCUGCUUCUUGCAGAGAUCCUGGUUUUUAA
Hne-1-1   AGG.GUGG.CA...GUUGGAAACCCUGCCGCCUCCCGUGCU..................................UG.GAAAGGAG.C.CA.UG.C.GAUGACACAUCCCGGCGCAGUCGCGACGGAUGCCUUCGGGCGCAGGUUCAGUUACCUGCGGCU
Swo-1-2   UAGGGUAA.AC...AG.GGAAACAGGUUUGCCUCCAUCAUG..................................AG.GAAAGGAG.C.GG.UA.U.UAUAUUUAUAUCACGCUUUUCCAUACUAACCCCCUACGGCCACAACCUUAGGGGAAGCGCGAU
Swo-1-3   UUGGGUAU.GG...AG.GGAAACCUCUGUGCCUCCCGUU....................................UG.GAAAGGAG.U.AAGAU.U.AAGUGUAGUGCUUAAUAACACCCCUUGUCCGGGGUGAUUUUUUUUGUGGAGGUGUUGAAGGUG
Swo-1-4   UUGGGUAU.GG...AG.GGAAACCUCUGUGCCUCCCGUU....................................UG.GAAAGGAG.U.AAGAU.U.AAGUGUAGUGCUUAAUAACACCCCUUGUCCGGGGUGAUUUUUUUUGUGGAGGUGUUGAAGGUG
env-2     CAGGGUCA.GC...AG.GGAAACGUGCUGGCCUCCCGUUU...................................UG.GAAAGGAG.A.CC.UC.C.UAUGUUCGACCUUCAAGGUAGAAAUAUCAACAAACUCCGGGUUUCCGUGAGCGAGGCCU....
env-3     UAGGGUUA.GC...AG.GGAAACGUGCUGGCCUCCCGUUU...................................UG.GAAAGGAG.A.CC.UC.C.UAUGUUGGACCUUCAGGGUAGAAAUAUCAAUAAGCUCCGGGUUUCCGUGAGCGAGGCCUGCAA
Chy-1-1   AAGGGUACUAC...CG.GGAAACCGGGGUGCCUCCCGUUU...................................UG.GAAAGGAG.U.UA.UC.A.AAGCGCCUUUUUAAAACGGGGCGCUUUUAAUUUUAGGAGGCUAAAGAAAUGCUUGAUUCUUUU
Tet-1-1   UAGGGUAU.UG...AU.GGAAACAUCAAUGCCUCCCGUGU...................................UG.GAAAGGAG.A.GG.CA.A.UGCAUUUGCAUUUAUGCAAAGCAAGAAUGCGUAAGGUAAAGUAAUUGUGUCCUUGCUUUGCGA
env-4     UAGGGUUG.AA...AG.GGAAACUUCUCAGCCUCCCGGAAU..................................UG.GAAAGGAGAA.AC.CA.C.AUGUUAGACCGCCAAGGUAGAAAUAUAAACAAACUGAGAGUAUCUUUAACUGAGGUUUGCAAU
env-5     UAGGGUGG.AC...AG.GGAAACUGGUCUGCCUCCCGACUU..................................UG.GAAAGGAG.A.CC.UU.UCAUGAUAGACCAGCACGGUCGAGACAUUAAUAAACUCCGUAUAUCUUUAAGUGAAGUCUGUAAU
Swo-1-5   .GCGGUAU.AA...GU.GGAAACGCUUGUGCCUCCCUUAUUU..................................G.GAAAGGAG.A.CC.AA.A.UUCACUAUUAGUUAGGUUUAGCCAUUUUUAGACCCCGGUUAUAUAGGAUAAGCCUGACAGGUG
Swo-1-6   AUAGGUAU.AA...GU.GGAAACACUUAUGCCUCCCCGUAUUG................................UG.GAAAGGAG.U.AG.UG.A.AAGAACUGUUCAGUUAAUUGAACAGGUGUUACUGUGCUUUUUCACAACACACAACACCUGUUU
Swo-1-7   AUAGGUAU.AA...GU.GGAAACGCUUAUGCCUCCCCGUAUUG................................UG.GAAAGGAG.U.AC.UU.A.UUUAGCUGUUCGUCAAAAGAACAGGUGUUAAUGUGCUUUCUUUCAUUAACACCUGUUUUUUAU
Dre-1-3   UAGGGUUU.GU...CU.GGAAACGGGCAGGCCUCCCAAUG...................................CG.GAAAGGAGAA.AC.AC.A.UUAUGCCAAAUGGCGAGUCCGUGUACUGUUUUUUCUUUCUUACAGUACACGGUUUUAUUUUUU
Tet-1-2   UAGGGUAG.UG...CU.GGAAACGGUGCUGCCUCCCGCC....................................UG.GAAAGGAG.A.AU.AG.G.AAGGCUGCUAUUCUCCUUUCGCAUACUAAAACGAAAGGAGUUUUUUUAUGAAGGUUCUUUGUC
Swo-1-8   UAGGGUAC.GG...CG.GGAAACGGCCGGGCCUCCCAUGG...................................UG.GAAAGGAG.A.AA.UC.A.AAUGCGCUUACAUAGCAGGUUAAUUCCAUUUCUAAUUAACCCUGUGCUUGUAUAUUUCUGCGC
Swo-1-9   AUGGGUAU.AGA..UU.GGAAACGGCUAUGCCUCCCCGUAUUG................................UG.GAAAGGAG.A.CG.AC.G.AGGGCUUAUUUUAUAUAGGUUUAAAUGGUCGUGUUCUUUUUCCAGGACACGACCUUCUUGUUU
Swo-1-10  GUGGGUAU.AG...UU.GGAAACGAUUGUGCCUCCCCGUAUUU.U..............................UG.GAAAGGAG.CCUA.AA.A.AGCCUGGUUAAGAGCAGGUAGUACUUGCUGGGCCAUAUUGGGCAUAAAAUCCAAAUAUUUUUU
Swo-1-11  GUGGGUAU.AU...GC.GGAAACGGAUAUGCCUUCCCGUAUUG................................UG.AAAAGGAG.A.UG.CU.C.AGCAGGCAGGUACUUUAUAAGCAUAUCCAUAAAGGGUUCAAUUGCUUAUUAUUGUGCAUCUGU
Swo-1-12  GUAGGUGU.AGA..CU.GGAAACGGAUACGCCUUCCCGUAUUG................................UG.AAAAGGAG.U.UA.GU.A.UCAACUGUUCGAAAUGCAGACAGAUGUUGUGUGCUCUAAUUCACAUACAUCUGUUUUAUUAUU
Swo-1-13  UUGGGUAC.AC...CG.GGAAACGGGUGUGCCUCCCACUG...................................UG.GAAAGGAG.A.AC.CU.A.UAUCCAAACGGUUGCUAUUAAGGGGCAAGUUUGUUCUUUCACAGACUUGCCCUAUUUUAAUAG
Csa-1-1   UAGGGUGC.AGGUUUG.GGAAACCACUGCGCCUCCCGCUU...................................UG.GAAAGGAA.A.GA.UU.U.AAAGGAUACUCUUUCCGAGUUUGUUCAAAGCGGGGGAGUAUCCUUUUUUGUUUCAAAAGAGUG
Dac-1-1   CA.GGUGG.UC...CC.GGAAACGGUAUCGCCCCCAUGUU...................................UG.GAAAGGAG.A.UU.UC.C.GCCAUGAGCGAUGAUUGUUCACCUGCAACAACGGAGAGAACCCAUGGAUAAAAUUUUUCGCGU
Dac-1-2   AUGGGUUC.UU...CG.GGAAACUGGCGAGCCUCCCACUCU..................................UG.GAAAGGAG.C.UG.AC.G.GACAUUAAUCAUCAAGGUGACUGGUGCCGUUUUUUGUCUUUCCCGGCGAUGGAGGGUCAUUUU
Swo-1-14  AUAGGUGC.AA...CU.GAAAACGGCUGUGCCUCCCUUU....................................UG.GAAAGGAG.A.GU.AU.U.UAUCCUGGGUACCGGUGAAAUCACCCGGAGUCUUCUUUCGAGAGUUUAGGGUUUUUUUAAUUC
Dac-1-3   CAGGGUUG.CA...UU.GGAAACGGUGUGGCCUCCCAGUCU..................................UG.GAAAGGAG.A.UU.CU.U.ACCAUGCGCUUAACGUUAAGACACUGCAAAGGAGCCUGACACACAUGGAUAAGAUUUUCCGCG
Gsp-1-1   CAGG..CCGGG...CC.GGAAACGGCCGGACCUCCCUUU....................................CG.GAAAGGAG.A.CUGCA.A.AUAAUCACUGAAAGGGAGACUCCCCAUGAACAAGAUCCUUCGCGUCAACAUGACGACUCUCAC
Gsp-1-2   .AGGGUGC.CG...CU.GGAAACGGCUGUGCCUCCCUUU....................................UG.GAAAGGAG.A.GC.CU.C.AAAGUACCGGACCUGUUCCUGUAUCCAAAGGCCUCUUUCCAUCUGCGGACAGACUCUUUGAUG
Gsp-1-3   UUGGGUCC.UG...CU.GGAAACGGCAGCGCCUCCCUUU....................................UG.GAAAGGAG.A.GU.CA.CCGAAC.GGCAUAAAAAAACCGUUACCCGCGGGGUCUCUUUCCAUCAAGAAAGGGACCUUUUUUUA
Pca-1-2   AAGGGUUU.CG...CU.GGAAACGGCGAUGCCUCCCGCGUU..................................UG.GAAAGGAG.A.UA.AU.C.CCGGAGUUCCGUUCCAUUAAUUUCGCAUUGGGAGGAAAACAAGCUAUGCACAUUGUACGUGUC

          ..<<<<<<.<<...<<.<....>>>>>>>>>>><<........................................>.>...>>>>.>.>>.>>.>.>>>>.................................................................
          ..1002?2.??...2?.2....2?2??2?2001 22........................................2.2...0022.?.??.??.?.????.................................................................
          ●●GGU●●-●●---●●-GGAAAC●●●●●●GCCUCCC●Y●○---------------------------------UG-GAAAGGAG-●-●●-●●-●-●●●●●●●●●●●●●●●●●●●●●●●●●●●●●●●●●●●●●●●●●●●●●●●●●●●●●●●●●●●●●●●●●●●
                              GNRA                                                         SD+++
```

```
Ppr-1-1   ACGGGUUC.UG...GC.GGAAACGCCCGAGCCUCCCUUU....................................UG.GAAAGGAG.A.UG.AC.A.GCAGCAGGACAAACCACACCAAAAGGGAGAAAACAAGAUGGACAAGAUUGUACGCGUAAACAU
Gsp-1-4   GUGGGCUC.UG...CU.GGAAACGGCAGGACCUUCCUU.....................................UG.AAAAGGAG.A.AC.CU.C.UCGCCACGCUAUGAUUGCGUUCUAGGGGCUCCUUUAUCCAAAGGAGCCCUUUUUGUUUGUGGC
Gsu-1-1   ACGGGUGU.UG...CU.GGAAACGGCAGCGCCUCCCCUC....................................UG.GAAAGGAG.A.UU.CC.G.GGCCACGUGCGCCCAGAGACCCUUUCCAGCAGGAAGGGGUUUUGUGUUUUCCGCCCCUAUUCA
Gme-1-1   ACGGGUUC.CG...CU.GGAAACGGCGGUGCCUCCCUUU....................................UG.GAAAGGAG.A.AC.UC.U.UUAUUCCCCUGCGUGGGGUAUGGGGCCUCUUUCCGUUUGGAGAGAGGCCCUUUUUGUUGUGCA
Gur-1-1   ACGGGUGC.UG...CC.GGAAACGGCUGCGCCUUCCUUU....................................UG.AAAAGGAG.A.AC.AC.C.CUCCGUCAGAACCGUUUCCGCUGCAGGCCUCUCUCCAGCCGGGGAGGGGCCUUUUUCGUUCCG
Gur-1-2   AUGGGUUC.UG...CC.GGAAACGGCAGCGCCUCCCUUU....................................UG.GAAAGGAGAA.AC.CG.C.AGCGUUGCAAAACGUUCAGGGGCCUCCUUAUUCGGACAAGGAGGCCCCUUUUUCAUUGUUUCG
Pca-1-3   ACGGGUUC.CG...CU.GGAAACAGUGGCGCCUCCCUUUU...................................UG.GAAAGGAG.A.UU.UU.A.CCCGGAACCAUUCCAGAUAGUAAAAGAUUGGGAGGAAGCAAGAGAAUGGAUAAAAUCUUUCGC
Gme-1-2   CCGGGUGC.CG...CC.GGAAACGGCGGCGCCUCCCUUU....................................CG.GAAAGGAG.A.AC.CA.U.CAAUGAGAAACGCUAUCUAUAGAAAGGGAGCAAUCAGUUCAUGGAUAAGAUAAUCCGCGUAAA
Ppr-1-2   ACGGGUUU.CG...CU.GGAAACAGCGGAGCCUCCCUUC....................................AG.GAAAGGAGCU.UG.UC.C.UCGUUAGAGAGGGUACAUAAAAUCAUAGGGAGAAGAUAACCAUGGACAAGAUUCUACGCGUUA
Pca-1-4   CAGGGUUU.CG...CU.GGAAACGGCGGAGCCUCCCUUU....................................CG.GAAAGGAG.A.UA.UU.U.GCAUUUUGAGCAACCUCGUCUGAGGAAGGGAGAACAGCACAUGUUAGGUGGUUAUUGGCAAAA
Gur-1-3   ACGGGUUU.CG...CU.GGAAACAGCGGAGCCUCCCUUU....................................CG.GAAAGGAG.A.CC.UA.U.CCGCUCAGCGGAUUUUAAAACAUGAAAGGGAGCAAUGAACAAUGGACAAAAUUUUACGCGUAA
Gsp-1-5   ACGGGUUU.CG...CU.GGAAACGGCGGGGCCUCCCUUU....................................CG.GAAAGGAGUA.AG.CA.A.GCCCUUGCGGGUAAACACUAUGAUUGGGAGAAGAUAAAGAAAUGGACAAGAUUUUACGCGUCA
Pca-1-5   AUGGGUUU.CG...CC.GGAAACAGCGGAGCCUCCCUGUUUU.................................CG.GAAAGGAG.A.CC.AU.U.GCCUUGGGCUGUUUGAGGUAAUGUAUCCAAAAAGGUUCUUUCCGAUGAAAGGGGGGAACCUUU
Gur-1-4   ACGGGUUU.CG...CG.GGAAACUGCGACGCCUCCCUU.....................................UG.GAAAGGAG.A.AU.UA.A.CGUGGCUUGAAGCCACAACAUUCAAAAAAGGGAAAGGAGUAAACGUAUGGAAAAGAUUGCCAG
Gme-1-3   AUGGGUAU.CG...CU.GGAAACGGCGUUGCCUUCCUUA....................................UG.CAAAGGGG.ACCC.AG.C.GACGCGGAGGUAUGUCCGCGACGUUCAGGGGCCUUUCUAUCCAGAGAGCGCCCCUGUUGUUUU
Gme-1-4   AUGGGUAU.CG...CU.GGAAACGGCGUUGCCUUCCUUA....................................UG.CAAAGGGG.ACCC.AA.C.GGUGCGGAGGUGUGUCCGCGACGUUCAGGGGCCUUUCUAUCCAGGAGAGCGCCCCUUUUGUUU
          ..<<<<<<.<<...<<.<....>>>>>>>>>>><<........................................>.>...>>>>.>.>>.>>.>.>>>>...........................................................
          ..1002?2.??...2?.2....2?2??2?200122.........................................2.2...0022.?.??.??.?.????...........................................................
          ●●GGGU●●-●●---●●-GGAAAC●●●●●●GCCUCCC●Y●○-----------------------------------UG-GAAAGGAG-●-●●-●●-●-●●●●●●●●●●●●●●●●●●●●●●●●●●●●●●●●●●●●●●●●●●●●●●●●●●●●●●●●●●●●●●●
                             GNRA                                                           SD+++
```

alignment positions 481 · · · 640

```
Pca-1-1   CUUUCAAACAAUCUGGCUGGUACUCAUCCUGGCGCUGACGGGCAUCCCCUGUCCCGCAUGGAGCGCUCCGGAGCCUUUGACCAUUUAUUCCGGGGCAGGAUUAAUGAAGCCGAUGGACGAGCUGAAAGCAGGCUUCGAAGCCCGCUAUAACAUCCCGGUU
Mth-1-1   UUCCCGUUUUUUACCUGAAUUUUUACCUGGAGGUGGUGGGGCAAAGGCGAGGCAGUAAAUAUAAAUGUCGAAUUUAUAAUGAAAAUCGCGAAAGGGGAAUUAUCUUGCCUAAGGUACUGGUAGUCGAUCAGGGUAAAUGCACCGCCUGCCGGCGCUGCG
Hdu-1-1   GCGUCAAUACACUUAUCUGAGAUUAUCUAUUACCGAGGUAUGCAAUUUCCGUUGUAAUUAUUGCUUGCCAGAUGGCUACAGACCACCAUCUCAUAAACAACGUUUUCUUAAUUUAGUAGAAAUUAAACGGCUUGCACGCACUUUUGCCAAUCUUGGUACA
Dre-1-1   AAAUAUGACAAAACUUUUUCGUGUGAACAUGACAACCAAAGAAGCCAAGAUUGAAGAGCUUCCCCAGAAGUAUGUUGGGCUCGCUGGCCGUGCCUUGACCUCAAGCUUUGUACUUGAUGAGGUAGAACCUAAAUGCCAUCCCCUGGGACCCAACAACAAA
Ame-1-1   UUUUUUGUAUUUAAGUGGGAUAUGAGUCGACGCCAUAGCGUCAGACGAAUUUGACGGGGGGAUAUUCAAAUGAUUGAGUUAUUAGAUACUGUAACACUAGAAGAGGCAAAACAGAUUGUCAUUGAAAAUUUCAAAGAAACAUCCUUGGAUAGUGAAAAG
Ame-1-2   AAAAAGUUAAUCCAAACACCAGCACAUCAUUAAGUAGAUGAUUAAGGGGGAAAAUACAUGAAAGACACAUUCCAAAGAAGCAUUAACUACAUGAGGAUUUCUAUCACGGAUCUAUGUAAUUUGCGUUGUCAAUAUUGUAUGCCUGAAAAGGGAAUCUAUA
Csp-1-1   UAUAAAAUCUCAAACUGGCAAGAAGUCCCUUAUCUAAAGUUAUAGUUGAAAUUUAAACUAAAGGGAGAGAUUGGUUAUGAAAAGAAGGUUUAGAGAAUUAUCGCUUAUUUUCCUACUAUUCCUGUGUAUUUUUGCUGUUGCUUGUUCUAAUCCAGCAACU
Dps-1-1   UACUUAUCUUAGGCUUUCCAUAACCGAUCGUUGUAACCUCCGUUGUCGUUACUGUAUGCCGGAAGAGGGUGUCCAGUUCCUUGAUCACUCUGAAAUUCUCUCCUAUCAGGAACUCCUCCGUAUCAGCCGUAUUUUUGUCGGAUUCGGGGUGAAUAAAAUU
Sfu-1-1   GGCCCCGCAGGGGCCGGAGCGGCGGGGUGGAUGGUCCGGGCGGGGAGGUUUGGGUGCCGCGCGGAGUCCCGCGGGUUUCCGCCGGGGAUGAAACGGCGUGCCGAUUGCGGCGGUUCACGCGGAUCGCCGAGUGUCCUGAGACAGCCUGCGGAAUGCG
env-1     UUCACGCUCCCGAUCCUGGGCAAAAAGCCGGAAGUCAUUUGACUUCCGGCUUUAAUCCAUCAAAGGAGGGAAAUUACCUUGAAAAACUAUGACUUACGAACAAUUAUUUUUGUGGCUUUGUGCUGUGACCUGGGUCUGUUCGCCAAGCGCGUCAUCGCGC
Swo-1-1   UAACGAGGUGAAUUAAGUCUCCCACUUCUGCCGGGUUCCUAUUAACAAACAGGUGGCGGGUUCUAACCCCCGCCCUCGUUGCAGCGGUGUGUUGAAAGUGCUCCGCAGUUUCUUCCGAUGCUUAAAGGGUUGUUUAUUGAUUUAUUAAGUGGCCUCGUAU
Dre-1-2   UUUUUUAUGAGGAGGCAGUCAUGACUAAGGUAAAAGAAUUGGUUAAUAAUAAACAAGUGGAAUUGGACAGGUUAACCGAUGUGUGUGAAAGUUAUCUUGAUGAUAUAAAUAUUCUGUCUCAAGCUGUUGACUAUCUAUUACAGCAACGUAGCGAAGGAAA
Hne-1-1   GUCGGUUACAGAAGUCUGCAAUUUUCGAUGUACCUACUGCUUGCCGGAUGGAUACCGAAAAACCGGUUCCAUGGACUUUCUCGCCGCAGACGAGAUUGAACGGCUCGUGCGCGCCUUCACCGGGCUUGGCAUCCGUAAGGUCCGGCUGACAGGCGGCGAA
Swo-1-2   UUUUAUUUCUUGUCGUUAAACCUUGGAAAGGGGUUCGGCGGUGAAAGUAUCAAGCAUUGCCGGGUUUCCGCAAAGGAAUCUGGUUAAGCCUGAUAUUUAUGCCCAAAAUGGGGCCCUGAAUCAAGGAGAAGUUGCCUUAUAGUUCAAUUCCAAAUUAUCU
Swo-1-3   ACCGAUCAGAAGGAACUGGUGGUAAAGCUAAAGGAAUUAUUUCCCCAGGGGAGAAUCACUUGUACCGAAGCUCAUGAUCUGGCCCGGGAGUGGGAUAUGGAUCCUCGGGAGCUGGGAGAAGCCUGUAAUGAGGCAGGCAUCAAGAUUUCUGGUUGUGAAU
Swo-1-4   ACCGAUCAGAAGGAACUGGUGGUAAAGCUAAAGGAAUUAUUUCCCCAGGGGAGAAUCACUUGUACCGAAGCUCAUGAUCUGGCCCGGGAGUGGGAUAUGGAUCCUCGGGAGCUGGGAGAAGCCUGUAAUGAGGCAGGCAUCAAGAUUUCUGGUUGUGAAU
env-2     ................................................................................................................................................................
env-3     CAUGGCCUGUAGUUAUUGUGUCACCGGAAUCGAGGAUCAUCAGAUCGCCCCGGACCAGUUGGUCAUGCCGGAUUUGCUCCGGCUGGUGGAGUUACUUCAUCGUCACGCGGGGAUCGAAAAAAUCCGCAUCACCGGUGGCGAACCCCUGCUUUAUCGUGAU
Chy-1-1   AACCGGGAAAUAAACUAUUUGCGGAUUUCGGUUACCGACAAAUGUAACCUUAACUGUUUUUACUGUAAACCCGGAAAUUUUCAAGAAUUUUCGCCGGGAGAUAUUUUAACCUUUGAGGAAAUUUUAGAAGUAGUCCGGGCCUUUUUACCCUUGGGUUUAA
Tet-1-1   AAACGAUGCAGUGCCUUCUCCGGAAGGCAUUUUUUUGUACCUUUGUUAGGGACUAAUUUCAAUGGUAAAAGCUAAAGAAUGGAAUCUUUCCAGUUUUAAUGGGUAAGGUUUAAACAAAAACUAAUAGUGAUGAUGGAGGAUGAAUACCAUGGCAAGAAUA
env-4     AUGGCCUGUACUUAUUGUGUAUCCAGUUUAAAUGACCAUAGACGCUCACCAGGAGAAUUAAGCACAGAAAAAUUAUUGAAACUAGUUAGUAUGUUAGUGAAACAUGCUGGUAUUGAAAAAGUUCGUUUGACUGGGGGAGAGCCUCUAUUACAUGCUAAUC
env-5     AUGGCUUGCACGUAUUGCGUGACAAGCCUGAGCGAUCAUAAACGUUCUCCGGAAGAAUUAAAUACGGACCAGAUACUAUAUCUUGUCCGAUUGUUAAAAGAUCAUGCAGGAAUUGAGAAAGUACGCCUAACUGG..........................
Swo-1-5   AUUAUUGGACUUUCUUCAAAUACGGGAGGCUCUUUUUGUCUGUAGGUUUGAUUUCUUGGGAGUUGUUAGAAUUUGAAAGGAGUGAUGGGAGUGGAACUGAUCAAAAUCACUAUUGAUGGAAAGGAGGUUGAAGUCCCGGCGGGUACAACAAUAAUGAAGG
Swo-1-6   UUUGUUUAAUUGAAUAGAACCGGCUCCAAGCCUUUGAGCCUAAUGACUGAAACUCAUUUGUCAGGGAGAAUCGGACUUUUAUGGUGGAAAAAGCCUGAAUCCCUGAGCCCGUUAAAAAAGUAUAAAAAUUAUGGACGAUUAAUGCAUUUUAUACUUUCAU
Swo-1-7   UUAACUUAAUAUGUAUUCCGAGCCCUCUUAACCUGCAGACAAAUCCAGGUUACAGGGCCUAUAGGUAUAAGUGGAAACACUUAUGCCUCCCCGUAUUGUGGAAAGGAGUAGUGAAAGAACUGUUCAGUUAAUUGAACAGGUGUUACUGUGCUUUUUCACA
Dre-1-3   GUUUUGGGGGGUGAAAUACUAGCACAGCAAAAAAUGUUACAAAUUCAACACCGUAUAUUAAAUACAUAGGAGGUUUUAUCAUGAAGAAUCGGUCAUUAAAAUAUCUUGCCUUGCUGUUAACAAUGCUUAUGCUGGUAGUUGUCGGCUGUGCUCAGCAACA
Tet-1-2   AAUAGUUGGGGUGGGUAUUUUUUCUGAAUGUUGUUAUUGUAGGUCCUAAGAGUGAUCCUGCAAAGGUACAUCAAGCGGGUUCCCGCGGCGAGGGCUAAAGGUGGCUUGUUUCCGAAGGUAAUAUGAGGUUUCAAAUGCUUGUAGAAGAGCUGUGAGAAAC
Swo-1-8   UUAAUAUAAAACCAAACCUGGUAAACCUUUUUGCCAGGUAUUUUGUUUUUUGCAGCCAGUUUUAUUAUACAUUGCUGCGUUAAAAGCAAUAUUGUUACUUGAAAAACCGAACCCUUCAAAACCUUAGAAGGCCUCUGUGAAGAUCACUUUGAAAAGAGAU
Swo-1-9   AAGAAGGAAAAAAGCGGGAUUCGUUCUGGUUCUCCCUAAGGUUUUGGGAAGCAAACGGGUACAGGUAGGCAUAAAAGCUAACGUCUCAAUUUAAUCAGGUAAUAUGCUGCAAGGCUAUUACAUACAAUCCCAAUCUUUUUCUCCCCGCUUGCAAACAAGC
Swo-1-10  UAUGCCCCUGGCCUUUGCUGGAGGCAUUUUUGUUUAUCCCAUAAAUGCAAUCAGGUACUCUGGCAAAAGAGUAUCUGCUGGUAGUAGUAGUUUUUAAGGGAGGUAGAGAUUGGUUAUGAAAUUUACUCGUCGUGGUUUCCUGAAGCUUUCAGGUCUCUCC
Swo-1-11  GUCCAGCAUUUCUUUAGCUGGGCUUUUUUAAUGCAGAUUUUAAAUUAUCUCAAAGUAAAUAAAAUUAGCCGGAAGUAUCGCUGAACUAGGAGUAAUGAAUAUCGUUCGGGCCAGUGCUUUUUAGUUAUCAUCAAAAAAGCCUCACAACUAUAAGGUUGU
Swo-1-12  UUAAUUGAAUAUUUGUUUCCAAGCCUUUGAACCUAAGGUUUAUAACUAUGGUUCCCAGGCCGAUGGGUAUAGAUUGGAAACGGCUAUGCCUCCCCGUAUUGUGGAAAGGAGACGACGAGGGCUUAUUUUAUAUAGGUUUAAAUGGUCGUGUUCUUUUUCC
Swo-1-13  UUAUUCCAGCCCGGUACUGCUUUCUGCCGGGUUUUAGUAUUUCUAGGAUAAUUAACGUUCUAACUGUAAUAAUGGAGGAGGUUAUAGAUAGUAAAGGAUUAAUUAAGUCGAUAAUAUGAGGCGAGAAACUUAUAUAUAGAUAGAGAUAUUAAGAUAGAGA
Csa-1-1   ACUAUGUUAAAAAAAAGGAGUGUUAAAAGAUGAAAAAAGUUAUAUUUAAAGUUGUUUUAGUAUGCAUUCUUUUAACUUUUGUCUCUACUUUCUCACUGUAUUCUUUUUCAAAACCUGUUAAAACAUAUAAAAUAGCAACAACUACAAGUAUAUAUGACAG
Dac-1-1   UAACAUGACCGACCUGACCACCACUGUUGAAGAUGUCCCUGAAGCAUGGCUCGGCCUCGGUGGUCGUGCUCUGACUUCAACCAUCGUUGCUACCGAGGUUGAUCCUGAAUGCCAUCCCCUUGGCCAGUUCAAUAAGUUGGUUUUUGCCCCCGGCCUGCUC
Dac-1-2   AUUUCAACCCCUUUGUUUGAAGGAAAAAUUGUUAUGAAACAACUGCGAAUGGUACUCUUUUCAUUGAUGAUCGUCCUGCUGCUGGCUGGGGCCGCCUUGGCUCAGGAGCACCUGAAGCUGGCCACGACCACAUCGACGGAAAACUCCGGUCUGCUCAGUG
Swo-1-14  AAAUUGAGUGUCGGUUCUGCGUCUACAAAUAAUAUUUAGGAGGUAUUAUUGUGUUGAAGUUCAGGCAGGAAUCCAGGUCUUCUUUGGUAUUAGCGCUGGUUGUUCUUUUUUGCUUAUUCCUAACCUUACCGGUACUGGCUGAAGGUGGUAGUGGUGACGG

          ................................................................................................................................................................
          ................................................................................................................................................................
          ●●●●●●●●●●●●●●●●●●●●●●●●●●●●●●●●●●●●●●●●●●●●●●●●●●●●●●●●●●●●●●●●●●●●●●●●●●●●●●●●●●●●●●●●●●●●●●●●●●●●●●●●●●●●●●●●●●●●●●●●●●●●●●●●●●●●●●●●●●●●●●●●●●●●●●●
```

```
Dac-1-3   UUAACAUGACCGACCUGACCACCACCGUUGAAGACGUCCCGGCUGAAUGGGCUGGUCUCGGUGGUCGUGCCCUGACCUCAACCAUCGUAGCUACCGAGGUUGAUCCUGAAUGCCAUCCUCUGGGAGAAAAAAACAAACUCGUUUUUGCCCCUGGCCUGCU
Gsp-1-1   CACCGGCACCGAAUCGGUACCAAAGGAAUGGGCAGCUCUCGGCGGUCGCGCCCUGACUUCCACCAUCGUGGCCAAGGAGGUUCCCCCCACCUGCCACCCCCUUGGGGAAAACAACAAGCUGGUCUUUGCCCCUGGACUGUUGAGCGGCACCACUGCCGCU
Gsp-1-2   CAUUUGGGGGAGACCUUUUCCCCUGUUAUUAGAAAACAGGAGGGUACGAUGAAGAAACGCAGCUUAGCAGCAACAGUGGUGGCAAUGGGCCUGGUGCUCAUCCCGGGUGUUCAUGCCAAAACGCUUGAGGAUGUGCUCAAGGAGAAAGGGGUCAUCACCG
Gsp-1-3   UUUCUGAAGCACGGGGUAAGGAGCCUUCAAUGUCGCUGGCAAAGAGGCCCUUAAGUACAUUCUGUAUCCACAGGAGGGGAUGAACUAGGUGGGGUUUUUUUCCGAAUCACUGCAGACAGCUUUCAGCCUCAUCAUGUCCAUGGACCGGGAGGUCUAUACG
Pca-1-2   AACAUGUCUGAUCUGACCGUCAAGACCGAAGAGGUACCCGCCGAAUGGAAAACCCUCGGUGGCCGUGCCCUGACCACCACGAUUGUGGCGGCCGAGGUAAACCCCACCUGUCACCCGCUCGGUCCCAAUAAUAAACUCAUUUUUGCCCCCGGAAUGCUGU
Ppr-1-1   GACCGACCUGACCAUGAAGACCGAGGCGGUGCCGGAAGCCUGGGCAGCCUAUGGCGGGCGCGCCCUGACCUCCACCAUCGUGGCAGCCGAAGUCCCCCCACCUGCCACCCCCUGGGCCCGAACAACAAACUGGUAUUCGCUCCCGGCCUGCUAUCCGGA
Gsp-1-4   UGAUCACGUGCUUCGAACAUGACUUCAAAACCAGAUAAAUGAUUCUCAACUCUCCGAGCCACGCCGCCUAAAGGGUAGUUUUCCCAUGGCGACCGGCUAGGGUGCCGCUGGAAACGGCUGUGCCUCCCUUUUGGAAAGGAGAGCCUCAAAGUACCGGACC
Gsu-1-1   AUCCCGGGAGGAAGCAUCCAUGGUCAUCAUUACCGACACCCCCAUCGAUCCCACAUCUGCCUACGGGCUUCUCUCAAAAGGGACUUCAGGCUCGAUUGUCCUUCACUACGCCGUGGUGAAACAGGACGCCGGCAACAGCGCGCCUACCGCCUGCAUCGAC
Gme-1-1   CCGGCAGGCCUGGCCUGUUCCGGCAUGGUGAGCGUGACUCACAACUAUACCCCCCUAGGAGGAACACAUGAAACACAGAAACAUCGCGGCAGUUUCGGCCAUUCUUGCGGCCACUAUGGCCUGCACCACAGCGGAGGCCAAGACCUUGGAGGACAUCCUC
Gur-1-1   GCUGCGCCGCUGCUGGUCCCUUUUGCACAGCCGCUGGUCGAGCACCCCGCAAAAUCCCCGGGAAUUUGCAUCCUGGCCAAUCCAAGAUACACUUGAAAACGGCAGUCAGGCAGUCAUUCCAUGUUAUUUAGGGGACGCUUGAAAUGUUCAAGAAAGGGGA
Gur-1-2   CCCAAUUCCGCAUGAUGACAAGGAAGGAAAUAAGCAAUGGUCAUAGUGUCAACAGAACCCAUCAAUCCAUCGGAAGCCUACGGGUUGAUCGAAAAGAGCAGGUCGGGAUCGGUCGUUCUUCAUUACGCAGUGGUCAAAGAGGAUAUCGGCGUCGGCAAAG
Pca-1-3   GUUAACAUGACGGACCUCACCUGCAAAGUUGAGGAAGUCCCCGCCGAAUUCGCAACCUUGGGUGGUCGCGCCCUGACCUCUACCAUCGUUGCGGCGGAAGUACCCCCCAGCUGUCAUGCCCUCGGCCCCAACAACAAGCUGGUUUUCGCCCCCGGCCAGC
Gme-1-2   CAUGGCCGACCUGAGCACCAGGAUCGAGGCGGUCCCCGCUGAAUGGGCGGGGCUUGGCGGCCGCGGCCUGACCUCGACCAUCGUUGCCGCAGAGGUCCCCCCACCUGUCACCCCCUGGGCCCCAGCAACAAACUUGUCUUCGCCCCGGGACUCCUGACC
Ppr-1-2   ACAUGACAGACAUGACCACCAAGGUCGAAGAAGUACCGGCUGAAUGGGCUGGUCACGGUGGACGUGGCCUGACCUCAACCAUCGUUGCCGCAGAAGUACCCCCCACAUGCCAUCCGCUGGGACCGAACAACAAGCUGGUAUUCGCACCUGGCCUGCUCUC
Pca-1-4   GAUCGCGGUCAUUGAUUUGACCAACGAAACAGUGGAAUACAUCGAGCCCAGUGAAGAUGACCUGAAAAAAUAUAUUGGCGGCAGCGGUCUGGGUGCUAAGUUGCUGUAUGAAAACACCGCGCCCGGUAUCGACCCGUUGGGGCCGGAAAACGUCCUGAUC
Gur-1-3   ACAUGACCGACCUGACCACCAAGCUUGAGACAGUGCCCACCGAAUGGGCCGGUCUCGGCGGCCGCGGUCUGACCUCCACCAUCGUCGCCGCAGAAGUGCCGGCCGACUGUCACCCCCUCGGACCCAACAACAAGCUGGUAUUUGCUCCGGGACUCCUUAC
Gsp-1-5   ACAUGACCGACCUCACCACCAGGACCGAGCCGGUACCGGCCGCCUGGGCCGGUUUGGGGGGCCGUGCCCUUACCUCCACCAUUGUUGCGGCCGAAGUGCCUGCCACCUGCCAUGCCCUGGGUGAGAACAACAAGCUGGUCUUUGCCCCCGGGCUUCUCAC
Pca-1-5   UUGCGUCGGGUGUUAUUGUAGCAGAGAUUUUUUGACUUGCAGGGUCAGGUCAAAAAAAAUUGAGCAAUACGCUGUUUUAUCGAUUGCGCAAUGUUCGAUCCGGGGGUAGGACUAAAAGGAAAAGUCCUUGUCUUGAAAAAUAUUUAACGGUGCCGUGCCG
Gur-1-4   GAUCGACAUGGGGGCGGCAGGCGGCCCCAUGGUAACGUUUGAAGAAGUCGGCGAGUAUGCCGGACUCGGCGGCAGGGCGAUGACGUCCCUCAUCAUCGCCAAGGAAGUGCACCCCCUCUGUCAUCCGCUGGGAGCGGAGAACAAGCUGGUCAUCGCCCCC
Gme-1-3   UUUGCGGGAUAGACCGCGUACAACGUGUCCGGUGUGAUGAUGCCGGAUUACGACAACAAGUGGAGAGAGGAGGAGGAUAUGAAAUACGAAGAGGUCGGAUGUUAUCUGGAAAUCAAUCUGGCAACGGGAACUAUCGAACGGGUGGCGCUGGAACCCGAGG
Gme-1-4   UUUCCCUCCGAGGCUCUAGUGCGGGCUGUCGGCGGAAAGCGGGUCUUGAAAAAAUUUAAAACCGUCUCCGAGCCGCGGCGCCUAAAGGGAACAUCCCCAUGGCGGUCCGGCCUAUGGGUAUCGCUGGAAACGGCGUUGCCUUCCUUAUGCAAAGGGGACC
          ................................................................................................................................................................
          ................................................................................................................................................................
          ●●●●●●●●●●●●●●●●●●●●●●●●●●●●●●●●●●●●●●●●●●●●●●●●●●●●●●●●●●●●●●●●●●●●●●●●●●●●●●●●●●●●●●●●●●●●●●●●●●●●●●●●●●●●●●●●●●●●●●●●●●●●●●●●●●●●●●●●●●●●●●●●●●●●●●●●●●●
```

alignment positions 641 · · · 724

```
Pca-1-1   CAGCUUAUCUACGGCGGUUCCGGCGAACUGUUCGGCAUGAUCGCCACCCGCAAAGCGGGUGACGUUUUCAUCCCCGGAGCCGA.
Mth-1-1   AGCUGGCCUGCUCUUUCAAACAUACCGGCGAAUUCAAUCCUGCGGCAGCCCGGCUGACAGUGGCUGUUUUCCUUGAUGACGAUU
Hdu-1-1   GAAAAAAUCCGAAUCACGGGUGGGGAGCCUACUUUACGUAAAGAUUUUCUAGAAAUAGUCGAAACCAUUUCACAAACAGAAGGC
Dre-1-1   CUAAUUUUUGCGCCUGGUCUGCUGGGAGGUACCAAUUGUGCUAACUCCGGACGUCUUUCCGUAGGAAGUAAGAGUCCUUUAACC
Ame-1-1   GUACCGUUAUUUAAAGGGGGGGGGAGGGUCCUUGCUACGGACAUCACAGCACCUAUCGAUGUGCCGGGAUUUGACCGCUCUACU
Ame-1-2   AAAAAACACAUCAAGAUAUAUUAACCUUGGAAGAAAUCGAACAAAUCGUCAGAAUAGGUGCGGAAAAUGGUAUUAAUAAAGUAC
Csp-1-1   ACCACACAGGAAUCAAAUGGAAGCAAUGUACCAAGUGCCACUGUUGAAACGACCCCUAUAAAAAAUAAUGUAUUGAUUCUUUCA
Dps-1-1   AGGAUAACCGGUGGAGAACCCUUUGUUCGCCGGGACUGUGUUGAUUUUAUGCAGGCCCUGAUGGAGCAAACAGCGCAUUUGGA.
Sfu-1-1   AUACGGAAGGUCACCAUUCACCAUAAAGGAGAGGAGCCAUGUUGAGAAAAUCGGUUUUGGCUGGUGCGGUGCUCGUUGCCCUGG
env-1     CCUUUGCAAAUAUCAUCACCGAUACACUUCAUAUUCCCGGCGGCAUUGGUACCAGCUUCUCCCUGAUGUUUUUGGUAAUUGCGG
Swo-1-1   GAGGAGAAGCAAAAGGGUUUUCGGGCAAGAAUGAUUUUGAAACCAUGGGAGGGUUUAUUGCUAUGGAAGCAGCCGUAAGCAGUU
Dre-1-2   GUUAUCUCAAGUCCAUGGCGGACAGUUUCGCCUGGAAGGUGAGGAUAUUUUGUUAAAGGAAGGUUACCCCCUAGAAGUUCUCAU
Hne-1-1   CCGACUGUCCGGAAGGAUCUGACGCAACUGAUCGCCCGCAUUUCAGCGACGCCGGGGGUUGAGAAAGUCGCCCUGACGACAAAU
Swo-1-2   UGGAAGGCACCCCCUGUUUCAGUGUGGGGGUUUUUUUGUUGCCUAAAAAAAAUGGAAGGAGUGAUGAUGGUGGAUUUGGUAAAAC
Swo-1-3   UGGGCUGCUUUUAAGCUGGACAAGCUUUUUCCCUGCAGGGAGGUGGGAAAAUAUGCUGGAUAAACACGAAAGAAAUAUAAACU.
Swo-1-4   UGGGCUGCUUUUAAGCUGGACAAGCUUUUUCCCUGCAGGGAGGUGGGAAAAUAUGCUGGAUAAACACGAAAGAAAUAUAAACUA
env-2     ....................................................................................
env-3     UUGAUUCCCUUCAUUGAGGGGCUGACUAAAGCCGGUUUGGAAGACAUCGGUCUGACCAGCAACGGACUUUUGCUAGCAAAAUCU
Chy-1-1   AAAAAGUACGGAUUACCGGCGGCGAGCCAUUGGUUAGAAAAAAUCUUUUAUAUUUAAUUGAAAAUAUUGCAGCUUUACCGGGUA
Tet-1-1   UCAAAGAAGUUUUUGAUGAUCCUUUUAUAUUCUAUUUUGUUGUUUAGUUUAUUUUCUCUGAACGGUUGUACAGGUUCUAAAACU
env-4     UAACCGAAGU..........................................................................
env-5     ....................................................................................
Swo-1-5   CCGCUGACAGUGUUGGGAUUAAUAUUCCAAGGCUAUGUUAUGACCCUGACCUCAGCGCCCUGGGAGCUUGUCGCCUGUGCGUGG
Swo-1-6   UUUUGCUGGGAUUGAGUCUUUAAUCCAUGGUUUAACGGCCGCGGUAUAAGUGGAAACGCUUGUGCCUCCCUUAUUUGGAAAGGA
Swo-1-7   ACACACAACACCUGUUUUUUGUUUAAUUGAAUAGAACCGGCUCCAAGCCUUUGAGCCUAAUGACUGAAACUCAUUUGUCAGGGA
Dre-1-3   AACCCCUAAGGAAGAAGUGAAGAAGGAAGCCGCAGAACCUGCAAUAAAAGAUGUUAUUCUAGCCACAACCACCAGUACCCAAG.
Tet-1-2   CUGAACUUGCGAGAAUGUUUUAUAAUAGUAAAAGUGGCUUUCUACAGAUUUAGAGCAUAGUCGCAGAAAGGGCAACGAUAAGUA
Swo-1-8   GUUUUACGUAGAAUAAUAUGGAAAAGAGUAAAGCGAGUAUUAUAGCGAGUAUUAUUUAGAAAAAAGAUAGGAGGGAUCAUGCUG
Swo-1-9   GGGUUUUUUUUUAAAUCAACCCAUGAAGGUUUAAUAUAUUUUCUUUGUCUUACGAUAGAACCAUCCAUAGUUACUUAAGUGCUG
Swo-1-10  GUUGCCGUAGUUGCUUCCGGCUUGGGGUUUGACUUGGAAGCAACUGCCGCACACGGGCACGCACUGAAGCUGGCAGGAUGCAC.
Swo-1-11  GAGGCUUUAAUUCUGUUACUAUAGCUCUAUUAUUAUUCUAUUCGCUGGGAAUCCUCAUGCCGUAGAAGGAACGCCAUACGAAGA
Swo-1-12  AGGACACGACCUUCUUGUUUAAGAAGGAAAAAAGCGGGAUUCGUUCUGGUUCUCCCUAAGGUUUUGGGAAGCAAACGGGUACAG

          ....................................................................................
          ....................................................................................
          ●●●●●●●●●●●●●●●●●●●●●●●●●●●●●●●●●●●●●●●●●●●●●●●●●●●●●●●●●●●●●●●●●●●●●●●●●●●●●●●●●○
```

```
Swo-1-13   AAGAUUGGGGGGAUUAAAAUAUGCUUUGUAUAUUUCAAAAUGGGGAAUAUUAGUUCCUCUUUUUUAGUUUAUAGCCAUAUCGUA
Csa-1-1    UGGAUUUUUAAGUUUUGUUGUUCCUGAGUUUGAAAAAAAGAAUAAUAUGAAACUUAAUUUUAUCUCUGUUGGCAGUGGUCAGGC
Dac-1-1    AGCGGCACCGCUGCGGCCCAAUCCGGCCGGAUGUCCUGCGGCGCUAAAAGUCCGCUGACCGGCGGCAUCAAAGAGAGUAAUGCC
Dac-1-2    CUCUGCUGCCGACCUUUGAAGCGCAGAACAACUGCAAGGUCGACGUGAUUGCCGUGGGUACCGGCAAGGCAAUCAAGCUGGGUA
Swo-1-14   GUCUGGAGGCGGGCACGAUGCCCCUCUGGGGUUAGCUUCUUCCAACCCUGCUGAUGGGGCAUCAAAUGUAGCAGUUAAUACUCA
Dac-1-3    UAGUGGUACCGCUGCGGCCCAAUCCGGUCGUAUGUCCUGCGGCGCUAAAAGUCCGCUGACCGGCGGCAUCAAAGAGAGUAAUGC
Gsp-1-1    AACAGCGGGCGCUUUUCCGCAGGCGCCAAAAGCCCCCUCACCGGCACUAUCAAGGAAAGCAAUGCCGGCGGGUUGGCCGCCCA.
Gsp-1-2    AAGAGGAUUACAAGGAAGUAACCAGGAGCAAGGCGCUGAAUUACAAGCCGGGCAAGGGGUUCACCCUGACCUCUGCCGACGAAA
Gsp-1-3    ACCGUCUGGACUUCCAUUUACGUAUCCAUAUGGUCAACGCUUUUUGCAUCGGUCAUCGGCAUCCCUGCCGGUAUUACUAUCGGC
Pca-1-2    CGGGCACCCCGGCCGCAAACUCCGGUCGCAUGUCCUGCGGCGGUAAAAGCCCUCUGACCUACGGCAUCAAAGAGUCCAAUGCCG
Ppr-1-1    ACUCCGGCCGCCAACUCCGGUCGCCUCUCCGCCGGCGCCAAGAGCCCCCUCACCUACACCAUCAAGGAGAGUAACGCCGGUGGG
Gsp-1-4    UGUUCCUGUAUCCAAAGGCCUCUUUCCAUCUGCGGACAGACUCUUUGAUGCAUUUGGGGGAGACCUUUUCCCCUGUUAUUAGAA
Gsu-1-1    UACCGGUUCGACGCUGGUGCGAUCGAGGAACUGGAGGCAAUCUCCGCCGAGCUGCGCCAGCAGUGGCAACUGGAAGACGUCCUU
Gme-1-1    AAAGAAAAGGGUGUCAUCACCGAAGCCGAUUACAAGGAAGUGACCAAGAGCAAAGCGUUCGAUUACAAGGUUGGAAACGGCUUC
Gur-1-1    UCUGGUAAGGCAUGCGGCCAUGCCGUCGUGGGGGAUAGGAAGCGUAGUGAAUCCGGCCAAGGAGGGAAACCUCCUGAUUCGGUU
Gur-1-2    CCACAUCAUGUAUCGAUUAUCGCUCGGAAGGCGACACCAAGGCCGAGUUGGGGAUUAUUUCGGGGGAACUGAAGGAAAAAUGG.
Pca-1-3    UGUCCGGCACCCCGGCCGCUAACUCCGGCCGUAUGUCCGCCGGCGCCAAGAGCCCCCUGACCGGUACCAUCAAGGAGUCCAACU
Gme-1-2    GGAACGCCGGCCGCCAACUCGGGCCGCCUCUCGGCCGGGGCCAAGAGCCCCCUGACCGGCACCAUCAAGGAAAGCAACGCCGGA
Ppr-1-2    CGGAACCCCGGCCGCCAACUCCGGUCGCCUCUCCGCCGGUGCCAAGAGUCCGCUCACCGGCACCAUCAAGGAGAGUAAUGCCG.
Pca-1-4    UGCAUGACCGGUCCUUAUGUCGGUACCAAGGUGGCUCAGUCGGGUCGUUGGGAGCUGAUCACUAAAGGUCCUCUGACCGGGAUU
Gur-1-3    CGGCACCGCCGCUGCCAACUCCGGCCGUCUCUCCGCCGGGGCCAAGAGCCCGCUCACCGGUGGCAUCAAGGAAAGCAACGCCGG
Gsp-1-5    CGGUACCCCUGCCGCCAACUCAGGCCGCCUCUCUGCAGGGGCCAAGAGCCCGCUUACGGGCACCAUCAAAGAAUCGAACGCCG.
Pca-1-5    GUGGCUUGGAUACCUGUUGUCAGCCCGGGGCUCGCCGUUUUCGAAUUUUCCGCUUAGUUUUUUUUGUGGGUUAUGGAAUAUCCU
Gur-1-4    GGCAUGCUGAGCGGCACCACCGGCUCCAUGACCGGCCGGCUGUCGGUCGGCUGCAAGAGCCCGCUGACCGGCACCAUCAAGGAA
Gme-1-3    UAAUGAGAGAGCAUCUGGGACGUCCCGGGGCCUGCAUCGGGGAAGCGUGCGAGGGAUUCUGCCAGCAUUCGCUACAGACCUGC.
Gme-1-4    CAGCGACGCGGAGGUAUGUCCGCGACGUUCAGGGGCCUUUCUAUCCAGAGAGCGCCCCUGUUGUUUUUUUGCGGGAUAGACCG.
           ....................................................................................
           ....................................................................................
           ●●●●●●●●●●●●●●●●●●●●●●●●●●●●●●●●●●●●●●●●●●●●●●●●●●●●●●●●●●●●●●●●●●●●●●●●●●●●●●●●●●○
```

References

[1] Zasha Weinberg, Jeffrey E Barrick, Zizhen Yao, Adam Roth, Jane N Kim, Jeremy Gore, Joy Xin Wang, Elaine R Lee, Kirsten F Block, Narasimhan Sudarsan, Shane Neph, Martin Tompa, Walter L Ruzzo, and Ronald R Breaker. Identification of 22 candidate structured RNAs in bacteria using the CMfinder comparative genomics pipeline. *Nucleic Acids Res*, 35(14):4809–4819, 2007.
